# 2021 ASN Virtual Meeting Abstracts

**DOI:** 10.1177/17590914211039028

**Published:** 2021-10-06

**Authors:** The American Society of Neurochemistry

## Presidential Lectures

## PRES-01 HOW TO SPECIFY AND MODULATE THE NEUROTRANSMITTER INVENTORY OF AN ENTIRE NERVOUS SYSTEM

Oliver Hobert

*Columbia University, Biological Sciences, New York City, USA*

All neuron types in the nervous system are defined by a battery of molecular features that endow them with specific functional features. One key identity feature of a neuron is its neurotransmitter identity. To understand how neurons acquire their unique identity, it is of paramount interest to map neurotransmitter identity throughout the nervous system and to then study how neurotransmitter identity is genetically specified – and perhaps even modulated under specific conditions. I will describe here my laboratories efforts to comprehensively assign neurotransmitter identity to all neurons in the nervous system of the nematode Caenorhabditis elegans. I will then go on to describe the gene regulatory mechanisms that specify neuro-transmitter identity throughout the entire nervous system of the worm. I will describe the plasticity of neurotransmitter identity in the intriguing context of sexual maturation, showing that neurotrans-mitter deployments changes differentially in the same neuron type of the opposite sex and I will describe the molecular mechanisms that bring about such changes.

## PRES-02 SIGNALING PATHWAYS REGULATING OLIGODENDROCYTE DIFFERENTIATION

Wendy Macklin

*University of Colorado School of Medicine, Dept Cell and Developmental Biology, Aurora, USA*

Oligodendrocyte differentiation and myelination are tightly regulated, and the mechanistic Target of Rapamycin (mTOR) is one important regulator of CNS myelination. mTOR functions through two distinct complexes, mTOR complex 1 (mTORC1) and mTORC2, by binding to either Raptor or Rictor, respectively. mTORC1 has been studied extensively, whereas mTORC2 is less well studied, but known to impact the cytoskeleton. In order to establish whether mTORC1 and mTORC2 have unique functions during CNS myelination, we conditionally ablated either Raptor or Rictor in the oligo-dendrocyte lineage in vivo. Raptor deletion using the 2’,3’ cyclic nucleotide phosphohydrolase (CNP) promoter resulted in significant hypomyelination in spinal cord, which phenocopied related studies deleting mTOR itself. By contrast, when Rictor (mTORC2) was comparably deleted, it had little impact on myelination. When Rictor was selectively deleted in oligodendrocyte progenitor cells (OPCs), however, using the platelet derived growth factor receptor alpha promoter (PDGFRa-Cre), significantly reduced myelination was seen in corpus callosum, although not spinal cord. Thus, loss of Rictor/ mTORC2 impacts oligodendrocytes quite differently from that of mTORC1 or mTOR. We hypothesize that mTORC2 is crucial for early cytoskeletal changes, in particular the actin cytoskeleton. In Rictor-deleted cells, dramatic reductions in differentiation were seen, with reduced branching as cells mature. Other regulators of the cytoskeleton include p21-activated kinase1 (PAK1), which promotes OPC morphological differentiation and myelin production. Inhibiting PAK1 early in oligodendrocyte development decreases oligodendro-cyte morphological complexity and alters F-actin spreading at the tips of OPC processes. How these cytoskeleton modulators are regulated is a major focus of these studies. Supported by NIH R37 NS082203.

## PRES-03 CONTROLLING DEGRADATION OF MEMBRANE COMPONENTS IN NEURONAL DENDRITES: WASTE MANAGEMENT IN TIME AND SPACE

Bettina Winckler

*University of Virginia, Cell Biology, Charlottesville, USA*

Neuronal endosomes are essential for membrane receptor trafficking to and within dendrites and axons. Endosomes participate in a variety of signaling events, as well as regulating the rates of recycling and degradation. In neurons, endosomal trafficking is involved in receptor trafficking and various neuronal functions, such as synaptic plasticity and axon outgrowth. Dysfunctions of the endo-lysosomal system have been implicated in a number of neuro-degenerative conditions. As in other cell types, neuronal endosomes are regulated by a variety of endosomal regulators, such as Rab proteins, which localize to different endosomal compartments. We use a rapidly degrading membrane protein as a tool to study the regulation of degradation in time and space along dendrites. We show that degradative lysosomes are clustered in proximal dendrites and in the soma, leading to a steep spatial gradient of degradative capacity along dendrites. Terminal degradation of dendritic cargos requires Rab7-dependent transport to the soma, suggesting that trafficking of proteins from the dendrites is required for degradation in the soma. Proteostasis is thus spatially regulated in neurons.

## PRES-04 TARGETING BRAIN PROTEOSTASIS IN ALZHEIMER'S DISEASE

Sergio Ferreira^1^

*^1^ Federal University of Rio de Janeiro, Institute of Biophysics, Rio de Janeiro, Brazil*

*^2^ Federal University of Rio de Janeiro, Institute of Medical Biochemistry, Rio de Janeiro, Brazil*

Defective brain hormonal signaling has been associated with Alzheimer's disease (AD), a disorder characterized by synapse and memory failure. Irisin is an exercise-induced myokine released on cleavage of the membrane-bound precursor protein fibronectin type III domain-containing protein 5 (FNDC5), also expressed in the hippocampus. We found that FNDC5/irisin is reduced in AD hippocampi and cerebrospinal fluid, and in the brains of experimental AD models. Knockdown of brain FNDC5/irisin impairs hippocampal long-term potentiation and novel object recognition memory in mice. Conversely, boosting brain levels of FNDC5/irisin rescues synaptic plasticity and memory in AD mouse models. Peripheral overex-pression of FNDC5/irisin rescues memory impairment, whereas blockade of either peripheral or brain FNDC5/irisin attenuates the neuroprotective actions of physical exercise on synaptic plasticity and memory in AD mice. By showing that FNDC5/irisin is an important mediator of the beneficial effects of exercise in AD models, our findings place FNDC5/irisin as a novel agent capable of opposing synapse failure and memory impairment in AD.

## Symposia

## S-01 Protein Homeostasis Regulation in Neurodegeneration

S-01-01

**ADAPTING PROTEIN HOMEOSTASIS TO AMELIORATE DEGENERATIVE DISEASES**

Jeff Kelly

*Scripps, Chemistry, La Jolla, USA*

No abstract text has been provided.

S-01-02

**CHAPERONE NETWORK PERTURBATIONS MODULATE NEURODEGENERATION-RELATED PROTEIN AGGREGATION**

Reut Shalgi

*Technion - Israel Institute of Technology, Faculty of Medicine, Haifa, Israel*

Neurodegenerative diseases (ND), such as Huntington's disease, Parkinson's disease, ALS and others, are often caused by mutant proteins that misfold in the cell, leading to the accumulation of protein aggregates. Misfolded and aggregated proteins are usually targeted by an elaborate network of molecular chaperones, the master regulators of protein folding in the cell. However, in the case of ND, the chaperone network is unable to successfully handle these patho-logical aggregates. While several studies highlighted important roles for a handful of chaperones in modulating aggregation, the roles of individual chaperones in regulation of aggregation has not been systematically examined, and the potential function of many of them in aggregation modulation is still largely unknown.

Here we describe a novel framework to test the roles of chaperone network perturbations in modulation of ND-related protein aggregation. In combination with a chaperone screen, we are able to quantitatively measure the functional effect of chaperones in the net-work on protein aggregation phenotypes. Focusing on protein aggregation related to Huntington's disease and ALS, we identified multiple different chaperones that significantly alleviate protein aggregation, while many others aggravate aggregation phenotype. Interestingly, aggregate types related to different NDs were rescued or aggravated by different chaperones. Surprisingly, different naturally occurring isoforms of some chaperones show differential functional effects on aggregation modulation. We investigated the roles of these isoforms in aggregation rescue, and revealed underlying principles of their roles in regulating the phenotype of different aggregate types.

Together, our data unraveled the regulatory role of chaperone networks in dealing with protein aggregates, towards the ultimate goal of understanding how potential network rewiring could defend cells against ND-related protein aggregation, and provided future nodes for therapeutic intervention.

S-01-03

**MECHANISM OF Α-SYNUCLEIN AMYLOID FIBRIL DISAGGREGATION BY THE HUMAN HSC70 CHAPERONE MACHINERY**

Bernd Bukau^1^

*^1^ University of Heidelberg, Center For Molecular Biology of Heidelberg University, Heidelberg, Germany*

*^2^ German Cancer Research Center (DKFZ), Division of Chaperones and Proteases, Heidelberg, Germany*

*^3^ DKFZ-ZMBH Alliance, Member of the DKFZ-ZMBH Alliance, Heidelberg, Germany*

Parkinson's disease (PD) is a debilitating neurodegenerative condition linked to the gradual accumulation of α-synuclein amyloid inclusions in the brain. The progressive conversion of monomeric soluble α-synuclein into toxic oligomeric and fibrillar species results in the formation of highly stable, ordered aggregates that constitute atypical substrates for the cellular protein quality control machinery. The constitutive human Hsp70 chaperone (Hsc70), assisted by co-chaperones DNAJB1 and Apg2, generates a powerful ATP-dependent disaggregation activity that efficiently resolubilizes α-synuclein amyloid fibrils *in vitro*. Combining NMR spectroscopy, equilibrium and kinetic binding experiments and disaggregation activity assays, we dissect the functional cycle of Hsc70 to build a detailed picture of chaperone action on an amyloid substrate. Our data provide crucial new insights into the mode of action of Hsc70 on amyloid fibrils and highlights the essential role of co-chaperones in the activity of Hsc70.

S-01-04

**UNDERSTANDING PROTEOSTASIS DISFUNCTION AND AGGREGATE MANAGEMENT IN NEURODEGENERATIVE DISEASE**

Judith Frydman

*Stanford University, Biology and Genetics, Stanford, USA*

Achieving correct protein folding and quality control is essential for normal cellular function. The accumulation of misfolded proteins is emerging as central to a wide range of disease states, including many neurodegenerative disorders such as Huntington's and Prion Disease. Molecular chaperones are a diverse family of enzymes that monitor and maintain all aspects of protein homeostasis. The role of chaperones in protein quality control functions will be discussed.

Chaperones assist the folding of newly translated and stress-denatured proteins, as well as affects protein quality control. A stress-inducible chaperone network protects cells from environmental stress and assists quality control. These chaperones also communicate with the ubiquitin-proteasome pathway to clear misfolded proteins from the cell. Protein quality control in the eukaryotic cytosol relies on the sequestration of misfolded cytosolic proteins in specific quality control compartments. Our studies of chaperone function provide a framework to understand the link between protein misfolding and aggregate management in neuro-degenerative disease.

## S-02 Recent Advances in Brain Lipid Research in Health and Diseases

S-02-01

**LIVER LOCALIZED FABP-1 INFLUENCES BRAIN ENDOCANNABINOID AND ARACHIDONIC ACID LEVELS**

Eric Murphy

*University of North Dakota, Biomedical Sciences, Grand Forks, USA*

Fatty acid binding protein-1 (FABP1) is localized in liver and its expression in L-cells demonstrates its important role in cellular fatty acid uptake and trafficking. In addition, its expression in L-cells increases phospholipid mass, including an increase in plasmalogen mass, suggesting an important role in phospholipid biosynthesis. In the brain, there are three FABP expressed, FABP3 found in neurons, FABP5 found in neurons and glia, and FABP7 localized in astrocytes. We have previously demonstrated that FABP3 has an important, yet ill-defined role in maintaining plasmalogen levels in the brain. Further, our work demonstrates that FABP3 is important for arachidonic acid (20:4n-6, ARA) uptake and trafficking in whole brain. Recently, in collaboration with the Schroeder laboratory at Texas A & M University, we demonstrate that in the absence of FABP1 expression in the liver, that brain 20:4n-6 level is increased, putatively via modulation of its level in serum. More importantly, lack of its expression also demonstrates an important role in brain endocannabinoid levels, mainly 2-arachidonoylglycerol (2-AG), although levels of other endocannabinoids are also impacted. This is important because the FABP1 T94A mutation, which is seen in individuals who suffer from non-alcoholic steatohepatitis, bio-chemically acts similar to the FABP1 null. This mutation is thought to be present in up to 25% of Americans. Collectively, our findings suggest that it is important to delineate the importance of this FABP1 mutation in influencing not only the brain 20:4n-6 levels, but also 2-AG levels in these individuals. This especially important due to the importance of the endocannabinoid system in satiety and other physiological functions controlled by the brain.

S-02-02

**YIN-YANG MECHANISMS REGULATING LIPID PEROXIDATION OF DOCOSAHEXAENOIC ACID AND ARACHIDONIC ACID**

Grace Sun

*University of Missouri, Dept Biochemistry, Columbia, USA*

Docosahexaenoic acid (22:6n-3, DHA) and arachidonic acid (20:4n-6, ARA) are two prominent polyunsaturated fatty acids (PUFA) in phospholipids in the Central Nervous System (CNS). These PUFAs are metabolically active partly through the “deacylation-reacylation” cycle mediated by phospholipases A2 (PLA2) and acyltransferases. Recent studies have provided evidence for metabolism of these PUFAs through different types of phos-pholipase A2 (PLA2), namely, the cytosolic phospholipase A2 (cPLA2) for release of ARA and the calcium-independent iPLA2 for release of DHA. These different PLA2s formed the basis of the Yin-Yang mechanism for metabolism of phospholipids with DHA and ARA. In turn, ARA is known for production of eicosanoids, which are largely pro-inflammatory, whereas docosanoids produced by DHA are neuroprotective and pro-resolving. Both PUFAs are also susceptible to lipid peroxidation mediated by oxygen free radicals and resulting in the production of 4-hydroxyhexenal (4-HHE) from DHA and 4-hydroxynonenal (4-HNE) from ARA. Although the physiological role for these peroxidation products have not been studied in detail, increases in 4-HNE have been linked to the cPLA2/ ARA pathway due to neuronal excitation, stroke, and traumatic brain injury. In contrary, increases in 4-HHE were linked to dietary supplementation with DHA. DHA supplementation not only results in increase in phospholipid species with (n-3) PUFA but also decrease in phospholipid species with (n-6) PUFA. Interestingly, while changes in (n-3)/(n-6) phospholipids due to dietary DHA could be shown in all brain regions, the related increase in 4-HHE appeared to occur mainly in the cerebral cortex and hippocampus, suggesting enrichment in lipid peroxidative activity in these brain regions. Since alkenyl aldehydes are metabolically active and can form adducts with proteins and nucleic acids, it is worth future studies to investigate whether changes in 4-HHE due to DHA supplementation may provide specific physiologic role in brain health and disease processes.

S-02-03

**LIPIDOMIC CHARACTERIZATION OF PRO-REPAIR LIPID PATHWAYS IN ALZHEIMER'S DISEASE AND MULTIPLE SCLEROSIS**

Ameer Taha

*University of California - Davis, Food Science and Technology, Davis, USA*

**Background:** Alzheimer's Disease (AD) and Multiple Sclerosis (MS) are neurodegenerative disorders that lack effective treatments and well-defined etiologies. Previous lipidomic studies have shown reductions in lipid mediators known to promote neuronal repair, in serum of AD and MS patients compared to unaffected controls. The cause(s) of these changes remain unknown.

**Hypothesis and objectives:** The present study tested the hypothesis that pro-repair lipid mediators are reduced in post-mortem brains of AD and MS patients because they are actively sequestered by brain esterified lipid pools.

**Methods:** Post-mortem pre-frontal cortex of AD and MS subjects, and unaffected controls, was submitted to lipidomic analysis with liquid chromatography coupled to mass-spectrometry following isolation of brain unesterified and esterified lipid pools.

**Results:** Compared to controls, pro-repair lipid mediator concentrations within neutral lipids were reduced in AD, but increased in MS. No significant changes were observed in unesterified lipid mediators.

**Conclusion:** This study provides new evidence of altered pro-repair lipid mediator turnover within pre-frontal cortex neutral lipids of MS and AD patients. Targeting this pathway might promote neuronal repair by increasing the availability of unbound, pro-repair lipid mediators in brain.

S-02-04

**BRAIN CERAMIDE ACCUMULATION: A NOVEL DRIVER OF NEURODEGENERATION IN ALZHEIMER'S DISEASE**

Juan Palavicini

*UT Health SA, Barshop and Medicine/Diabetes, San Antonio, USA*

To date effective disease modifying Alzheimer's disease (AD) therapies remain elusive. Thus, delaying the onset or progression of AD represents a major unmet health care need that is an urgent matter of public health. Better-elucidation of AD pathogenesis is a critical step forward towards developing more effective therapeutics. Although amyloid-β (Aβ) is considered the central neurotoxic component of AD pathogenesis, it accumulates decades before the onset of AD, and by itself is not sufficient to cause dementia. Unraveling the major molecular mechanisms downstream of Aβ that drive neurodegeneration represents a critical knowledge gap that remains to be elucidated.

Dr. Alzheimer noted remarkable glial lipid granule accumulation.

Despite these early observations, glial lipoid deposits have been mostly neglected by the literature for decades. More recently, human genetic studies also point to lipid metabolism as a major player in AD etiology. Despite century-old and recent mounting evidence associating lipid metabolism with AD, and the fact that the brain is the richest organ in terms of lipid content and diversity, lipids are commonly “forgotten” by the AD field. Ceramides, the central core molecules in the metabolism of sphingolipids, have been established as a potent bioactive agent involved in multiple signaling pathways. Ceramide accumulation in AD was first described almost two decades ago, a finding subsequently confirmed by multiple laboratories. Despite strong evidence implicating impaired ceramide metabolism with AD, the molecular mechanisms underlying impaired ceramide homeostasis in the brain remain poorly understood.

We provide novel preliminary data demonstrating that the two classical neuropathological hallmarks of AD (Aβ and tau pathologies), as well the greatest known AD risk factor (aging), independently lead to accumulation of brain ceramides, particularly very long chain (VLC) ceramide species.

## S-03 The Central Role of Glia in Regulating Responses to CNS Infection

S-03-01

**EXPLORING THE ROLES OF MICROGLIA IN HOST DEFENSE AND DISEASE IN RESPONSE TO INFECTION OF THE CNS WITH A NEUROTROPIC CORONAVIRUS**

Thomas Lane^1^, Amber Syage^1^, Vrushali Mangale^2^, Yuting Cheng^1^, Dominic Skinner^1^, Atakan Ekiz^2^, Ryan O'Connell^2^, Kim Green^1^

*^1^ University of California, Irvine, Department of Neurobiology & Behavior, Irvine, USA*

*^2^ University of Utah, Department of Pathology, Salt Lake City, USA*

The present study examines functional contributions of microglia in host defense, demyelination, and remyelination following infection of susceptible mice with a neurotropic coronavirus. Treatment with PLX5622, an inhibitor of colony stimulating factor 1 receptor (CSF1R) that efficiently depletes microglia, prior to infection of the central nervous system (CNS) with the neurotropic JHM strain of mouse hepatitis virus (JHMV) resulted in increased mortality compared with control mice that correlated with impaired control of viral replication. Single cell RNA sequencing (scRNASeq) of CD45+ cells isolated from the CNS revealed that PLX5622 treatment resulted in muted CD4+ T cell activation profile that was associated with decreased expression of transcripts encoding MHC class II and CD86 in macrophages but not dendritic cells. Evaluation of spinal cord demyelination revealed a marked increase in white matter damage in PLX5622-treated mice that corresponded with elevated expression of transcripts encoding disease-associated proteins Osteopontin (Spp1), Apolipoprotein E (Apoe), and Triggering receptor expressed on myeloid cells 2 (Trem2) that were enriched within macrophages. In addition, PLX5622 treatment dampened expression of Cystatin F (Cst7), Insulin growth factor 1 (Igf1), and lipoprotein lipase (Lpl) within macrophage populations which have been implicated in promoting repair of damaged nerve tissue and this was associated with impaired remyelination. Collectively, these findings argue that microglia tailor the CNS microenvironment to enhance control of coronavirus replication as well as dampen the severity of demyelination and influence repair.

S-03-02

**STAPHYLOCOCCUS AUREUS CRANIOTOMY INFECTIONS ALTER GLIAL RESPONSIVENESS**

Tammy Kielian

*University of Nebraska Medical Center, Pathology and Microbiology, Omaha, USA*

Neurosurgery to relieve life-threatening edema (decompressive craniectomy) or gain temporary access to the brain for tumor resection (craniotomy) requires removal of a portion of the skull (i.e. bone flap). The incidence of infection after craniotomy/craniectomy ranges from 1-3%, with approximately half caused by *Staphylococcus aureus* (*S. aureus*), which forms a biofilm on both surfaces of the bone flap. Biofilms are bacterial communities encased in a self-produced matrix that are recalcitrant to antibiotics due to their metabolic dormancy. Our laboratory has developed a mouse model of *S. aureus* craniotomy-associated biofilm infection that is typified by a unique immune compartmentalization, namely preferential neutrophil recruitment in the subcutaneous galea, whereas monocytes are more prominent in the brain parenchyma. Granulocytic-myeloid-derived suppressor cells (G-MDSCs), an immature myeloid population with anti-inflammatory properties, are present in both compartments, but most abundant in the galea. This immune profile is associated with bacterial persistence out to 9 months post-infection, which is reflective of biofilm growth. We have utilized single cell RNA-sequencing (scRNA-seq) to reveal the complex transcriptional heterogeneity of resident microglia and infiltrating monocytes in the brain in addition to transcriptionally diverse granulocyte subsets in the subcutaneous galea and bone flap during craniotomy infection. In the brain, trajectory analysis identified the transition of microglia from a homeostatic/anti-inflammatory to pro-inflammatory and proliferative populations. Microglia augment oxidative metabolism in response to *S. aureus* biofilm without a significant increase in glycolysis, which may account for their limited ability to attenuate *S. aureus* burden in the brain during craniotomy infection. Analysis of tissues from patients with craniotomy infection have revealed similar inflammatory attributes to the mouse model, demonstrating its utility for developing novel therapeutic strategies. Supported by the NIH National Institute of Neurological Disorders and Stroke (R01NS107369).

S-03-03

**DEFENSE OF THE BRAIN BY MICROGLIA AND OTHER CNS MYELOID CELLS**

Dorian McGavern

*National Institutes of Health, National Institute of Neurological Disorders & Stroke, Bethesda, USA*

The central nervous system is inhabited by a specialized ensemble of myeloid cells that are highly dynamic and survey their immediate surroundings, which include the parenchyma, perivascular spaces, pia mater, dura mater, and choroid plexus. These cells are long-lived, help maintain homeostasis, participate in reparative responses following injury, and protect the CNS from invading microbes. The CNS is also surveyed and sometimes invaded by circulating, blood-derived myeloid cells that can contribute to diametrically opposed outcomes, like vascular breakdown vs. repair, depending on the inflammatory context. This lecture will focus on recent developments in our understanding of CNS myeloid cells, both resident and blood-derived. A special emphasis will be placed on how myeloid cells specifically survey and respond in the meninges vs. brain parenchyma to ward off viral challenges as well as the imprint left behind long after these pathogens are cleared.

S-03-04

**THE MULTI-FACETED ROLE OF GLIA IN THE PARASITIZED BRAIN**

Emma Wilson^1^, Edward Vizcarra^1^, Will Agnew-Svoboda^2^, Kristina Bergersen^1^, Todd Fiacco^2^, Martin Riccomagno^2^

*^1^ University of California, Riverside, Biomedical Sciences, Riverside, USA*

*^2^ University of California, Riverside, Department of Molecular Cell Biology and Neuroscience, Riverside, USA*

The common protozoan parasite, *Toxoplasma gondii*, exists for the lifetime of the host within neurons as slow-replicating cysts. Immune deficiency of the host leads to fatal encephalitis highlighting the requirement for continuous inflammation in the brain for protection. In the immune-competent host, significant changes in neuro-chemistry are linked to sub-clinical neuronal pathology, changes in host behavior and enhanced correlations to neurological disease. The Wilson lab aims to understand the alterations in the infected brain that could cause enhanced disease susceptibility while ultimately controlling the resident parasite and inflammatory response. Analysis of gene expression over the course of infection suggests a stabilization by week 8 with signatures of anti-inflammatory mechanisms and neuronal repair. An early and consistent component of infection-induced change in the brain is the presence of reactive astrocytes. Using a novel reporter system for identifying reactive astrocytes we can demonstrate significant heterogeneity during infection. Elucidating the function of reactive astrocytes during chronic infection and inflammation will be critical for our understanding of a balanced and protective immune response in the brain.

## S-04 Molecular Mechanisms Underlying Heterogeneity in Depression

S-04-01

**STRESS-INDUCED VASCULAR CHANGES DRIVING DEPRESSION VS STRESS RESILIENCE**

Caroline Menard^1^, Katarzyna Dudek^1^, Laurence Dion-Albert^1^, Fernanda Neutzling Kaufmann^1^, Ellen Tuck^1,2^, Ellen Doney^1^, Manon Lebel^1^

*^1^ CERVO Brain Research Center, Université Laval, Department of Psychiatry and Neuroscience, Quebec, Canada*

*^2^ Trinity College Dublin, Smurfit Institute of Genetics, Dublin, Ireland*

Major depressive disorder (MDD) is a chronic and recurrent psychiatric condition characterized by depressed mood, social isolation and anhedonia. It will affect 20% of individuals with considerable economic impacts. Unfortunately, 30-50% of depressed individuals are resistant to current antidepressant treatments. MDD is twice as prevalent in women and associated symptoms are different. Depression's main environmental risk factor is chronic stress, and women report higher levels of stress in daily life. However, not every stressed individual becomes depressed, highlighting the need to identify biological determinants of stress vulnerability but also resilience. Based on a reverse translational approach, rodent models of depression were developed to study the mechanisms underlying susceptibility vs resilience. Indeed, a subpopulation of animals can display coping mechanisms and a set of biological alterations leading to stress resilience. The etiology of MDD is multifactorial and involves several physiological systems. Exacerbation of immune responses from both innate and adaptive systems is observed in depressed individuals and mice exhibiting depression-like behaviours. Increasing attention has been given to neurovascular health since higher prevalence of cardiovascular diseases is found in MDD patients and inflammatory conditions are associated with depression, treatment resistance and relapse. I will provide an overview of vascular and immune changes associated with stress vulnerability vs. resilience in mouse models of depression and MDD patients. Lack of treatment efficacy suggests that neuron-centric treatments do not address important causal biological factors and better understanding of stress-induced adaptations, including sex differences, could contribute to develop novel therapeutic strategies including personalized medicine approaches.

S-04-02

**SEX SPECIFIC IMMUNE MECHANISMS OF DEPRESSION: TRANSLATING THERAPEUTIC TARGETS**

Georgia Hodes

*Virginia Tech, Integrated Life Science Building, Room 2009, Blacksburg, USA*

There is a bi-directional relationship between the immune system and major depressive disorder (MDD). Little is known about mechanisms contributing to the higher incidence of inflammatory and stress-related illness in females. We used multiplex ELISA to quantify plasma cytokine protein expression regulated by treatment resistant and non-treatment resistant depression in men and women compared to age matched (21-55 years) healthy controlls. To test the ability of mouse stress models to recapitulate the cytokine changes found in depressed individuals we examined circulating levels of cytokines for male and female mice exposed to 6-day variable stress and 28-day variable stress. Additional studies examined cytokine levels following 6 days of variable stress in four-core genotype mice which allow dissociation of genetic sex from gonadal sex. We found there are sex differences in the peripheral immune response to MDD or stress that transcend species. Women with treatment resistant MDD have a sex specific profile of T-cell related immune response suggestive of an autoimmune disease. Women with treatment resistant depression and men that are not treatment resistant show activation of cytokines released by the innate immune system. Rodent stress paradigms also produced a similar pattern for their regulation of peripheral cytokines. Sex differences in immune profiles were highly dependent on genetic sex although we did find some effects of gonadal sex in a subset of cytokines. These data demonstrate that segregation by sex and treatment resistant status are important for understanding the impact of depression or stress on cytokine profiles in humans and rodents.

S-04-03

**COULD STRESSING OUR GUT MICROBES MAKE US DEPRESSED?**

Marie-Claude Audet

*University of Ottawa, Nutrition Sciences, Ottawa, Canada*

Despite the increase of the psychological and economic burden attributable to depressive illnesses, available pharmacological interventions have variable success rates in reducing symptoms. The development of effective antidepressant strategies has been limited in part by our incomplete understanding of the mechanisms underlying depression, especially given that the illness is heterogeneous. Appreciable evidence suggest that intestinal microbial communities could play a key role in the pathogenesis of mental illnesses, including depression. Our work and that of others has shown that in addition to increase pro-inflammatory cytokines peripherally and centrally, social stressors that elicit depressive-like states in adult mice disturb the gut microbiota. Using a chronic social stressor, we reported that long-lasting bacterial changes in male mice were linked to their susceptibility to the depressive-like effects of the stressor and to brain expression of pro-inflammatory cytokines. Chronically stressed mice also had long-lasting pro-inflammatory cytokine elevations in the small intestine, which were accompanied by a decreased expression of tight junction proteins. In subsequent experiments, we demonstrated that gut microbial communities and expression of inflammatory and barrier integrity markers along the gut-brain axis were altered in mouse offspring born to stressed dams, and that changes in abundance of specific bacteria in humans were related to severity of depressive symptoms only in individuals that had experienced trauma in their childhood. Although our observations in mice models and human populations are correlational, they suggest that perturbations of gut microbial colonization patterns and of inflammatory processes along the gut-brain axis during development could be key factors in conferring increased risk for depressive illnesses. In a context where treatments for depression are not optimal, the identification of early processes that could promote vulnerability to the depressive effects of stressors could represent a useful tool in the design of interventions to prevent and/or alleviate depression.

## S-05 Glia Cells in Neuronal Circuitry Function: Does Sex Matter?

S-05-01

**CRITICAL ROLES FOR MICROGLIA DURING CRITICAL PERIODS OF BRAIN DEVELOPMENT**

Margaret McCarthy

*University of Maryland School of Medicine, Department of Pharmacology, Baltimore, USA*

Multiple brain regions develop differently in males versus females in response to testicular steriod synthesis during the perinatal critical period for sexual differentiation. In the laboratory rat, astrocytes and microglia display sex differences in morphology, function and number in discrete brain regions. Within the developing medial amygdala the number of astrocytes surviving to the juvenile period is determined by microglial mediated phagocytosis, which occurs at significantly higher rates in neonatal males than females. The reduced number of astrocytes correlates with higher degrees of neuronal excitation which appears to specifically drive higher rates of rough and tumble play in young males. In the developing preoptic area, the number of neurons in the sexually dimorphic nucleus is also controlled by phagocytic activity of microglia, which are also influenced by immune system mast cells. Overall there is extensive cross talk between astrocytes, microglia and other immune cells of the brain which direct patterns of synaptogenesis, cell proliferation and cell survival, thereby altering the developmental trajectory of the brain distinctly in males and females.

S-05-02

**DARK MICROGLIA IN HEALTH AND DISEASE**

Marie-Eve Tremblay^2^

*^1^ CRCHU de Québec-Université Laval, Axe Neurosciences, Quebec, Canada*

*^2^ University of Victoria, Division of Medical Sciences, Victoria, Canada*

Discussion of our recent characterization of an ultrastructurally distinct microglial subset that is predominantly associated with pathological states, using a combination of immunocytochemical electron microscopy, array tomography, and focused-ion beam scanning electron microscopy with 3D reconstruction. The dark microglia are rare in healthy adult mice, but they become highly prevalent upon chronic stress, fractalkine signaling deficiency (CX3CR1 knockouts), aging, and Alzheimer's disease pathology, where they account for two-thirds of the normal microglial population. These cells display well-characterized markers of cellular stress, make extensive interactions with synapses and directly ensheath the basement membrane of capillaries. Recently, we also found evidence that these cells are similarly abundant during normal brain development, in the first postnatal weeks when synaptic pruning mainly takes place, and upon exposure to maternal immune activation, where they display sex differences. We additionally observed these cells in human postmortem brain samples. Overall, these findings indicate that dark microglia could represent a subset of cells that become stressed as a result of their hyperactive involvement with the remodeling of neuronal circuits across development, stress-induced plasticity, aging, and disease pathology.

S-05-03

**SEX DIFFERENCES IN HIV-RELATED COGNITIVE AND MOTORIC DISORDERS: A GLIO-CENTRIC PERSPECTIVE**

Pamela Knapp

*Virginia Commonwealth University, Dept Anat & Neurobiol, Richmond, USA*

Despite increased availability and efficacy of combination anti-retroviral therapy (cART), people living with HIV (PLWH) experience a constellation of CNS disturbances resulting in both cognitive and motor dysfunction. The overall prevalence of such symptoms has not decreased, although the severity has been reduced, and HIV-related dementia is uncommon. Some clinical studies show significant sex-related differences in types/severity of behavioral deficits observed in PLWH. Behavioral deficits must involve altered CNS neuronal function, although neurons themselves are not infected by HIV. Instead, they are bystander targets of viral toxins or inflammatory factors released by HIV-infected monocytes/microglia. These in turn affect the function of other glia and neurons, a cascade of events demonstrated in many culture and animal models. Among the multiple factors presumably involved in sex-related behavioral differences, we propose a role for differential glial reactivity and the resulting neuronal damage. We have modeled CNS effects of HIV using an inducible transgenic mouse expressing the HIV-1 Tat protein. In comparative studies, Tat+ males display greater deficits in both motor and cognitive/anxiety tests, although both sexes exhibit some altered behavior over time. After 2-week Tat exposure, male but not female mice exhibited heightened anxiety, altered fear extinction, and significant deficits in spatial memory. Related changes were seen in neuronal spine density and synaptic proteins in critical brain regions, concomitant with altered levels of the plasticity-related Arc protein. After 3-month exposure, both sexes showed similar reductions in open field ambulation, but males showed additional motor and anxiety deficits. Although CNS volumes and total neuron numbers were unchanged, males had higher levels of astrogliosis and microglial nitrosative stress, as well as more spine reduction and abnormal levels of excitatory and inhibitory pre- and post-synaptic proteins in some CNS regions. Overall, increased behavioral deficits in males correlated with more glial activation and synaptic damage, both of which are implicated in cognitive/motor impairments in patients. Results suggest that glia in males and females may respond differently to HIV, contributing to distinct behavioral outcomes between sexes. Support: DA024461/DA044939

S-05-04

**GLUTAMATE TRANSPORTERS AS REGULATORS OF CNS MYELINATION**

Babette Fuss^1^, Edna Suárez-Pozos^1^, Elizabeth Thomason^1^, Yu Par Myo^1^, Miguel Escalante^2,1^, Paul A. Rosenberg^3^, Jeffrey L Dupree^1^, Arturo Ortega^2^

*^1^ Virginia Commonwealth University, Anatomy and Neurobiology, Richmond, USA*

*^2^ Centro de Investigación y de Estudios Avanzados del Instituto Politécnico Nacional, Departamento de Toxicología, México DF, México*

*^3^ Boston Children's Hospital, F.M. Kirby Neurobiology Center, Boston, USA*

Myelination of central nervous system (CNS) axons enables efficient signal propagation via saltatory conduction, and it provides metabolic support to ensure axonal integrity. Thus, the developmental establishment of CNS myelin by a highly specialized cell type, the oligodendrocyte (OLG), represents a critical component of building a fully functional CNS. Current models propose that mye-lination can be modulated by axonal electrical activity that is mediated, at least in part, by vesicular release of the excitatory amino acid glutamate along unmyelinated axonal segments. While good progress has been made in defining the effects of axonally released glutamate on progenitor cells of the OLG lineage, much less is known about the glutamate-responses in differentiating and pre-mye-linating OLGs. We introduce here, sodium-dependent glutamate transporters as OLG expressed glutamate-responsive transmembrane proteins modulating CNS myelination. In our earlier studies, we demonstrated that activation of sodium-dependent glutamate transporters in differentiating OLGs promotes process outgrowth and branching. This aspect of OLG maturation is driven by actin cyto-skeleton dynamics, and it is considered a critical step toward the initiation of CNS myelination. Mechanistically, activation of glutamate transport was found to initiate a signaling cascade involving reverse mode activation of sodium-calcium exchange and calcium influx. In continuing studies, we have started to investigate CNS myelination *in vivo* in conditional *Glt-1 (EAAT2/Slc1a2)* knockout mice and our data, thus far, suggest a developmental effect that may be seen preferentially in males. Hence, the OLG expressed glutamate transporter GLT-1 emerges as a novel player modulating CNS mye-lination by a mechanism that controls the morphological changes necessary for efficient initiation of CNS myelination and that may generate a sexually dimorphic vulnerability during a critical window of development.

## S-06 Epigenetic Approaches in Addiction Research: Interrogation of Molecular Mechanisms and Potential Treatment Strategies

S-06-01

**MIMICKING METHAMPHETAMINE USE DISORDER WITH FOOTSHOCK PUNISHMENT: EPIGENETIC AND TRANSCRIPTIONAL CONSEQUENCES IN THE RAT BRAIN**

Jean Lud Cadet

*NIDA Intramural Research Program, Molecular Neuropsychiatry Research Branch, Baltimore, USA*

Methamphetamine use disorder (MUD) is a major public health problem throughout the world. MUD is associated with severe neuro-psychiatric complications. Investigations that have focused on the effects of methamphetamine on catecholaminergic systems have not helped to develop therapeutic agents against MUD. Therefore, we have hypothesized that the manifestations of MUD may be secondary to perturbations that involve long-lasting epigenetic, transcriptional, and/or biochemical changes in the brain. Rats were trained to self-administer methamphetamine over a period of a month. Thereafter, the animals received contingent shocks while self-administering the drug. Rats were then euthanized and used in various biochemical, transcriptional, or epigenetic experiments at various timepoints after withdrawal from methamphetamine self-administration. We measured genome-wide transcriptional changes using Illumina 22K Rat microarrays. We used a deep sequencing approach with Illumina HiSeq2500 to identify potential alterations in DNA hydroxymethylation. Rats exposed to methamphetamine during self-administration experiments escalated their methamphetamine intake. Contingent shocks separated methamphetamine-exposed rats into punishment-resistant (PR, addicted) rats that took methamphetamine compulsively and punishment-sensitive (non-addicted) rats that decreased their lever pressing. Genome-wide DNA sequencing revealed increased DNA hydroxymethylation of several potassium channels, with PCR showing increased expression of their mRNAs in the nucleus accumbens of PS rats. Thus, prolonged exposure to compulsive methamphetamine self-administration in the presence of adverse consequences does cause long-lasting epigenetic and transcriptional perturbations that could be targeted to treat MUD. Supported by NIDA Intramural Research Program

S-06-02

**ROLE OF DNA HYDROXYMETHYLATION IN METHAMPHETAMINE ADDICTION**

Subramaniam Jayanthi

*National Institute of Drug Abuse, Molecular Neuropsychiatry, Baltimore, USA*

Methamphetamine (METH) use disorder (MUD) is a very serious, potentially lethal, biopsychosocial disease. Exposure to METH causes long-term changes to brain regions involved in reward processing and motivation, leading vulnerable individuals to engage in pathological drug-seeking and drug-taking behavior that can remain a lifelong struggle. According to the U.S. National Survey on Drug Use and Health, 375,000 Americans (aged 18-25) and 1.2 million (aged 26 or older) are active METH users. Despite the widespread use of METH, much remains to be done to develop effective therapeutic approaches to treat MUD, a fact that is, in part, related to a relative lack of understanding of the molecular neurobiology of this brain disease.

Genome-wide analyses such as microarrays and next-generation sequencing following acute and chronic exposure to METH have demonstrated striking changes in gene expression and epigenetic regulatory mechanisms that lead to alterations in diverse cellular functions characteristic of MUD and, ultimately altered behavior. Though understudied, recent observations have identified METH-induced changes in epigenetic markers including DNA methylation and histone modifications. Methylation of CpG (5mC ) islands blocks transcription factor binding, binding of MBD proteins, and recruitment of co-repressor proteins. These processes result in condensed and transcriptionally repressive chromatin. In contrast, hydroxymethylation of CpG (5hmC) which is differentially enriched in the brain in comparison to other tissues has been proposed to promote transcription via DNA demethylation. We found that METH-addicted rats showed differential DNA hydroxymethylation in the nucleus accumbens (NAc) in comparison to both control and abstinent rats. These changes occurred mostly at intergenic sites located on long interspersed elements (LINEs). Moreover, we observed differential DNA hydroxymethylation and increased expression of specific members of potassium channels in the NAc that may promote abstinence from drug taking behaviors. These results suggest a potentially novel therapeutic approach to the care of patients who suffer from methamphetamine use disorder.

S-06-03

**NOT ALL STIMULANT DRUGS ARE THE SAME: INSIGHTS FROM HISTONE ACETYLATION AND DEACETYLATION**

Veronica Bisagno

*CONICET, ININFA, Buenos Aires, Argentina*

Psychostimulants produce different behavioral and neurobiological profiles. For example, some stimulants cause neuroplastic changes leading to addiction and cognitive deficits while others enhance cognition. Epigenetic mechanisms are known contributory factors to drug-induced neuroadaptations. These epigenetic changes include dysregulation of histone deacetylases (HDACs) proposed to participate in maintaining aberrant transcriptional programs associated with altered cognitive functions and behaviors. We have previously reported that the psychostimulant drugs modafinil and methamphetamine differentially influence the acetylation status of histones 3 and 4 at promoters of HDACs in the mouse prefrontal cortex. HDACs are divided into zinc-dependent [class I (HDAC1,2,3,8), class IIa (HDAC4,5,7,9), class IIb (HDAC6,10), and class IV (HDAC11)] and NAD-dependent [class III (sirtuins1-7)] enzymes. Interestingly, class IIa (4, 5, 7, 9) HDACs get phos-phorylated and exported from the nucleus to cytoplasm after various stimuli. The phosphorylation state of Class IIa HDACs can impact their enzymatic activity. In this talk, we will present evidence that psychostimulants (modafinil, methamphetamine and caffeine) differently influence the expression of Class IIa HDAC in vivo (mouse prefrontal cortex and dorsal striatum) and in vitro (N2a cell line). The differential impact of these psychostimulant drugs on these HDACs might offer a partial explanation for some of their divergent behavioral effects and therapeutic profiles. Continued understanding of how psychostimulants drugs alter epigenetic regulators is essential for identifying transcriptional changes that occur after exposure to these drugs.

S-06-04

**METHAMPHETAMINE SELF-ADMINISTRATION UNDER PUNISHMENT IS ASSOCIATED WITH SELECTIVE ACTIVATION OF NG**

Oscar Torres^1^, Subramaniam Jayanthi^2^, Michael McCoy^2^, Jean Lud Cadet^2^

*^1^ San Diego Mesa College, Behavioral Science, San Diego, USA*

*^2^ National Institute on Drug Abuse, Molecular Neuropsychiatry Research Branch, Baltimore, USA*

Methamphetamine (METH) addicts lose control over drug consumption despite suffering multiple adverse consequences. To mimic these negative events, we introduced a rat model of METH self-administration (SA) with response-contingent punishment. These procedures allowed us to distinguish between two addiction-like phenotypes in rats, those that sustained METH SA despite negative consequences (shock-resistant, SR) and rats that significantly reduced their METH intake (shock-sensitive, SS). Here, we further developed our adverse consequence model and examining incubation of METH craving by measuring cue-induced drug seeking. Transcriptional and translational alterations in the striatum were also examined. Male Sprague-Dawley rats were trained to self-administer METH (0.1mg/kg/injection) or saline intravenously (i.v.) during twenty-two 9-h sessions that consisted of 3 separate 3-h sessions separated by 30 minutes. Subsequently, rats were subjected to incremental footshocks during thirteen additional 9-h METH SA sessions. Cue-induced drug craving was then assessed at 2 and 21 days after the footshock phase. Nine days following the last drug seeking test, the dorsal striatum was dissected and processed for gene expression and protein analyses. All rats escalated their METH intake, with both SR and SS phenotypes showing similar drug taking patterns during SA training. However, SR rats continued their METH intake despite negative consequences, and showed greater cue-induced drug craving following withdrawal. PCR arrays revealed significant differences in neurotrophins and their receptors between the 2 phenotypes. Protein analysis revealed that pTrkA and nerve growth factor receptor expression were increased only in the SS phenotype. Moreover, SS rats showed increased abundance of several phos-phorylated proteins known to participate in Ras/Raf/MEK/ERK signaling cascade including cRaf, ERK1/2, MSK1, and CREB. Taken together, our adverse consequence-based model highlights the possibility of identifying rats by addiction-like phenotypes, subsequent vulnerability to relapse-like behaviors, and differential changes in intracellular signaling cascade activated by nerve growth factor-TrkA/p75NTR interactions.

## S-07 Iron Metabolism in the CNS: Implications for Neurodegenerative and Demyelinating Diseases

S-07-01

**H-FERRITIN IRON DELIVERY TO OLIGODENDROCYTES AND MYELINATION DURING DEVELOPMENT**

James Connor, Brian Chiou, Elizabeth Neely, Quinn Wade

*Penn State University, College of Medicine, Hershey, USA*

Iron deficiency is the most common nutrient deficiency worldwide and affects approximately one third of infants and children. Iron delivery to the developing brain is essential for energy and metabolic support needed for processes such as myelination and neuronal development. It is well-known that oligodendrocytes (OGs) are the highest iron staining cells in the brain and the ability of these cells to take up a substantial amount of iron during development is necessary for proper myelination. Inadequate iron delivery frequently results in severe neurological deficits that are attributed to hypomyelination. Our laboratory and others have identified H-ferritin (Hft), traditionally considered solely an iron storage protein, as the primary iron delivery protein to OGs, and this study if the first to demonstrate Hft delivery during development. We have also identified a novel receptor on rodent OGs, T-cell immunoglobulin and mucin domain-containing protein-2 (Tim-2), that H-ferritin utilizes to act on OGs. In this study, we observed that post-natal day 14 mice have the highest levels of uptake of Hft-bound iron, which reflects the developmental pattern of myelination and OG maturation. Consistent with these results, we confirmed the presence of Tim-2 for iron acquisition via extracellular Hft on OGs. Our data indicate that iron uptake is tightly regulated and suggests that there is a set-point for iron delivery that is established during development and maintained throughout adulthood. We propose there is a critical window during which the brain is growing that is amenable to relatively high levels of iron uptake. The data from this study could have significant implications in the clinical treatment of brain iron deficiency during development.

S-07-02

**MANAGING BRAIN IRON AT THE BLOOD-BRAIN BARRIER**

Daniel Kosman

*University at Buffalo, Biochemistry, Buffalo, USA*

Aqueous Fe^2+^ is as neurotoxic as it is essential. This ‘yin-yang’ is an integral part of cerebral iron homeostasis. Key to this homeostasis is the transcellular flux of ferrous iron across the brain microvascular endothelial cells (hBMVEC) that establish the tight junctions characteristic of the brain microvasculature. Elucidation of the *molecular details* of this trafficking has lagged behind examination of the systemic outcomes associated with knockdown of a transporter likely involved in it. There are three of these proteins: the Zrt-, Irt-like solute uptake transporters, ZIP8 and ZIP14, and the Fe^2+^ efflux protein, Ferroportin (FPN). In essentially all these whole organism KOs, there is dysregulation of cerebral iron metabolism. However, this experimental paradigm typically can't tease apart the contributors to this pathophysiology, *e.g.* is hyper-accumulation due to whole body metal excess, or to a defect in *secretion* of abluminal metal back into the circulation. Using a well-validated blood brain barrier (BBB) model provided by hBMVECs in a transwell culture dish in which the ECs form a ZO-1/Occludin/Claudin-mediated monolayer that mimics the barrier properties of brain capillaries. These cells are polarized thus providing a unique model system in which to examine the following details of this transcellular metal flux. **1)** The relative Me^2+^-uptake activities at the apical and basal membranes. **2)** The relative Me^2+^-efflux activities from each cell face. **3)** The paracrine signaling between ECs and underlying glia that modulate these uptake and efflux activities **4)** A system in which the trajectory of Me^2+^ at the BBB can be quantified. **5)** A cell model in which systematic up-or down-regulation of these uptake and efflux proteins at the plasma membrane can be correlated quantitatively with changes in the Me^2+^ flux across this barrier. In this system, the role of soluble APP (sAPP) in iron trafficking at the BBB also has been delineated. A survey of key results is presented in this presentation.

S-07-03

**CELLULAR RESPIRATION AND IRON DEFICIENCY: TAKING A DEEP BREATH**

Juana Pasquini^1^, V Rosato Siri^1^, PV Martino Adami^2^, ME Guitart^1^, L Morelli^2^, J Correale^3^

*^1^ Univ. of Buenos Aires, Biol Chem., Sch. Pharm & Biochem, Buenos Aires, Argentina*

*^2^ Instituto de Investigeciones Bioquimicas de Buenos Aires, Laboratorio de Envejecimiento y Neurodegeneración, Buenos Aires, Argentina*

*^3^ FLENI, Instituto de Investigaciones Neurológicas Dr. Raúl Carrea, Buenos Aires, Argentina*

Argentina Iron deficiency (ID) represents one of the most prevalent nutritional deficits, affecting almost two billion people worldwide. Gestational iron deprivation constitutes a useful experimental model, as it induces hypomyelination due to oligodendroglial immaturity and thus allows the study of oligodendrocyte (OLG) requirements for progression to a mature myelinating state. As from gestational day 5, pregnant mice were fed either an ID diet (4mg iron/kg) or a control diet (40 mg iron/kg), and energy metabolism was assessed in primary cultures of OLG and astrocytes (AST) from newly born control and ID pups. In particular, oxygen consumption and extracellular acidification rates were measured using a Seahorse extracellular flux analyzer. Neither ID AST nor OLG showed significant differences in respiration driving ATP synthesis, respiration driving proton leak or non-mitochondrial respiration as compared to their respective controls. However, they both exhibited decreased spare respiratory capacity, indicating that maternal ID effectively induces mito-chondrial dysfunction. Absence of glycogen granules was observed in ID AST and an increase in ROS production was seen in ID OLG. Mitochondrial fission was increased in ID AST while fusion was prevalent in ID OLG. Electron microscopy also showed abnormal cristae in ID mitochondria. These findings further prove that the regulation of cell metabolism may impact cell fate decisions and maturation.

S-07-04

**IRON METABOLISM IN OLIGODENDROCYTES AND ASTROCYTES, IMPLICATIONS FOR MYELINATION AND REMYELINATION**

Pablo Paez

*SUNY at Buffalo, Department of Pharmacology and Toxicology, School of Medicine and Biomedical Sciences, Buffalo, USA*

Iron is a key nutrient for normal CNS development and function, thus, iron deficiency as well as iron excess may result in harmful effects in the CNS. Oligodendrocytes and astrocytes are crucial players in brain iron equilibrium. Oligodendrocytes are the cells with the highest iron levels in the brain which is linked to their highly metabolic needs associated with the process of myelination. In contrast, astrocytes do not have a high metabolic requirement for iron. However, these cells are in close contact with blood vessel and have a strong iron transport capacity. To study the mechanisms of iron metabolism in oligodendrocytes and astrocytes we have created several conditional knock-out mice in which proteins involved in iron uptake, release and storage, such as the divalent metal transporter 1 (DMT1), the ferritin heavy chain (Fth) and the transferrin receptor 1 (Tfr1) were postnatally ablated specifically in oligo-dendrocytes or astrocytes. To define the molecular mechanism of iron absorption and storage in oligodendrocytes, we have tested the function of these proteins in oligodendrocyte iron status and function during brain development as well as in the cuprizone model of myelin injury and repair. Furthermore, to stablish the role of astrocytes in brain iron absorption and distribution, the same proteins were specifically ablated in astrocytes in the postnatal, adult and aging mouse brain. Since normal CNS iron homeostasis is indispensable for proper oligodendrocyte maturation and myelination, understanding the molecular mechanisms of iron uptake, storage and efflux in oligodendrocytes and astrocytes is necessary for planning effective strategies for iron management during CNS development as well as for the treatment of demyelinating diseases.

## S-08 Glial Chloride Channels and Transporters: At the Intersection of Cell Volume and Brain Health

S-08-01

**THE CROSSTALK BETWEEN MLC1, GLIALCAM AND THE GLIAL CHLORIDE CHANNELS CLC-2 AND LRRC8 IN LEUKODYSTROPHIES**

Raul Estevez^1^

*^1^ University of Barcelona/IDIBELL, Physiological Sciences, L'Hospitalet, Spain*

*^2^ CIBERER, rare diseases, Madrid, Spain*

Leukodystrophies constitute a large group of genetic disorders primarily affecting CNS white matter. Within these, Megalencephalic leukoencephalopathy with subcortical cysts (MLC) is characterized by early-onset macrocephaly, epilepsy and cerebral white matter edema. It can be caused by mutations in two different genes: *MLC1*, which is more frequent, and *GLIALCAM*. MLC1 is a membrane protein of unknown functions while GlialCAM is an adhesion molecule that belongs to the immunoglobulin superfamily. GlialCAM works as an obligatory subunit of MLC1, being required for MLC1 endo-plasmic reticulum exit and targeting to astrocyte-astrocyte junctions. In addition, GlialCAM is further characterized as an auxiliary subunit of the ClC-2 chloride channel, targeting it to cell-cell junctions and modifying its functional properties. Furthermore, lack of MLC1 and GlialCAM reduce the current of the volume-regulated anion channel (VRAC). Here, we will summarized our last findings about the implication of a reduced function of the ClC-2 and VRAC channels in the pathophysiology of MLC and the way these channels can be regulated.

S-08-02

**NKCC1 ION TRANSPORTER IN BRAIN INJURY**

Dandan Sun^1^

*^1^ University of Pittsburgh, Neurology, Pittsburgh, USA*

*^2^ VAPHS, GRECC, Pittsburgh, USA*

Stroke with comorbid hypertension (HT) is associated with worsened outcome and not improved with post-stroke blood pressure (BP) lowering therapies. HT and arteriosclerosis stenosis are the most significant risk factors for dementia epidemics. However, currently, there are no therapies for symptomatic treatment of vascular dementia or reducing HT-associated worsened stroke outcomes. The serine-threonine WNK kinase family [with no lysine (K)], and its two downstream kinases SPAK (the STE20/SPS1-related proline/alanine-rich kinase) and OSR1 (oxidative stress-responsive kinase 1) activate brain Na^+^-K^+^-Cl^−^ cotransporter isoform 1 (NKCC1) via protein phosphorylation. Ischemic stroke stimulates the WNK-SPAK kinases-mediated phosphorylation of NKCC1 protein, which contributes to intracellular Na^+^ and Cl^−^ overload, cytotoxic edema, and excitotoxicity ischemic cell damage. Stimulation of brain WNK-SPAK-NKCC1 complex in angiotensin II (Ang II)-induced hypertensive mice led to worsened ischemic stroke brain damage. Stroke triggered 2-5 fold upregulation of WNK proteins (isoforms 1, 2, 4), SPAK/OSR1 and NKCC1 proteins in the Ang II-infused hypertensive brains after stroke, which were located in cortical neurons and associated with nuclear translocation of phospho-NF-kB protein and increased NF-κB recruitment on *Wnk1, Wnk2, Wnk4, Spak* and *Nkcc1* gene promoters. Post-stroke administration of SPAK inhibitor ZT-1a significantly reduced WNK-SPAK-NKCC1 complex activation, brain lesion, and neurological function deficits in the Ang II hypertensive mice without lowering BP. We conclude that the Ang II-induced stimulation of NF-kB transcriptional pathway is involved in upregulating brain WNK-SPAK-NKCC1 cascade and leads to worsened ischemic stroke outcomes, indicating WNK-SPAK-NKCC1 complex as therapeutic target for stroke therapy with comorbid HT.

*This research was supported by VA Grant I01BX002891 (DS), IK6 BX005647 (DS), and NIH RO1NS038118 (DS).*

S-08-03

**GLUTAMATE-RELEASING SWELL1/LRRC8A CHANNEL IN ASTROCYTES MODULATES SYNAPTIC TRANSMISSION AND PROMOTES STROKE DAMAGE**

Zhaozhu Qiu, Jianan Chen, Junhua Yang, Jiachen Chu, james Osei-Owusu

*Johns Hopkins University, Physiology, Baltimore, USA*

By releasing glutamate, astrocytes actively regulate synaptic transmission and also contribute to excitotoxicity in neurological diseases. However, the mechanisms of astrocytic glutamate release have been debated. Here, we report non-vesicular release of glutamate through the glutamate-permeable volume-regulated anion channel (VRAC). Both cell swelling and receptor stimulation activated astrocytic VRAC, which requires its only obligatory subunit, Swell1 (a.k.a Lrrc8a). Astrocyte-specific *Swell1* knockout mice exhibited impaired glutamatergic transmission due to the decreases in presynaptic release probability and ambient glutamate level. Consistently, the mutant mice displayed hippocampal-dependent learning and memory deficits. During pathological cell swelling, deletion of astrocytic Swell1 attenuated glutamate-dependent neuronal excitability and protected mice from brain damage after ischemic stroke. Our identification of a new molecular mechanism for channel-mediated glutamate release establishes a role for astrocyte-neuron interactions in both synaptic transmission and brain ischemia. It provides a rationale for targeting VRAC for the treatment of stroke and other neuro-logical diseases associated with excitotoxicity.

S-08-04

**FUNCTIONAL HETEROGENEITY AND PHYSIOLOGICAL ROLES FOR HETEROMERIC LRRC8 CHANNELS IN THE CNS**

Alexander Mongin^1^, Corinne Wilson^1^, Preeti Dohare^1^, Shaina Orbeta^1^, Julia Nalwalk^1^, Yunfei Huang^1^, Russell Ferland^2^, Rajan Sah^3^, Annalisa Scimemi^4^

*^1^ Albany Medical College, Department of Neuroscience and Experimental Therapeutics, Albany, NY, USA*

*^2^ University of New England, Department of Biomedical Sciences, Biddeford, ME, USA*

*^3^ Washington University School of Medicine, Department of Internal Medicine, St. Louis, MO, USA*

*^4^ State University of New York at Albany, Department of Biology, Albany, NY, USA*

Volume-regulated anion channels (VRACs) are a group of ubiquitously expressed ion channels that are activated by cell swelling and permeable to a variety of negatively charged and net-neutral molecules. Their broader physiological significance in the brain remains under investigation. VRACs are formed by the five proteins from the leucine-rich repeat-containing family 8 (LRRC8A-E), among which LRRC8A is indispensable for channel function. In the recent work, we established that in brain astrocytes LRRC8 isoforms produce several distinct heteromeric populations of VRACs, with different permeability for negatively charged and neutral osmolytes. In order to further explore the physiological roles of VRACs in the brain, we generated brain-wide knock-out of LRRC8A in neurons, astrocytes, and oligodendroglia, using *Nestin* ^Cre^-targeted *Lrrc8a* ^flox/flox^ excision. Mice devoid of brain LRRC8A were born close to the expected Mendelian ratio and developed without overt histological abnormalities. Surprisingly, >95% of the LRRC8A-null mice died between 5 and 9 weeks of age. All LRRC8A knockouts, but none of their littermates, displayed a seizure phenotype confirmed by video-EEG recordings. Immuno-histochemistry and brain slice electrophysiology experiments identified reactive astrogliosis and disruptions in cell excitability and GABAergic signaling in the hippocampus of LRRC8A-null mice. We found reductions in total tissue glutamine levels and decreased immunoreactivity of astrocytic glutamine synthetase, the glutamate transporter GLT-1, and the GABA transporter GAT-1. Together, these findings suggest that VRAC provides vital control of brain excitability in mouse adolescence. VRAC deletion leads to a lethal phenotype involving progressive astrogliosis and hyperexcitability linked to dysregulation of astrocytic uptake and supply of amino acid neurotransmitters and their precursors.

Supported by NIH R01 NS111943

## S-09 Star of the Show: Astrocytes Take Center Stage in Controlling Neural Circuit Formation

S-09-01

**RETINAL INPUTS SIGNAL ASTROCYTES TO RECRUIT INTERNEURONS INTO VISUAL THALAMUS**

Michael Fox

*Fralin Biomedical Research Institute at VTC, Department of Biological Sciences, Roanoke, United States*

Inhibitory interneurons comprise a fraction of the total neurons in visual thalamus but are essential for sharpening receptive field properties and improving contrast-gain of retinogeniculate transmission. During early development, these interneurons undergo long-range migration from germinal zones, a process regulated by the innervation of visual thalamus by retinal ganglion cells. Here, using transcriptomic approaches, we identified a motogenic cue, Fibroblast Growth Factor 15 (FGF15), whose expression in visual thalamus is regulated by retinal input. Targeted deletion of functional FGF15 in mice led to a reduction in thalamic GABAergic interneurons similar to that observed in the absence of retinal input. This loss may be attributed, at least in part, to misrouting of interneurons into non-visual thalamic nuclei. Unexpectedly, expression analysis revealed that FGF15 is generated by thalamic astrocytes and not retino-recipient neurons. Thus, these data show that retinal inputs signal through astrocytes to direct the long-range recruitment of interneurons into visual thalamus.

S-09-02

**ASTROCYTE MODULATION OF CELL-TYPE SPECIFIC SYNAPTIC ORGANIZATION MEDIATED BY SONIC HEDGEHOG SIGNALING**

A. Denise Garcia

*Drexel University, Biology, Philadelphia, USA*

Astrocytes are essential elements in the structural and functional properties of synapses. The molecular signaling mechanisms mediating reciprocal neuron-astrocyte interactions remain poorly understood. In the postnatal and adult mammalian forebrain, the molecular signaling pathway, Sonic hedgehog (Shh) is actively transduced by discrete subpopulations of astrocytes. Shh signaling is initiated by neurons which produce SHH, leading to transcription of SHH target genes, including the transcription factor, Gli1. Though best understood for its prominent roles in embryonic neurodevelopment, the functional significance of SHH-mediated neuron-astrocyte interactions remain poorly defined. To address this question, we performed targeted disruption of Shh signaling selectively in astrocytes and examined structural and functional properties of cortical neurons in postnatal and adult brains. Mutant mice exhibited an elevated density of dendritic spines in cortical neurons. Interestingly, these phenotypes were observed selectively in layer V cortical neurons, where Gli1 astrocytes are abundant, but not in layer II cortical neurons, where few Gli1 astrocytes are found. The increase in spine density emerges during adolescence and persists into adulthood, and arises from prolonged stability of individual spines, as observed by chronic, *in vivo* imaging by 2P LSM. These structural phenotypes are accompanied by aberrant neuronal activity that is associated with a pronounced reduction in expression of Kir4.1, a glial-specific, inward rectifying K^+^ channel. Kir4.1 is essential for maintaining K^+^ homeostasis and limiting neuronal activity, suggesting that astrocyte modulation of neuronal activity is a necessary component of establishing the mature neuronal circuit. Collectively, these observations demonstrate that Shh-mediated reciprocal interactions between neurons and astrocytes during neural circuit development play a key role in establishing and maintaining appropriate synaptic connectivity.

S-09-03

**ASTROCYTE MORPHOGENESIS IS DEPENDENT ON BDNF SIGNALING VIA ASTROCYTIC TRKB.T1**

Michelle Olsen^1^, Leanne Holt^2^, Raymundo Hernandez^1^, Beatriz Torres^1^

*^1^ Virginia Tech, School of Neuroscience, Blacksburg, USA*

*^2^ Cedar-Sinai Medical Center, Icahn School of Medicine, New York, USA*

Brain-derived neurotrophic factor (BDNF) is a critical growth factor involved in the maturation of the CNS, including neuronal morphology and synapse refinement. Herein, we demonstrate astrocytes express high levels of BDNF's receptor, TrkB (in the top 20 of protein-coding transcripts), with nearly exclusive expression of the truncated isoform, TrkB.T1, which peaks in expression during astro-cyte morphological maturation. Using a novel culture paradigm, we show that astrocyte morphological complexity is increased in the presence of BDNF and is dependent upon BDNF/TrkB.T1 signaling. Deletion of TrkB.T1, globally and astrocyte-specifically, in mice revealed morphologically immature astrocytes with significantly reduced volume, as well as dysregulated expression of perisynaptic genes associated with mature astrocyte function. Indicating a role for functional astrocyte maturation via BDNF/TrkB.T1 signaling, TrkB.T1 KO astrocytes do not support normal excitatory synaptogenesis or function. These data suggest a significant role for BDNF/TrkB.T1 signaling in astrocyte morphological maturation, a critical process for CNS development.

S-09-04

**ASTROCYTES, SYNAPSES AND E/I BALANCE**

Iryna Ethell

*University of California School of Medicine, Biomedical Sciences, Riverside, USA*

Astrocytes are implicated in synapse formation, maturation and elimination that are associated with developmental refinements of neuronal circuits and synaptic plasticity that underlie learning and memory. Astrocyte dysfunctions are also linked to synapse pathologies that lead to imbalance between excitatory and inhibitory (E/I) synaptic activity and are associated with several neurologic disorders, including autism spectrum disorders (ASD) and epilepsy. EphB receptor/ephrin-B signaling is among several mechanisms implicated in synaptogenesis and synaptic plasticity. Our recent work suggests its new role in glial-neuronal interactions that may influence both excitatory and inhibitory synapses. We hypothesized that astro-cytic ephrin-B1 may affect synapse formation in the developing hippocampus by influencing trans-synaptic interactions between neu-ronal ephrins and EphB receptors. Using a loss-of-function and a gain-of function approach we showed that astrocytic ephrin-B1 negatively regulates excitatory synapse formation in the hippo-campus during early postnatal day (P) 14-P28 development. A higher number of dendritic spines and enhanced evoked AMPAR and NMDAR excitatory postsynaptic currents (EPSCs) most likely contributed to enhanced excitation of CA1 pyramidal neurons in astro-cyte-specific ephrin-B1 knock-out (KO) mice. In contrast, EPSCs were reduced in CA1 neurons neighboring ephrin-B1 overexpressing (OE) astrocytes. Our studies also implicate astrocytic ephrin-B1 in the development of inhibitory circuits in the hippocampus. Evoked inhibitory postsynaptic currents (IPSC) were reduced in CA1 neurons of P14-28 KO mice, most likely due to reduced number inhibitory synapses on CA1 neurons and impaired maturation of parvalbumin (PV)-expressing inhibitory interneurons. In contrast, astrocyte-specific ephrin-B1 OE increased the number of PV-expressing cells. Astrocyte-specific ephrin-B1 deletion also led to a significant down-regulation of the gene expression associated with astrocyte/microglia reactivity and oligodendrocyte differentiation, but an increased expression of genes associated with excitatory synaptogenesis and neuronal hyperexcitability. The dysregulation of excitatory/inhibitory (E/ I) balance induced by the astrocyte-specific deletion of ephrin-B1 in developing hippocampus was most likely responsible for enhanced susceptibility to seizures, impaired sociability and increased repetitive behaviors observed in these mice. The ability of astrocytic ephrin-B1 influence both excitatory and inhibitory circuits during development can potentially contribute to developmental refinement of neuronal circuits.

## S-10 Novel Non-Narcotic Strategies for Pain Management

S-10-01

**HIGHLY SELECTIVE A3 ADENOSINE RECEPTOR AGONISTS AS NON-NARCOTIC ANALGESICS FOR NEUROPATHIC PAIN**

Daniela Salvemini

*Saint Louis University, School of Medicine, Pharmacology and Physiology, St .Louis, USA*

Chronic neuropathic pain affects 15-20 millions of individuals in the US alone. Neuropathic pain conditions are chronic, often severe, debilitating and exceedingly difficult to treat. Current treatments provide moderate pain relief and have many side effects as exemplified by the use of opioids. There is an urgent need to identify therapeutic targets based on new mechanisms. The purine nucleoside adenosine has long been known to exert potent antinociceptive effects in numerous preclinical models. In human proof-of-principle studies, adenosine has been reported to promote potent analgesia in patients with neuropathic pain. However, the use of adenosine in the treatment of neuropathic pain is limited by its very short half-life and severe cardiovascular side effects due to activation of the A1 and A2A adenosine receptor subtypes (A1AR; A2AAR). Up until recently, the dogma has been that adenosine's effects are mediated by its actions at A1AR, and maybe at the A2AAR. However, targeting the A1AR or A2AAR failed as a therapeutic approach because of the severe cardiovascular side effects resulting from activating these receptor subtypes. Over the last several years, we have discovered that the analgesic effects of adenosine are not only mediated by A1AR or A2AR but also by the A3AR. Our work in this area for the last decade validated the A3AR as a target for therapeutic intervention with A3AR agonists and led to the identification of highly selective A3AR agonists that were used to probe the roles of the ade-nosine-A3AR axis in pain. I will describe the pharmacological profile of selective A3AR agonists in preclinical models of neuropathic pain in rodents and discuss molecular and biochemical signaling pathways engaged downstream of A3AR activation. Noteworthy, highly selective A3AR agonists are now in clinical development as novel non-narcotic analgesics.

S-10-02

**UNLOCKING NAV1.7'S PAIN POTENTIAL**

Rajesh Khanna

*University of Arizona, Pharmacology, Tucson, USA*

NaV1.7 is a key ion channel in pain signaling. Gain-of-function mutations in the human NaV1.7 gene produce sensory neurons hyperexcitability associated with severe pain; whereas loss-of-function mutations generate congenital insensibility to pain syndromes. However, efforts to develop NaV1.7 inhibitors for pain therapeutics have consistently failed. Post-translational modifications of NaVs and/or auxiliary subunits and protein-protein interactions have been reported as NaV-trafficking mechanisms. We recently reported that modification of the axonal collapsin response mediator protein 2 (CRMP2) by a small ubiquitin-like modifier (SUMO) controls both trafficking and currents of NaV1.7 (Dustrude *et al.*, *J. Biol. Chem.* 288: 24316-31 (2013)). Capitalizing on this unique pathway for NaV1.7 regulation, Regulonix Holding Inc. identified compounds by computationally docking 50,000 small molecules to a pocket encompassing the residue SUMOylated (K374) in CRMP2. These compounds were designed to inhibit the E2-conjugating enzyme Ubc9-CRMP2 interaction, which, in turn, would block CRMP2 from being SUMOylated by Ubc9. Among the over 266 compounds identified in this manner, several (*i*) exhibited superb inhibition of the Ubc9-CRMP2 interaction, (*ii*) bound to CRMP2 - but not Ubc9, and notably, (*iii*) did not affect any other CRMP2-mediated functions, including facilitation of neurite outgrowth, ability to bind to protein partners (tubulin, N-type voltage-gated calcium (CaV2.2), and N-methyl-D-aspartate receptor (NMDAR)), and ability to regulate CaV2.2 activity. Superb anti-allodynic activities without loss of motoric performance or sympathetic side effects were observed for several compounds. Furthermore, animal pharmacological studies indicated that some of the compounds displayed extended duration of action (2–16-fold) compared with morphine upon intrathecal administration to rats. Additional studies demonstrated inhibition of NaV1.7 currents in human and porcine sensory neurons, thus increasing likelihood of translational success and ‘de-risking’ compound selection. Thus, we advance an innovative approach by focusing on a unique mechanism of action of the compounds that involves an indirect targeting to control surface expression and activity of the NaV1.7 channel.

S-10-03

**DISRUPTION OF PROTEIN-PROTEIN INTERACTIONS AS A NON-NARCOTIC ANALGESIC STRATEGY**

Andrea Hohmann^1,2,3^, Wan-Hung Lee^2^, Lawrence Carey^1,2,3^, Shahin Saberi^1^, Claudia Rangel-Barajas^1^, Zhili Xu^1,3^, Vishakh Iyer^1,3^, Kendra Bunner^1,3^, Jonathon Crystal^1,3^, George Rebec^1,3^, Yvonne Lai^1^

*^1^ Indiana University, Psychological and Brain Sciences, Bloomington, USA*

*^2^ Indiana University, Interdisciplinary Biochemistry Program, Bloomington, USA*

*^3^ Indiana University, Program in Neuroscience, Bloomington, USA*

Aberrant increases in NMDA receptor (NMDAR) signaling contributes to central nervous system sensitization and chronic pain by activating the enzyme neuronal nitric oxide synthase (nNOS) and generating the signaling molecule nitric oxide (NO). The scaffolding protein postsynaptic density 95kDA (PSD95) tethers nNOS to NMDARs. Consequently, the PSD95-nNOS complex represents a therapeutic target. We hypothesized that disruption of the protein-protein interface between PSD95 and nNOS would suppress NMDAR-dependent pathological states without unwanted side-effects of NMDAR antagonists. The small molecule PSD95-nNOS inhibitor IC87201 and a related analog, ZL006, inhibited binding of purified PSD95 and nNOS proteins in AlphaScreen without altering binding of PSD95 to an unrelated protein, ErbB4. Both PSD95-nNOS inhibitors also suppressed glutamate-induced cell death with efficacy comparable to the NMDAR antagonist MK-801. IC87201 and ZL006 produced antinociception in multiple models of inflammatory pain and also suppressed formalin-evoked Fos protein expression in rat lumbar spinal cord. IC87201 and ZL006 also suppressed allodynia induced by the chemotherapeutic agent paclitaxel with efficacy similar to MK-801. MK-801 also impaired both memory and motor functions, which were not inhibited by ZL006 and IC87201 under similar conditions. Neither IC87201 nor ZL006 produced reward or aversion in a conditioned place preference (CPP) assay. However, both ligands blocked CPP to morphine. The PSD95-nNOS inhibitor also suppresses morphine-induced dopamine efflux in the nucleus accumbens shell. Collectively, our results demonstrate that disrupting PSD95-nNOS protein-protein interactions is effective in attenuating pathological pain without producing unwanted side effects (i.e. motor ataxia, reward) associated with NMDAR antagonists. Supported by CA200417, DA042584, and DA037673.

S-10-04

**BLOCKING S1PR1 PREVENTS OPIOID-INDUCED ADVERSE EFFECTS**

Timothy Doyle^1,2^, Daniela Salvemini^1,2^

*^1^ Saint Louis University, Henry and Amelia Nasrallah Center for Neuroscience, St .Louis, USA*

*^2^ Saint Louis University, Pharmacology and Physiology, St .Louis, USA*

Opioid-induced alterations in sphingolipid metabolism in the spinal cord and increased formation of the bioactive sphingolipid metabolite sphingosine-1-phosphate (S1P) are implicated in the development of opioid-induced hyperalgesia (OIH; increased pain sensitivity) and analgesic tolerance. These opioid-induced adverse effects hamper opioid use for treating chronic pain and can contribute to dependence and abuse. S1P produces distinct effects through five G protein-coupled receptors (S1PR1-5) and several intracellular targets. We now report that S1P contributes to the development of OIH, tolerance and dependence through S1P1 receptor subtype 1 (S1PR1) signaling and blocking S1PR1 with S1PR1 antagonists attenuated these opioid-induced adverse behaviors. Targeting S1PR1 reduced opioid-induced neuroinflammation that included reducing glial activity, nuclear factor κB and mitogen-activated protein kinase p38 signaling activation and inflammatory cytokine expression, such as interleukin-1β, while increasing IL-10 expression. Our findings identify S1PR1 as a critical path for S1P signaling in response to sustained morphine and reveal downstream neuroinflammatory pathways impacted by S1PR1 activation. Our work supports investigating S1PR1 antagonists as a clinical approach to mitigate opioid-induced adverse effects and the repurposing of the functional S1PR1 antagonist FTY720, which is already FDA-approved for multiple sclerosis, as an opioid adjunct.

This work was supported by grants from the National Institute of Drug Abuse (RO1DA043543; Drs. Salvemini).

## S-11 Mechanisms of White Matter Injury and Repair After Traumatic Brain Injuries

S-11-01

**AXON-MYELIN PROTECTION AND PLASTICITY IN TRAUMATIC WHITE MATTER INJURY**

Regina Armstrong

*USUHS, Anatomy, Physiology & Genetics, Bethesda, USA*

Long axons in white matter tracts are particularly vulnerable to the forces from traumatic brain injury (TBI). The subsequent trajectory of recovery versus progression of white matter pathology is a key feature underlying neurodegeneration among patients who experience persistent symptoms after TBI. Importantly, the dynamic interactions between damaged axons and ensheathing myelin open opportunities for novel therapeutic interventions that are being explored in pre-clinical models. Using a mouse model of concussive TBI, our studies identify a progression of both myelinated axon conduction deficits and axon-myelin pathology in the corpus callosum, implicating white matter injury in impaired information processing, which is common in TBI patients. Partial recovery during the subacute phase reveals a potential therapeutic window associated with remyelination. However, with longer time post-TBI, atrophy of the corpus callosum indicates progression to chronic stage pathology. To test mechanisms to mitigate white matter injury after TBI, we examined the role of SARM1 protein that is essential for execution of the conserved axon death pathway. Neuropathological analysis of mice with *Sarm1* gene deletion (*Sarm1*-/-) versus wild type (*Sarm1*+/+) littermates showed that loss of *Sarm1* reduces axon damage, demyelination and white matter atrophy after TBI. Longitudinal MRI studies of live mice detected significant corpus callosum atrophy after TBI in *Sarm1*+/+ mice that was attenuated in *Sarm1*-/-mice. Furthermore, at this chronic phase post-injury, *Sarm1*+/+ mice exhibited functional deficits in Miss-step wheel running and sleep behavior that were not present in *Sarm1*-/-mice. These studies underscore the role of SARM1 in white matter injury after TBI, and provide evidence for TBI as a potential clinical indication for therapeutics targeting this axon degeneration pathway.

These studies were funded by the U.S. Department of Defense and the Uniformed Services University through the UCSF-USUHS Partnership: Brain Injury and Disease Prevention, Treatment, and Research and the Center for Neuroscience and Regenerative Medicine. The authors declare no competing financial interests. Opinions are those of the authors and do not represent the University, the Department of Defense, or the federal government.

S-11-02

**REPAIR AND RESTORATION OF THE CEREBROVASCULATURE FOLLOWING TRAUMATIC BRAIN INJURY: THERAPEUTIC IMPLICATIONS**

Andre Obenaus

*University of California Irvine, Pediatrics, Irvine, USA*

Cerebral vascular injury is a defining feature of traumatic brain injuries (TBI) which leads to secondary injury and influences clinical outcomes. There is an urgent need to develop new therapies to repair and restore cerebral vessels. We previously reported that TBI leads to an immediate and early loss of vessels at the injury site and vascular abnormalities adjacent to the TBI. Without therapeutic intervention we observed that vessels attempt to repair and restore vascular function to the injured brain. Interestingly, we reported that increased b-catenin and Wnt expression in cerebral vessels which coincides with vascular repair after brain injury. Wnt/b-catenin signaling promotes blood vessel formation during embryonic development, but its role in vascular repair after TBI remains unclear. Ongoing studies demonstrate that we can enhance or retard vascular repair and thereby modulating vascular function using a range of pharmacological and small molecule compounds. In mice the restored vasculature of showed increased branching and elongated vessels that coincided with a reduction in hemorrhage. Our findings suggest that Wnt/b-catenin signaling becomes activated after TBI to promote vascular repair thus providing a potential target for future therapeutic intervention.

S-11-03

**TBI-INDUCED INTRACELLULAR SIGNALING AND MYELIN DISRUPTION IN**

**OLIGODENDROCYTES**

Haesun Kim

*Rutgers University, Biological Sciences, Newark, USA*

Myelin loss in brain is a common occurrence in traumatic brain injury (TBI) that results from impact-induced acceleration forces to the head. Fast and abrupt head motions, either resulting from violent blows and/or jolts, cause rapid stretching of the brain tissue, and the long axons within the white matter tracts are especially vulnerable to such mechanical strain. Recent studies have shown that mechanotransduction plays an important role in regulating oligo-dendrocyte progenitors cell differentiation into oligodendrocytes. However, little is known about the impact of mechanical strain on mature oligodendrocytes and the stability of their associated myelin sheaths. We used an *in vitro* cellular stretch device to address these questions, as well as characterize a mechanotransduction mechanism that mediates oligodendrocytes’ responses. Mechanical stretch caused myelin protein loss in oligodendrocytes. Cell death was not observed. Myelin protein loss was accompanied by an increase in intracellular Ca^2+^ and Erk1/2 activation. Chelating Ca^2+^ or inhibiting Erk1/2 activation was sufficient to block the stretch-induced loss of myelin protein. Further biochemical analyses revealed that the stretch-induced myelin protein loss was mediated by the release of Ca^2+^ from the endoplasmic reticulum (ER) and subsequent Ca^2+^-dependent activation of Erk1/2. Altogether, our findings characterize an Erk1/2-dependent mechanotransduction mechanism in mature oligo-dendrocytes that de-stabilizes the myelination program.

S-11-04

**LIF COUNTERPOSES TRAUMATIC WHITE MATTER INJURY AND PRESERVES SENSORIMOTOR FUNCTION**

Steven Levison

*Rutgers University, Pharmacology, Physiology & Neuroscience, Newark, USA*

Cytokines and growth factors are key candidates for mediating the changes induced by damage to the brain as they can affect astrocyte proliferation, microglial activation and cell survival. Leukemia inhibitory factor (LIF), a member of the interleukin-6-type cytokine family, is rapidly induced after CNS injury and participates in all of these processes. We have compared the extent of damage to subcortical white matter in LIF heterozygous (LIF-H) and wild type (WT) mice using a model of mild traumatic brain injury. In WT juvenile mice LIF transcripts increased ∼15 fold 24 hours following a closed head injury accompanied by both astroglial and microglial activation. LIF-H mice had decreased astrocyte activation and a blunted microglial response. Over time, the WT mice recovered whereas damage increased and subsequently neurological function diminished in the LIF-H mice. As LIF deficiency was detrimental to recovery, we asked whether elevating brain levels of LIF would be beneficial. Therefore, we administered recombinant LIF to wild type mice intranasally either at 4 hours or at 3 days after injury. LIF was administered twice per day for 3 days. Intranasal LIF decreased astroglial activation, reduced axonal damage, preserved numbers of oligodendrocytes and corpus callosum white matter and improved sensorimotor function compared to vehicle treated injured mice. These data support the view that LIF functions as an important trophic cytokine in the CNS and that sustaining levels of LIF during the subacute phase after injury will improve neurological function. Supported by grant # CBIR13IRG017 and CBIR19FEL014 from the NJ Commission on Brain Injury Research.

## S-12 Therapeutic Potential of Psychedelics

S-12-01

**NEURAL MECHANISMS UNDERLYING THE PROSOCIAL EFFECTS OF MDMA**

Robert Malenka

*Stanford, Psychiatry, Stanford, USA*

Positive prosocial interactions contribute to the development and maintenance of a range of adaptive, cooperative behaviors. Conversely, inability to participate in normal social interactions is a debilitating symptom of several prominent neuropsychiatric disorders. Although the role of neuromodulators in social behaviors is an active area of investigation, relatively little is known about the detailed neural mechanisms that influence sociability. This talk will review evidence that release of serotonin in the nucleus accumbens plays a critical role in promoting sociability. Deficits in the action of serotonin in the nucleus accumbens may contribute to sociability deficits in mouse models of autism spectrum disorder (ASD). Consistent with this hypothesis, administration of MDMA enhances sociability in both wildtype and ASD mice due to its serotonin-releasing properties in the nucleus accumbens.

S-12-02

**UNTANGLING KETAMINE'S ANTIDEPRESSANT CIRCUITRY THROUGH PARALLEL HUMAN AND MOUSE EXPERIMENTS**

Boris Heifets

*Stanford University School of Medicine, Department of Anesthesiology, Perioperative & Pain Medicine, Stanford, USA*

Ketamine (KET), one of our oldest anesthetic agents, has found new life as a rapid-acting antidepressant. Its profound psychoactive effects and use as a psychotherapeutic adjunct have drawn comparisons with psychedelic-assisted psychotherapy, another promising strategy for the rapid treatment of depression. While KET appears to have significant therapeutic potential, scaling its use for millions of patients is limited by its well-known abuse potential, short duration of effect, and toxicity associated with chronic high dose use. Developing safer, better KET-like drugs requires a clear understanding of KET's mechanism. Despite early enthusiasm for N-Methyl D-Aspartate receptor (NMDAR) antagonism as KET's primary antidepressant mechanism, multiple failed clinical trials of other NMDAR antagonists suggest this account is incomplete. Recent work has broadened possible explanations for KET's anti-depressant mechanism to include a multiplicity of low-affinity molecular targets, challenging the usefulness of a traditional molecular pharmacology approach.

In this presentation, I will show parallel mouse and human experiments that describe the distributed ensemble of neural activity that may define KET's antidepressant effect. We recently found that KET's antidepressant effect is completely blocked by pretreating depressed patients with naltrexone, an opioid receptor antagonist. Using this key insight, we have mapped opioid-receptor dependent KET effects across the entire rodent brain. We have combined whole brain clearing methods and light sheet imaging with a genetically modified mouse line in which a fluorescent reporter gene is selectively transcribed only in neurons that are active during a drug experience. Comparing brain-wide unbiased “ensemble” maps generated by KET versus KET+naltrexone, we have identified several cortical and subcortical areas that may be sufficient to drive ketamine's antidepressant response. I will also describe our work testing the role of conscious subjective awareness during KET infusion for the induction of KET's antidepressant effect. Our preliminary data from depressed patients presenting for noncardiac surgery indicates that KET delivered during general anesthesia maintains its antidepressant efficacy, and acute KET-associated EEG responses may predict the clinical response.

S-12-03

**IMAGING THE RAPID AND LONG-LASTING ACTIONS OF PSYCHOACTIVE DRUGS: FROM KETAMINE TO PSILOCYBIN**

Alex Kwan

*Yale University, Psychiatry, New Haven, USA*

Pilot studies have hinted that serotonergic psychedelics such as psilocybin may relieve depression, and could possibly do so by promoting neural plasticity. Intriguingly, another psychotomimetic compound, ketamine, is a fast-acting antidepressant and induces synapse formation. The similarities in behavioral and neural effects have been puzzling because the compounds target distinct molecular receptors in the brain. In this talk, I will discuss recent work on determining the actions of ketamine and psilocybin on cortical neurons in mice. In particular, I will describe a series of studies using subcellular-resolution optical microscopy to dissect the impact of these compounds on dendritic structure and dendritic calcium signaling. Based on these results, I will summarize by proposing a framework based on dendritic excitability that may underlie ketamine and psilocybin's capacities to promote neural plasticity.

S-12-04

**PSYCHEDELICS AND RELATED PLASTICITY-PROMOTING NEUROTHERAPEUTICS**

David Olson

*University of California, Davis, Chemistry; Biochemistry & Molecular Medicine, Davis, USA*

Neural plasticity—broadly defined as the ability of the brain to change and adapt—is of fundamental importance to a properly functioning nervous system. It is the basis for learning and memory and enables our brains to recover from the pathological changes that underlie neuropsychiatric diseases such as depression, post-traumatic stress disorder, and substance use disorder. Recently, our group discovered that psychedelic natural products and related compounds, such as LSD, DMT, and ibogaine, rapidly promote structural and functional neural plasticity in rodents. These compounds are capable of re-wiring neural circuitry to produce long-lasting antidepressant, anxiolytic, and anti-addictive behavioral responses. Psychedelics have inspired our total synthesis and medicinal chemistry efforts to develop safer and more effective neurotherapeutics, and they serve as key chemical tools in our studies to understand the fundamental bio-chemical mechanisms that give rise to neural plasticity.

## S-13 Extracellular Vesicles in Alzheimer's Disease

S-13-01

**DYNAMICS BETWEEN EXTRACELLULAR VESICLES AND AMYLOID-Β PROTEIN**

Michael Nichols

*University of Missouri - St. Louis, Chemistry & Biochemistry, St. Louis, USA*

Extracellular vesicles (EVs) are class of cell-secreted particles that include microvesicle (MV) and exosome subgroups. These cargo-holding vesicles mediate cell-to-cell communication and appear to be linked in some way with neurodegenerative diseases such as Alz-heimer's disease (AD). Both the smaller exosomes (10-100 nm diameter) and the larger MVs (100 nm-1 µm) have been shown to carry amyloid-β (Aβ) and tau proteins as part of their cargo, and their levels are elevated in AD. However, less is known about the specific mechanisms of EVs in AD. The rational for the studies described herein comes from longtime observations of microglial cell clustering around neuritic Aβ plaques in AD brains. Due to their close proximity, it is reasonable to imagine that there may be processes linking microglia, EVs and Aβ.

Multiple strategies were used to characterize MVs released from microglia after ATP stimulation. Confocal microscopy, dynamic light scattering, and transmission electron microscopy revealed a polydis-perse population of small spherical structures ranging in size (diameter) from 150-600 nm but centering around 300-350 nm. Using a fluorescently-labeled membrane insertion probe, MVs were tracked in several different assays. This method, along with an Aβ ELISA, allowed demonstration of a strong interaction between MVs and oligomeric/protofibrillar Aβ. MVs had much less interaction with monomeric Aβ, yet displayed an inhibitory effect on Aβ monomer aggregation. An additional pathway of EV/Aβ interaction was characterized that involved trafficking of Aβ into MVs by microglia. Primary microglia rapidly internalized Aβ protofibrils, compartmentalized the protein aggregates into MVs, and released these Aβ-containing MVs after stimulation of the microglia with ATP. Further EV/Aβ interactions remain to be investigated, including differences between EV subtypes and a better understanding of the role of EVs in neurodegenerative and inflammatory processes.

S-13-02

**PLASMA EXTRACELLULAR VESICLES ENRICHED FOR NEURONAL AND ASTROCYTIC ORIGIN IN ALZHEIMER'S DISEASE**

Dimitrios Kapogiannis^1^

*^1^ National Institute on Aging (NIA/NIH), Laboratory of Clinical Investigation, Baltimore, USA*

*^2^ Johns Hopkins University School of Medicine, Neurology, Baltimore, USA*

**Background:** Neuronal and astrocytic-derived Extracellular Vesicles (NEVs and AEVs) can be isolated from blood with immuno-precipitation against neuronal/glial proteins. NEV cargo provides diverse Alzheimer's disease (AD) biomarkers, including Abeta42, total and phospho-Tau, insulin signaling mediators, synaptic proteins, cellular stress response factor REST, lysosomal enzymes, and mtRNAs. In a large case control study based on Baltimore Longitudinal Study of Aging participants, NEV biomarkers predicted future AD diagnosis with ∼90% area-under-curve (AUC), and>80% sensitivity and specificity. AEV AD biomarkers include potentially neurotoxic complement proteins. **Methods:** We follow a two-step methodology, isolating total EVs from plasma through particle precipitation, then, NEVs or AEVs through immunoprecipitation against neuronal L1CAM or astrocytic GLAST. A second validation study of NEV biomarkers for AD prediction was based on 292 serum samples from 146 Wisconsin Registry for Alzheimer's prevention (WRAP) participants, divided into longitudinal cognitive decliners and stable participants. To study functional effects of circulating EVs, we co-cultured AEVs and NEVs with rat cortical and human iPSC-derived neurons and performed cell viability and neurodegeneration assays. **Results:** Biomarkers: Decliners had higher levels of p231Tau and t-Tau, and lower ratios of p231-TaU/t-Tau and p181Tau/t-Tau (all p<0.01) compared to stable individuals; a model combining longitudinal NEV biomarkers achieved ∼94 % AUC, with high sensitivity and specificity for predicting cognitive decline. Functional assays: AEVs (and, less effectively, NEVs) of AD participants induced Membrane Attack Complex (MAC) expression on recipient neurons, membrane disruption, neurodegeneration with reduced neurite density, and decreased cell viability. Neurodegenerative effects were not produced by total EVs from the same AD participants or AEVs/NEVs from FTLD or control participants, and were suppressed by the MAC inhibitor CD59. **Conclusions:** NEV biomarkers can predict cognitive decline in late middle age and, perhaps, diagnose preclinical AD. Circulating AEV/NEV can induce neurodegeneration, which depends on MAC deposition. Inhibition of EV-associated complement components may be therapeutically beneficial in AD.

S-13-03

**THE ROLE OF THE CERAMIDE TRANSFER PROTEIN (CERTL) IN NEURO-INFLAMMATION IN ALZHEIMER'S DISEASE**

Pilar Martinez-Martinez

*Maastricht University, Neuroscience, Maastricht, Netherlands*

Ceramide and sphingomyelin (SM) are sphingolipids, important for cell membrane structure and cell biology. In the brain tissue of Alz-heimer's disease (AD) patients, levels of certain ceramide species are elevated, whilst other sphingolipids like SM are decreased. Ceramide transfer proteins (CERTs) are the only known ceramide carriers, crucial for ceramide and SM regulation. Blockage of CERTs ceramide transfer activity leads to reduced levels of SM *in vitro*. Moreover, besides their ceramide transfer function, CERT proteins are also able to activate the complement system and co-localize with amyloid-β (Aβ) plaques in the AD brain. To date, the significance of these observations to the pathophysiology of AD remained uncertain. We will illustrate novel mechanism of action of CERT, and highlight its role in abeta homeostasis, neuro-inflammation and lipid levels in the brain. Based on these novel observations new research pathways will be revealed for identifying therapeutic targets of AD and other neurodegenerative diseases.

S-13-04

**ABETA-ASSOCIATED EXOSOMES FROM ASTROCYTES DRIVE AMYLOID PATHOLOGY IN ALZHEIMER'S DISEASE**

Erhard Bieberich

*University of Kentucky, Department of Physiology, Kentucky, USA*

Alzheimer's disease (AD) is characterized by build-up of amyloid β peptide (Aβ) and phosphorylated tau. However, amyloid plaque and tau tangle formation are often not directly correlated with neuro-toxicity and cognitive decline. We proposed an alternative mechanism implying an unknown but critical factor that mediates neuro-toxicity of amyloid and tau. We first focused on amyloid toxicity and discovered that Aβ associates with astrocyte-derived and ceramide-enriched lipid vesicles (exosomes) we termed astrosomes. Aβ-associated astrosomes nucleate amyloid plaques in the 5xFAD mouse, a model for familial AD. We also showed that pharmacological inhibition of ceramide generation reduced plaque formation and improved cognition, indicating that Aβ association to astrosomes is a critical factor in AD pathology. Our current research shows that association of Aβ to astrosomes is not only critical for amyloid plaque nucleation, but also for Aβ neurotoxicity. We found that Aβ-associated astrosomes induce mitochondrial damage and caspase 3 activation in neurons at an Aβ concentration several orders of magnitude lower than what was previously reported to be neurotoxic. Currently, we investigate the molecular mechanism underlying mito-toxicity of Aβ-associated astrosomes and therapeutic strategies targeting ceramide to protect neurons in AD. This work is supported by grants NIH R01AG034389 and R01NS095215, and VA 1 I01 BX003643.

## S-14 Unconventional Roles for Astrocytes in Disease

S-14-01

**DISEASE-RELEVANT DIFFERENCES BETWEEN HUMAN AND MOUSE ASTROCYTES**

Ye Zhang, Jiwen Li, Marlesa Godoy, Linsey Stiles, Angela Chen, Ina Wanner

*UCLA, Psychiatry, Los Angeles, USA*

Astrocytes are critical for the development, function, and homeo-stasis of the central nervous system. In a range of diseases and injuries, astrocytes undergo reactive with both beneficial and detrimental effects depending on the disease context. However, most of our knowledge of reactive astrocytes has been obtained from mouse studies. Our understanding of astrocyte biology in humans remains in its infancy. Previous methods to purify primary human astrocytes requires culturing in serum, blood components that induce reactive astrogliosis in injury and disease. We recently developed an immunopanning method to purify human astrocytes and a serum-free culturing condition that maintains primary human astrocytes at resting state.

Here, we used this method to purify primary human and mouse astro-cytes and compared their responses to four types of disease-relevant perturbations: oxidative stress, inflammatory cytokine TNFa treatment, hypoxia, and poly I:C treatment which mimics viral infection. We found that human astrocytes are more susceptible to oxidative stress compared to mouse astrocytes. To determine the cellular and molecular mechanisms underlying the differences in oxidative stress susceptibility between human and mouse astrocytes, we performed mitochondria respiration assays. We found that mouse astrocytes exhibit higher rate of mitochondria respiration than human astro-cytes, thus producing more reactive oxygen species (ROS). We found that mouse astrocytes developed adaptive advantages in ROS detoxification pathways compared to human astrocytes, including (1) higher rate of oxidation at peroxisomes, an organelle involved in ROS detoxification, (2) higher expression of catalase, an enzyme that breaks down hydrogen peroxide, and (3) higher expression of glucose-6-phosphate dehydrogenase, an enzyme of the pentose phosphate pathway, which generates NADPH that neutralizes ROS. We also found that TNFa, hypoxia, and poly I:C treatments induced different molecular responses in human vs mouse astrocytes. For example, TNFa induced cellular senescence pathway in human but not mouse astrocytes. These differences may contribute to differences between mouse models and human patients with neuro-logical disorders.

S-14-02

**PROTECTIVE ROLES FOR ASTROCYTIC CELL DEATH SIGNALING DURING CNS VIRAL INFECTION**

Brian Daniels, Marissa Lindman, Kimberly Newman, Colm Atkins

*Rutgers University, Cell Biology and Neuroscience, Piscataway, USA*

Programmed cell death (PCD) is a key feature of the innate immune response to viral infection, as it both limits replicative niches for viruses and instructs subsequent antiviral immunity. How-ever, programmed cell death is also a hallmark of immunopathology and may represent a maladaptive strategy for pathogen control in nonregenerative tissues such as the central nervous system (CNS). Necroptosis is an immunogenic form of PCD that is induced via activation of receptor interacting protein kinase-3 (RIPK3). Engagement of RIPK3 activity in neurons can induce inflammatory gene transcription in the absence of cell death, demonstrating specialized adaptation of this pathway in neural cells. However, potential specialized responses for this pathway in glial cells have not yet been described. Here, we demonstrate that engagement of the necroptotic kinase RIPK3 during Zika virus infection drives neuroin-flammatory astrocyte activation and restricts viral replication without inducing necroptotic cell death. Using mouse models of viral encephalitis, we show that astrocytic RIPK3 signaling is required to limit CNS viral burden, promote protective neuroinflammation, and ensure host survival. These effects were associated with the RIPK3-dependent induction of a neuroinflammatory astrocytic trans-criptional program. These data identify previously unknown, death-independent functions for RIPK3 in astrocytes, key cellular mediators of neuroimmunological responses to infection.

S-14-03

**THE ASTROCYTE IMMUNOPROTEASOME MEDIATES PROTECTION DURING CHRONIC CNS AUTOIMMUNITY**

Jessica Williams

*Cleveland Clinic, Neurosciences, Cleveland, USA*

Multiple sclerosis (MS) is an inflammatory and neurodegenerative disease of the CNS for which there is a critical need for therapeutic intervention, specifically in patients with chronic, progressive disease. During MS pathogenesis, multiple inflammatory mediators including reactive oxygen species (ROS), which cause oxidative stress and damage intracellular proteins, and interferon (IFN)g, which is known to enhance inflammation in early disease stages, are present. Astrocytes in and around MS lesions are critical for the maintenance of homeostasis and are responsive to many inflammatory mediators during neuroinflammation, including IFNg. Paradoxically, IFNg also has protective functions during chronic stages of CNS autoimmunity. Analyzing astrocytes in postmortem human tissue specimens from patients with chronic MS, we found that an IFNg-stimulated protein complex, the immunoproteasome (iP), is upregulated in specific CNS regions and that iP expression corresponded with a reduction in oxidative stress. We confirmed that iP expression was stimulated by IFNg in regional primary adult human astrocytes and that specific inhibition of the iP using ONX 0914 following IFNg stimulation resulted in increased ROS, an accumulation of oxidatively damaged proteins, and enhanced caspase-3- mediated cell death. In a murine model of chronic MS, experimental autoimmune encephalomyelitis (EAE), ONX 0914 treatment led to exacerbated disease and increased lesion size, astrocyte oxidative stress and poly-ubiquitinated protein accumulation. Similarly, mice with a conditional deletion of IFNgR on astrocytes had a reduction in astrocyte iP expression and exhibited exacerbated EAE with increased lesion size, and evidence of enhanced oxidative stress and poly-ubiquitinated protein accumulation in astrocytes compared to littermate controls. Taken together, our data suggest for the first time that IFNg signaling in astrocytes upregulates the iP with regional specificity and has a novel role in contributing to CNS homeostasis by reducing ROS and degrading damaged poly-ubiquitinated proteins during chronic neuroinflammation. Understanding the role of the astrocyte iP and further dissecting the protective functions of IFNg during chronic neuroinflammation may lead to development of novel targets for treatment of progressive MS.

S-14-04

**ASTROCYTIC MITOCHONDRIAL TRANSFER REGULATES NEURONAL SURVIVAL AND PLASTICITY AFTER STROKE**

Kazuhide Hayakawa

*Massachusetts General Hospital, Radiology, Charlestown, USA*

Recently, it was suggested that neurons can release and transfer damaged mitochondria to astrocytes for disposal and recycling. This ability of brain cells to exchange mitochondria may represent a new paradigm for cell-cell signaling in the central nervous system (CNS). Here, we show that astrocytes can also release functional mito-chondria that enter into neurons. Astrocytic release of extracellular mitochondria was mediated by a calcium-dependent mechanism involving CD38/cyclic ADP ribose signaling. Transient focal cerebral ischemia in mice induced astrocytic mitochondria entry to adjacent neurons that amplified cell survival signals. Suppression of CD38 signaling with siRNA reduced extracellular mitochondria transfer and worsened neurological outcomes. These findings suggest a new mitochondrial mechanism of neuroglial crosstalk that may underlie endogenous neuroprotective mechanisms after stroke.

## S-15 Small Molecule Approaches to Understanding and Treating Neurological Disease

S-15-01

**ACCUMULATION OF 8,9-UNSATURATED STEROLS AS A UNIFYING MECHANISM FOR DRUG-INDUCED REMYELINATION**

Drew Adams^1^, Zita Hubler^1^, Dharmaraja Allimuthu^1^, Ilya Bederman^1^, Matthew Elitt^1^, Mayur Madhavan^1^, Kevin Allan^1^, Elizabeth Schick^1^, Eric Garrison^2^, Molly Karl^2^, Daniel Factor^1^, Zachary Nevin^1^, Joel Sax^1^, Matthew Thompson^1^, Yuriy Fedorov^1^, Jing Jin^3^, William Wilson^3^, Martin Giera^4^, Franz Bracher^5^, Robert Miller^2^, Paul Tesar^1^

*^1^ Case Western Reserve University School of Medicine, Genetics and Genome Sciences, Cleveand, USA*

*^2^ George Washington University, Anatomy and Regenerative Biology, Washington, DC, USA*

*^3^ Rice University, Department of Biosciences, Houston, USA*

*^4^ Leiden University, Medical Center, Leiden, Netherlands*

*^5^ Ludwig-Maximilians University, Department of Pharmacy, Munich, Germany*

Remyelination represents an unmet need in multiple sclerosis therapy. High-throughput screens conducted in recent years have validated a diverse range of FDA-approved drugs and small mole-cules that promote oligodendrocyte formation from oligodendrocyte progenitor cells. Recently we reported that dozens of small molecules that enhance oligodendrocyte formation share the ability to inhibit a narrow range of steps in cholesterol biosynthesis. By inhibiting enzymes between CYP51 and EBP, these molecules cause the cellular accumulation of specific 8,9-unsaturated sterols, which are sufficient to promote oligodendrocyte formation when supplied in purified form. These studies suggest novel druggable targets and lead molecules to accelerate the development of the first remyelinating therapeutics.

S-15-02

**CHEMISTRY-BASED METHODS FOR IDENTIFYING BINDING SITES FOR STEROID MODULATORS OF ION CHANNELS**

Douglas Covey

*Washington Univ. in St. Louis, Dept. Developmental Biology/School of Medicine, St. Louis, USA*

The endogenous neurosteroid allopregnanolone (AlloP) is a positive allosteric modulator (PAM) of γ-aminobutyric acid type-A (GABA-A) receptors. AlloP and analogues of this neurosteroid are of therapeutic interest for the treatment of clinical depression and other medical conditions. Identification of amino acids at putative binding sites for AlloP on GABA-A receptors and other ion channels has been achieved using molecular biology methods. However, much remains to be learned about the molecular details of neurosteroid interactions with the sites thus far identified. In this regard, photoaffinity labeling of these sites by neurosteroid analogues provides information about the location, orientation and number of binding sites for neurosteroids on these receptors. Such information is useful for the design of new drugs that act at these sites. Photoaffinity labeling when combined with click chemistry (click photolabels) provides an enhanced ability to obtain this information using mass spectrometry and molecular modeling. Additionally, click photolabels also are useful for identifying binding sites that may not yet have been detected by site-directed mutagenesis studies. This presentation will discuss results obtained with neurosteroid click photolabels for GABA-A receptors. This work was supported by NIH grants GM108799 and The Taylor Institute for Innovative Psychiatric Research.

S-15-03

**ALLOSTERIC REGULATION OF PROTEIN QUALITY CONTROL E3 LIGASE**

Matt Scaglione^1^, Adam Kanack^2^, Michael Olp^2^, Jamie Scaglione^1^, Brian Smith^2^

*^1^ Duke University, Molecular Genetics and Microbiology, Durham, USA*

*^2^ Medical College of Wisconsin, Biochemistry, Milwaukee, USA*

The accumulation of protein aggregates is a hallmark of most neurodegenerative diseases. To counteract protein aggregation cells utilize an array of pathways including molecular chaperones that refold misfolded proteins, and ubiquitination enzymes that target misfolded proteins for degradation. One E3 ligase, C-terminus of Hsc70 Interacting Protein (CHIP), sits at the interface of protein folding and protein degradation. CHIP functions by binding molecular chaperones and E2 ubiquitin conjugating enzymes to facilitate the ubiquitination and subsequent degradation of chaperone client proteins. The clearance of these chaperone bound client proteins prevents the toxic accumulation of misfolded proteins. The removal of these toxic proteins by CHIP is neuroprotective. Consistent with this, increasing CHIP levels is neuroprotective in models of neuro-degenerative diseases, and decreasing levels of CHIP accelerates phenotypes associated with neurodegeneration. Based on these observations we hypothesized that small molecules that increase CHIP activity may be protective. Here we will discuss our efforts to develop small molecules that regulate CHIP activity.

S-15-04

**MULTIPLE SCLEROSIS RISK GENE MERTK IS REQUIRED FOR MICROGLIAL ACTIVATION AND SUBSEQUENT REMYELINATION**

Tracy Yuen^1^, Kimberle Shen^1^, Mike Reichelt^2^, Roxanne Kyauk^1^, Hai Ngu^2^, Yun-An Shen^1^, Oded Foreman^2^, Zora Modrusan^3^, Brad Friedman^4^, Morgan Sheng^1^

*^1^ Genentech, Inc., Department of Neuroscience, South San Francisco, USA*

*^2^ Genentech, Inc., Department of Pathology, South San Francisco, USA*

*^3^ Genentech, Inc., Department of Microchemistry, Lipidomics, and Proteomics, South San Francisco, USA*

*^4^ Genentech, Inc., Department of Bioinformatics and Computational Biology, South San Francisco, USA*

In neurological diseases such as Multiple Sclerosis (MS), the failure to repair demyelinated lesions contributes to axonal damage and clinical disability. We provide evidence that *Mertk*, a gene highly expressed by microglia that alters MS risk, is required for efficient remyelination. Compared to WT mice, Mertk-KO mice show impaired clearance of myelin debris and remyelination following demyelination. Using single-cell RNA-sequencing, we characterize *Mertk*-influenced responses to cuprizone-mediated demyelination and remyelination across different cell types. Mertk-KO brains show an attenuated microglial response to demyelination, but an elevated proportion of interferon-responsive microglia. In addition, we identify a transcriptionally-distinct subtype of oligodendrocytes specific to demyelinated lesions. The inhibitory effect of myelin debris on remyelination is mediated in part by IFNγ, which further impedes microglial clearance of myelin debris and inhibits oligodendrocyte differentiation. Together, our work establishes a role for *Mertk* in microglia activation, phagocytosis, and migration during the remye-lination process and identifies a new mechanism for myelin debris inhibiting remyelination.

## S-16 Enteric Nervous System Remodeling

S-16-01

**THE ENTERIC NERVOUS SYSTEM IN GUT HOMEOSTASIS**

Meenakshi Rao^1^

*^1^ Boston Children's Hospital, Division of Gastroenterology, Boston, USA*

*^2^ Harvard Medical School, Department of Pediatrics, Boston, USA*

Enteric glia are the largest population of glial cells outside the brain yet their phenotypic diversity, normal functions, and roles in disease are not well understood. In order to investigate enteric glial functions *in vivo,* previous studies targeted cells expressing glial fibrillary acidic protein (GFAP) for genetic ablation in mice. These studies found that enteric glia are essential for the regulation of intestinal epithelial barrier integrity and epithelial cell proliferation, and that glial dysfunction causes intestinal inflammation. While only a subset of enteric glia expresses GFAP, virtually all express proteolipid protein 1 (PLP1). We used the PLP1 promoter to drive expression of diphtheria toxin subunit A (DTA) in enteric glia and discovered that these cells are not essential for maintenance of intestinal epithelial barrier integrity or epithelial cell proliferation *in vivo,* contrary to long-standing beliefs. Using PLP1-DTA mice to probe glial functions *in vivo*, we are uncovering unexpected roles for enteric glia in many aspects of gut homeostasis, from motility to host defense.

S-16-02

**ENTERIC GLIAL CELLS AT CENTER STAGE OF DIGESTIVE DISEASES**

Malvyne Rolli-Derkinderen, Philippe Naveilhan, Pascal Derkinderen, Michel Neunlist

*Inserm U1235, Faculté de Médecine, Nantes, France*

Gone are the days when enteric glial cells (EGC) were considered merely as satellites of enteric neurons. Like their brain counterpart astrocytes, EGC express an impressive number of receptors for neurotransmitters and intercellular messengers, thereby contributing to neuroprotection and to the regulation of neuronal activity. EGC also produce different soluble factors that regulate neighboring cells among which are intestinal epithelial cells. A better understanding of EGC response to an inflammatory environment, often referred to as enteric glial reactivity, could help define the physiological role of EGC and the importance of this reactivity in maintaining gut functions. In chronic inflammatory disorders of the gut such as Crohn's disease (CD) and ulcerative colitis (UC), EGC exhibit abnormal phenotype and their neighboring cells are dysfunctional, but it remains unclear whether EGC are only passive bystanders or active players in the pathophysiology of both disorders. The aim of the current presentation is to review the physiological roles and properties of EGC, their response to inflammation, their role in the regulation of the intestinal epithelial barrier and to discuss the emerging concept of CD as being an enteric gliopathy.

S-16-03

**ENTERIC GLIA AND GUT EPITHELIAL FUNCTIONS IN HEALTH AND DISEASE**

Vladimir Grubisic^1^, Holger Eltzschig^2^, Brian Gulbransen^1^

*^1^ Michigan State University, Neuroscience program, Department of physiology, East Lansing, MI, USA*

*^2^ McGovern Medical School, University of Texas, Health Science Center at Houston, Houston, TX, USA*

The intestinal lining is the largest surface where the body meets the outside world and its proper functioning is important for gastroin-testinal health and overall well-being. Gut epithelium is controlled by extrinsic innervation and the enteric nervous system, an intricate network of neurons and glial cells residing in the gut wall. Enteric glia contribute to the acute regulation of gut reflexes such as motility and secretomotor functions, but their role in the intestinal epithelial barrier is unclear. While in vitro studies have demonstrated direct glial effects on the epithelial cells, different animal models of glial ablation have shown that enteric glia are either required or dispensable for epithelial barrier function. We found that enteric glia do not acutely regulate epithelial barrier in healthy mouse colon and hypothesized that enteric glia contribute to gut inflammation which may induce persistent gut dysfunction. Enteric glia express adenosine A2B receptors (A2BRs), but their function is not understood. We used *Sox10* ^CreERT2+/^ ;*Adora2b* ^f/f^ mice to ablate glial A2BRs and assessed the effects of acute colitis using 2% dextran sodium sulfate (DSS). Weight loss, macroscopic tissue damage, and the infiltration of CD45+ cells at the level of the myenteric plexus were comparable between *Sox10* ^CreERT2+/–^;*Adora2b* ^f/f^ mice and littermate controls (*Adora2b* ^f/f^). Colonic permeability was increased during the resolution of colitis in *Adora2b* ^f/f^ animals compared to their healthy controls and *Sox10* ^CreERT2+/–^;*Adora2b* ^f/f^ mice. DSS colitis induced persistent changes in the expression of tight junction protein transcripts and the loss of glial A2BRs normalized the expression of Claudin-1. The ablation of glial A2BRs protected against increases in proinflammatory mediators such as eotaxin-1, G-CSF, IL-1a, IP-10, KC, and MIG during acute colitis, and protected against changes in eotaxin-1, KC, IL-12(p40), and IL-17 during resolution. In conclusion, glial A2BR signaling modulates immune responses during acute colitis and its effects on downstream mechanisms may contribute to persistent gut epithelial barrier dysfunction.

## Colloquia

## C-01 Astrocyte-Neuron Interaction in Health and Disease

C-01-01

**TWO METABOLIC FUELS, GLUCOSE AND LACTATE, DIFFERENTIALLY MODULATE EXOCYTOTIC GLUTAMATE RELEASE FROM CULTURED ASTROCYTES**

Vladimir Parpura^1^, Vedrana Montana^1^, Daniel Flint^2^, Helle Waagepetersen^3^, Arne Schousboe^3^

*^1^ University of Alabama at Birmingham, Department of Neurobiology, Birmingham, AL, USA*

*^2^ Luxumbra Strategic Research, LLC, NA, Arlington, VA, USA*

*^3^ University of Copenhagen, 3Department of Drug Design and Pharmacology, Faculty of Health and Medical Sciences, Copenhagen, Denmark*

Astrocytes have a prominent role in metabolic homeostasis of the brain and can signal to adjacent neurons by releasing glutamate via a process of regulated exocytosis. Astrocytes synthesize glutamate *de novo* owing to pyruvate entry to the citric/tricarboxylic acid cycle via pyruvate carboxylase, an astrocyte specific enzyme. Pyruvate can be sourced from two metabolic fuels, glucose and lactate. Thus, we investigated the role of these energy/carbon sources in exocytotic glutamate release from astrocytes. Purified astrocyte cultures were acutely incubated (1 hour) in glucose and/or lactate-containing media. Astrocytes were mechanically stimulated, a procedure known to increase intracellular Ca^2+^ levels and cause exocytotic glutamate release, the dynamics of which were monitored using single cell fluo-rescence microscopy. Our data indicate that glucose, either taken-up from media or mobilized from the glycogen storage, sustained glutamate release, while the availability of lactate significantly reduced the release of glutamate from astrocytes. Based on further pharma-cological manipulation during imaging along with tandem mass spectrometry (proteomics) analysis, lactate alone, but not in hybrid fuel, caused metabolic changes consistent with an increased synthesis of fatty acids. Proteomics analysis further unveiled complex changes in protein profiles, which were condition-dependent and generally included changes in levels of cytoskeletal proteins, proteins of secretory organelle/vesicle traffic and recycling at the plasma mem-brane in aglycemic, lactate or hybrid-fueled astrocytes. These findings support the notion that the availability of energy sources and metabolic milieu play a significant role in gliotransmission.

C-01-02

**EXPLOITABLE AND CONTEXT-SPECIFIC ASTROCYTE MOLECULAR MECHANISMS**

Xinzhu Yu

*University of Illinois at Urbana-Champaign, Molecular and Integrative Physiology, Urbana, USA*

Astrocytes tile the central nervous system and are widely implicated in brain diseases, but the molecular mechanisms by which astrocytes contribute to brain disorders remain incompletely explored. By performing astrocyte gene expression analyses following 14 experimental perturbations of relevance to the striatum, we discovered that striatal astrocytes mount highly context-specific molecular responses. Through data mining, we also identified astrocyte pathways in Huntington's disease (HD) that were reciprocally altered with respect to the activation of striatal astrocyte G-protein coupled receptor (GPCR) signaling. Furthermore, selective striatal astrocyte stimulation of the G_i_-GPCR pathway *in vivo* corrected several HD-associated astrocytic, synaptic, and behavioural phenotypes with accompanying improvement of HD-associated astrocyte signaling pathways. Overall, our data show that astrocytes are malleable, utilising context-specific responses that can be dissected molecularly and used for phenotypic benefit in brain disorders.

C-01-03

**GLIAL AUTOPHAGY IN PARKINSON'S DISEASE**

Margaret Ho

*ShanghaiTech University, School of Life Science and Technology, Shanghai, China*

Glia are fundamental players in the nervous system and contribute to neuronal development, function, and degeneration. Our previous study implicated that neuronal *auxilin (aux)*, a clathrin-uncoating factor and homolog of *Cyclin-G-associated kinase (GAK)*, is a causative factor of Parkinson's disease (PD) using *Drosophila* as a model. Despite its prevalent expression in glia, whether glial *GAK/ aux* contributes to neurodegeneration remains elusive. Here we present evidence that glial *GAK/aux* is a new player in autophagy. *aux* contributes to PD via regulating glial autophagic clearance independent of its clathrin-uncoating activity. Downregulation of *aux* expression in glia specifically induces age-dependent lysosome and autophagosome accumulation, leading to impaired autophagic flux, autophagosome-lysosome fusion defects, and accumulation of auto-phagic substrate p62. Downregulation of *GAK* expression in immortalized microglial cells causes similar lysosome and auto-phagosome defects. Pathologically, downregulating glial *aux* expression results in age-dependent locomotor deficits, DA neuron loss, and shortened lifespan, all neurodegenerative phenotypes associated with PD. Taken together, our findings reveal that *GAK/aux* regulates autophagic degradation in glia, a possible route for clearing unwanted neuronal aggregates associated with neurodegeneration, demonstrating the importance of glia-neuron interaction during both normal and pathological conditions.

C-01-04

**THE GUIDING ROLE OF GFAP IN ASTROCYTIC PLASTICITY IN RAT HYPOTHALAMIC NEUROENDOCRINE REGULATION**

Yu-Feng Wang, Ping Wang, Tong Li, Yang Liu, Jiawei Yu, Dongyang Li, Dan Cui

*Harbin Medical University, Physiology, Harbin, China*

Neuronal activity is closely modulated by changes in astrocytic structures and functions, or astrocytic plasticity. Recently, glial fibrillary acidic protein (GFAP) has emerged as a critical component in the structural and functional plasticity of astrocytes. However, the mechanisms underlying GFAP-modulating astrocytic plasticity remain poorly understood. Here, using interactions between astro-cytes and magnocellular neuroendocrine neurons in rat supraoptic nucleus as a model, we studied how GFAP modulates astrocyte morphology and functions under suckling stimulation or osmotic challenges. The results showed that dynamic changes in GFAP filaments in astrocytes are accompanied by synchronous change in the spatial location of other membrane proteins, such as aquaporin 4 (AQP4), synaptobrevin or VAMP, and volume-regulated anion channel (VRAC). This GFAP plasticity is related to actin filament reorganization. Moreover, GFAP retraction is accompanied with increased molecular association between GFAP and action, particularly at somata and proximal processes. The extension of GFAP filaments requires the pulling of actin filaments at peripheral end of astrocyte processes as well as the volume expansion at the processes. The later effect requires normal functions of AQP4 and VRAC. Hence, changes in GFAP and other functional proteins in astrocytes may play a role of guiding the spatial localization of various functional proteins in cytoplasmic transportion and mem-brane protein cycling.

## C-02 Lipid metabolism: Regulation of Myelination in Health and Disease

C-02-01

**LIPID SIGNALING REGULATES CNS MYELINATION THROUGH THE STEROL REGULATORY ELEMENT BINDING PROTEIN (SREBP) PATHWAY**

Judith Grinspan^1^, Hubert Monnerie^1^, Micah Romer^1^, Sangwon Kim^2^, Kelly Jordan-Sciutto^3^

*^1^ Children's Hospital of Philadelphia, Department of Neurology, Philadelphia, USA*

*^2^ Johns Hopkins School of Medicine, Department of Medicine, Baltimore, USA*

*^3^ University of Pennsylvania, School of Dental Medicine, Philadelphia, USA*

Myelin is about 70% lipid, thus the ability of oligodendrocytes to manufacture lipids is key for myelination. In many tissues, sterol regulatory element-binding proteins (SREBPs) are master regulators that initiate transcription of lipid enzymes and ultimately synthesis of cholesterol and fatty acids. When sterol levels are low, SREBPs in precursor form are transported from the endoplasmic reticulum to the golgi to release the cleaved form which then translocates to the nucleus to initiate transcription. We have shown *in vitro* that SREBP activation is required for oligodendrocyte maturation, including process extension and myelin protein expression*. In vivo*, deletion of the SCAP gene, which transports SREBP to the golgi for cleavage, results in lack of myelination however*,* studies have shown that astrocytes are able to donate cholesterol and other sterols to oligo-dendrocytes. The necessity of SREBP for myelination suggests that pathological conditions that involve loss of SREBP cleavage and transport to the nucleus would affect differentiation of oligodendro-cytes progenitors. Our laboratory studies the white matter loss seen in HIV+ individuals who have HIV-associated neurocognitive disorder (HAND), even when their viral load is well-controlled by anti-retrovirals (ARVs). Thus, the ARVs themselves may play a role in the myelin deficits. In fact, the amount of white matter loss can be correlated with the amount of time on ARVs and a transcriptomic comparison of HAND+ individuals with or without ARVs shows that some myelin transcripts remain dysregulated even with ARV treatment. One major side effect of select ARV drugs is lipid dysregulation, including SREBP alterations, but this has not been studied in oligodendrocytes or in the CNS. Our studies on oligodendrocytes show that SREBP regulation is a target for these antiretrovirals and is one of the cellular processes dysregulated by select ART agents, thus likely compromising myelination.

C-02-02

**PEROXISOMAL METABOLISM AND CNS MYELINATION: INSIGHTS INTO ADRENOLEUKODYSTROPHY FROM A ZEBRAFISH MODEL**

Quentin Raas, Abigail Deschiffart, Freshner Briana, Stevenson Tammy, Stephens Miranda, Scholl Erika, Josh Bonkowsky

*University of Utah, Pediatric Neurology, Salt Lake City, USA*

Peroxisomal metabolism is essential to myelin turnover and maintenance, and disorders of peroxisomal function have characteristic severe white matter lesions of the nervous system. X-linked adrenoleukodystrophy (X-ALD), the most common peroxisomal disorder, is caused by mutations in the ATP-binding cassette transporter D1 (*ABCD1*) gene. X-ALD is characterized by substantial phenotype heterogeneity, ranging from the peripheral dying back axonopathy that affects adults, known as adrenomyeloneuropathy (AMN), to a severe cerebral inflammatory demyelinating form affecting boys during childhood (cALD). ABCD1 is involved in the import of very long chain fatty acids (VLCFAs) into the peroxisome for their degradation. VLCFAs, including both saturated and monounsaturated forms, are highly enriched in myelin. While VLCFAs are a sensitive biomarker for X-ALD, they do not predict AMN versus cALD phenotype, and in fact studies have shown that correction of VLCFA levels failed to protect against disease progression. X-ALD lacks treatment and the current mouse model only partially recapitulates phenotypes. We have generated ABCD1 mutants in the small vertebrate model system zebrafish (*Danio rerio*). Zebrafish ABCD1 mutants have elevated VLCFA levels; abnormal patterning and decreased numbers of oligodendrocytes with increased cell death in the CNS; hypomyelination in the spinal cord; and impaired motor function. Induction of human ABCD1 expression in oligodendro-cytes reduced embryonic apoptosis of these cells and improved motor function. We have developed a novel approach to discover therapeutics. Because zebrafish are small, develop quickly and are inexpensive, we are using them in a high throughput screen based on impaired motor behavior, to identify compounds that could rescue the myelination defect. The antimalarial, FDA-approved compound chloroquine has been identified from the behavioral screen. Chloroquine induces the expression of SCD1, shifting saturated VLCFA towards monounsaturated VLCFA and that strategy could be beneficial in X-ALD. Overall, the zebrafish model of X-ALD can provide relevant insights for additional work on X-ALD patho-genesis, and can lead to future clinical trials.

C-02-03

**SUBCELLULAR DIVERSION OF CHOLESTEROL BY NEUROPATHY-LINKED MUTATIONS IN PMP22**

Lucia Notterpek, Ye Zhou, David Borchelt

*Univ. of Florida Neuroscience Dept., McKnight Brain Institute, Gainesville, USA*

Abnormalities in lipid metabolism have been linked with hypomye-linating and de-myelinating neuropathies, indicating a critical role for cholesterol in myelin synthesis by oligodendrocytes and Schwann cells. In this study, we examined the subcellular trafficking of choles-terol in models of hereditary neuropathies associated with misex-pression of peripheral myelin protein 22 (PMP22). Previous studies in Schwann cells lacking PMP22 indicate alterations in cholesterol metabolism, which is associated with severe hypomyelination of peripheral nerves. Since overproduction of wild type (WT) and mutated forms of PMP22 also cause peripheral nerve pathology with myelin defects, we examined cholesterol metabolism in tissues and cells modeling these forms of neuropathy. In samples from homozygous Trembler J (TrJ) mice carrying a Leu16Pro mutation in PMP22, cholesterol was retained in the *Golgi* compartment.

Overproduction of the WT protein, which models Charcot-Marie-Tooth type 1A (CMT1A) neuropathies, triggered cholesterol sequestration to lysosomes and reduction of ATP-binding cassette transporter (ABCA1) -dependent cholesterol efflux. Conversely, lysosomal targeting of cholesterol by 1-2.5 µM U18666A treatment increased WT-PMP22 levels in lysosomes. Mutagenesis of a choles-terol recognition amino acid consensus (CRAC) sequence, or CRAC-domain, in human PMP22 lead to increased levels of PMP22 in the ER and Golgi compartments, along with higher cytosolic, and lower membrane-associated cholesterol. Importantly, cholesterol trafficking defects observed in PMP22-deficient Schwann cells were rescued by reintroduction of the WT-but not the CRAC-mutant-PMP22. We also observed that myelination deficits in dorsal root ganglia explants from heterozygous PMP22-deficient mice were improved by choles-terol supplementation. Collectively, these findings indicate that PMP22 is critical in cholesterol metabolism, and this mechanism is likely a contributing factor in PMP22-linked hereditary demye-linating neuropathies.

## C-03 Astroglial Metabolism and the Brain Noradrenergic System

C-03-01

**NORADRENERGIC REGULATION OF GLIAL ACTIVATION: RELEVANCE TO AD AND MS?**

Douglas Feinstein

*University of Illinois, Dept. Anesthesiology, Chicago, USA*

Aside from its role as a neurotransmitter, noradrenaline (NA) has other functions in the CNS. This includes restricting development of inflammatory activation, providing neurotrophic support to neurons, and providing neuroprotection against oxidative stress. In vitro studies have shown that NA regulates proinflammatory activation of glial cells, mediated in part through suppression of the NFkB signaling pathway. In recent years, it has become evident that dysregulation of NA levels or signaling contributes to a variety of neurological diseases and conditions including Alzheimer's disease (AD) and Multiple Sclerosis (MS). The basis for dysregulation in these diseases is often due to damage occurring to noradrenergic neurons present in the locus coeruleus (LC), the major source of NA in the CNS. LC damage is present in AD, MS, and a number of other diseases and conditions. In mouse models of AD, experimental lesion of the LC leads to increases in neuropathology and amyloid plaque burden, while treatment with drugs to increase NA or reduce LC damage reduce plaque burden. LC damage is also present in the EAE mouse model of MS, and raising NA levels reduces disease severity and inflammatory activation. Further understanding of these events will be of value for the development of treatments for AD, multiple sclerosis, and other diseases and conditions having a neuroinflammatory component.

C-03-02

**ASTROCYTES INTEGRATE LOCAL SENSORY AND BRAIN-WIDE NEUROMODULATORY SIGNALS IN THE MOUSE VISUAL CORTEX**

Matthew Holt

*VIB, VIB-KU Leuven Center for Brain and Disease Research, Leuven, Belgium*

Astrocytes play multiple functions in the central nervous system (CNS), from control of blood flow through to modulation of synaptic activity. Data from *ex vivo* experiments have suggested that transient increases in intracellular Ca^2+^ are key to controlling these functions. Paradoxically, responses to sensory stimuli recorded *in vivo* are usually weak and unreliable.

In this talk, I will present evidence suggesting that astrocytes in mouse visual cortex are primed to respond to local neuronal activity by the neuromodulator norepinephrine, released during heightened arousal. Primed astrocytes respond to neuronal activity, elicited by complex visual stimuli, in a classical retinotopic pattern. Hence, under physiological conditions, astrocyte activity is dependent on the integration of information across signaling modalities. Such activity adds an unexpected layer of complexity to astrocyte function and may enable astrocytes to specifically and subtly regulate local network activity and plasticity.

C-03-03

**ASTROGLIAL METABOLIC EXCITABILITY MEDIATED BY LACTATE AND NORADRENALINE**

Nina Vardjan^1,2^, Anemari Horvat^1,2^, Helena Haque Chowdhury^1,2^, Marko Kreft^1,2,3^, Robert Zorec^1,2^

*^1^ University of Ljubljana, Faculty of Medicine, Institute of pathophysiology, Ljubljana, Slovenia*

*^2^ Celica Biomedical, Laboratory of Cell Engineering, Ljubljana, Slovenia*

*^3^ University of Ljubljana, Biotechnical Faculty, Department of Biology, Ljubljana, Slovenia*

Astrocytes under stimulation exhibit predominantly glycolytic metabolism with lactate as the end glycolytic product despite the normal oxygen levels (*i.e.* aerobic glycolysis). Aerobic glycolysis in astrocytes is activated through astroglial surface receptors, including adrenergic receptors, coupled to calcium and/or cyclic adenosine monophosphate (cAMP) signaling pathways each contributing to astroglial glucose metabolism. Upon noradrenergic stimulation lactate produced in aerobic glycolysis is released from astrocytes and can be used as a neuronal fuel or acts as a signal in the brain. Recent studies suggested that astrocytes express low levels of the lactate GPR81 receptor that is in fat cells part of an autocrine loop, in which the G_i_-protein mediates reduction of cAMP. Whether extracellular lactate as a signal affects brain metabolism, in particular astroglial metabolism, is unclear. We measured the cytosolic levels of cAMP, calcium, glucose, and lactate in single isolated astrocytes using fluo-rescence resonance energy transfer (FRET)-based nanosensors. In contrast to fat cells, in astrocytes stimulation by extracellular lactate and the selective GPR81 agonists, like adrenergic stimulation, elevated intracellular cAMP and lactate, which was reduced by the inhibition of adenylate cyclase. GPR81 agonists increased cytosolic cAMP also in GPR81-knock out astrocytes, suggesting that the lactate effect is GPR81-independent and mediated by an alternative receptor-like mechanism that enhances aerobic glycolysis and lactate production via a positive feedback mechanism.

C-03-04

**NEURON-GLIA METABOLIC COUPLING : ROLE IN NEURONAL PLASTICITY AND NEUROPSYCHIATRIC DISORDERS**

Pierre Magistretti^1^

*^1^ King Abdullah University of Science and Technology, Biological, Environmental Science and Engineering Division, Thuwal, Saudi Arabia*

*^2^ UNIL/CHUV, Department of Psychiatry, Lausanne, Switzerland*

A tight metabolic coupling between astrocytes and neurons is a key feature of brain energy metabolism (Magistretti and Allaman, Neuron, 2015). Two basic mechanisms of neurometabolic coupling between neurons and astrocytes have been decribed : the glycogenolytic effect of VIP and of noradrenaline and the glutamate-stimulated aerobic glycolysis. Both mechanism of neuron-astrocyte metabolic coupling result in the release of lactate from astrocytes as an energy substrate for neurons (Magistretti and Allaman, Neuron, 2015; Magistretti and Allaman, Nat Neurosci Rev, 2018). L-Lactate is not only an energy substrate but also a signaling molecule for long-term memory consolidation and for maintenance of LTP (Suzuki et al, Cell, 2011). Beta-2 adrenergic receptor activation selectively located on astrocytes and resulting in glycogenolysis trigger this lactate-mediated effect on plasticity and memory (Gao et al, PNAS, 2016). L-lactate stimulates the expression of synaptic plasticity-related genes such as *Arc*, *Zif268* and BDNF through a mechanism involving NMDA receptor activity and its downstream signaling cascade Erk1/ 2 (Yang et al, PNAS, 2014). A transcriptome analysis in cortical neurons has shown that the expression of a total of 20 genes is modulated by L-Lactate; of these, 16 involved in plasticity and neuroprotection are upregulated and 4 involved in cell death are downregulated (Margineanu et al. Front. Mol Neurosci, 2018). This set of results reveal a novel action of L-lactate as a signaling mole-cule in addition to its role as an energy substrate (Magistretti and Allaman, Nat Neurosci Rev, 2018). These actions of L-Lactate are also relevant for animal models of neuropsychiatric disorders. Indeed we have shown that peripheral administration of lactate exerts anti-depressant-like effects in three animal models of depression (Carrard et al, Mol.Psy., 2016). Finally, we have shown that the transfer of L-Lactate from astrocytes to neurons plays a key role in an appetitive memory task involving the basolateral amygdala such as cocaine place preference in mice (Boury-Jamot et al. Mol Psy, 2016).

## C-04 Microglia-Neuron Interactions in Health and Disease

C-04-01

**MIDBRAIN MICROGLIA: UNIQUE CELL PHENOTYPES AND THEIR IMPACT ON NEURONAL HEALTH AND FUNCTION**

Lindsay De Biase^1^, Eric Moca^1^, Daniela Lecca^2^, Keenan Hope^1^, David Tweedie^2^, Hannah Sitoy^1^, Lindsay Masukawa^1^, Nigel Greig^2^

*^1^ University of California Los Angeles, Physiology Department, Los Angeles, USA*

*^2^ National Institute on Aging, Intramural Research Program, Baltimore, USA*

During aging, the function and resilience of neurons declines at different rates in different parts of the brain. The causes of this variability in neuronal susceptibility during aging are not known. Microglia support tissue homeostasis, modulate synaptic connections between neurons, and produce inflammatory factors, all of which can shape cognitive decline and disease susceptibility. Within the basal ganglia, a collection of deep brain nuclei that regulate movement and key forms of learning, we found that microglia in young adult mice exhibit striking regional differences in basic properties and functional states, raising the possibility that microglial capacity to shape neuronal health and synaptic function throughout the lifespan varies. In particular, microglia in the ventral tegmental area (VTA) and substantia nigra pars compacta (SNc) of young adult mice showed distinct cellular phenotypes compared to their counterparts elsewhere. During aging, we found that VTA/SNc microglia begin to proliferate months before microglia in other brain regions. These increases in resident microglia were associated with elevation in local levels of inflammatory factors, generating “pockets” of inflammation in the VTA/SNc as early as late middle age, which may profoundly impact surrounding dopamine neurons. We are currently using molecular, electrophysiological, and advanced imaging approaches to identify factors that underlie this premature VTA/SNc microglial aging and to define its impact on surrounding neurons. The aim of these studies is to identify strategies for modulating microglial phenotype to preserve the health of vulnerable populations of neurons within the brain.

C-04-02

**MICROGLIA REGULATE SYNAPTIC FORMATION, NEURONAL ACTIVITY, AND LOCAL SYNCHRONIZATION**

Junichi Nabekura

*National Institute for Physiological Sciences, Homeostatic Development, Okazaki, Japan*

Microglia constantly move their processes in the brain *in vivo* suggesting their surveillance of local brain structures. We attempted to understand the functional significance of this dynamic motility of microglial processes. First, we focused on the prima facie interaction between microglia and neurons *in vi*vo. Microglial processes selectively and regularly contacted onto synapses in the adult mouse cortex (Wake et al. 2009). Excitatory synaptic transmission was facilitated during microglial contact, but this was diminished by systemic LPS injection. In addition, the synchronous activity of neurons within the local cortical area was disrupted by both microglial ablation and LPS injection (Akiyoshi et al 2018). This finding suggests that microglial contact onto synapses not only modulates synaptic transmission, but also contributes to the generation of synchronicity of local cortical circuit activity. When we next examined the damaged brain, microglial contact onto synapses was more prolonged in duration and was frequently associated with the elimination of synapses (Wake et al 2009). In addition, microglia tended to extend their processes toward neuronal elements exhibiting damage and this appeared to rescue neurons by facilitating the suppression of neuronal excitotoxicity (Kato et al. 2016). On the other hand, in our examination of the immature brain, microglial contact onto neuronal dendrites induced the generation of synapses and cortical circuits (Miyamoto et al 2016). Thus, microglia adapt their modulatory action on neurons depending on the specific brain environment.

C-04-03

**ELUCIDATING NICHE-ASSOCIATED FUNCTION OF MICROGLIA BY LEVERAGING THE MOUSE RETINA**

Daniel Saban

*Duke Univ Med Ctr, Depts Ophthalmology and Immunology, Durham, USA*

A macrophage niche is not simply a location within a given tissue where these phagocytes reside or accumulate. Rather, it is a local system in which the microenvironment regulates the fate and function of these macrophages and in turn helps meet the dynamic needs of that tissue in health or disease. Elucidation of macrophage function in the context of its niche is a robust means to investigate the biology or pathobiology of a given tissue and how macrophages are contributing to it. However, very little is known in this regard. I will present how our lab has been leveraging scRNA-Seq of the mouse retina to identify niche-associated populations of microglia. I will also share our most recent work, which is defining the subretinal space in context of being an inducible microglial niche during retinal degeneration and explain what this novel concept is revealing about disease.

C-04-04

**NEURONAL ACTIVITY CONTROLS MICROGLIAL PROCESS DYNAMICS IN AWAKE MICE**

Long-Jun Wu

*Mayo Clinic, Department of Neurology, Rochester, USA*

Microglia are resident immune cells that dynamically survey the brain parenchyma. Microglial processes interact with neuronal dendrites; however, the role that neuronal network activity plays in regulating microglial dynamics is not entirely clear. Most studies of microglial dynamics have either utilized slice preparations or in vivo imaging in the anesthetized mice. Here we demonstrate that microglia in the awake mice survey a small territory and exhibit stunted process dynamics. By contrast, reduced neuronal activity under general anesthesia greatly increases microglial process extension and the area of territory surveyed. Similarly, reduction of local neuronal activity via sensory deprivation or optogenetic inhibition increases microglial process surveillance. Using pharmacological and chemogenetic approaches, we demonstrate that reduced norepinephrine signaling is necessary for the observed increases in microglial process surveillance. Thus, our results demonstrate that noradrenergic tone in the awake mice normally suppresses microglial process surveillance under basal physiological conditions. Our results therefore indicate the importance of awake imaging for studying microglia-neuron interactions and advance a “set point” theory for how neuronal activity influences microglial process dynamics.

## C-05 A Closer Look: Inherited Versus Acquired Disorders of Myelin

C-05-01

**MODULATING PROTEOLIPID PROTEIN AS A THERAPEUTIC STRATEGY FOR PELIZAEUS-MERZBACHER DISEASE**

Paul Tesar

*Case Western Reserve University, Genetics and Genome Sciences, Cleveland, USA*

Mutations in *proteolipid protein 1* (*PLP1*) result in failure of mye-lination and severe neurological dysfunction in the X-linked pediatric leukodystrophy Pelizaeus-Merzbacher disease (PMD). Most *PLP1* variants, including supernumerary copies and various point mutations, are fatal. However, *PLP1*-null patients and mice display comparatively mild phenotypes, suggesting that reduction of aberrant *PLP1* expression might provide a universal therapeutic strategy across PMD genotypes. Here we show effective *in vivo* suppression of *Plp1* in the severe *jimpy* (*Plp1*^jp^) point mutation mouse model of PMD. CRISPR-Cas9 mediated germline knockdown of *Plp1* in *jimpy* mice increased myelinating oligodendrocytes and restored nerve conduction velocity, motor function, and lifespan to wild-type levels, thereby validating *PLP1* suppression as a therapeutic approach. To evaluate the therapeutic potential of *Plp1* suppression in postnatal PMD mice, we tested antisense oligonucleotides (ASOs) that stably decrease mouse *Plp1* mRNA and protein *in vivo* in the central nervous system. Administration of a single intraventricular dose of *Plp1*-targeted ASOs to postnatal *jimpy* mice increased myelination, improved motor behavior, and extended lifespan through an 8-month endpoint. Collectively, these results support the development of *PLP1* suppression as a disease-modifying therapy for PMD. More broadly, we demonstrate that oligonucleotide therapeutics can be delivered to oligodendrocytes *in vivo* to modulate neurological function and lifespan, opening a new treatment modality for myelin disorders.

C-05-02

**CORTICAL REMYELINATION IN AN ACQUIRED MODEL OF MYELIN LOSS**

Jennifer Orthmann-Murphy^1^, Cody Call^3^, Gian Carlo Molina-Castro^3^, Herriet Hsieh^3^, Peter Calabresi^2^, Dwight Bergles^3^

*^1^ University of Pennsylvania, Neurology, Philadelphia, USA*

*^2^ Johns Hopkins University, Neurology, Baltimore, USA*

*^3^ Johns Hopkins University, Neuroscience, Baltimore, USA*

Multiple sclerosis (MS) is an acquired inflammatory demyelinating disorder associated with neurodegeneration. Cortical demyelination plays a critical role in the pathogenesis of the progressive form of MS, leading to physical and cognitive disability and brain atrophy. Although the cortex is capable of spontaneous remyelination in both multiple sclerosis and the cuprizone model of demyelination in mice, it is unknown whether the pattern of cortical myelination is re-established following injury. To determine the specificity of cortical myelin repair, we longitudinally monitored individual oligodendro-cytes and their myelin sheaths using in vivo two-photon microscopy in adult MOBP-EGFP mice given a cuprizone diet. This approach offers new opportunities for defining the mechanisms of oligo-dendrogenesis and axon recognition after myelin loss, as well as evaluating the effectiveness of potential therapeutics in vivo.

C-05-03

**MODELING OF TUBB4A ASSOCIATED LEUKODYSTROPHY**

Akshata Almad^1^, Sunetra Sase^1^, Alex Boecker^2^, Luis Garcia^1^, Erika Holzbaur^2^, Steve Scherer^2^, Adeline Vanderver^1,3^

*^1^ Children's Hospital of Philadelphia, Neurology, Philadelphia, USA*

*^2^ University of Pennsylvania, Physiology, Philadelphia, USA*

*^3^ University of Pennsylvania, Neurology, Philadelphia, USA*

Mutations occurring in the cytoskeletal gene TUBB4A (tubulin beta 4A) leads to a range of neurological disorders. Majority of the population is affected from Hypomyelinating Atrophy of Basal Ganglia and Cerebellum (H-ABC), a rare leukodystrophy resulting from the recurring mutation p. Asp249Asn (D249N) in *TUBB4A*gene. Patients exhibit dystonia, gait impairment and cognitive deficits due to loss of neurons and myelin in the basal ganglia and cerebellum. We have developed a novel knock-in mouse model harboring heterozygous (*Tubb4a*^*D249N/*+^) and the homozygous (*Tubb4a*^*D249N/D249N*^) mutation that recapitulates the progressive motor dysfunction with tremor, dystonia and ataxia seen in H-ABC. *Tubb4a*^*D249N/D249N*^mice have myelination deficits along with dramatic decrease in mature oligodendrocytes and their progenitor cells. Additionally, a significant loss occurs in the cerebellar granular neurons and striatal neurons in *Tubb4a*^*D249N/D249N*^mice. *In vitro* studies show decreased survival and dysfunction in microtubule dynamics in neurons from *Tubb4a*^*D249N/D249N*^mice. Thus *Tubb4a*^*D249N/D249N*^mice demon-strate the complex cellular physiology of H-ABC, likely due to independent effects on oligodendrocytes, striatal neurons, and cerebellar granule cells in the context of altered microtubule dynamics, with profound neurodevelopmental deficits. We have also developed a human induced pluripotent stem cell (iPSC) based model from patients with *TUBB4A*^*D249N*^mutation to generate iPSC derived medium striatal neurons (MSN). MSNs were successfully derived from control and *TUBB4A*^*D249N*^patient iPSCs, however early studies indicate decreased neuronal survival and increased apoptosis in *TUBB4A*^*D249N*^ patient derived neurons. Ongoing studies are focused on modeling the spectrum of TUBB4A associated mutations and understanding the cellular mechanisms and affected pathways in H-ABC. These studies will be important from basic science and pre-clinical perspective with human iPSC serving as a platform for future drug screening.

## C-06 Leukocyte Trafficking to the Inflamed CNS: Chemokines and Integrins

C-06-01

**CHEMOKINES ENHANCE TRANSCELLULAR BBB PERMEABILITY: A SNEAK ATTACK THROUGH CAVEOLAE**

Sarah Lutz

*University of Illinois at Chicago, Anatomy and Cell Biology, Chicago, USA*

Entry of pathogenic T cells into the central nervous system (CNS) causes disease in the multiple sclerosis (MS) animal model experimental autoimmune encephalomyelitis (EAE). Extravasation across the blood-brain barrier (BBB) occurs via transcellular trafficking within endocytic vesicles such as caveolae, or between endothelial cells by tight junction dissolution. However, signals targeting infiltrating cells to transcellular instead of paracellular migration remain unclear. Our data suggest that endothelial cell Caveolin-1 engage chemokine receptor positive T cells in luminal-to-abluminal trafficking into the CNS. Our findings suggest that chemo-kine-enhanced Caveolin-1 signaling may be a target for modulating BBB permeability.

C-06-02

**INDUCED CNS EXPRESSION OF CXCL1 INCREASES CORONAVIRUS-INDUCED DEMYELINATION VIA ENHANCED NEUTROPHIL ACCUMULATION**

Thomas Lane, Dominic Skinner, Amber Syage, Gema Olivarria

*University of California, Irvine, Neurobiology & Behavior, Irvine, USA*

Intracranial inoculation of the neuroadapted JHM strain of mouse hepatitis virus (JHMV) into susceptible strains of mice results in an acute encephalomyelitis and chronic immune-mediated demyelination. Previous studies from our laboratory have demonstrated an important role for neutrophils in contributing to demyelination in JHMV-infected mice. However, potential mechanisms by which neutrophils augment white matter damage have not been well characterized. Using JHMV infection of transgenic mice, in which expression of the neutrophil chemoattractant chemokine CXCL1 is under the control of a tetracycline-inducible promoter active within GFAP-positive cells, results in sustained CXCL1 expression within the CNS that correlates with increased neutrophil numbers in both the brain and spinal cord throughout disease. We used flow cyto-metry and protein analysis to characterize neutrophils that migrate to the CNS both morphologically and phenotypically before damage to CNS occurs. In addition, we performed single cell RNA sequencing (scRNAseq) on CD45+ cells isolated from the spinal cords of JHMV-infected transgenic mice to examine how sustained neutrophil recruitment into the CNS affects the immunological landscape during the damage phase of disease. Neutrophils that migrate to the CNS following JHMV infection have a distinct morphology that correlates with increased proinflammatory neutrophil associated protein levels. This study highlights the role of neutrophils in responding to murine coronavirus infection of the CNS and their sensitivity to the micro-environment which can induce morphologic and gene expression profile changes that correlate with an increase in the severity of demyelination.

C-06-03

**BLOCKADE OF CXCR2 PROMOTES THE RECRUITMENT OF A REPARATIVE MYELOID SUBSET TO THE INFLAMED CNS**

Benjamin Segal, Andrew Sas, Kevin Carbajal

*The Ohio State University, Neurology, Colombus, USA*

Studies in animal models have shown that, in certain settings, alternative types of inflammation can exert protective and/ or regenerative effects. These beneficial immune responses often involve myeloid cells that produce trophic factors and anti-, as opposed to pro-, inflammatory molecules. A mouse model that has been used to study inflammation-driven repair in the central nervous system (CNS) involves the administration of zymosan, a yeast cell wall extract, by intraocular ( i.o.) injection at the time of, our shortly following, retro-orbital optic nerve crush (ONC) injury. Normally, retinal ganglion cells (RGC, the neurons that give rise to the optic nerve) do not extend lengthy axons beyond a crush injury site; however, robust axonal growth occurs after induction of “sterile” vitreal inflammation via i.o. injection of zymosan. Although monocytes/ macrophages have most commonly been implicated in reparative immune responses in the dermis and other extra-CNS tissues, we found that neutrophils predominated zymosan-induced vitreal infiltrates. Treatment of mice with an anti-CXCR2 antisera following ONC and i.o. zymosan injection, in order prevent neutrophil migration to the eye, unexpectedly enhanced RGC axonal regeneration. Further analyses revealed that anti-CXCR2 impeded the infiltration of mature neutrophils into the posterior chamber of the eye, allowing the entry of a novel myeloid subset. Here, we characterize the phenotype of this pro-regenerative cell type and examine its mechanism of action.

C-06-04

**REGULATION OF LYMPHOCYTE ENTRY IN THE CNS BY INTEGRINS AND CHEMOKINES**

Estelle Bettelli, Simon Glatigny

*Benaroya Research Institute, Immunology, Seattle, USA*

CD4^+^ T helper (Th) cells play a central role in orchestrating protective immunity but also in driving the development of autoimmunity. Multiple Sclerosis (MS) is a human autoimmune disease of the central nervous system (CNS) characterized by the infiltration of inflammatory lymphocytes and myeloid cells into the brain and spinal cord, leading to demyelination, axonal damage, and progressive loss of motor functions. The release of T cells in the circulation and their migration in the central nervous system are tightly regulated processes. Integrin alpha 4 and Sphingosine-1 phos-phate receptor 1 (S1P_1_) neutralization are being used as disease modifying therapies in MS. Using experimental autoimmune encephalomyelitis (EAE) models, we have studied how the modulation of these pathways regulate the trafficking of T cells. Specifically, we will discuss how Itga4 and S1P1 modulation can selectively regulate the entry of Th1, Th17 and regulatory T cells in the CNS and the development of EAE.

## C-07 CRMPs in the CNS: Novel Roles in Health and Disease

C-07-01

**CRMP2 AND NEUROPILIN-1: FROM THE COVID-19 FOG A NEW PAIN TARGET EMERGES**

Rajesh Khanna

*University of Arizona, Pharmacology, Tucson, USA*

Severe acute respiratory syndrome coronavirus 2 (SARS-CoV-2) is the causative agent of COVID-19, a coronavirus disease that, as of April 14, 2021, has infected more than 137 million people and caused over 2.9 million deaths worldwide and paralyzed global economies. The pandemic continues unabated and certain aspects of the disease continue to baffle clinicians and researchers. Although over 150 vaccine candidates are in the preclinical or the exploratory stage of development, there is still much to learn from SARS-CoV-2. A remarkable finding, from our laboratory, was that the SARS-CoV- 2 Spike protein can reverse allodynia in a rodent model of neuro-pathic pain. We identifed and characterize a novel role of neuropilin receptor 1 (NRP-1), upstream of the cytosolic regulator collapsin response mediator protein 2 (CRMP2), in nociceptive processing. In this talk, I discuss: (1) the molecular gateways used by the virus to enter the nervous system, particularly the nociceptors; (2) the potentially analgesic effect of the SARS-CoV-2's spike protein results in a reduced pain response during infections, thus making this virus even more insidious; (3) testimonials from chronic pain patients who report transient pain loss during COVID-19. Prior to the ‘surprise’ emergence of the COVID-19 pandemic in December of 2019, the United States and parts of the World were mired by the opioid epidemic. Thus, the findings presented in this symposium are relevant to two current global health crises as emerging data suggest that the COVID-19 pandemic is likely to compound the opioid epidemic.

C-07-02

**TARGETING THE CRMP2-CLN6 COMPLEX IN BATTEN DISEASE**

Jill Weimer

*Sanford Research, Pediatrics and Rare Diseases Group, Sioux Falls, USA*

Neurons are dependent upon the transport of cargo along micro-tubule networks and delivery of this cargo is necessary to support neuronal development, differentiation, maintenance, and health. A novel protein complex, composed of CLN6: an ER-associated protein of unknown function implicated in Batten disease, CRMP2: a tubulin binding protein important in regulating neurite microtubule dynamics and transport, and KLC4: a classic microtubule binding motor protein, is required for vesicular transport in neurons. Loss of CLN6 alters CRMP2 interactions with other protein partners, disrupts ER-vesicle trafficking, and leads to reduced neurite outgrowth and complexity. Treatment with a CRMP2 modulating compound, lanthionine ketamine ester, partially restored these deficits in a mouse model of CLN6 deficiency, indicating that stabilization of CRMP2 interacting partners may prove beneficial in lieu of restoring the CCK complex. Taken together, these findings boast a novel mechanism of ER vesicle transport in the axon, and provide new insights into thera-peutic targets for neurodegenerative disease.

C-07-03

**THE NGR-FC PROTEIN DELIVERED BY HEMATOPOIETIC CELLS ENHANCES NEUROREPAIR IN A MODEL OF MS: INVOLVEMENT OF CRMP2**

Steven Petratos

*Monash University, Neuroscience, Melbourne, Australia*

Deletion of the *ngr1* allele limits the severity of experimental auto-immune encephalomyelitis (EAE) by preserving axonal integrity. However, whether this favorable outcome observed in EAE is a consequence of an abrogated neuronal-specific pathophysiological mechanism, is yet to be defined. Here we show that, Cre-loxP-mediated neuron-specific deletion of *ngr1* preserved axonal integrity, whereas its re-expression in *ngr1*^*−/-*^ female mice potentiated EAE-axonopathy. As a corollary, myelin integrity was preserved under Credeletion in *ngr1*^*flx/flx*^, retinal ganglion cell (RGC) axons whereas, significant demyelination occurred in the *ngr1*^*−/-*^optic nerves following the re-introduction of *NgR1*. Moreover, Cre-loxP-mediated axon-specific deletion of *ngr1* in *ngr1*^*flx/flx*^ mice also demonstrated efficient anterograde transport of fluorescently-labeled ChTxB in the optic nerves of EAE-induced mice. However, the anterograde transport of ChTxB displayed accumulation in optic nerve degenerative axons of EAE-induced *ngr1*^*−/-*^ mice, when *NgR1* was re-introduced but was shown to be transported efficiently in the contralateral non-rAAV2- transduced optic nerves of these mutant mice. We further identified that the interaction between the axonal motor protein, kinesin-1 and collapsin response mediator protein 2 (CRMP2) was unchanged upon Cre deletion of *ngr1*. Whereas, this Kinesin-1/CRMP2 association was reduced when *NgR1* was re-expressed in the *ngr1*^*−/-*^ optic nerves. We then utilized transplantable HSCs as a cellular delivery method of the NgR(310)ecto-Fc fusion protein a therapeutic fusion protein that has been successfully applied as a treatment in animal models of spinal cord injury and glaucoma. Animals transplanted with LV-NgR(310)ecto-Fc-overexpressing HSCs, recovered from the peak of neurological symptoms associated with EAE, exhibiting axonal regeneration and eventual remyelination in the white matter tracts. Our data suggest that NgR1 governs axonal degeneration in the context of inflammatory-mediated demyelination through the phos-phorylation of CRMP2 by stalling axonal vesicular transport. Moreover, axon-specific deletion of *ngr1* preserves axonal transport mechanisms, blunting the induction of inflammatory demyelination. We suggest that HSCs can be utilized as carriers of the therapeutic NgR-Fc protein for specific delivery into EAE lesions and can potentiate neurological recovery by limiting NgR1-dependent signaling, thereby enhancing neurorepair.

## Selected Oral Presentations

## OR-01 Oral Presentations 1

OR-01-01

**OVEREXPRESSING LOW-DENSITY LIPOPROTEIN RECEPTOR REDUCES TAU-ASSOCIATED NEURODEGENERATION IN RELATION TO APOE-LINKED MECHANISMS**

Yang Shi^1^, Prabhakar Sairam Andhey^2^, Christina Ising^3^, Kairuo Wang^1^, Lisa L. Snipes^2^, Kevin Boyer^2^, Stephanie Lawson^2^, Kaoru Yamada^4^, Wei Qin^5^, Melissa Manis^1^, Javier Remolina Serrano^1^, Bruno A. Benitez^5^, Robert E. Schmidt^2^, Maxim Artyomov^2^, Jason D. Ulrich^1^, David M. Holtzman^1^

*^1^ Washington University in St. Louis, Neurology, St. Louis, USA*

*^2^ Washington University in St. Louis, Pathology and Immunology, St.Louis, USA*

*^3^ German Center for Neurodegenerative Diseases, Neurodegenerative Diseases and Geriatric Psychiatry, Bonn, Germany*

*^4^ The University of Tokyo, Neuropathology, Tokyo, Japan*

*^5^ Washington University in St. Louis, Psychiatry, St.Louis, USA*

*APOE* is the strongest genetic risk factor for late-onset Alzheimer's disease. ApoE exacerbates tau-associated neurodegeneration by driving microglial activation. However, how apoE regulates micro-glial activation and whether targeting apoE is therapeutically beneficial in tauopathy is unclear. Here we show that overexpressing an apoE metabolic receptor LDLR (low-density lipoprotein receptor) in P301S tauopathy mice markedly reduces brain apoE, and ameliorates tau pathology and neurodegeneration. LDLR overex-pression in microglia cell-autonomously downregulates microglial *Apoe* expression, and is associated with suppressed microglial activation as is in apoE-deficient microglia. Both apoE-deficiency and LDLR-overexpression strongly drive microglial immunometa-bolism towards enhanced catabolism over anabolism, whereas LDLR-overexpressing microglia also uniquely upregulate specific ion channels and neurotransmitter receptors upon activation. ApoE-deficient and LDLR-overexpressing mice harbor enlarged pools of oligodendrocyte progenitor cells (OPCs), and show greater preservation of myelin integrity under neurodegenerative conditions. They also show less “disease-associated astrocyte” activation in the setting of tauopathy.

OR-01-02

**AQP4 STOP CODON READTHROUGH FACILITATES AMYLOID-Β CLEARANCE FROM THE BRAIN**

Darshan Sapkota, Joseph D. Dougherty

*Washington University School of Medicine, Department of Genetics, St Louis, USA*

Alzheimer's disease (AD) is initiated by the toxic aggregation of Amyloid beta (Aβ). Immunotherapeutics aimed at reducing Aβ are in clinical trials but with very limited success to date. Identification of orthogonal approaches for clearing Aβ may complement these approaches for treating AD. In the brain, the astrocytic water channel Aquaporin 4 (AQP4) is involved in clearance of Aβ, and the fraction of AQP4 found perivascularly is decreased in AD. Further, an unusual stop codon readthrough event generates a conserved C-terminally elongated variant of AQP4 (AQP4X), which is exclusively perivascular. However, it is unclear if the AQP4X variant specifically mediates Aβ clearance. Here, using AQP4 readthrough-specific knockout mice that still express normal AQP4, we determine this isoform indeed mediates Aβ clearance. Further, with high-throughput screening and counterscreening, we identify small molecule compounds that enhance readthrough of the AQP4 sequence, and validate a subset on endogenous astrocyte AQP4. Finally, we demonstrate these compounds enhance brain Aβ clearance *in vivo,* which depends on AQP4X. This suggests derivatives of these compounds may provide a viable pharmaceutical approach to enhance clearance of Aβ and potentially other aggregating proteins in neuro-degenerative disease.

OR-01-03

**‘A1' ASTROCYTES ARE NEUROTOXIC AFTER STROKE**

Todd Peterson^1^, Evan Brahms^2^, Alex Munch^3^, Maya Weigel^3^, Kenya Inoue^1^, Ben Barres^3^, Marion Buckwalter^2^, Shane Liddelow^4^

*^1^ University of North Carolina - Wilm, Psychology, Wilmington, USA*

*^2^ Stanford University, Neurology and Neurological Sciencese, Stanford, USA*

*^3^ Stanford University, Neurobiology, Stanford, USA*

*^4^ New York University, Neuroscience and Physiology, New York, USA*

Multiple different neurological disorders, like neurodegenerative disorders or neural insults such as stroke create a neuroinflammatory response that leads to glial cell activation. These cells change their conformation and take on multiple different phenotypes, causing them to lose much of their normal functions and take on an inflammatory role. Activation of microglia following injury leads to the conversion of astrocytes into an “A1” phenotype that is more neuro-toxic in (Parkinson's Disease and optic nerve crush models). Three cytokines, tumor necrosis factor (TNF), interleukin 1a (Il-1a), and complement component 1q (C1q) are released from microglia and have been determined to induce the A1 astrocyte phenotype. We prevented “A1” neurotoxic astrocyte formation after stroke with triple knockout (TNF-, Il-1a-, and C1q-) mice, and compared their neuroin-flammatory response and stroke size to wildtype mice. We hypothesized that inhibition of “A1” astrocytes in triple knockout mice would lead to less astrogliosis and reduced infarct size. Following distal middle cerebral artery occlusion, the infarct size of triple knockout mice was significantly less than wildtype mice at both 1 (*p*<0.05) and 7 days (*p*<0.0001) post-ischemia. We also found a reduction in astrogliosis in the triple knockout animals 7 days post-stroke (*p*<0.05). No significant reduction of infarct size (*p*=0.4343) was found 28 days post-dMCAO, but there was a reduction in astrogliosis (*p*<0.05) in the triple knockout animals. Removing “A1” neurotoxic astrocytes from the neuroinflammatory response to stroke led to a reduction in the glial response to stroke and reduced infarct volume. Understanding the neurotoxic phenotype of this astrocyte activation and its feedback role on other resident and infiltrating cells could lead to treatments to reduce an exacerbated neuroinflammatory response.

OR-01-04

**SPHINGOLIPID-DEPENDENT MEMBRANE ORGANIZATION AND SIGNALING ORCHESTRATING MYELIN REPAIR**

Sara Grassi, Simona Prioni, Chiara D'Aprile, Livia Cabitta, Paola Giussani, Laura Mauri, Alessandro Prinetti

*University of Milan, Department of Medical Biotechnology and Translational Medicine, Milan, Italy*

Recombinant human IgM22(rHIgM22) binds to myelin and oligo-dendrocytes(OLs) and promotes remyelination in mouse models of multiple sclerosis(MS). However, the target antigen and the signaling mechanisms through which rHIgM22 exerts its function are still unclear.

*In vitro* analysis revealed that rIHgM22 binds to sulfatide, but also to phosphatidylinositol, phosphatidylserine and phosphatidic acid. Moreover, the composition of the lipid microenvironment of its antigen can modulate the affinity of the antibody, suggesting that reorganization of lipid membrane might be relevant in its biological activity.

In rat mixed glial cells(MGC), rHIgM22 causes an increase in the levels of PDGFαR and induces a dose-dependent proliferative response in all the cells in the culture, with the most significant response associated with astrocytes. Moreover, rHIgM22 increases the production and release of sphingosine 1-phosphate(S1P) in MGC while total levels of ceramide remain unchanged. Furthermore, release of S1P is strongly reduced by a selective inhibitor of PDGFαR. Increased S1P production is not mediated by regulation of sphingosine kinase 1 and 2, instead, we observe a significant reduction of S1P lyase. Remarkably, rHIgM22 treatment does not induce changes in the production and/or release of S1P in astrocytes, but it increases its release in BV2 cells, suggesting that rHIgM22 indirectly influences the proliferation of astrocytes in MGCs, by affecting ceramide/S1P balance.

Analysis of the effect of rHIgM22 on glycosphingolipid metabolism in MGC and astrocytes revealed no significant effects on the lipid pattern, while in OPCs and OLs we observe an increase in the levels of different gangliosides, known for their ability to interact with and modulate the activity of different growth factor receptors.

Considering all this, we propose that rHIgM22 protective effects might be mediated by alterations of lipid-dependent membrane organization and/or signalling in different cell types present in the nice of MS lesions and that a complex cross talk between different cell types is underlying the ultimate repair effect elicited by this antibody.

OR-01-05

**ACUTE LOSS OF PROLIFERATING NG2+ CELLS AFTER SPINAL CORD INJURY CHRONICALLY ALTERS SCAR FORMATION AND AXON GROWTH**

Christina Marion^1^, Ping Wei^1^, Dana McTigue^1,2^

*^1^ Wexner Medical Center, Ohio State University, Department of Neuroscience, Columbus, USA*

*^2^ Ohio State University, Belford Center for Spinal Cord Injury, Columbus, USA*

The extent to which NG2^+^cells contribute to glial and fibrotic scar formation after spinal cord injury (SCI) is poorly understood. To selectively ablate dividing NG2^+^ cells responding to SCI, we utilized a novel transgenic mouse line in which cells expressing NG2 also express a thymidine kinase from herpes simplex virus (NG2-tk mice). Intraventricular administration of antiviral agent ganciclovier (GCV) causes apoptosis in dividing NG2^+^cells (including oligodendrocyte progenitors and pericytes). Immediately following unilateral white matter-specific C5 SCI in NG2-tk mice, a drug delivery pump was placed subcutaneously with attached catheter inserted into the ventricle to administer GCV or saline for 14 days. Our prior work showed ablation of NG2^+^ cells significantly altered density and distribution of glial and fibrotic scars through 21d post-injury. Compared to controls, these alterations prolonged hemorrhage, enhanced edema, and impaired forelimb recovery, but also increased axons entering the lesion area (Hesp et al., 2018). The present study assessed long-term consequences of acute NG2^+^cell ablation. Drug pumps were removed after 14 days, allowing a recovery period before assessment at sixor eight-weeks post-SCI. In contrast to earlier time points, overall density of GFAP in the glial scar and laminin within the fibrotic scar did not significantly differ between NG2^+^ cell-ablated and non-ablated mice. However, there were differences in scar density and patterning, suggesting altered recovery after GCV cessation. A unique pattern emerged in the gray matter adjacent to the lesion, with significantly increased laminin profiles resembling blood vessels in NG2^+^ cell-ablated mice at both time points. Consistent with acute findings, more axonal profiles were maintained within lesions in GCV-treated mice. Iimpaired motor recovery also persisted through 8w post-injury. Clarifying the roles of NG2+ oligodendrocyte progenitors versus pericytes will be important to further define the contributions of each cell type to beneficial and deleterious cellular responses to CNS injury.

## OR-02 Oral Presentations 2

OR-02-01

**MULTI-SCALE ANALYSIS OF ASTROCYTE MORPHOLOGY AND ULTRASTRUCTURE IN HEALTHY AND ALZHEIMER'S DISEASE MODEL MICE**

Alexandra Schober^1^, Amy Zhou^1^, Yasmina Richa^1^, Tatiana Tibuleac^1^, Braxton Phillips^1^, Rachel Tooth^1^, Maxine Wu^1^, Gavin Frame^1^, J. Benjamin Kacerovsky^1^, Christopher K. Salmon^1^, Tabish A. Syed^2^, Kaleem Siddiqi^2^, Keith K. Murai^1^

*^1^ RI-MUHC, Centre for Research in Neuroscience, Montreal, Canada*

*^2^ McGill University, School of Computer Science & Centre for Intelligent Machines, Montreal, Canada*

Astrocytes comprise a highly complex cell population with diverse structural and functional properties in both the healthy and diseased central nervous system. Astrocytic pathology has been implicated in numerous neurodegenerative diseases, however, their structural rearrangement in brain diseases such as Alzheimer's disease (AD) remain poorly understood. Here, we applied super-resolution imaging and 3-dimensional (3D) electron microscopy approaches to better understand the structural changes of astrocytes in AD model (hAPP/ PS1) mice. Structured illumination microscopy (SIM) of genetically-labeled astrocytes allowed us to capture global modifications to individual astrocyte shape while resolving certain aspects of their subcellular organelle distribution. We observed perturbations to the complex branching pattern of whole astrocytes and additionally, mapped the organization of mitochondria throughout the cell in AD model samples. Results from SIM imaging were complemented with analysis using focused ion beam scanning electron microscopy (FIB-SEM) that enabled 3D serial reconstruction of astrocyte ultrastructure. Using FIB-SEM, we found dramatic restructuring of astrocyte subcompartments in neuropil and astrocytic endfeet, including major alterations of astrocytic process shape and the restructuring/redistribution of mitochondria. Quantitative analysis of SIM and FIB-SEM approaches allowed us to directly compare the astrocytic surface structure and the organelle distribution/ morphology within astrocyte sub-compartments using custom MATLAB scripts, in healthy and AD model tissue. This multi-level structural analysis of astrocytes provides an important understanding of how astrocytes are constructed in the healthy brain and altered with brain disease.

OR-02-02

**TAUOPATHY-INDUCED GLIOVASCULAR DYSFUNCTION IDENTIFIED BY CELL-SPECIFIC VIRAL TRAP-SEQ**

Yutaro Komuro, Mitchell Rudd, Michal Machnicki, Bianca Yugar, S. Thomas Carmichael, Jason Hinman

*UCLA, Neurology, Los Angeles, USA*

Increasing evidence suggests that the inciting pathogenic events in Alzheimer's disease may involve disruption of the multicellular interactions within the neurovascular unit. The interruption of specific molecular pathways within the neurovascular unit occurs antecedent to classic pathology and therefore is an attractive target to modulate neurodegeneration. However, the majority of molecular pathways regulating neural and gliovascular signaling remain unknown.Single-cell and whole tissue gene expression approaches have been used in various animal models of dementia to discern new pathways but are limited by sequencing depth and spatiotemporal accuracy of transcriptional profiling. We developed an approach for identifying relevant and novel pathways within the multicellular environment of the neurovascular unit interactions in Alzheimer's disease. Cell-specific viral constructs coding for antigen-tagged ribosomes (TRAP) were used to study variance in spatial and temporal gene expression patterns within and between cell types in the PS19 (P301S) transgenic model of tauopathy. The engineered lentiviruses and adeno-associated viruses express an HA-tagged ribosomal protein (Rpl10a-HA) driven by a cell-type specific promoter (Synapsin, GFAP, or PDGFRa) for the major cell types of the neurovascular unit. We demonstrated the ability of the viruses to target specific cell types under particular spatiotemporal conditions and confirmed cell-specificity *in vivo* using both known cell-specific markers and novel markers identified by sequencing. We then combined this cell-specific vTRAP system with the PS19 mouse model of tauopathy to generate multiple cell-type specific transcriptomic databases of the neurovascular unit across several pathologically-relevant time points. Gene ontology analysis indicates a prodromal drive by gliovascular cells including pericytes and astrocytes to support injured neurons. This occurs through specific and temporally regulated coordination of multiple molecular programs driving neuronal differentiation, neurite outgrowth, and synaptogenesis. This suggests a paradigm for gliovascular rescue of neurodegeneration phenomena and implicates numerous novel perivascular molecular pathways in the pathogenesis of tauopathy and Alzheimer's disease.

OR-02-03

**PROTECTIVE ROLE OF SLC4A4 IN ASTROCYTE-BLOOD-BRAIN-BARRIER (BBB) INTEGRITY IN HEALTHY AND ISCHEMIC STROKE BRAIN**

Qi Ye^1,2^, Juyeon Jo^1,2^, Chih-Yen Wang^1,2^, Hyun Kyoung Lee^1,2^

*^1^ Baylor College of Medicine, Pediatric Neurology, Houston, USA*

*^2^ Jan and Dan Duncan Neurological Research Institute at Texas Children's Hospital, Pediatric Neurology, Houston, USA*

Astrocytes provide diverse support for brain function by maintaining essential interactions with endothelial cells to form the BBB. Pathological astrocyte-BBB interactions contribute to ischemic stroke, which is the 5^th^ leading cause of death in the US. However, the mechanisms underlying maintenance of BBB integrity both in health and diseases such as stroke remain poorly defined. our study focuses on an astrocyte-enriched sodium-bicarbonate cotransporter, Slc4a4, which was previously identified as an astrocyte-specific regulator of both intracellular and extracellular pH. While pH homeostasis is essential for metabolic activity in the brain, the role of Slc4a4 in astrocyte-BBB integrity remains unknown. To address the role of Slc4a4 in this context, we generated new transgenic mouse lines that temporally ablate Slc4a4 in astrocytes. Using this genetic mouse model, we show loss of Slc4a4 in adult significantly dampens astrocyte endfeet Ca^2+^ signaling and generates enlarged blood vessels with disrupted endothelial junctions. Combination of transcriptome, proteomic and metabolomic profiling of Slc4a4-ablated astrocytes and conditioned media (CM) of Slc4a4- ablated astrocytes reveal dysregulation of genes associated with vasculature-BBB maintenance, inflammatory chemo-cytokines, and glial metabolism, further supporting a crucial role for Slc4a4 in BBB integrity by governing astrocyte-endothelia cell crosstalk. Among differentially expressed genes associated with Slc4a4 deletion, we confirm the upregulation of Ccl2 (C-C Motif Chemokine Ligand 2) in the CM of Slc4a4-ablated astrocytes and Slc4a4-deficient brain. We further validate the Slc4a4-Ccl2 axis using *in vitro* BBB model and find that blocking Ccl2 pathway restores endothelial junction and permeability. Using a mouse model of stroke, we found loss of Slc4a4 exacerbates stroke-induced motor dysfunction, mortality and BBB disruption coupled with impaired reactive gliosis. Together, our study indicates the indispensable role of Slc4a4 mediated astrocyte-BBB interaction, providing insights for the potential of glia meta-bolism regulators as novel therapeutic approach for BBB-related CNS disorders.

OR-02-04

**LIPOXYGENASE MEDIATORS AND MICROSTRUCTURAL WHITE MATTER INTEGRITY IN ALZHEIMER'S DISEASE AND SUBCORTICAL ISCHEMIC VASCULAR DISEASE**

Julia Zebarth^1,2^, Di Yu^1,2^, Fenghua You^1,2^, Joel Ramirez^1^, Pak Cheung Chan^1,2^, Mario Masellis^1,2^, Richard Swartz^1,2^, Krista L. Lanctôt^1,2^, Nathan Herrmann^1,2^, Demitrios J. Sahlas^3^, Jacqueline A. Pettersen^4^, Sandra E. Black^1,2^, Ameer Taha^5^, Walter Swardfager^1,2^

*^1^ Sunnybrook Research Institute, Hurvitz Brain Sciences Program, Toronto, Canada*

*^2^ University of Toronto, Pharmacology and Toxicology, Toronto, Canada*

*^3^ McMaster Univeristy, Medicine, Hamilton, Canada*

*^4^ University of Northern British Columbia, Medicine, Prince George, Canada*

*^5^ UC Davis, Food Science and Technology, Davis, USA*

Linoleic acid derived lipoxygenase hydroxyoctadecadienoic acid metabolites (9-HODE and 13-HODE) have anti-inflammatory properties but their clinical associations with Alzheimer's disease (AD) and subcortical ischemic vascular disease (SIVD) have not been established. We aim to examine these metabolites with respect to microstructural white matter integrity and vascular brain lesion characteristics. Patients from memory and stroke prevention clinics (n=69) were stratified based on SIVD (minimal vs. abundant white matter hyperintensities [WMH] based on 3.0T structural MRI), and AD (clinical diagnosis). 9-HODE and 13-HODE were extracted from serum using solid phase extraction and concentrations measured by ultra high-performance liquid chromatography mass spectrometry. Microstructural brain tissue integrity was examined as fractional anisotropy (FA) and mean diffusivity (MD) using diffusion tensor imaging. Using multivariate analysis of covariance controlling for age and sex, 13-HODE was lower in people with AD (F_1,68_=6.52, p=0.013, n=23) but not with SIVD (F_1,68_=0.14, p=0.7, n=38). 13-HODE was associated with higher FA in normal appearing white matter (β=0.303, p=0.017), negatively associated with periventricular WMH-FA (β=-0.381, p=0.048), and positively associated with with periventricular WMH-MD (β=0.257, p=0.048). In patients without AD, 9-HODE and 13-HODE were negatively associated with WMH volume (β=-0.322, p=0.018 and β=-0.324, p=0.019). Additionally, 9- HODE was positively associated with FA in normal appearing white matter (β=0.341, p=0.041) and periventricular WMH-MD (β=0.381, p=0.025). Anti-inflammatory linoleic acid oxylipins were associated with preserved white matter integrity, smaller vascular brain lesion volumes, and vascular brain lesion microstructural characteristics. AD was associated with a relative deficit in these mediators.

OR-02-05

**ENDOPLASMIC RETICULUM STRESS INITIATES JANUS KINASE (JAK) 1-DEPENDENT GENE EXPRESSION IN ASTROCYTES**

Savannah Sims^1^, Gordon Meares^1,2^

*^1^ West Virginia University, Immunology, Microbiology, and Cell Biology, Morgantown, USA*

*^2^ West Virginia University, Neuroscience, Morgantown, WV, USA*

Neurodegenerative diseases are associated with misfolded proteins in the endoplasmic reticulum (ER) and dysregulated immune signaling. ER stress occurs when the protein folding capacity of the ER is overwhelmed, resulting in initiation of the unfolded protein response (UPR) to restore homeostasis. Unresolved UPR activation leads to cell death and inflammation. We have described an ER-stress induced Janus Kinase (JAK) 1 - Signal Transducer and Activator of Transcription (STAT) 3-dependent mechanism that promotes expression of inflammatory mediators including Interleukin-6 (IL-6). JAK1 is initiated by cytokine stimulation to promote inflammatory gene expression, and additionally, using RNA-seq, we have shown JAK1 controls many ER stress-induced genes. We found that less than 10% of the ER stress-induced JAK1-dependent genes are also induced by cytokine stimulation, demonstrating ER stress and cyto-kine stimulation induce distinct JAK1-dependent transcriptional profiles in astrocytes, a nonneuronal brain cell that responds to insult by producing soluble immune mediators. We found that JAK1 controls expression of genes that are not regulated by STATs, but by activating transcription factor (ATF) 4. We have shown that JAK1 and ATF4 physically interact and, via chromatin immunopre-cipitation (ChIP), in response to ER stress, ATF4 is effectively recruited to these promoters of these genes in a JAK1-dependent manner suggesting that JAK1 is involved in directing ATF4-dependent gene expression in a gene specific manner. These findings suggest JAK1 is a major driver of transcription in response to cellular stress, and JAK1 exhibits novel signaling mechanisms to regulate gene expression through ATF4.

OR-02-06

**ADVANCED TAU PATHOLOGY AND PRION-LIKE PROPAGATION IN A MONKEY MODEL OF ALZHEIMER'S DISEASE**

Danielle Beckman^1^, Sean Ott^1^, Amanda Dao^1^, Eric Zhou^1^, Kristine Donis-Cox^1^, William Jansen^2^, Scott Muller^3^, Paramita Chakrabarty^4^, Jeffrey Kordower^3^, John Morrison^1,5^

*^1^ University of California - Davis, California National Primate Research Center, Davis, USA*

*^2^ Icahn School of Medicine at Mount Sinai, Friedman Brain Institute, NY, USA*

*^3^ Rush University Medical Center, Department of Neurological Sciences, Chicago, USA*

*^4^ University of Florida, Center for Translational Research in Neurodegenerative Disease, Gainesville, USA*

*^5^ University of California - Davis, Department of Neurology, School of Medicine, Davis, USA*

Alzheimer's disease (AD) is a devastating condition that affects around 5.8 million of Americans, and these numbers are projected to reach nearly 14 million individuals around 2050. Although AD was first described more than 100 years ago, until nowadays that are not effective treatments to counteract or efficiently slow down the progression of the disease. This is likely, due at least in part, to the fact that AD animal models have been focused in the use of transgenic rodent models, failing to translate the findings to humans with the disease. We describe here a non-human primate model of AD presenting extensive tau pathology, mainly affecting the hippocampus and connected areas. Adult rhesus monkeys injected with adeno-associated viruses expressing mutant tau (P301L/S320F) in the left entorhinal cortex (ERC) show after 3 months, extensive prion-like tau propagation with full fibrillary tangles in the hippocampus, as well as pre-tangles and phospho-tau in projection areas such as the contralateral ERC and in the retrosplenial and visual cortices. Importantly, neuroinflammation is extensive in the affected areas, with a major role of microglia in pathological tau oligomer pro-pagation. Finally, the mutant h4R injected coaptates monkey 3R tau generating more tau aggregation and spreading. These results highlight the first stages of tau pathology and propagation and support the importance of a non-human model of AD, with natural full expression of tau protein, that is highly translational to humans.

## OR-03 Oral Presentations 3

OR-03-01

**NEURONS AND ENDOTHELIAL CELLS INDUCE ASTROCYTE MATURATION**

Zila Martinez-Lozada^1^, William Todd Farmer^2^, Keith K. Murai^2^, Michael B. Robinson^1^

*^1^ Children's Hospital of Philadelphia, Pediatrics, Philadelphia, USA*

*^2^ McGill University, Centre for Research in Neuroscience, Montreal, Canada*

Astrocytes are positioned between neurons and blood vessels with fine processes that oppose synapses and endfeet that enwrap the vasculature. This localization allows astrocytes to participate in several aspects of brain homeostasis, including glutamate clearance, but it also permits neurons and endothelia to influence astrocyte biology. Astrocytes express two subtypes of glutamate transporters. The expression of one of these transporters, GLT1/EAAT2, increases dramatically during synaptogenesis, providing a surrogate marker of astrocyte maturation. We and others have demonstrated that astro-cytes cultured alone express little or no GLT1 and that co-culturing with neurons or endothelia induces expression of GLT1 in astrocytes. While several signaling pathways have been implicated in neuron-dependent induction, we previously showed that Notch is required for the effect of endothelia. Our research has two goals: 1) determine which components of Notch signaling mediate the effect of endo-thelia, and 2) determine how neurons and endothelia synergistically affect the transcriptome of astrocytes. To address the first goal, we cultured cortical astrocytes in the presence or absence of the mouse endothelioma cell line bEND.3. Using recombinant Notch ligands, neutralizing antibodies, and shRNAs directed against Notch ligands in bEND.3 cells, we found that both delta-like Notch ligands, DLL1 and DLL4, are involved in endothelial-dependent induction of GLT1. Our studies also showed that astrocytes increase expression of DLL4 in endothelia. This now published study showed that reciprocal communication between astrocytes and endothelia contributed to the maturation of both cells (Martinez-Lozada & Robinson Neurochem Int 2020). To address the second goal, we cultured cortical astrocytes alone or in the presence of bEND.3 or rat cortical neurons, or both. We isolated astrocytes using fluorescence-activated cell sorting and performed RNA-seq and bioinformatic analysis. We found that neurons and endothelia have both complementary/additive and competitive effects on the astrocyte transcriptome. Overall, our study indicates that astrocytes receive an intricate combination of signals from neurons and endothelial cells which ultimately dictate astrocyte biology.

OR-03-02

**ENDOTHELIN-1 SIGNALING REGULATES NEURAL STEM CELLS IN THE HEALTHY AND INJURED ADULT BRAIN**

Katrina Adams^1^, Payal Banerjee^1^, Marianna Bugiani^2^, Vittorio Gallo^1^

*^1^ Children's National Medical Center, Center for Neuroscience Research, Washington, USA*

*^2^ VU University Medical Center, Department of Pathology, Amsterdam, Netherlands*

Demyelination occurs in a large variety of central nervous system (CNS) insults, pathologies, and neurodegenerative diseases, including Multiple Sclerosis (MS). Parenchymal oligodendrocyte progenitor cells (OPCs) located throughout the CNS participate in endogenous remyelination of white matter lesions, migrating to the injury site and maturing into myelinating oligodendrocytes, albeit at low levels. An additional source of OPCs following CNS injury is neural stem cell (NSC)-derived OPCs generated in the subventricular zone (SVZ) of the lateral ventricles. Therefore, promoting increased levels of NSC gliogenesis is a promising therapeutic strategy for demyelinating disorders like MS. We previously identified the Endothelin-1 (ET-1) signaling pathway as a novel regulator of NSC and OPC proliferation during early postnatal development (Adams et al. 2020). Therefore, we asked whether ET-1 also regulates stem and progenitor cells in the adult SVZ, both during homeostasis and after demyelinating injury. We found that the majority of NSC populations in the adult mouse SVZ express the ET-1 receptor, Ednrb. Ablation of Ednrb from NSCs reduced both the number of activated and quiescent NSCs, indicating that ET-1 signaling is required for maintenance of NSCs in the adult mouse. Following focal demyelination of the corpus callosum, SVZ NSCs upregulated expression of ET-1. Ablation of ET-1 reduced the percentages of proliferating NSCs and proliferating OPCs in the SVZ, suggesting that ET-1 plays a critical role in the SVZ proliferative response to injury. RNAseq of cultured primary NSCs and OPCs treated with ET-1 identified genes involved in stem cell maintenance, including Notch signaling, and OPC migration. Lastly, we confirmed that ET-1 and EDNRB expression are conserved in the adult human SVZ, indicating that this pathway may be a potential target for promoting SVZ-mediated cellular repair.

OR-03-03

**CORTICAL NEURON DENDRITE DEVELOPMENT REQUIRES THE LVS MOTIF OF PLEXINA4 RECEPTOR, AND TRANSCRIPTIONAL AND TRANSLATIONAL REGULATION**

Oday Abushalbaq, Tracy S. Tran

*Rutgers University, Department of Biological Sciences, Newark, New Jersey, USA*

Neuronal circuit development is a complex process consisting of precise steps in the directional growth and maturation of axons and dendrites to synapse formation and elimination. The development of axons and dendrites, in particular, is largely dependent on extra-cellular guidance cues. The large family of Semaphorin molecular cues is known to regulate axon guidance, dendrite elaboration and synapse elimination in the developing mammalian nervous system. In particular, the class 3 secreted Semaphorin 3A (Sema3A), through its receptor complex Neuropilin-1 (Nrp1)/PlexinA4 (PlxnA4), has been shown to induce dendritic elaboration in cortical neurons *in vitro* and *in vivo*. Previously, we showed that the KRK amino acid motif in the PlxnA4 receptor is required for cortical dendritic elaboration through the interaction with the RhoGEF FARP2 and activation of Rac1. However, it is not known whether other signaling motifs in the PlxnA4 receptor could also mediate dendrite development. Here, we show that the LVS motif, located in the Rho GTPase binding domain of PlxnA4, is required for Sema3A-mediated den-dritic elaboration in cortical neurons. Moreover, our findings show for the first time that gene transcription and protein translation are required in Sema3A-induced dendritic elaboration of cortical neurons *in vitro* during the initial stages of dendritic elaboration, but not later stages. Next, we will identify novel downstream gene targets of Sema3A-induced dendrite elaboration by using RNA sequencing in our established primary cortical neuron system and *in vivo* using specific genetically modified mouse lines. Taken together, our study provides novel insights to the intracellular mechanisms underlying Sema3A-mediated dendritic morphogenesis in cortical neuron development, and dissects the multifunctional roles of the PlxnA4 receptor in the proper formation of neuronal connections.

OR-03-04

**TGFBETA1 DEPENDENT GPNMB EXPRESSION IN NEURAL STEM CELLS INHIBITS OLIGODENDROGENESIS**

Daniel Radecki, Jayshree Samanta

*University of Wisconsin-Madison, Comparative Biosciences, Madison, USA*

Demyelination of the central nervous system (CNS) is a pathological feature of many neurodegenerative diseases, and leads to altered signaling, axonal stress, and neurodegeneration. Multiple Sclerosis (MS) presents with widespread demyelination, which is refractory to current treatments and interventions. Recent research identified a population of adult neural stem cells (NSCs) that express the transcription factor Gli1 in the adult subventricular zone (SVZ) and are capable of generating new oligodendrocytes for remye-linating the CNS. Loss of Gli1 in these NSCs further increases their recruitment to the site of demyelination and differentiation into mye-linating oligodendrocytes, with functional improvement in an Experimental Autoimmune Encephalomyelitis (EAE) model of MS. To understand the molecular underpinnings of enhanced remyelina-tion, we performed an RNAseq screen in NSCs with loss of Gli1 from demyelinated brain and identified Glycoprotein non-metastatic melanoma b (Gpnmb) as the most differentially expressed gene with ∼6 fold higher expression in wildtype NSCs. In the healthy brain, Gpnmb is expressed in subsets of NSCs, microglia, astrocytes, oligo-dendrocyte precursors (OPCs), and neurons but not mature oligodendrocytes. Global knockout of Gpnmb results in an increase in mature oligodendrocyte generation during remyelination, while over-expression of Gpnmb *in vitro* suppresses oligodendrocyte gene expression; thus indicating that Gpnmb inhibits remyelination. We also found that TGFß1 secreted from the site of demyelination increases Gpnmb expression in NSCs. In addition, increased Gpnmb levels stimulate TGFß-R2 expression, the ligand binding subunit of the TGFß1 receptor dimer, indicating Gpnmb may function in a feed-forward loop to sensitize NSCs to TGFß1. Taken together, our data strongly implicate Gpnmb as a negative regulator of remyelination that is regulated by and feeds forward to amplify TGFß1 signaling.

OR-03-05

**DIVERSE GABAERGIC NEURONS ORGANIZE INTO SUBTYPE-SPECIFIC LAMINAE IN THE VENTRAL LGN**

Ubadah Sabbagh^1,2^, Jessica Wei^3^, Ryan Ha^1^, Rachana Somaiya^1,2^, Michael Fox^1,4,5^

*^1^ Fralin Biomedical Research Institute at VTC, Center for Neurobiology Research, Roanoke, VA, USA*

*^2^ Virginia Tech, Graduate Program in Translational Biology, Medicine, and Health, Blacksburg, VA, USA*

*^3^ Fralin Biomedical Research Institute at VTC, neuroSURF program, Roanoke, VA, USA*

*^4^ Virginia Tech, Biological Sciences, Blacksburg, VA, USA*

*^5^ VTC School of Medicine, Pediatrics, Roanoke, VA, USA*

In the visual system, retinal axons convey visual information from the outside world to dozens of distinct retinorecipient brain regions. In rodents, two major areas that are densely innervated by this retinal input are the dorsal lateral geniculate nucleus (dLGN) and ventral lateral geniculate nucleus (vLGN), both of which reside in the thalamus. The dLGN is well-studied and known to be important for classical image-forming vision. The vLGN, on the other hand, is associated with non-image-forming vision and its neurochemistry, cytoarchitecture, and retinothalamic connectivity all remain unresolved, raising fundamental questions of its role within the visual system. Here, we sought to shed light on these important questions by studying the cellular landscape of the vLGN and map its connectivity with the retina. Using *in situ* hybridization, immuno-histochemistry, electrophysiology, and genetic reporter lines, we discovered at least six transcriptionally distinct subtypes of inhibitory neurons that are distributed into distinct adjacent sublaminae. Using trans-synaptic viral tracing and in vitro electrophysiology, we found that cells in each these sublaminae receive direct inputs from retina. Lastly, by genetically removing this visual input to the vLGN, we found that the organization of these sublaminae is dramatically disrupted, suggesting a crucial role for sensory input in the cyto-architectural maintenance of the vLGN. Taken together, these results not only identify novel subtypes of vLGN cells, but they also point to new means of organizing visual information into parallel pathways – by anatomically creating distinct sensory channels. This subtype-specific organization may be key to understanding how the vLGN receives, processes, and transmits light-derived signals in the subcortical visual system.

## OR-04 Oral Presentations 4

OR-04-01

**CANNABIDIOL INHIBITS MICROGLIAL INFLAMMATORY-TYPE REACTIONS THROUGH DECREASING GLUCOSE UPTAKE-DEPENDENT OXIDATIVE STRESS**

Maurício Dos-Santos-Pereira^1,2^, Francisco Guimarães^1^, Rita Raisman-Vozari^2^, Patrick Michel^2^, Elaine Del Bel^1^

*^1^ Universidade de São Paulo, Basic and Oral Biology, Ribeirão Preto, Brazil*

*^2^ Paris Brain Institute, Experimental Therapeutis of Parkinson's disease, Paris, France*

Here, we studied the anti-inflammatory potential of cannabidiol (CBD),the major non-psychoactlve component of cannabis. For that we used microglial cells in culture that were isolated from post-natal mouse brain through a procedure that relies on the adhesion preference of these cells to the polycation polyethyleneimine (Sepulveda-Díaz et aI, Glia, 2016; dos-Santos Pereira et aI, Glia, 2018). We established that CBD (1-10 µM) was highly efficient in reducing inflammatory-type responses triggered by the Toll-inflam-matory cytokines TNF-a and IL-1ß and that of glutamate,a noncyto-kine mediator of inflammation. Interestingly, CBD was also highly effective against other inflammatory signaling molecules,theTLR-2 agonist Pam3CSK-4 and the P2X7 agonist BzATP, indicating that CBD inhibitory effects were not restricted to a particular inflammatory pathway.The effects of CBD were predominantly receptor independent; they were only marginally blunted by SCH336, a selective antagonist/inverse agonist at CB2 receptors and insensitive to antagonists of CB1 receptors and PPAR-y. Additional experiments revealed that CBD had the capacity to restrain LPS-Induced Inflammatory events by interfering with a signaling cascade involving the ROS producing enzyme NADPH oxidase and subsequently NF-xB dependent signaling. Importantly, we noticed that NF-KB inhibition by either CBD (1,10 µM) or TPCA-1, an IKB kinase inhibitor counteracted the rise in glucose uptake that is observed after exposure of microglial cells to LPS, suggesting that CBD occluded pro inflammatory events by lowering glucose consumption. Comforting this view, CBD anti-inflammatory effects were mimicked by 2-deoxy-D-glucose (2-DG), a synthetic non-metabollzable glucose analog. CBD and 2-DG led to a reduction of glucose-derived NADPH, a requisite factor for ROS production by NADPH oxidase. Altogether, our findings suggest that CBD possesses potent anti-inflammatory effects towards microglial cells through an antioxidant mechanism that restrains glucose utilization and NADPH synthesis.Present data also further confirm that CBD may have a therapeutic interest in conditions where neuro-inflammatory processes are prominent.

OR-04-02

**MICRORNAS TARGETING NEURONAL NMDA RECEPTORS**

Sowmya Gunasekaran, R.V. Omkumar

*Rajiv Gandhi centre for Biotechnology, Molecular Neurobiology Division, Thiruvananthapuram, India*

N-Methyl-D-Aspartate Receptors (NMDARs) are glutamate-gated calcium channels, which play a major role in synaptic plasticity.

Dysregulation of NMDAR is associated with several neuro-degenerative and neuropsychiatric disorders. The NMDAR subunits GluN2A/ *Grin2A* and GluN2B/ *Grin2B* have a developmentally regulated expression profile (Cathala L et al., 2002, J.Neurosci.,20. p5899). We found that *Grin2B* expression is highest in days *in vitro* 7-9 (DIV 7-9) in rat hippocampal primary neuronal cultures and declines thereafter. *Grin2A* expression becomes significant by DIV 7-9 and the level is maintained subsequently.

Drugs that target NMDAR have major side effects and hence alternate subtle strategies are needed to indirectly target these receptors. The role of microRNAs (miRNAs) in neurological disorders is being widely explored (Wang W. et al, 2012, Learn. Mem.,19. p359). Our objective is to understand the mechanism of action of some miRNAs involved in NMDAR-mediated synaptic plasticity that are also differentially expressed in disease conditions. Some miRNAs that are altered in schizophrenia, Huntington disease and autism were predicted to interact with NMDAR subunits. Luciferase assays showed that miRNAs such as miR-223 and miR-129 interacted with the subunits of NMDAR. Expression levels of the target proteins in primary hippocampal neurons was downregulated after transfection of miRNA. To study regulation by these miRNAs *in vivo* we have established the MK-801 model and the MAM model of Schizophrenia. We have injected rats with MK-801 at post natal days 30-40 for five days followed by five day washout period. We injected MAM at gestational day 17 and the pups were weaned at P30. The animals underwent different behavior tests such as open field test, object recognition test and Morris water maze test. It was found that the treated animals have major cognitive impairment. Expression of some of the miRNA/s and their target proteins in these animals is under investigation. These studies may unravel novel mechanisms, which can be of therapeutic potential.

OR-04-03

**KETOGENIC DIET MODULATES MICROGLIAL MORPHOLOGY AT STEADY-STATE AND PROMOTES RESILIENCE TO REPEATED SOCIAL DEFEAT STRESS IN MICE**

Fernando González Ibáñez^1^, Kaushik Sharma^2^, Chloe McKee^3^, Katherine Picard^1^, Kanchan Bisht^2^, Haley A Vecchiarelli^3^, Nathalie Vernoux^1^, Jessica Deslauriers^1^, Marie-Ève Tremblay^3^

*^1^ CRCHU de Québec-Université Laval, Axe Neurosciences, Quebec, Canada*

*^2^ Center for Brain Immunology and Glia (BIG), University of Virginia, Department of Neuroscience, Charlottesville, USA*

*^3^ University of Victoria, Division of Medical Sciences, Victoria, Canada*

Ketogenic diet (KD; high-fat, low-carbohydrate) has emerged as a lifestyle change that may promote stress resilience. This study investigates the possible resilience-promoting properties of KD and aims to unravel underlying mechanisms by focusing on microglia specifically, the resident immune cells of the brain. Using 2 month-old adult male C57BL/6 mice, we studied the effects of KD *versus* normal diet (ND) exposure for 4 weeks. The consequences of chronic stress under KD *versus* ND were investigated by comparing non-stressed controls with animals undergoing 10 days of repeated social defeat (RSD). After RSD, mice underwent a social interaction test (SIT) to classify them as resilient or susceptible to stress. We are focusing on the ventral hippocampus CA1 *stratum radiatum*, previously shown to be affected by chronic stress. Our results show that after RSD, ketogenic diet increased the proportion of resilient animals, 57.14% of KD mice (n=28) *vs* 36.36% of ND (n=22). We studied the effect that ketogenic diet on its own might have on microglia. Using TMEM119/IBA1 double staining we have observed that KD does not affect microglia number and distribution. Nevertheless, the microglia of KD animals show increased soma and arborization area. This observation is very important given that changes in microglia morphology suggest changes in their function, suggesting. Further analyses of stress susceptible *versus* resilient animals, together with ultrastructural studies, will expand our understanding of KD on microglial function. Ongoing analysis using scanning electron microscopy will allow us to perform ultrastructural characterization of microglial function, their interactions with other cell types, synaptic elements, as well as provide valuable intracellular information regarding their phagocytic activity and markers of cellular stress.

OR-04-04

**COMPREHENSIVE ANALYSIS OF THE MICROGLIA TRANSCRIPTOME ACROSS THE HUMAN LIFESPAN USING SINGLE CELL RNA SEQUENCING**

Moein Yaqubi^1^, Jack Antel^1,2^, Luke Healy^1,2^

*^1^ McGill University, IPN, Montreal, Canada*

*^2^ Montreal Neurological Institute, Neuroimmunology Unit, Montreal, Canada*

Microglia as the main resident immune cells of the central nervous system (CNS) have critical, underappreciated roles in development and in the maintenance of brain homeostasis. Microglia also become highly reactive throughout the course of all neurological disease including multiple sclerosis. To gain a clear insight into the role of microglia in CNS pathology we first need to understand the molecular mechanisms underlying development of these cells. Further-more, it has been shown that animal model organisms consistently fail to mimic human physiological conditions. In the present study, we performed whole, single-cell RNA sequencing on 13,568 microglia isolated from surgical samples of second trimester fetal, pediatric (18 months to 2 years old), adolescent (10 to 14 years old) and adult (40 to 62 years old) brains. Using Seurat (v3) package to analyze the data we showed there are multiple subtypes of microglia during human development contributing to a continuum genes expression. We showed fetal microglia have a distinct transcriptomic expression profile compared to all post-natal samples. Based on the transcriptional signatures we predict fetal cells to have a higher phagocytic capacity than microglia from other ages while postnatal microglia have a greater proinflammatory gene signature and this increases with age. Moreover, we revealed the adolescent derived microglia were transcriptionally distinct from pediatric and adult cells, and they exhibited the most metabolically active transcriptomic profile. Finally, we correlated our findings about human microglia with mouse microglia datasets from the literature and observe that in spite of high correlation between human and mouse, microglia samples cluster according to their species and not according to developmental age. To understand the diverse function of human microglia during disease we must first gain insight into the gene expression programs that underlie each stage of normal development. This study is a valuable resource to explore transcriptional changes of human microglia during development at the single cell level.

OR-04-05

**IF YOU GIVE A FLY A COMPLEMENT: INVESTIGATING THE GLIAL RESPONSE TO CNS INSULT AND INJURY**

Matthew Boisvert, Mary Logan

*Oregon Health and Science University, Neurology, Portland, USA*

The complement system is a highly conserved signaling cascade that targets pathogens and apoptotic cells for removal by systemic immune cells. Recent work reveals that complement factors (e.g., C1q, C3, C4b) play important roles in glial-dependent synaptic refinement and are characteristically expressed in glia during central nervous system injury. Reactive glia mediate the brain's response and recovery after a wide variety of insults and injury, and glia-derived complement components consistently increase in everything from aging and neurodegeneration to traumatic brain injury and stroke. Complement inhibition attenuates some glial reactivity and neuro-degeneration-related synaptic and behavioral deficits, but the role of this pathway in the glial injury response is largely opaque. To further explore how the brain mediates damage, this work establishes *Drosophila melanogaster* as a model to examine complement-like pathways in reactive glia. Flies display a robust glial injury response, with a short generation time and bounty of genetic tools making them a vital paradigm to examine the basic mechanisms of glial reactivity. My analyses indicate that fly thioester-containing protein (TEP) family members Tep2 and Tep4 share ∼40% protein sequence similarity with the secreted mammalian C3 and C4b complement factors, with conservation in important functional domains. This work reveals that Tep2 and 4 are expressed by glial cells and are elevated in multiple paradigms of central nervous system injury. Furthermore, Tep2/4 localize around the site of axonal injury, and glial Tep knockdown inhibits axonal degeneration. I hypothesize that Drosophila Teps play mammalian complement-like roles in the fly brain and are involved in glial injury response. Overall, this project investigates glial complement-like functionality in the brain, parsing apart the roles these Teps play in reactive glia and injury, focusing on their impact and interactors.

## Poster sessions

## Poster Session A

A01

**CONDITIONAL DELETION OF THE ANION CHANNEL SUBUNIT LRRC8A IN THE MOUSE BRAIN LEADS TO SPONTANEOUS SEIZURES AND MORTALITY**

Julia Nalwalk^1^, Corinne Wilson^1^, Preeti Dohare^1^, Shaina Orbeta^1^, Rajan Sah^2^, Yunfei Huang^1^, Russell Ferland^3^, Alexander Mongin^1^

*^1^ Albany Medical College, Department of Neuroscience and Experimental Therapeutics, Albany, NY, USA*

*^2^ Washington University School of Medicine, Department of Internal Medicine, St. Louis, MO, USA*

*^3^ University of New England, Department of Biomedical Sciences, Biddefore, ME, USA*

Volume-regulated anion channels (VRACs) are ubiquitous, swelling-activated chloride channels, which regulate cell volume and participate in other cellular functions. Physiological roles for VRACs in the brain remain poorly understood. We and others reported 100% lethality and behavioral seizures in mice with brain-specific deletion of the essential VRAC subunit LRRC8A. Here, we used EEG and video recordings to explore frequency and characteristics of seizures and establish their potential link to mortality LRRC8A-null animals. *Lrrc8a* ^flox/flox^ mice were bred with *Nestin*-Cre+ animals to produce brain-wide LRRC8A knockouts (KO), heterozygotes (Het), and *Lrrc8a* ^flox/+^ and *Lrrc8a* ^flox/flox^ controls. Western blotting showed complete loss of LRRC8A protein in KO. Cortical EEG electrodes were implanted at 5-6 weeks, and individually housed animals were recorded until the age of 10-12 weeks (controls) or time of death (KO). In the initial study, we found 100% mortality in KO during early to late adolescence (5-9 weeks) with frequent behavioral seizures. In the present longitudinal EEG work, all KO mice showed moderate-to-severe EEG and behavioral seizure activity (n=6). In striking contrast, no EEG abnormalities or behavioral seizures were present in fl/+, fl/fl, or Het littermates (n=6/group). Unlike other genotypes, KO did not build nests. Four out of six KO mice died, with three of them having seizure preceding the death, one animal died without a coinciding EEG seizure, and two animals remain under observation. In conclusion, we determined that all KO animals with brain-wide deletion of the essential VRAC subunit LRRC8A develop spontaneous seizures. These findings point to an unexpected and previously unknown critical role for LRRC8A anion channels in the regulation of brain excitability.

Supported by NIH R01 NS111943

A02

**CORTICAL NEURON DENDRITE DEVELOPMENT REQUIRES THE LVS MOTIF OF PLEXINA4 RECEPTOR, AND TRANSCRIPTIONAL AND TRANSLATIONAL REGULATION**

Oday Abushalbaq, Tracy S. Tran

*Rutgers University, Department of Biological Sciences, Newark, New Jersey, USA*

Neuronal circuit development is a complex process consisting of precise steps in the directional growth and maturation of axons and dendrites to synapse formation and elimination. The development of axons and dendrites, in particular, is largely dependent on extra-cellular guidance cues. The large family of Semaphorin molecular cues is known to regulate axon guidance, dendrite elaboration and synapse elimination in the developing mammalian nervous system. In particular, the class 3 secreted Semaphorin 3A (Sema3A), through its receptor complex Neuropilin-1 (Nrp1)/PlexinA4 (PlxnA4), has been shown to induce dendritic elaboration in cortical neurons *in vitro* and *in vivo*. Previously, we showed that the KRK amino acid motif in the PlxnA4 receptor is required for cortical dendritic elaboration through the interaction with the RhoGEF FARP2 and activation of Rac1. However, it is not known whether other signaling motifs in the PlxnA4 receptor could also mediate dendrite development. Here, we show that the LVS motif, located in the Rho GTPase binding domain of PlxnA4, is required for Sema3A-mediated dendritic elaboration in cortical neurons. Moreover, our findings show for the first time that gene transcription and protein translation are required in Sema3A-induced dendritic elaboration of cortical neurons *in vitro* during the initial stages of dendritic elaboration, but not later stages. Next, we will identify novel downstream gene targets of Sema3A-induced dendrite elaboration by using RNA sequencing in our established primary cortical neuron system and *in vivo* using specific genetically modified mouse lines. Taken together, our study provides novel insights to the intracellular mechanisms underlying Sema3A-mediated dendritic morphogenesis in cortical neuron development, and dissects the multifunctional roles of the PlxnA4 receptor in the proper formation of neuronal connections.

A03

**BONE MORPHOGENETIC PROTEIN 4 AND LUTEOLIN TO TARGET AUTOPHAGY IN GLIOBLASTOMA**

Nadia Al-Sammarraie, Swapan K. Ray

*University of South Carolina School of Medicine, Department of Pathology Microbiology and Immunology, Columbia SC, USA*

Glioblastoma is a deadly malignant tumor that originates from abnormal astrocytes in the brain and, less commonly, in the spinal cord. It is characterized by rapid recurrence after surgery, resistance to treatment, and high mortality rate. The location of the tumor, the heterogenous nature of the disease, and the accumulation of multiple mutations in tumor suppressors contribute to therapy resistance and recurrence. Autophagy is a self-eating and recycling process of the damaged cellular components and organelles, providing a survival mechanism to the aggressive tumors that grow in stressful micro-environments such as nutrient deficiency and hypoxia. Targeting autophagy in glioblastoma is now widely accepted to be a promising therapeutic strategy. Recombinant proteins, dietary supplements, or natural compounds have shown anti-tumor effects in glioblastoma and other cancers. However, glioblastoma rapidly develops resistance to a single therapy. A synergistic combination therapy could provide better growth inhibitory effects on glioblastoma, showing the efficacy at lower doses and causing less damages to the adjacent normal cells. Bone morphogenetic protein 4 (BMP4) is a member of the transforming growth factor beta (TGFβ) family of multi-function proteins. BMP4 plays a critical role during nervous system development and maturation and it is found to be down regulated in glio-blastoma leading to poor outcomes, while treating glioblastoma cells with recombinant BMP4 has shown promising antitumor actions. Luteolin (LTL) is a flavonoid found in several plants and it has antioxidant, neuroprotection, anti-inflammatory, and tumor inhibitory properties. We aimed to investigate the synergistic anti-tumor efficacy of BMP4 and LTL in two human glioblastoma cell lines (U87MG with p53 wild-type and U251 with p53 mutant-type), targeting autophagy flux in nutrient-deficient growth medium. Our study designed to show that targeting autophagy with synergistic combination of BMP4 and LTL caused morphological and molecular changes (Beclin-1, LC3 II, and p62) leading to disparate levels of growth inhibition due to dissimilar status of the tumor suppressor p53 in the glioblastoma cell lines.

A04

**NON-CANONICAL TARGETS OF HIF1A IMPAIR OLIGODENDROCYTE PROGENITOR CELL FUNCTION**

Kevin Allan, Lucille Hu, Marissa Scavuzzo, Andrew Morton, Artur Gevorgyan, Erin Cohn, Benjamin Clayton, Ilya Bederman, Stevephen Hung, Cynthia Bartels, Mayur Madhavan, Paul Tesar

*Case Western Reserve University, Genetics and Genome Sciences, Cleveland, USA*

All mammalian cells sense and respond to insufficient oxygen, or hypoxia, through the activity of hypoxia-inducible factors (HIFs), an evolutionarily conserved family of transcriptional regulators that promote oxygen-independent energy metabolism and angiogenesis. While HIF activation is transiently protective for all cells, prolonged HIF activity drives distinct pathological responses in different tissues and exerts a powerful influence over fate decisions in a multitude of progenitor and stem cell types. How HIFs achieve this pleiotropic effect is largely unknown. In the central nervous system (CNS), prolonged HIF accumulation blocks myelin development as seen in stroke, premature birth, and subsets of cerebral palsy. Here, we demonstrate that HIF1a activates unique non-canonical targets to impair the function of oligodendrocyte progenitor cells (OPCs) to generate oligodendrocytes, a glial subtype of the CNS responsible for myelin formation. Beyond the canonical hypoxia response targets shared between all cell types, HIF1a activated a unique set of gene targets in OPCs through interaction with the OPC-specific transcription factor OLIG2. These non-canonical targets, including *Ascl2* and *Dlx3*, when ectopically expressed were sufficient to block oligo-dendrocyte development through suppression of the key oligodendro-cyte regulator *Sox10*. Chemical screening of 1,800 bioactive compounds revealed that inhibition of MEK/ERK signaling overcame the HIF1a-mediated block in oligodendrocyte generation by restoring *Sox10* expression without impacting canonical HIF1a activity. MEK/ ERK inhibition also drove oligodendrocyte formation in hypoxic regions of human oligocortical spheroids. Collectively, this work defines the mechanism by which HIF1a impairs oligodendrocyte formation. More broadly, we establish that cell-type-specific HIF1a targets, independent of the canonical hypoxia response, can developmentally or pathologically perturb cell function in response to low oxygen.

A05

**ABSENCE OF P75NTR PROMOTES DEVELOPMENT OF PROGENITORS ALONG THE OLIGODENDROCYTE LINEAGE IN THE POSTNATAL RAT SUBVENTRICULAR ZONE**

Subhashini Joshi, Wilma Friedman

*Rutgers University-Newark, Department of Biological Sciences, Newark, USA*

During neonatal and early postnatal brain development, progenitors from the Subventricular Zone (SVZ) can give rise to neurons and glia that contribute to the development of the cortex, corpus callosum and olfactory bulb. Independent studies have shown that neurotrophins, a family of growth factors, can influence many aspects of neuronal and glial stem cell development including proliferation, differentiation, migration and quiescence. We found that the pan neurotrophin receptor, p75NTR, is expressed by a subpopulation of nestin-expressing progenitor cells in the dorsolateral SVZ (dSVZ) throughout the postnatal development of rats from ages P1-P21. We also observed increased expression of p75NTR between the ages P7-P10, which coincides with the period of gliogenesis during development, however the role of p75NTR in the postnatal rat dSVZ during this period remains unexplored. We aim to understand the role of p75NTR in influencing the progenitor population in the dSVZ by characterizing the p75NTR expressing cells *in vivo* and isolating and comparing the proliferation and differentiation capabilities of the p75NTR-expressing cells with cells of the dSVZ that lack p75NTR, and dSVZ cells isolated from a p75NTR KO rat. We find that the absence of p75NTR reduces the proliferative capacity of neuro-spheres cultured from the SVZ and promotes differentiation of progenitor cells along the oligodendrocyte lineage.

A06

**REGULATION OF SYSTEM XC-EXPRESSION IN CORTICAL ASTROCYTES BY ARSENITE**

Audrey Wood, Caroline Baggeroer, Regina Catague, Sandra Hewett

*Syracuse University, Biology/Neuroscience, Syracuse, USA*

Millions of people in the United States are exposed chronically to inorganic arsenic, mostly in the form of arsenite, through drinking water. Because arsenite can be transported by phosphate and glucose permeases, it can cross the blood-brain barrier and enter astrocytes of the central nervous system (CNS), where is has been shown to have negative effects. Numerous studies demonstrate that arsenite generates oxidative stress in various cell types, though whether this occurs in astrocytes has yet to be definitively determined. This may be because astrocytes contain a large and essential reservoir of the antioxidant glutathione (GSH). Increased production of oxidized GSH occurs upon its consumption to reduced GSSG, a process dependent on the activity of system x_c_^−^, a cystine/glutamate antiporter that imports the rate-limiting substrate, cyst(e)ine. Our lab previously demonstrated that exposure of astrocytes to arsenite increases steady-state mRNA expression of the system x_c_^−^ substrate-specific light chain, xCT, through a transcriptional mechanism. Further, astrocytic arsenite exposure increased both astrocyte-derived GSH and GSSG. Thus, we posit that arsenite enhances astrocytic xCT expression through its production of ROS. To test this, primary cortical astrocyte cultures were treated for four hours with sodium arsenite (15 µM) alone or with sodium arsenite (15 µM) plus the antioxidant idebenone (30 µM), after which xCT mRNA expression was determined via qPCR ΔΔCt analysis. The rapid redox cycling drug menadione (30 µM)±idebenone (30 µM) was used as positive control. Data thus far demonstrate that treatment with arsenite and menadione increases astrocytic xCT mRNA production, and that idebenone treatment inhibits this increase. These data are consistent with the idea that arsenite produces an increase in ROS in astrocytes that regulates the expression of system x_c_^−^ in order to enhance antioxidant production. Supported by NINDS R01 NS051445.

A07

**SCREENING AND IDENTIFICATION OF GLYCOMIMETIC INHIBITORS OF IMMUNE CELL INFLAMMATION IN ALZHEIMER'S DISEASE**

Kapur Dhami, Ganesh Shrestha, Saroj Kafle, Alexei Demcheko, Christopher Spilling, Michael R Nichols

*University of Missouri St. Louis, Department of Chemistry and Biochemistry, St. Louis, USA*

The accumulation of extracellular plaques in the brain is a signature feature of Alzheimer's disease (AD). The plaques, primarily composed of aggregated amyloid-β peptide (Aβ), create an inflammatory environment involving immune cells such as microglia. Activation of pattern recognition receptors (PRRs) in microglia by oligomeric Aβ aggregates, such as protofibrils, triggers an innate immune response leading to the release of inflammatory mediators that contribute to disease progression and severity. Numerous studies have demonstrated the involvement of lipopolysaccharide (LPS) receptors cluster differentiation 14 (CD14) and Toll-like receptor 4 (TLR4) in Aβ mediated neuroinflammation. LPS is a toxic outer-leaflet component of gram-negative bacteria. We hypothesized that compounds with potential LPS inhibitory effect can be equally effective for the inhibition of Aβ mediated neuroinflammation. Previous work from our laboratory identified the monosaccharide, Lipid A analog AM-12 as a potent antagonist of LPS-induced inflammation. AM-12 possesses a glucopyranoside core, lipid chains connected by ether linkages, and an Fmoc-protected amino acid to add ionic character. A new series of AM-12 derivatives was tested for LPS antagonistic activity in human THP1 macrophages. The compounds were effective inhibitors and did not display toxicity or agonistic activity. This new class of compounds contained changes at the amino acid carboxy terminus and the functional group in the glucopyranoside ring. The presence of a hydrogen acceptor group at the 4-position of the ring improved the antagonistic activity of the compounds. Furthermore, the original AM-12 compound inhibited Aβ42 protofibril-mediated inflammation in THP macrophages. Another class of compounds comprised of anomeric lactones with varying length of the nonpolar ether-linked side chain at the 2- and 3- position also inhibited LPS induced inflammation in a concentration dependent manner. However, the chain length also played a role in causing toxicity to the THP1 macrophages. It is hoped that the continuing studies will produce sugar-based compounds that effectively block LPS and Aβ-triggered inflammation.

A08

**PHARMACOGENETIC INHIBITION OF AFFERENT EXCITABILITY ALLEVIATES VEGF-INDUCED VISCERAL HYPERSENSITIVITY IN A MOUSE MODEL OF UCPPS**

Alison Xiaoqiao Xie, Anna Malykhina

*University of Colorado, Denver, Surgery, Aurora, USA*

Patients with urological chronic pelvic pain syndrome (UCPPS) experience chronic pelvic pain (CPP) and lower urinary tract symptoms (LUTS). The UCPPS symptoms are closely associated with nociceptive sensitization in the nervous system, which underlies visceral allodynia and hyperalgesia. Previous studies suggested that afferent hypersensitivity in bladder-projecting sensory neurons plays an important role in the generation and the maintenance of UCPPS symptoms, especially bladder pain and urinary frequency. In this study, we tested the hypothesis that visceral hypersensitivity and the symptoms of UCPPS could alleviated by pharmacogenetic inhibition of sensory neuronal excitability in a mouse model of UCPPS. Vascular endothelial growth factor (VEGF) level is correlated with CPP in UCPPS patients, and in animal models of bladder overactivity. Intravesical instillation of VEGF_165_ induced nociceptive sensitization and bladder nerve remodeling in both male and female C57BL6/J mice. Bladder overactivity and pain were evaluated by *in vivo* cystometry and Von Frey assay, respectively. To directly manipulate afferent sensitivity, *Designer Receptors Exclusively Activated by Designer Drugs* (DREADDs) were expressed in bladder-projecting sensory neurons via targeted adeno-associated viral vector (AAV) injections. Transgenic mice expressing Cre-recombinase and fluorescence reporters in bladder sensory neurons and afferent nerves were used to guide cellular expression of DREADDs as well as to reveal the potential nerve remodeling via neuroimaging. We found that VEGF_165_ induced sensory nerve remodeling in the urinary bladder, bladder overactivity, and visceral mechanical allodynia and hyperalgesia. The VEGF_165_-induced symptoms were likely due to VEGF receptor signaling-mediated upregulation of nociceptors in bladder sensory nerves. Pharmacogenetic inhibition of bladder-projecting sensory neurons using DREADDs significantly attenuated VEGF_165_-induced visceral mechanical allodynia and hyperalgesia and alleviated the symptoms of bladder overactivity. Our data suggests a functional role for VEGF signaling in peripheral sensory neurons in a murine model of UCPPS. Further investigation of the molecular targets in VEGF signaling pathways in bladder sensory neurons will shed lights on alternative treatments for UCPPS.

A09

**CLASS IIA HISTONE DEACETYLASES IN NEUROINFLAMMATION FOLLOWING TRAUMATIC BRAIN INJURY**

David Crockett, Victoria L. DiBona, Emily C. Kelly-Castro, Mikayla Voglewede, Anusha Patil, Kinjal Shah, Kavya Mehndiratta, Ishan R. Podar, Huaye Zhang

*Rutgers-Robert Wood Johnson Medical School, Neuroscience and Cell Biology, Piscataway, USA*

Each year millions of Americans will suffer either a traumatic injury to the brain or to the spinal cord. At present, there is no effective cure. It is clear that an effective treatment will have to be multifactored focusing on a wide range of issues. One major factor that needs to be addressed is the ongoing neuroinflammation triggered by the injury, evidence of which can be detected months to years following the initial insult. Prolonged inflammation can lead to continued loss of neurons and oligodendrocytes and consequently loss of function. A major player in the inflammatory response is the microglia, the brain's resident immune cells. Microglia are highly ramified at “rest” but change into rounded phagocytic cells following injury. The mechanism associated with this activation and morphological change is not completely understood. We have recently demonstrated that the polarity protein Par1b/MARK2 (DiBona et al 2019) may be instrumental in the inflammatory response. Par1b/MARK2 is a serine/thionine kinase and is a member of Par1/MARK (microtubule affinity regulating kinase) family of polarity proteins. Par1/MARK proteins are noted for their roles in regulating a number of cellular processes within the brain including neuronal migration, dendritic development and spine formation. The loss of Par1b/MARK2 facilitates the microglial inflammatory responses *in vitro* and *in vivo* following traumatic brain injury (TBI) in mice. In order to better understand the mechanisms of injury-induced inflammation, we are examining downstream effectors of Par1b/ MARK2. One candidate is the class IIa histone deacetylases (HDAC4, HDAC5, HDAC7 and HDAC9). Par1b/MARK2 has been shown to regulate cellular localization, and function of with class IIa HDACs through N-terminal phosphorylation (Dequiedt et al 2006). HDAC7 activity plays and important role in macrophage immune responses (Shakespeare et al 2013 and Gupta et al 2020). We have found intense immunoreactivity of microglia at the focal injury site in the brains of mice subjected to TBI. Interestingly, reactive astro-cytes response similarly show strong immunoreactivity for HDAC7.

A10

**IN VIVO STUDY OF TARGETING THE INFLAMMATORY TGF-Β /NON-SMAD SIGNALING PATHWAY IN THE EPILEPTOGENIC MODEL OF MICE**

Warda Ain-uddin, Maha Shahid, Uzair Nisar, Farzana Shaheen, M. Iqbal Choudhary, Atta -ur-Rahman, Iqra Mukhtar, Shabana Usman Simjee

*International Center of Chemical and Biological Sciences (University of Karachi), H.E.J Research Institute of Chemistry, Karachi, Pakistan*

Objective: Despite extensive research on epileptogenesis, current medications only provide symptomatic control of seizures. Thus, there is high demand in the investigation of new mechanisms and approaches for the development of treatments for 30% of epilepsy cases that prove drug resistant. Inflammation, neural loss, plasticity, mossy fibre sprouting and blood–brain barrier dysfunction are the most common causes of epileptogenesis. Increasing evidence supports that transforming growth factor (TGF-β) /non-SMAD pathway is of utmost importance in neuroinflammation mediated epileptogenesis. Therefore, exploring TGF-β /non-SMAD pathway could provide an interesting opportunity to discover and validate targets for novel therapeutics for controlling pharmacoresistant epilepsies. Methodology: Male Balb/c mice were categorized into 5 groups i.e., normal-control, Pentylenetetrazole-control, drug control (diazepam and valproic acid) and test group i.e., Isoxylitone (E/Z-2- propanone-1,3,5,5-trimethyl-2-cyclohexen-1-ylidine) abbreviated as (ISOX). Kindling was induced by giving sub-convulsive dose of pentylenetetrazole (PTZ, 40 mg/kg) every alternate day until seizure score 5 develops in the PTZ-control group. Treatments were given to respective groups 30 min prior to PTZ dose. When animals acquired consistent score 5 for at least 3days, experiments were terminated and brain samples i.e., cortex and hippocampus were isolated for further gene expression studies. Result: The experimental findings revealed that ISOX (30 mg/kg) not only significantly suppressed the PTZ-induced seizures but also halted the epileptogenesis by altering the non-SMAD associated TGF-β genes. ISOX pre-treatment significantly upregulated the RhoA, ROCK2 and AKT expressions and downregulated the ROCK1, MAPK14, and NFkB expressions as compared to PTZ-control group in hippocampus region, suggesting the disease modifying effect of ISOX and other treatments in epileptogenesis. Conclusion: Our findings suggest that non-SMAD/ TGF-β signaling pathway act as critical target in epilepsy, and also rationalizes the ISOX as a promising newer neuroprotective, anti-inflammatory, and disease-modifying agent in forestalling the epileptogenesis

A12

**CALPAIN ISOFORMS DIFFERENTIALLY REGULATE INFLAMMATORY MEDIATORS IN RODENTS AND MS PATIENTS**

Narendra Banik^1^, Mollie Capone^2^, Nicole Trager^1^, Denise Matzelle^1^, Azim Hossain^2^, Rachel Polcyn^2^, Donald Shields^1^, Azizul Haque^2^

*^1^ MUSC, Neurosurgery, Charleston, USA*

*^2^ MUSC, Microbiology and Immunology, Charleston, USA*

*^3^ VA Medical Center, Neurology, Charleston, USA*

Multiple sclerosis (MS) is a neurodegenerative demyelinating disorder that impairs neuronal function in the brain and spinal cord. The incidence of MS has been increasing worldwide over the past decades, but no drug is currently available to provide a definitive cure. Studies in experimental autoimmune encephalomyelitis (EAE), an animal model for MS, have provided convincing evidence that T cells specific for self-antigens mediate pathology in this disease. Interactions between the major histocompatibility complex II (containing antigenic peptides) and the T cell receptor activate CD4+ T cells that perpetuate EAE and MS. Increased calcium activated neutral proteinase (calpain) activity has been observed in MS and EAE; in addition, calpain is implicated in the activation of T cells (Th1/Th17), degradation of myelin proteins, and T cell chemotaxis. Inhibiting calpain has been shown to reduce these activities. While calpain is activated in brain and spinal cord of MS patients, the precise involvement of the two calpain isoforms, calpain-1 and calpain- 2, remains poorly defined. Studies in our lab suggest that calpain-2 expression is significantly increased in brain and spinal cord postmortem tissues of MS patients as compared to calpain-1. Current MS therapies (with various side effects) target immunomodulation, but have only modest effects in attenuating the immunologic and neuro-degenerative components of the disease. Since axon/myelin degeneration is implicated in the resulting disability in progressive cases of MS, development of novel therapeutic strategies directed toward both inflammation and neurodegenerative processes is imperative. Our study demonstrates that elevated levels of calpain expression/ activity and reactive oxygen species are detected in human microglia cells; these findings are attenuated following calpain inhibitor treatment. Thus, calpain inhibitors may be employed as future therapeutic agents for treating MS symptoms, and distinct calpain isoforms may be useful as biomarkers for MS. Supported in part by grants from the SCIRF and the Veterans Administration.

A13

**SEASONAL CHANGES IN ADK IN TANYCYTES ARE ASSOCIATED WITH SEASONAL CHANGES IN PURINERGIC SIGNALING THAT UNDERLIE HIBERNATION**

Carla Frare, Sarah Rice, Kelly Drew

*University of Alaska Fairbanks, Chemistry and Biochemistry, Fairbanks, USA*

Hibernation is a seasonal strategy to conserve energy, characterized by modified thermoregulation, an increase in sleep pressure and drastic metabolic changes. Glial cells such as astrocytes and tanycytes are the brain metabolic sensors but it remains unknown whether they contribute to seasonal expression of hibernation. The onset of hibernation is controlled by an undefined endogenous circannual rhythm in which adenosine plays a role through the activation of the adenosine A_1_ receptor (A_1_AR). Seasonal changes in brain tissue levels of adenosine may contribute to an increase in A_1_AR agonist sensitivity leading to the onset of hibernation. The primary regulator of extracellular adenosine concentration is adeno-sine kinase (ADK), which is located in astrocytes. Moreover, tanycytes the other glial cells of interest are known to regulate seasonal change in phenotypes in other species. We asked if a seasonal change in ADK in hypothalamic astrocytes or periventricular tanycytes was associated with seasonal A_1_AR agonist sensitivity. We collected Arctic ground squirrel (*Urocitellus parryii*) brain tissue in summer, fall and winter after intracardiac perfusion with 4% paraformaldehyde for immunohistochemical analysis. We used a mouse anti-vimentin antibody (1:1000, Millipore, MA Cat# MAB3400, RRID:AB_94843 ) to identify tanycytes and a rabbit anti-ADK to identify astrocytes (ADK, 1:4000, Bethyl Laboratories, TX Cat# A304-280A, RRID:AB_2620476 ). We performed immuno-histochemistry for seasonal groups in parallel and quantified ADK fluoresence intensity. We compared tanycyte morphology and ADK expression within two brain regions involved in energy homeostasis, the hypothalamus and the area postrema. We found seasonal changes in tanycyte morphology in the hypothalamus. We also observed for the first time the presence of ADK in tanycyte cell bodies, and we observed a seasonal change in ADK expression in tanycytes but not in astrocytes. Although still speculative, our findings contribute to a model whereby adenosine kinase in tanycytes regulates seasonal changes in extracellular concentration of adenosine underling the seasonal expression of hibernation.

A14

**ASTROCYTE-SELECTIVE KNOCKDOWN OF ADENOSINE KINASE AMELIORATES COMBINED RADIATION-AND CHEMOTHERAPY-INDUCED COGNITIVE DYSFUNCTION**

Munjal Acharya^1^, Robert Krattli^1^, Leila Alikhani^1^, Al Anoud Baddour^1^, Janet Baulch^1^, Detlev Boison^2^

*^1^ University of California Irvine, Radiation Oncology, Irvine, USA*

*^2^ Rutgers University, Neurosurgery, Piscataway, USA*

The combined cranial radiation- (RT) and chemo-therapy (temozolomide, TMZ) are the standard of care for brain cancer treatment. Unfortunately, clinical RT-TMZ treatment has been linked with debilitating cognitive decline. This is particularly a pressing problem for the grade II/III glioblastoma and childhood brain cancer survivors who often live long post-treatment and endure persistent RT-induced cognitive deficits (RICD) with no clinical recourse. Our data showed that acute RICD was accompanied by elevated astrogliosis. Among other gliotransmitters, astrocytes regulate the synaptic adenosine pools, an endogenous neuroprotectant, and modulator of cognition, via metabolic clearance through the cytosolic adenosine kinase (ADK). In the adult brain, astrocytes are the predominant source of ADK. We have shown the prevention of RICD and astrogliosis by pharmacologic inhibition of ADK. To elucidate the astrocyte-specific mechanism of adenosine modulation, we utilized the rAAV-based Adk-miRNA vector (Adk-KD) to study its impact on the brain exposed to clinically relevant RT-TMZ treatment. Under the GfaABC1D promoter, the rAAV vector was designed to knock down astrocytic ADK and provide a powerful tool to mechanistically understand the impact of RT-TMZ. Adult WT mice receiving RT-TMZ (fractionated RT, 8.67 Gy x3 doses+adjuvant TMZ, 25 mg/kg) and intra-hippocampal injections of the scrambled vector showed a significant decline in the memory consolidation and cognitive function tasks (novel object or place recognition, fear extinction memory) 6 weeks post-RT-TMZ. Irradiated brains showed elevated ADK immunoreactivity linked with hypertrophic astrocytes. Conversely, mice receiving the Adk-KD vector 1-week post-RT-TMZ showed significantly improved cognitive function 6-weeks post-exposure. The Adk-KD vector selectively transduced astrocytes and knocked down (>80%) ADK throughout the brain, attenuated RT-TMZ-induced astrogliosis, and increases in A1, and A2a receptor expression. The astrocyte-selective gene silencing approach provides direct evidence supporting our hypothesis that cranial RT-TMZ disrupts adenosine metabolism, and interventions targeted to reduce astrocytic ADK may prove beneficial to ameliorate brain cancer therapy-induced cognitive dysfunction.

A15

**LIFESPAN EXTENSION AND MEMORY PRESERVATION IN AGED SYSTEM XC- - DEFICIENT MICE IS ASSOCIATED WITH ALTERED HIPPOCAMPAL METABOLOME**

Gamze Ates^1^, Lise Verbruggen^1^, Hideyo Sato^2^, Eduard Bentea^1^, Ann Massie^1^

*^1^ Vrije Universiteit Brussel, Neuro-Aging & Viro-Immunotherapy, Brussels, Belgium*

*^2^ Niigata University, Medical Technology, Niigata, Japan*

Life expectancy keeps increasing worldwide, yet the current average healthy life years is predicted to be around 64. One of the most impactful consequences of aging is a deterioration of cognitive functions that rely on proper hippocampal function. Investigating ways to maintain cognition during aging has therefore become a public health priority.

We show that mice lacking xCT (xCT^−/-^), the functional subunit of the cystine/glutamate antiporter system x^c-^, live longer than their wildtype littermates (xCT^+/+^). Moreover, at 18 months, xCT^−/-^ mice have preserved cognitive functions when analyzing the performance of both genotypes in the Barnes maze task, contrary to xCT^+/+^ mice. To investigate possible mechanisms through which xCT deletion retains memory function, we performed untargeted metabolomics analysis on hippocampus of adult (3m old) and aged (18m old) xCT^+/+^ and xCT^−/-^ mice.

The metabolic profile of adult xCT^+/+^ and xCT^−/-^ mice shows limited differences, yet random forest classification analysis reveals a distinct metabolome when mice age in the absence of xCT. The key differences are observed in several groups of glycerolipids, sphingo-lipids and in one-carbon metabolism.

Non-enzymatic post-translational modifications such as glycation and carbamylation, are known to be increased with aging and negatively affect cellular functions. In our study, levels of carbo-xymethyllysine, a marker of advanced glycation end products, are significantly lower in both adult and aged xCT^−/-^ mice, compared to age-matched xCT^+/+^ controls. Homocitrulline, a marker of carbamylation, shows an overall increase in the hippocampus of aged compared to adult mice and this effect is most prominent in xCT^+/+^ mice. Furthermore, the neurotransmitter acetylcholine -involved in learning and memory - and the sulfate ester of the hormone dehydroepiandrosterone (DHEA-S) -having anti-inflammatory and neurotrophic capacities-are significantly increased with aging; an effect that is driven by the xCT^−/-^ mice. Overall, these results show several metabolic changes in the hippocampus of aged xCT^−/-^ mice that could explain preservation of hippocampal function.

A16

**IF YOU GIVE A FLY A COMPLEMENT: INVESTIGATING THE GLIAL RESPONSE TO CNS INSULT AND INJURY**

Matthew Boisvert, Mary Logan

*Oregon Health and Science University, Neurology, Portland, USA*

The complement system is a highly conserved signaling cascade that targets pathogens and apoptotic cells for removal by systemic immune cells. Recent work reveals that complement factors (e.g., C1q, C3, C4b) play important roles in glial-dependent synaptic refinement and are characteristically expressed in glia during central nervous system injury. Reactive glia mediate the brain's response and recovery after a wide variety of insults and injury, and glia-derived complement components consistently increase in everything from aging and neurodegeneration to traumatic brain injury and stroke. Complement inhibition attenuates some glial reactivity and neuro-degeneration-related synaptic and behavioral deficits, but the role of this pathway in the glial injury response is largely opaque. To further explore how the brain mediates damage, this work establishes *Drosophila melanogaster* as a model to examine complement-like pathways in reactive glia. Flies display a robust glial injury response, with a short generation time and bounty of genetic tools making them a vital paradigm to examine the basic mechanisms of glial reactivity. My analyses indicate that fly thioester-containing protein (TEP) family members Tep2 and Tep4 share ∼40% protein sequence similarity with the secreted mammalian C3 and C4b complement factors, with conservation in important functional domains. This work reveals that Tep2 and 4 are expressed by glial cells and are elevated in multiple paradigms of central nervous system injury. Furthermore, Tep2/4 localize around the site of axonal injury, and glial Tep knockdown inhibits axonal degeneration. I hypothesize that Drosophila Teps play mammalian complement-like roles in the fly brain and are involved in glial injury response. Overall, this project investigates glial complement-like functionality in the brain, parsing apart the roles these Teps play in reactive glia and injury, focusing on their impact and interactors.

A17

**ADVANCED TAU PATHOLOGY AND PRION-LIKE PROPAGATION IN A MONKEY MODEL OF ALZHEIMER'S DISEASE**

Danielle Beckman^1^, Sean Ott^1^, Amanda Dao^1^, Eric Zhou^1^, Kristine Donis-Cox^1^, William Jansen^2^, Scott Muller^3^, Paramita Chakrabarty^4^, Jeffrey Kordower^3^, John Morrison^1,5^

*^1^ University of California - Davis, California National Primate Research Center, Davis, USA*

*^2^ Icahn School of Medicine at Mount Sinai, Friedman Brain Institute, NY, USA*

*^3^ Rush University Medical Center, Department of Neurological Sciences, Chicago, USA*

*^4^ University of Florida, Center for Translational Research in Neurodegenerative Disease, Gainesville, USA*

*^5^ University of California - Davis, Department of Neurology, School of Medicine, Davis, USA*

Alzheimer's disease (AD) is a devastating condition that affects around 5.8 million of Americans, and these numbers are projected to reach nearly 14 million individuals around 2050. Although AD was first described more than 100 years ago, until nowadays that are not effective treatments to counteract or efficiently slow down the progression of the disease. This is likely, due at least in part, to the fact that AD animal models have been focused in the use of transgenic rodent models, failing to translate the findings to humans with the disease. We describe here a non-human primate model of AD presenting extensive tau pathology, mainly affecting the hippocampus and connected areas. Adult rhesus monkeys injected with adeno-associated viruses expressing mutant tau (P301L/S320F) in the left entorhinal cortex (ERC) show after 3 months, extensive prion-like tau propagation with full fibrillary tangles in the hippocampus, as well as pre-tangles and phospho-tau in projection areas such as the contralateral ERC and in the retrosplenial and visual cortices. Importantly, neuroinflammation is extensive in the affected areas, with a major role of microglia in pathological tau oligomer propagation. Finally, the mutant h4R injected coaptates monkey 3R tau generating more tau aggregation and spreading. These results highlight the first stages of tau pathology and propagation and support the importance of a non-human model of AD, with natural full expression of tau protein, that is highly translational to humans.

A18

**CENTRAL AND PERIPHERAL GLUCOSE ABERRATIONS RESULTING FROM AN ASTROCYTIC TRANSPORTER DEFECT IN ALZHEIMER'S DISEASE**

Steven Barger^1^, Jin Hee Sung^1^, Carter Pacheco^2^, Yang Ou^1^

*^1^ University Arkansas Med Sci, Geriatrics, Little Rock, USA*

*^2^ University Arkansas Med Sci, College of Medicine, Little Rock, USA*

Alzheimer's disease (AD) is associated with a reduction in cerebral glucose metabolism, and impaired glucose tolerance is also common in the periphery in AD. We detected aberrant subcellular localization of the GLUT1 glucose transporter in cells of the cerebral parenchyma in AD. Specifically, smaller fractions of GLUT1 were in the plasma membrane of cells that expressed a 45-kDa version of the transporter. Endothelial cells express a 55-kDa GLUT1, and this form of the protein was unaltered in AD, though its total levels were reduced in type-2 diabetes. We found these AD-related manifestations present in a mouse line that transgenically overexpresses human Aβ in the absence of an APP transgene. The 45-kDa protein has often been attributed to astrocytes, but considerable expression can be found in neurons as well. Astrocytes extend endfeet to the cerebrovasculature that are critical to the transport of glucose from endothelium into the deeper brain parenchyma. And while the lactate shuttle hypothesis posits that the fuel ultimately delivered to neurons is not glucose, it is clear that glucose must be imported into astrocytes as an initial step. We hypothesized that a drop in the functional amount of GLUT1 in astrocytes produces the aberrations in glucose metabolism found in AD, including retention of enough glucose in the blood to create peripheral glucose intolerance. To test this idea, we employed a conditional knockdown (cKD) of one allele of the GLUT1 gene (*Slc2a1*) using a Cre/Lox system wherein Cre is driven by a *Gfap* promoter. Gfap-cKD mice showed a reduction in cerebral meta-bolism of glucose, as well as peripheral glucose intolerance. These findings indicate that an impairment in roughly fifty percent of the glucose handled by astrocytes in the CNS can create not only significant reduction of brain glucose utilization but can also leave enough glucose in the blood to present with impaired glucose tolerance.

A19

**PLASMA NEURONAL-ORIGIN EXTRACELLULAR VESICLES PROVIDE BIOMARKERS FOR COGNITIVE IMPAIRMENT IN PARKINSON'S DISEASE**

Joseph Blommer^1^, Toni Pitcher^2^, Maja Mustapic^1^, Wassilios Meissner^3^, Tim Anderson^2^, Dimitrios Kapogiannis^1^

*^1^ National Institute on Aging, Laboratory of Clinical Investigation, Baltimore, USA*

*^2^ New Zealand Brain Research Institute, Parkinson's Research Group, Christchurch, New Zealand*

*^3^ University Hospital Bordeaux, Department of Neurology, Bordeaux, France*

**Background:** Parkinson's Disease (PD) pathogenesis involves α- synuclein accumulation, but also impaired insulin sensitivity, characterized by decreased Tyr and Ser insulin receptor substrate-1 (IRS- 1) phosphorylations. Some PD patients develop mild cognitive impairment (PD-MCI) or dementia (PD-D), partially from co-existing Alzheimer's disease (AD) pathology. Given its importance for disease prognosis, there is a need to develop biomarkers for distinguishing PD normal cognition (PD-N) from PD-MCI/D. Neuronal-origin extracellular vesicles (NEVs) contain pathogenic proteins and insulin signaling mediators.

**Methods:** From 104 PD-N, 83 PD-MCI, and 39 PD-D patients and 48 age/sex-matched Controls, we immunocaptured plasma NEVs using anti-L1CAM antibody. We performed electro-chemiluminescence immunoassays for: 1) pathogenic proteins (α- synuclein, amyloid-beta42, total and p181-tau); 2) insulin-signaling markers (pTyr20/pSer312-IRS-1, and mTOR/pmTOR).

**Results:** α-synuclein was lower in PD than Controls (p<0.01), decreased stepwise in PD-MCI and PD-D compared to PD-N (p<0.01), and tended to decrease with increasing motor symptom severity by MDS-UPDRS-III (p=0.06). Amyloid-beta42 trended towards being higher in PD-MCI and PD-D compared to PD-N (p=0.06). pTau181 was higher in PD patients than Controls (p<0.01) and in PD-MCI compared to PD-N (p<0.05) and tended to increase with MDS-UPDRS-III (p=0.09). Total Tau was not different between groups. pTyr20-IRS-1 was lower in PD than Controls and in PD-MCI compared to PD-N (p<0.05) and decreased with increasing MDS-UPDRS-III (p<0.01). The ratio pSer312/pTyr20-IRS-1 was higher in PD patients than Controls (p<0.05), and in PD-MCI (p<0.05) compared to PD-N.

**Conclusions:** PD patients with cognitive impairment exhibited lower NEV α-synuclein and pTyr20-IRS-1 and higher pTau181 levels than cognitively intact PD patients. Additionally, α-synuclein and pTyr20- IRS-1 were associated with PD motor symptom severity. Plasma NEVs are a valuable tool for discovering biomarkers in PD.

A20

**ROLE OF EXTRACELLULAR VESICLES IN C9ORF72-ALS**

Maria elena Cicardi

*Thomas Jefferson University, Neuroscience, Philadelphia, USA*

ALS is a neurodegenerative disease affecting upper and lower motor neurons. The most common ALS mutation is the G_4_C_2_ expansion in the *C9orf72*. The toxic mechanisms associated to G_4_C_2_ expansion are formation of nuclear RNA foci and aberrant repeats associated non-AUG (RAN) translation of five different dipeptide proteins (DPRs) : polyGA, polyGP, polyGR, polyPA, polyPR; the highest toxicity is associated to polyPR and polyGR. All the DPRs are loaded into the extracellular vesicles (EVs) which are organelles continuously released from cells mainly grouped into microvesicles and exosomes. In this work we studied toxicity associated to the production of DPR^+^ EVs and which pathological pathways are associated to their internalization. By a transwell system we put in not-direct contact NSC34 transiently transfected with plasmids expressing DPRs and rat primary cortical neurons (CNs) transfected with synapsin-driven TdTomato to follow them over time by live imaging. We found a significant decrease in viability of CNs that were in contact with NSC34 overexpressing polyGR with 50 repeats (GR50). To understand the contribution of polyGR EVs, we isolated EVs from culture media of NSC34 transfected with GR50 by ultracentrifugation: GR50 was present in both microvesicles and exosomes. When the EVs were added to the CNs media, we found that only polyGR^+^ exosomes population was able to recapitulate the toxicity observed in the transwell experiment. To directly prove the contribution of polyGR^+^ EVs, we inhibited the production of EVs in NSC34 with GW4869 and we performed the transwell experiment observing that the polyGR associated toxicity was abolished. We further investigated the contribution of different polyGR lengths finding that EVs loaded with GR100 caused an even bigger decrease in recipient CNs viability. We investigate if the reception of DPR^+^ EVs was also able to trigger the activation of known pathological mechanisms such as TDP43 mislocalization and RAN-translation activation; they both appear to be enhanced in neurons treated with GR50 EVs. In conclusion we showed that the production of EVs from ALS-affected neurons is a mechanism that can enhance and exacerbate the progression of ALS.

A21

**STRIATAL ASTROCYTES: IDENTITY, FORM AND FUNCTION**

Baljit Khakh

*UCLA, Physiology Department, Los Angeles, USA*

Astrocytes tile the central nervous system, but their functions in neural microcircuits in vivo and their roles in mammalian behavior and disease are incompletely defined. We will report data from the laboratory whereby we used 2-photon laser scanning microscopy (2PLSM), in vitro electrophysiology, in vivo electrophysiology/ imaging, immunohistochemistry, astrocyte specific RNA-Seq and new genetic approaches to reduce and activate striatal astrocyte signaling in brain slices and in vivo. The talk will report data from experiments that used these methods to explore the molecular identity, morphological form and functions of striatal astrocytes in physiology as well as in the context of mouse models of Huntington's disease. Taken together, the available data indicate that the striatum represents a useful brain nucleus to explore basic and disease related aspects of astrocyte biology.

A22

**CELL TYPE-SPECIFIC DELETIONS OF TFEB/TFE3 IN THE REGULATION OF BRAIN HOMEOSTASIS**

Henri Antikainen, Radek Dobrowolski, Haesun Kim

*Rutgers University, Biology, Newark, USA*

Transcription factor EB (TFEB) is a central regulator of lysosomal biogenesis and autophagy that along with its MiT/TFE family members responds to nutrient signaling and cellular stress. TFEB can promote brain homeostasis as seen in studies where neuronal and astrocytic overexpression of TFEB ameliorates outcomes in neuro-degenerative models. In prolonged cellular stress TFEB can promote cell death via integrated stress signaling. We have previously described an Alzheimer's disease-like phenotype with neuronal death and aggregating toxic proteins such as beta-amyloid and phospho-tau in TFEB fl/fl-nestin cre mice, which lack TFEB in neuronal progenitor cells. In this mouse line, three different mature cell lineages are affected by the genetic manipulation: neurons, oligodendrocytes and astrocytes. In the current study, we are examining the effects of TFEB loss in each of these cells types and their contributions to the disease-like phenotype. Oligodendrocytes do not appear to be affected by the loss of TFEB, as seen by their retained myelination capability in TFEB fl/fl-nestin cre mice. Astrocytes are more reactive in TFEB fl/fl-nestin cre in the corpus callosum, striatum and cortical layers. We are using astrocyte-specific TFEB and global TFE3 knockout mice to test whether the loss of astrocytic TFEB/TFE3 increases astrocyte reactivity and pro-inflammatory signaling. We are also using the KO mouse line in a traumatic brain injury model to determine effects of astrocytic TFEB/TFE3 loss on cellular outcomes of TBI, such as neuronal and astrocyte death, accumulation of toxic proteins and astrocyte and microglia reactivity.

A23

**A NOVEL STRATEGY TO IMAGE CALCIUM SIGNALING MECHANISMS OF NG2-GLIA FOLLOWING CNS VIRAL INFECTION**

Laura Bell, Glenna Wallis, Karen Wilcox

*University of Utah, Neuroscience, Pharmacology&Toxicology, Salt Lake City, USA*

NG2-glia are commonly known as oligodendrocyte progenitor cells. However, accumulating evidence suggests that these cells have many additional functions, leading to their classification as a major glial cell-type in their own right. We recently demonstrated that NG2-glia become reactive, increase proliferation, and participate in scar formation in the Theiler's Murine Encephalomyelitis Virus (TMEV) mouse model of infection-induced epilepsy. To investigate functional changes that may accompany NG2-glia reactive processes, we generated a triple-transgenic mouse to express a fluorescent reporter (tdTomato) and a genetically encoded calcium indicator (GCaMP6f) under control of an inducible NG2-promoter. This provides a novel approach to investigate damage-associated changes in calcium signaling in NG2-glia. Mice homozygous for tamoxifen-inducible cre and floxed-tdTomato were bred to mice homozygous for floxed-GCaMP6f to produce mice that are heterozygous for all three genes. Tamoxifen was administered (20mg/kg, i.p. in peanut oil, every-other-day for a total of three injections) to induce tdTomato and GCaMP6f expression specifically in NG2-glia. Time-series images were acquired using a 2-Photon microscope in coronal hippo-campal brain slices at least 60µM below the surface. Imaging rates were optimized between 1-5 Hz to monitor both spontaneous calcium transients and those evoked by the focal ATP (100uM) application. Both spontaneous and ATP-induced calcium transients were consistently observed in NG2-glia in the hippocampus of acutely prepared brain slices. While the application of ACSF alone did not induce pressure-sensitive calcium transients in NG2-glia, ATP application resulted in robust and reproducible calcium transients throughout both the soma and fine processes of NG2-glia (1-3 slices from n= 5 mice). Our approach provides a novel strategy to better understand the roles of NG2-glia in inflammatory states and in epilepsy development. We show that NG2-glia react to purinergic damage signals (ATP) through transient increases in intracellular calcium. Future work will determine whether calcium signaling is altered following TMEV infection at timepoints known to induce reactive changes in NG2-glia.

A24

**COMPLEMENT-DEPENDENT NEURON-GLIA INTERACTIONS MEDIATE THE LOSS OF PERISOMATIC INHIBITORY SYNAPSES IN TOXOPLASMA GONDII INFECTION**

Gabriela Carrillo^1^, Valerie Ballard^1^, Taylor Glausen^1^, Zack Boone^2^, Joseph Teamer^1^, Cyrus Hinkson^1^, Elizabeth Wohlfert^2^, Ira Blader^2^, Michael Fox^1^

*^1^ Virginia Tech, Fralin Biomedical Research Institute, Roanoke, USA*

*^2^ University at Buffalo, Department of Microbiology and Immunology, Buffalo, USA*

Prolonged infection and inflammation within the brain can alter the connectivity and function of neuronal circuits. The intracellular para-site, *Toxoplasma gondii*, is one pathogen that can chronically infect the brain and lead to encephalitis and seizures. Currently, 1 in 3 humans are infected with *Toxoplasma gondii* worldwide, and these infections have been identified as a considerable risk factor for developing complex neurological and psychiatric disorders that arise from alterations in assembly and maintenance of synapses, such as schizophrenia. To directly assess changes in inhibitory synapses, we employed Serial Block Face Scanning Electron Microcopy and quantified perisomatic synapses in neocortex and hippocampus following parasitic infection. Ultrastructural analyses, in combination with genetic and immunohistochemical tools, revealed that persistent infection not only led to a significant loss of inhibitory perisomatic synapses, it also induced the ensheathment of neuronal somata and perisomatic nerve terminals by activated myeloid-derived cells (including microglia), suggesting they may displace or phagocytose synaptic elements in long-term infection. How might microglia contribute to inhibitory synapse loss in parasitic infection? Based on the well-established role of the innate complement cascade contributing to synaptic refinement in the developing brain and synapse loss in the diseased brain, we assessed complement components in parasitic infection. Indeed, C1q and C3 are highly upregulated in neocortex and hippocampus following persistent *T. gondii* infection. Therefore, we hypothesized that these components may be required for microglia-induced synapse loss during long-term infection. Using transgenic and knockout mouse models, our current studies demonstrate that C3 is, in part, required for phagocytic microglia to ensheath neurons in long-term infection, likely leading to the loss of inhibitory perisomatic synapses. Together, these studies highlight novel ways in which immune molecules regulate neuron-glia interactions to alter mature inhibitory circuits in parasitic brain infection.

A25

**DIFFERENTIAL GENE EXPRESSION BETWEEN MICROGLIA AND MACROPHAGES DURING ACUTE CNS VIRAL INFECTION**

Ana Beatriz DePaula-Silva^1^, Carlos Gorbea^1^, Demian Cazalla^1^, Karen Wilcox^2^, Robert Fujinami^1^

*^1^ University of Utah, Pathology/Biochemistry, Salt Lake City, USA*

*^2^ University of Utah, Pharmacology & Toxicology, Salt Lake City, USA*

Worldwide, 50 million people have epilepsy, a severe neurological disorder characterized by recurrent seizures. Temporal lobe epilepsy (TLE) is the most prevalent form of acquired epilepsy and difficult to control with available anti-seizure drugs. Thus, new disease-modifying therapies are needed to treat and prevent seizures in high-risk groups. Although the precise mechanisms that lead to epilepsy remain unclear, evidence from experimental and clinical work suggests that inflammation is an important contributor. Brain inflammation, induced by viral infection of the central nervous system (CNS), alters the excitatory and inhibitory balance among neurons and is a significant cause of acute seizures.

We have developed an experimental model of virus-induced seizures/ epilepsy, an animal model of TLE. In our model, C57BL/6J mice infected intra-cranially with Theiler's murine encephalomyelitis virus (TMEV) develop encephalitis leading to acute seizures/epilepsy. We found that infiltrating macrophages play a key role in seizure development. We isolated infiltrating macrophages and resident microglia from the CNS of TMEV-infected mice and performed RNA-seq to determine the expression profiles of genes relating to CNS infection/inflammation. We identified new genes that are uniquely expressed by microglia or macrophages during the peak of neuroinflammation, and the function of these microglial-specific genes in the context of neuroinflammation is currently being explored. We found a population of infiltrating macrophages that expresses high levels of TREM-1 (triggering receptor expressed on myeloid cells-1). Activation of this receptor leads to the initiation and amplification of inflammatory responses through the production and secretion of inflammatory cytokines by these macrophages. We found that TREM-1 knockout mice have decreased seizure incidence and severity. We also found that the CNS-infiltrating macrophages had significantly diminished activation towards an inflammatory reactive state, as shown by lower major histocompatibility complex-II expression, in mice treated with TREM-1 inhibitor. We are continuing to explore the role of macrophages and microglial cells leading to inflammation and seizures.

A26

**IMPACT OF MILD LATERAL FLUID PERCUSSION INJURY ON MATURE PRE-EXISTING OLIGODENDROCYTES**

Alexandra Adams^1^, Jihyun Kim^1^, Bryan Pfister^2^, Haesun Kim^1^

*^1^ Rutgers University, Biological Sciences, Newark, USA*

*^2^ New Jersey Institute of Technology, Biomedical Engineering, Newark, USA*

**Impact of mild lateral fluid percussion injury on mature pre-existing oligodendrocytes**

Alexandra Adams, Jihyun Kim, Bryan J Pfister, Haesun A Kim Rutgers University, Biological Sciences, Newark, NJ, New Jersey Institute of Technology, Biomedical Engineering, Newark, NJ Myelin loss in brain is a common occurrence after traumatic brain injury (TBI) that results from impact-induced acceleration forces to the head. Axons within the white matter tracts are especially vulnerable to mechanical strain and subsequent axonal injury. Little is known about the effect of mechanical strain on mature oligo-dendrocytes and their associated myelin sheaths. We used an *in vitro* stretch device to characterize a mechanotransduction mechanism that mediates the oligodendrocyte response to injury. Stretch-injury caused transient and reversible myelin protein loss in oligodendro-cytes without cell death. Biochemical analyses revealed that the stretch-induced myelin protein loss was mediated by the release of calcium from the endoplasmic reticulum (ER) and consequent calcium-dependent activation of Erk1/2. Inhibition of Erk1/2 or ER calcium release was sufficient to attenuate stretch-induced myelin protein loss in mature oligodendrocytes. For *in vivo* studies, mild fluid percussion injury induced Erk1/2 activation in mature oligo-dendrocytes of the corpus callosum within 24 hours, in both focal and distal regions. Erk1/2 activation was accompanied by a decrease in the CC1^+^ immunoreactivity within 3 days. Axonal degeneration was evident in the focal but not the distal region, indicating that the loss of CC1^+^ cells in the distal area occurred independent of Wallerian degeneration. A significant decrease in the number of nodes of Ranvier was observed in both the distal and focal regions of the corpus callosum and conversely, an increase in the appearance of hemi-nodes, which indicates a disruption in the nodal-paranodal organization. Therefore, mild TBI elicits both focal and diffuse effects on mature oligodendrocytes and myelin. More importantly, the impact of mild TBI on the distal oligodendrocytes is manifested without oligodendrocyte death or axonal loss, suggesting an oligo-dendrocyte intrinsic response that disrupts myelin homeostasis.

A27

**INHIBITION OF BMP SIGNALING FOR PREVENTION OF CELL DEATH FROM CA^2+^ OVERLOAD IN CO-CULTURE MODEL OF SPINAL CORD INJURY**

Nadia Al-Sammarraie, Swapan K. Ray

*University of South Carolina School of Medicine, Department of Pathology Microbiology and Immunology, Columbia SC, USA*

Traumatic spinal cord injury (SCI) is the most prevalent injury to the spinal cord, most commonly resulting from car accidents. Direct injury to the spinal cord can cause immediate loss of neural tissue, including neurons and glial cells. Severe direct injury leads to permanent damage to the spinal cord at, above, and below the injury site, manifesting autonomic dysfunction, respiratory problems, paralysis, or even death. Previous studies in our laboratory indicated that SCI led to activation of multiple pathological pathways contributing to secondary damage and induction of apoptotic death in SCI lesion and penumbra. An earliest event in the pathogenesis in SCI is the increase in intracellular calcium ion (Ca^2+^), which is one of the most critical intracellular messengers that regulate multiple cellular functions in healthy and disease states. Similarly, bone morphogenetic protein (BMP) molecules, which are poly functional cytokines, are also increased during SCI and correlated with impaired autophagy and increased neuronal and glial death following injury. However, the link between intracellular Ca^2+^ overload and elevated BMP signaling in pathogenesis in SCI remains largely unknown. In this study, we aimed to understand the impact of inhibition of BMP signaling on Ca^2+^ mediated apoptotic death in neurons and astrocytes in co-culture model of SCI. First, tried to decipher how the intracellular Ca^2+^ overload affected the cell morphology and survival in co-culture model of SCI. Second, we aimed to evaluate the impact of inhibition of BMP signaling on survival and proliferation of both types. Next, we studied the molecular mechanisms by which Ca^2+^ disrupted auto-phagy flux and how inhibition of BMP signaling at an early stage of Ca^2+^ overload sustained autophagy flux and prevented apoptotic death. In conclusion, our study suggested BMP signaling as a potential therapeutic target in SCI for sustaining autophagy flux, protecting both neurons and astrocytes from Ca^2+^ overload, and improving outcomes in SCI.

A28

**REPETITIVE CLOSED HEAD INJURIES AND COGNITIVE DEFICITS IN RODENTS: IT THERE A ROLE FOR TAU?**

Adam Bachstetter^1^, Colleen Bodnar^1^, Josh Morganti^1^, Jose abisambra^1^

*^1^ University of Kentucky, SCoBIRC / Dept. of Neuroscience, Lexington, USA*

*^2^ University of Florida, Dept. of Neuroscience, Gainesville, USA*

As concussion has become a common household topic, the seriousness of these mild traumatic brain injuries (mTBI) has been lost in the general public. However, as scientists have continued to dive deep into the study of pathology and physiology following mTBI it has been found that the chronic pathology and behavioral dysfunction that is seen following a ‘mild’ TBI is anything but. Clinical-neuropathological correlation studies provide evidence that conversion of tau into abnormally phosphorylated proteotoxic intermediates (p-tau) could be part of the pathophysiology triggered by a single TBI and enhanced by repeated TBIs. However, the link between p-tau and mTBI in rodents remains controversial. To address this question experimentally, we induced a single or repetitive (two hit -2x) closed head injury (CHI) to WT or rTg4510 mice. We found that 2xCHI increased tau phosphorylation in WT mice and rTg4510 mice. Behavioral characterization in WT mice found chronic deficits in the radial arm water maze in 2x CHI mice that had partially resolved in the 1x CHI mice. Moreover, using Manganese-Enhanced Magnetic Resonance Imaging with R1 mapping – a novel functional neuroimaging technique – we found greater deficits in the rTg4510 mice following 2x CHI compared to 1x CHI. To integrate our findings with prior work in the field, we conducted a systematic review of rodent mild repetitive CHI studies. Following Prisma guidelines, we identified 25 original peer-reviewed papers. Results from our experiments, as well as our systematic review, provide compelling evidence that tau phosphorylation is modified by experimental mild TBI studies; however, p-tau level changes are not universally reported. Together, our results provide evidence that repetitive TBIs can result in worse and more persistent neurological deficits compared to a single TBI, but the direct link between the worsened outcome and elevated p-tau could not be established.

A29

**INTRANASAL ADMINISTRATION OF TYPE-1 INTERFERON SUPPRESSES EARLY VENEZUELAN EQUINE ENCEPHALITIS VIRUS INFECTION AND NEUROINVASION**

Matthew Cain, Mark Miller, Robyn Klein

*Washington University School of Medicine, Dept. of Medicine - Infectious Disease, St. Louis, USA*

Neuroinvasive alphaviruses, including Venezuelan equine encephalitis virus (VEEV), cause fatal encephalitis in humans. Unlike most arboviruses, aerosolized VEEV is infectious and can result in enhanced disease severity. Combined with its capability to be grown to high-titer, this makes VEEV a significant bioterrorist risk. Unfortunately, specific therapies for neuroinvasive alphaviruses are limited. Type I interferons (IFNs) have potent anti-viral activity and IFN signaling is associated with restricting infection and viral clearance**.** Pretreatment with exogenous IFNα has been demonstrated to protect against VEEV infection. However, the capacity of type-1 IFN treatment to limit VEEV post-exposure has not been demonstrated. This study assessed neuroinvasion and IFN innate immune responses during intranasal VEEV infection in mice. In this lethal model of infection, VEEV arrives in the CNS by 24 hours post-infection. VEEV predominately spreads along the olfactory tract, with GAP43+ immature olfactory sensory neurons within the olfactory neuroepithelium (ONE) acting as an early site of infection. Despite rapid VEEV neuroinvasion, host CNS type-1 IFN response is delayed compared to VEEV CNS entry, representing a potential therapeutic window. This study evaluated the efficacy of recombinant IFNα administered intranasally during VEEV infection. Importantly, this IFNα treatment delayed onset of sequelae associated with VEEV encephalitis and extended survival by several days. 2-photon imaging and immunohistochemistry demonstrated targeted protection of both the ONE and olfactory tract within the brain during early infection. Additionally, this study evaluated the CNS IFN response triggered by intranasal IFNα treatment to identify interferon-stimulated genes (ISGs) that are associated with suppression of VEEV infection. To-gether these data demonstrate the efficacy of intranasal delivery of IFNα on limiting early VEEV CNS infection and represent a critical and promising first evaluation of such a treatment strategy for human alphavirus infection.

A30

**CONFOCAL MICRO RAMAN SPECTROSCOPIC STUDY ON THE EFFECT OF FLUOXETINE AND ETHANOLIC EXTRACT OF GARLIC IN THE BRAIN OF DEPRESSED RAT**

Khushboo.

*University of Allahabad, Department of Biochemistry, Allahabad, India*

Khushboo and Bechan Sharma#

Department of Biochemistry, University of Allahabad, Allahabad 211002, UP-India;

A depressive disorder is among the most common psychiatric diseases and is expected to become the second most common form of psychiatric illness by the year 2020 and affects around 350 million people worldwide. Usually, existing antidepressant drugs used for neurological disorders have several side effects. Fluoxetine is a second-generation antidepressant drug and the administration of fluo-xetine (SSRI) has been shown to suppress the growth of glioblastoma cells and expression of BDNF, which resulted in the enhancement of long-term effects on the brain. As an outcome of this, there is growing consideration of workers towards exploring relatively safer, efficient, and more cost-effective antidepressant molecules isolated from plant extracts. In the current study, we have investigated the antidepressant, neuroprotective and antioxidant effects of garlic extract against reserpine induced depression in the experimental rat model. Animals were divided into four different groups: (1) control, (2) reserpine, (3) reserpine with garlic extract, and (4) reserpine with fluoxetine. The forced swimming test was used to evaluate the anti-depressant activity of garlic extract and fluoxetine. The superoxide dismutase, glutathione transferase, catalase, and some other metabolic enzyme levels (antioxidant enzymes) were significantly decreased in the depressed rat brain. The levels of serotonin and acetylcholinesterase activity were also altered due to reserpine; fluo-xetine and garlic extract treatment, which suggested the alteration in biosynthesis and degradation of serotonin taking place in the rat brain and were acquired by using Confocal micro Raman spectrometer equipped with a BX43 microscope excited by 785 nm laser source together with 10x eyepiece. The obtained spectral features have been pre-processed and correlated with biochemicals present in the cerebellum, cerebrum, hippocampus, hypothalamus, and neocortex and we found significant alterations, absence, and presence of different biochemicals in our respective treatments. The study showed the significant neuroprotective and antidepressant activity of garlic extract against reserpine induced depression.

A31

**METHYL JASMONATE RESCUES NEURONAL CONNECTIVITY DEFECTS IN THE UNPREDICTABLE CHRONIC MILD STRESS MOUSE MODEL OF DEPRESSION**

Oritoke Aluko^1,2,3^, Annabella Pignataro^2^, Omamuyovwi Ijomone^1^, Solomon Umukoro^3^, Martine Ammassari-Teule^2^

*^1^ Federal University of Technology, Akure, Nigeria, Department of Physiology, Akure, Nigeria*

*^2^ IRCCS, Santa Lucia Foundation, Rome, Italy, Department of Neurobiology, Rome, Italy*

*^3^ University of Ibadan, Ibadan, Nigeria, Department of Pharmacology and Therapeutics, Ibadan, Nigeria*

Human brain imaging studies show that depression alters structural and functional connectivity in brain circuits governing cognition and emotions. Consistent with this, the unpredictable chronic mild stress (UCMS), a mouse model of depression, causes dendritic spine and dendrite arbor retraction in principal neurons together with disruption in Parvalbumin-positive (PV^+^) interneurons in cortico-limbic regions involved in the control of mood. Based on data showing that the plant stress hormone - methyl jasmonate (MJ) rescues the UCMS-induced depressive behavioral phenotype, here we examine whether the compound also prevents neuronal connectivity defects in the basolateral amygdala (BLA), CA1 hippocampus subfield (CA1), and medial prefrontal cortex (PFC). Male C57BL/6 mice were injected with MJ (50 mg/kg) or saline (SAL) before each of the two daily exposure to unpredictable mild stressors administered over ten days. On day 11, mice were sacrificed for Golgi-staining and immuno-histochemistry analyses. Results showed that mice exposed to UCMS exhibited a massive reduction in spine density and dendritic arbor extension and fewer PV^+^ cells in the three regions examined. MJ treatment alleviated structural alterations in all regions but more in PFC than in BLA and CA1. The treatment also reduced PV+ cell loss in all regions but more in BLA and CA1 than in PFC, where those cells were mainly disrupted by UCMS exposure. Thus, in parallel with the alleviation of behavioral markers of depression, MJ preserves neurons' morphological integrity and rescues the PV+ regulated excitatory/inhibitory balance in the mood circuitry. The global reinstatement of physiological levels of neuronal connectivity and activity supports the MJ's relevance to treating depressive subtypes associated with a parallel collapse of dendrite morphology and PV+ cells at multiple brain sites.

A32

**PROGESTERONE IMPROVES COGNITION AND NEURONAL PLASTICITY IN AGED C57BL/6 MALE MICE**

Soumyabrata Banerjee, Kenneth Jenrow, Julien Rossignol, Raj Bose, Gary Dunbar

*Central Michigan University, Psychology, Neuroscience Program, Mount Pleasant, USA*

Progesterone is a multifaceted hormone and neurosteroid found in both males and females. During aging, serum levels of progesterone decline. The neuroprotective role of progesterone is documented in many studies and includes reduction in traumatic-brain-injury-induced behavioral deficits and associated increases in antioxidant catalase activity. Although the cognitive-enhancing effects of progesterone are well documented, less is known about its effects on age-related cognitive impairment. Our hypothesis was that exogenous administration of progesterone would reduce age-related deficits in cognition and LTP in male mice. To test this, we used groups of young (4-month-old) and aged (24-26-month-old) male C57BL/6 mice. Daily subcutaneous injections of progesterone (5 mg/ kg) or equivalent volumes (200 µl) of vehicle (30% 2-hydroxy beta-cyclodextrine) were administered to cohorts from each group for 21 consecutive days. Cognitive functioning was assessed during the 21-days treatment interval using a water-T-maze reversal task. Mice had to locate a hidden platform in one of two arms of the maze, in which the location was switched once the mouse achieved an 80% success rate. At the end of the 21-day treatment period, long-term potentiation (LTP) was recorded in the dentate gyrus (DG) following theta-burst stimulation of the medial perforant path. Results revealed that aged, vehicle-treated mice attained fewer reversals and required more days to complete each reversal than young vehicle-treated mice. The LTP was also reduced in these aged mice. Treatment of progesterone improved the performance of aged mice, resulting in an increase in number of reversals attained and requiring fewer days to complete each reversal. LTP was also improved and was closer to that observed in young, vehicle-treated mice. No significant changes were observed in young mice receiving progesterone treatments. Our results indicate that progesterone treatment (5 mg/kg/day, s.c. for 21 consecutive days) attenuates age-related cognitive impairment and enhances neuronal plasticity relative to aged, vehicle-treated controls. *This work financially supported by the John G.Kulhavi Professorship in Neuroscience at CMU and the Filed Neurosciences Institute.*

A33

**ROLE OF ADENOSINE RECEPTOR SUBTYPES A1 AND A2A IN DEMYELINATION AND REMYELINATION IN CUPRIZONE MODEL OF MULTIPLE SCLEROSIS**

Olamide Adebiyi^1,2^, Margaret Bynoe^1^

*^1^ Cornell University, Microbiology&Immunology, Ithaca, USA*

*^2^ University of Ibadan, Veterinary Physiology&Biochemistry, Ibadan, Nigeria*

Multiple sclerosis (MS) is a debilitating, neuroinflammatory and demyelinating disease characterized by sensory changes, visual impairment, motor dysfunction leading to irreversible disability. We investigated the role and contribution of A1 and A2A adenosine receptors (ARs) in oligodendrocyte precursor cell (OPC), oligo-dendrocyte (OL) and myelin regulation in cuprizone model of MS. Multiple sclerosis was induced in wild type C57BL/6 mice (WT) or C57BL/6 mice with global deletion of A1 adenosine receptor (A1 AR-/-) or A2A AR (A2A AR-/-), by feeding them 0.2% cuprizone diet (CuD) for four weeks. Luxol fast blue, Nissl, immunofluo-rescence staining, Western blotting, and several behavioral tests were performed to evaluate demyelination/remyelination.

At three weeks post CuD, A1 AR-/- mice began to show signs of lethargy, weight loss, and shivered vigorously. The corpus callosum, hippocampus of A1 AR-/- mice was completely demyelinated. This contrasted with A2A AR-/- mice that had a significant increase in myelin or hypermyelination, whereas WT mice on CuD had inter-mediate myelin quantity. Memory and olfactory sensitivity were also impaired in A1 AR-/- mice with signs of anxiety. Immunofluo-rescence staining with GFAP, IBA1 revealed astrogliosis in the AR knock out mice. Western blot assay quantification of MOG revealed a significant decrease in A1 AR-/- but an increase in A2A AR-/- mice. NeuN and MBP immunofluorescence staining also followed similar trend. However, olig2 was upregulated and downregulated in A1 AR-/- and A2A AR-/-, mice respectively. Using TUNEL staining, we verified that knocking out the A1 AR caused a 30-40 fold increase of apoptotic cells in different brain regions. Furthermore, WT mice on cuprizone diet showed significant decrease in numbers of A1 AR in the brain.

These results indicate that A1 and A2A ARs are involved in OPC/OL functions and suggest their opposing roles in myelin, OPC and OL regulation. We conclude that the neuropathological findings in multiple sclerosis may be connected to the depletion of A1 adenosine receptors.

A34

**ENDOTHELIN-1 SIGNALING REGULATES NEURAL STEM CELLS IN THE HEALTHY AND INJURED ADULT BRAIN**

Katrina Adams^1^, Payal Banerjee^1^, Marianna Bugiani^2^, Vittorio Gallo^1^

*^1^ Children's National Medical Center, Center for Neuroscience Research, Washington, USA*

*^2^ VU University Medical Center, Department of Pathology, Amsterdam, Netherlands*

Demyelination occurs in a large variety of central nervous system (CNS) insults, pathologies, and neurodegenerative diseases, including Multiple Sclerosis (MS). Parenchymal oligodendrocyte progenitor cells (OPCs) located throughout the CNS participate in endogenous remyelination of white matter lesions, migrating to the injury site and maturing into myelinating oligodendrocytes, albeit at low levels. An additional source of OPCs following CNS injury is neural stem cell (NSC)-derived OPCs generated in the subventricular zone (SVZ) of the lateral ventricles. Therefore, promoting increased levels of NSC gliogenesis is a promising therapeutic strategy for demyelinating disorders like MS. We previously identified the Endothelin-1 (ET-1) signaling pathway as a novel regulator of NSC and OPC proliferation during early postnatal development (Adams et al. 2020). Therefore, we asked whether ET-1 also regulates stem and progenitor cells in the adult SVZ, both during homeostasis and after demyelinating injury. We found that the majority of NSC populations in the adult mouse SVZ express the ET-1 receptor, Ednrb. Ablation of Ednrb from NSCs reduced both the number of activated and quiescent NSCs, indicating that ET-1 signaling is required for maintenance of NSCs in the adult mouse. Following focal demyelination of the corpus callosum, SVZ NSCs upregulated expression of ET-1. Ablation of ET-1 reduced the percentages of proliferating NSCs and proliferating OPCs in the SVZ, suggesting that ET-1 plays a critical role in the SVZ proliferative response to injury. RNAseq of cultured primary NSCs and OPCs treated with ET-1 identified genes involved in stem cell maintenance, including Notch signaling, and OPC migration. Lastly, we confirmed that ET-1 and EDNRB expression are conserved in the adult human SVZ, indicating that this pathway may be a potential target for promoting SVZ-mediated cellular repair.

A35

**MITOCHONDRIAL DYNAMICS ARE ALTERED IN CHRONIC EXPERIMENTAL AUTOIMMUNE ENCEPHALOMYELITIS LONGITUDINALLY**

Kelley Atkinson, Marvellous Osunde, Micah Feri, Saima Noori, Sandhya Sriram, Wendy Rincon, Maria Sekyi, Adam Alluis, Teressa Benbarka, Seema Tiwari-Woodruff

*University of California, Riverside, Biomedical Sciences, Riverside, USA*

Postmortem studies of multiple sclerosis (MS) patients demonstrate decreased mitochondrial activity in addition to inflammation throughout the central nervous system. Gait abnormalities in MS patients have been linked to Purkinje cell (PC) demyelination, blebbing axons, and atrophy of dendrites in the cerebellum. Similar changes in PCs are also observed in experimental autoimmune encephalomyelitis (EAE). In addition, axonal degeneration is linked to loss of metabolic support and demyelination. We hypothesize that mitochondrial dysfunction causes axonal degeneration in the context of inflammatory degeneration. To test this hypothesis, cerebellar pathology was investigated longitudinally in EAE disease course from peak disease (day 21) to late disease (day 90). Behavior (walking gait test and rotarod), pathology (immunohistochemistry and Western blot), and mitochondrial function (Seahorse XFp Mito Stress Test) were assessed. From peak disease to late disease, the average EAE clinical score was 2.5. Behavioral tests showed that EAE mice had decreased time on the rotarod and decreased stride length compared to normal. Similarly, increased inflammation and decreased myelination were observed at all timepoints. Mitochondria electron transport chain levels showed no change at peak disease, but chronic EAE showed decreased COXIV, ATP Synthase, mito-chondria fusion (Mfn2), and increased mitochondrial fission (Drp1). Mitochondria dysfunction was evident with decreased basal respiration beginning at peak disease and lasting through chronic disease. These data demonstrate that mitochondria dysfunction is evident by peak disease following irreversible axon damage. Future studies will determine whether early treatment with an estrogen receptor beta ligand before axon damage occurs can alleviate mito-chondria dysfunction and slow neurodegeneration in EAE.

A36

**EXTRACELLULAR VESICLES FROM MULTIPLE SCLEROSIS B CELLS AS MEDIATORS OF OLIGODENDROGLIAL TOXICITY**

Joyce Benjamins^1^, Liljana Nedelkoska^1^, Hanane Touil^3^, Paul Stemmer^2^, Nicholas Carruthers^2^, Leah Zuroff^3^, Amit Bar-Or^3^, Robert Lisak^1^

*^1^ Wayne State Univ Sch Med, Dept Neurology, Detroit, USA*

*^2^ Wayne State University, Institute of Environmental Health Science, Detroit, USA*

*^3^ University of Pennsylvania, Dept Neurology, Philadelphia, USA*

**Objective:** To characterize extracellular vesicles (EVs) released by B cells from multiple sclerosis (MS) patients.

**Background:** In MS, B cells mediate pathogenesis by mechanisms separate from immunoglobulin (Ig) production. Supernatants (Sup) from B cell cultures from 53 MS patients but only 1 of 53 matched controls kill cultured rat oligodendroglia (OL) (Lisak et al. 2012, 2017, unpublished). Killing does not require complement, and does not correlate with Sup levels of IgG, IgM or cytokines tested. OL death is mediated by factors in EVs (Benjamins et al. 2019).

**Methods:** B cells were cultured in EV-depleted serum-free medium. EVs were prepared from Sup by filtration and ultracentrifugation. Sup or EVs were diluted 1:4 with OL culture medium and tested for OL toxicity.

**Results**: Analysis of EV particle size showed a major peak between 70-90 nm, consistent with the size of exosomes. Proteomics analysis of MS and control EVs showed characteristic exosomal markers and B cell proteins. We developed methods for proteomic analysis of the low amounts of protein in the isolated EVs, based on TMT multi-plexing, and a strategy for RNASeq, lipidomic and integrated bio-informatic analyses. Studies in progress will give sample-size estimates based on analysis of variability for detection of significant differences between MS and control.

**Conclusions:** Both particle sizing and proteomics indicate that EVs released by MS and control B cells consist primarily of particles with the properties of exosomes. A multi-omics approach may allow identification of candidates responsible for OL toxicity in exosome-enriched fractions from MS B cells.

**Funding:** NIH R21 NS118227 (RPL, AB-O), National MS Society, #4929A2/3 (RPL, JAB, AB-O); MS Society of Canada #204209

(AB-O); Parker Webber Chair in Neurology Endowment, DMC Foundation/WSU School of Medicine (RPL); NIH P30 ES020957, P30 CA022453, S10OD010700 (PMS)

A37

**TRANSCRIPTOME PROFILE OF YOUNG VS MATURE MAGNOCELLULAR NEURONS AND ASSOCIATED GLIAL CELLS IN THE NEUROHYPOPHYSIS**

Malak Alzidaneen, Derick Thompson, Abiodun Odufuwa, John Watt

*University of North Dakota, Biomedical sciences, Grand Forks, USA*

Mature mammalian CNS neurons do not regenerate or recover following injuries, ischemic events or neurodegenerative disease. It is confirmed that there are different transcription factors that have been recently linked to axonal growth and survival, changes in regulation of these transcription factors with aging will eventually affect the gene expression levels of many other genes including those involved in axonal regeneration. It has been previously shown that there is a robust collateral sprouting response within the distant terminal field of magnocellular neurons, the neurohypophysis, arising from the contralateral non-injured supraoptic neurons in response to unilateral denervation of MCNs tracts, the response peaked at age 35 days then it was followed by a complete loss of the regenerative capacity between 35 days and 125 days of age. Our aim is to compare the transcription profile between young regenerating neurons and mature non-regenerating neurons and thier associated glia, the pituicytes, in the neurohypophysis. To address these questions we will be using the hypothalamic-neurohypophysis system as a model system; a unique CNS system with which we can study the epigenetic changes that undelie the decline in neuronal plasticity in the context of dynamic neuronal-glial interaction. RNA seq analysis and enrichement analysis would help determnine which genes are up-regulated or down-regulated between the two age groups and how these changes in gene expression can relate to age associated alterations in neuronal survival and axonal outgrowth.

A38

**EXPRESSION OF THE EXTRACELLULAR MATRIX-ASSOCIATED MOLECULES IN CULTURED ASTROCYTES AND IN VIVO**

Irina Dominova

*Immanuel Kant Baltic Federal University, School of Life Sciences, Kaliningrad, Russia*

It is now well-known that astrocytes are active participants in the tripartite synapse. However, today there is an opinion that synapse represents the tetrapartite structure, which includes additionally the extracellular matrix (ECM), which is formed by glycoproteins and proteoglycans, released by neurons and glial cells. ECM does not only affect the synapse stability and function but the function of the glial cell as well. The aim was to study diversity in the expression values (EVs) of ECM-associated molecules (ECM-AMs) in cultured astrocytes and in vivo.

In the present study, I demonstrate heterogeneity in ECM-AMs EVs between astrocytes in vivo obtained by immunomagnetic separation from P3 rats, and cultured astrocytes from the Bst, Ctx and Hip. Transcriptomic data demonstrated the increase in EVs of ECM-AMs genes in cultures. Studies of the ECM-AMs gene families showed EVs of most Col family were increased in Bst, whereas in Hip EVs of the most genes were decreased in cultures. In all comparison groups, EVs of Col1a1 and Col4a1 were increased. For Thbs family genes we found in the cultures EVs were increased including Thbs1 and Thbs2. Itg family genes EVs showed up-and downregulation in the same comparison group. Analysis of Lam family genes EVs revealed the most Lam genes were upregulated in all groups, e.g. Lamc1 and Lamb2 were increased in all groups. A similar tendency was found for Sdc genes EVs, Sdc1 and Sdc4 were upregulated in cultures of Ctx and Hip, whereas Sdc1 was upregulated in the culture of Bst. Fn1, Spp1, CD44 were increased in all groups but TnN and Hmmr were increased in cultures of Bst and Ctx. In contrast, Agrn and Reln were downregulated in cultures of Bst and Ctx, and Ctx and Hip, respectively.

Altogether obtained data indicate the significant contribution of glial cells in releasing of ECM-AMs and formation ECM in the brain, as well as on dramatic differences between cultured astrocytes and cells in vivo.

This study was financially supported by the Grant of the Russian Foundation for Basic Research 18-34-00152.

A39

**MICRORNAS TARGETING NEURONAL NMDA RECEPTORS**

Sowmya Gunasekaran, R.V. Omkumar

*Rajiv Gandhi centre for Biotechnology, Molecular Neurobiology Division, Thiruvananthapuram, India*

N-Methyl-D-Aspartate Receptors (NMDARs) are glutamate-gated calcium channels, which play a major role in synaptic plasticity. Dysregulation of NMDAR is associated with several neuro-degenerative and neuropsychiatric disorders. The NMDAR subunits GluN2A/*Grin2A* and GluN2B/*Grin2B* have a developmentally regu-lated expression profile (Cathala L et al., 2002, J.Neurosci.,20. p5899). We found that *Grin2B* expression is highest in days *in vitro* 7-9 (DIV 7-9) in rat hippocampal primary neuronal cultures and declines thereafter. *Grin2A* expression becomes significant by DIV 7-9 and the level is maintained subsequently.

Drugs that target NMDAR have major side effects and hence alternate subtle strategies are needed to indirectly target these receptors. The role of microRNAs (miRNAs) in neurological disorders is being widely explored (Wang W. et al, 2012, Learn. Mem.,19. p359). Our objective is to understand the mechanism of action of some miRNAs involved in NMDAR-mediated synaptic plasticity that are also differentially expressed in disease conditions. Some miRNAs that are altered in schizophrenia, Huntington disease and autism were predicted to interact with NMDAR subunits. Luciferase assays showed that miRNAs such as miR-223 and miR-129 interacted with the subunits of NMDAR. Expression levels of the target proteins in primary hippocampal neurons was downregulated after transfection of miRNA. To study regulation by these miRNAs *in vivo* we have established the MK-801 model and the MAM model of Schizophrenia. We have injected rats with MK-801 at post natal days 30-40 for five days followed by five day washout period. We injected MAM at gestational day 17 and the pups were weaned at P30. The animals underwent different behavior tests such as open field test, object recognition test and Morris water maze test. It was found that the treated animals have major cognitive impairment. Expression of some of the miRNA/s and their target proteins in these animals is under investigation. These studies may unravel novel mechanisms, which can be of therapeutic potential.

A40

**CIRCADIAN RHYTHMS IN MOTOR CORTEX PERSIST IN VIVO BUT NOT EX VIVO**

Ilia Katritch, Erik Herzog

*Washington University in St Louis, Biology, Saint Louis, USA*

Many behaviors in animals, such as sleep and feeding, are often only performed at certain times of day. However, even in the absence of a daily environmental cycle, this circadian behavioral rhythmicity will persist, organized around the animal's internal representation of time of day. In mammals, many cells maintain their own self-sustaining molecular core clock, characterized by the daily rise and fall of certain circadian proteins*.* The many cellular clocks of the body are entrained to the earth's daily cycle by a hypothalamic brain region, the Suprachiasmatic Nucleus (SCN). However, how this internal clock is translated from SCN rhythms to behavior is still unexplored. Our project studies the mechanisms driving circadian rhythms in primary motor cortex (M1), as it controls many voluntary motor behaviors separating the role of circadian rhythms in cortical neurons and astrocytes. As a measure of molecular clock function, we recorded the expression of the core clock protein PER2 using the bioluminescent reporter luciferase fused to PER2 (PER2::LUC) recorded in vivo in anesthetized animals with a CCD camera and ex vivo from cultured M1 slice, or *per2* promoter expression in either neurons or astrocytes in freely behaving mice using virally induced expression of the fluorescent reporter *per2*-Venus, recorded directly from M1 using an optic fiber. We found that, in intact animal cortical PER2 expression is rhythmic, peaking in the middle of the light phase. *per2* promoter expression is also rhythmic in both neurons and astrocytes, but peaks during the dark phase. In ex vivo M1 slice, PER2 rhythmicity is lost upon excision but are restored with a single addition of glucocorticoid agonist Dexamethasone (DEX) for up to 4 days. We concluded that the molecular clock in Neocortex requires Glucocorticoid input to remain rhythmic.

A41

**EXAMINING BDNF/ASTROCYTIC TRKB.T1 SIGNALING AS A MECHANISM UNDERLYING PERISYNAPTIC ASTROCYTE PROCESS RECRUITMENT**

Beatriz Torres, Leanne Holt, Michelle Olsen

*Virginia Polytechnic Institute and State University, School of Neuroscience, Blacksburg, USA*

Astrocytes are a morphologically complex cell type and that have emerged as critical players in both the development and physiology of the central nervous system (CNS). Their morphological complexity allows astrocytes to dynamically interact with neuronal synapses, where they establish and maintain synaptic connectivity by cradling synapses with peripheral astrocyte processes (PAPs). Astrocyte morphological maturation and the formation of PAPs occurs concurrently with synapse development. However, the signaling mechanisms that draw a PAP to “cradle” the synapse are not understood. In neurons, brain derived neurotrophic factor (BDNF) promotes neuronal growth, survival, and synaptic refinement. RNA sequencing data from isolated astrocytes has demonstrated that astro-cytes express high levels of the BDNF receptor, TrkB. Specifically, they predominantly express a truncated form of TrkB, TrkB.T1. Our recent work suggests that BDNF/TrkB.T1 signaling in astrocytes is an important signaling mechanism underlying astrocyte morphogenesis. Here, we examine if BDNF/TrkB.T1 signaling in astrocytes mediates the arrival of PAPs to developing synapses. *In vitro* co-cultures of wild type neurons with TrkB.T1 deficient astro-cytes demonstrates that these morphologically immature astrocytes do not support normal synaptogenesis and function. *In vivo*, using TrkB.T1 knockout mice, immunohistochemistry and analysis of structurally formed synapses via pre-and post-synaptic (VGluT1/ PSD95) co-localization suggests this receptor may mediate normal synapse formation. We further aim to manipulate the whisker barrel cortex to examine the arrival of PAPs to a synapse and the relevance of BDNF/TrkB.T1 signaling in this astrocyte experience-dependent structural plasticity. Alterations in synaptic function and development have been implicated in multiple neurodevelopmental disorders, but the majority of the research has only focused on the neuronal players. This work will advance the understanding of the role of astrocytes in synaptic development and offer new therapeutic targets where synapse development is implicated.

A42

**UPREGULATION OF VOLTAGE-GATED CALCIUM CHANNEL SUBUNIT Α2Δ1 MEDIATES CHRONIC STRESS-INDUCED ENHANCEMENT OF NMDA RECEPTOR ACTIVITY**

De-Pei Li, PeiRu Zheng

*University of Missouri, Medicine, Columbia, USA*

Chronic unpredictable stress (CUS) augments NMDA receptor activity in corticotropin-releasing hormone (CRH) neurons in the hypothalamic paraventricular nucleus (PVN). However, the underlying mechanisms are unknown. Recent studies have shown that NMDA receptor activity is regulated by voltage-gated Ca^++^ channel (VGCC) subunit α2δ1. In this study, we tested a hypothesis that chronic stress increases NMDA receptor activity through upregulation of α2δ1 subunit. Rat hypothalamic CRH neurons were identified by specific expression of GFP driven by CRH premotor. CUS increased α2δ1 protein expression level in the PVN tissue. Immuno-staining revealed that α2δ1 was distributed on hypothalamic CRH neurons. In brain slice preparation, NMDA receptor blocker AP5 decreased the firing activity of PVN-CRH neurons in CUS rats while had no effect on firing activity in un-stressed rats. However, disruption of α2δ1 binding with NMDA receptor with gabapentin eliminated inhibitory effect of AP5 on firing activity of PVN-CRH neurons in CUS rats. Furthermore, CUS increased NMDA currents elicited by puff application of NMDA onto PVN-CRH neurons. Gabapentin normalized the increase in NMDA currents in CUS rats. Microinfusion of either gabapentin or AP5 into the PVN through an implanted cannula targeting the PVN normalized high CORT levels in CUS rats. Infusion of gabapentin plus AP5 into the PVN did not induce further decreased in CORT levels in CUS rats. Taken together, these data suggest that chronic unpredictable stress increases NMDA receptor activity through upregulation of α2δ1 subunit. Disruption of NMDA receptor binding with α2δ1 normalize hyperactivity of PVN-CRH neurons and HPA axis.

A43

**HIPPOCAMPAL 5HT1A AND 5HTT ALTERATIONS LEAD TO COGNITIVE DEFICITS ASSOCIATED WITH MAJOR DEPRESSIVE-LIKE BEHAVIORS IN RATS**

Gwladys Ngoupaye Temkou^1,2^, Thobeka Madlala^2^, Makwena Mokgokong^2^, Musa Mabandla^2^

*^1^ Faculty of Science, University of Dschang, Animal Biology, Dschang, Cameroon*

*^2^ Discipline of Human Physiology, School of Laboratory Medicine & Medical Sciences, College of Health Sciences, School of Laboratory Medicine & Medical Sciences, Durban, South Africa*

Current models used to study the pathophysiology of major depressive disorder (MDD) are laborious and time-consuming. This study characterized the impact of a 14-day combined stress model (CS; corticosterone injection (40mg/kg, s.c.) and chronic immobilization stress) in adult male Sprague Dawley rats. Depressive-like behaviors and cognitive deficits were assessed in the Sucrose preference and Morris Water Maze (MWM) tests, respectively. Subsequently, effects of the stress model on the serotonergic system (measured by examining the concentration of acetylcholinesterase (AChE) and expressions of the serotonin transporter (5-HTT) and serotonin 1A receptor (5-HT1A)) were determined in the hippocampus and prefrontal cortex (PFC), regions implicated in the pathophysiology of MDD. Findings show that the CS group expressed increases in time to reach the MWM platform, PFC 5-HT1A, and hippocampal 5- HT1A and AChE, but reductions in sucrose preference ratio, time in MWM target quadrant, and hippocampal 5-HTT compared to their control group. Compared to the 28 days of the corticosterone-only group, PFC 5-HT1A was reduced, while 5-HTT was higher in the CS group. Taken together, our CS model induced depressive-like behavior with early cognitive deficits in rats, mainly affecting the hippocampus. The CS model can be useful in investigating new and comprehensive treatment strategies for MDD.

A44

**NEW TOOLS TO IMAGE GAP JUNCTION STRUCTURE AND SIGNALING IN GLIA**

Randy Stout^1^, Antonio Cibelli^2^, Daniel Tannis^1^, David Spray^2^

*^1^ New York Institute of Technology College of Osteopathic Medicine, Department of Biomedical Sciences, Old Westbury, USA*

*^2^ Albert Einstein College of Medicine, Dominick P. Purpura Department of Neuroscience, Bronx, USA*

The gap junction nexus is a supramolecular structure at which connexin-based gap junction channels cluster to join adjacent cellular compartments. Gap junctions are prominent in the brain and act as adhesions, serve as platforms that modulate cell morphology, and mediate intercellular communication. Gap junctions in astrocytes and oligodendrocytes are required for normal brain function. Fluorescent protein tags and live-cell microscopy have been important approaches in gap junction research. These tools have synergized with electrophysiology techniques to greatly enhance our understanding mechanisms by which gap junctions contribute to brain health and cognition. A major gap junction isoform expressed in astrocytes is connexin 43 (Cx43). Cx43 has been studied extensively using fluorescent protein tags but tagging Cx43 with peptide additions generates problematic collateral effects by either completely eliminating channel function (amino-terminal tag) or by preventing protein binding of important regulators of channel function and gap junction structure (carboxyl-terminal tag). We present new tagging strategies that allow channel function while maintaining many of the protein interactions with the carboxyl-terminus of Cx43. We present insights gained new microscopy and image analysis techniques. We use virtual reality and spatial computing approaches to examine interactions between the gap junction nexus and the other organelles (e.g. mitochondria and endoplasmic reticulum). Finally, we will present results of computational simulations that suggest proximity to other organelles and nexus structural stability may modulate intercellular calcium signaling through gap junctions.

A45

**ACYL-COA SYNTHETASE 6-MEDIATED REGULATION OF DOCOSAHEXAENOIC ACID (DHA) METABOLISM AND NEUROPROTECTION**

Regina Fernandez Fernandez^1^, Sora Kim^2^, Victoria D Diaz^3^, Brian P Hermann^3^, Shelley N Jackson^4^, Jeffrey B Eells^5^, Jessica M Ellis^1^

*^1^ East Carolina University, Physiology, East Carolina Diabetes and Obesity Institute, Greenville, USA*

*^2^ Purdue University, Nutrition Science, West Lafayette, USA*

*^3^ University of Texas at San Antonio, Biology, San Antonio, USA*

*^4^ National Institute on Drug Abuse, Structural Biology Core, Baltimore, USA*

*^5^ East Carolina University, Anatomy and Cell Biology, Greenville, USA*

The omega-3 fatty acid, docosahexaenoic acid (DHA), is enriched in the central nervous system and thought to protect against neuro-logical dysfunction. While dietary DHA supplementation increases DHA levels in peripheral tissues, it often fails to increase brain DHA levels. The indirect relationship between dietary-DHA and brain-DHA highlights the existence of unique mechanisms that allow DHA to be enriched in the brain. To this end, our lab recently discovered an enzyme that is required for neural brain DHA enrichment, long-chain acyl-CoA synthetase 6 (Acsl6), which performs the initial reaction required for cellular fatty acid metabolism. By producing an Acsl6 deficient mouse (Acsl6^−/-^ ), we now have a model with large and specific reductions (35-72%) in brain DHA-containing phospho-lipids. In situ hybridization data demonstrates that Acsl6 is expressed in neurons throughout the brain and MALDI lipid imaging demonstrates that Acsl6^−/-^ brains have reductions in DHA-containing phos-pholipids across the entire brain, an effect that is specific to Acsl6 expression in neurons but not astrocytes. Interestingly, the composition of compensatory replacement lipids was not consistent across the entire brain but was rather differential in a regionally specific manner. The high levels of DHA-containing phospholipids in the cerebellum are largely replaced by arachidonic acid-containing phos-pholipids in the absence of Acsl6, along with evidence of age-related cerebellar astrogliosis, microglia activation, and induction of inflammation-related genes. Behaviorally, Acsl6-/- mice present with disruptions in motor function concomitant to deregulated neurotrans-mitter homeostasis. Our findings suggest that Acsl6 has unique roles for regulating brain lipid metabolism and that Acsl6-mediated DHA deficiency results in motor dysfunction and aging-induced neuro-pathology.

A46

**DIFFERENTIAL ASSOCIATIONS BETWEEN LINOLEIC ACID-DERIVED SOLUBLE EPOXIDE HYDROLASE OXYLIPINS AND BDNF IN DIABETES AND DEPRESSION**

Natasha Anita^1,2,3^, Di Yu^1,2^, Chelsi Major-Orfao^2,3^, Michelle Nguyen^1,2,3^, Sophie Wong^1,2,3^, Theresa Pedersen^4^, Ameer Taha^4^, Walter Swardfager^1,2,3^

*^1^ University of Toronto, Pharmacology & Toxicology, Toronto, Canada*

*^2^ Sunnybrook Research Institute, Hurvitz Brain Sciences, Toronto, Canada*

*^3^ University Health Network, Toronto Rehabilitation Institute, Toronto, Canada*

*^4^ University of California, Food Science and Technology, Davis, USA*

Type 2 diabetes mellitus (T2DM) is associated with increased depression. Dietary fatty acids can be converted by cytochrome P450 enzymes (CYP450s) into neuroprotective epoxides; however their beneficial effects are limited when metabolized by soluble epoxide hydrolase (sEH) into inactive or cytotoxic diols. We found higher serum diol concentrations in T2DM patients with depression, and the linoleic acid-derived 12,13 diol/epoxide ratio, a proxy measure of sEH flux, was associated with depression severity. Relevant mechanisms are not fully understood; however, sEH has been related to brain derived neurotrophic factor (BDNF) in animals. Here, we examine the serum 12,13 diol/epoxide ratio and BDNF concentrations in T2DM patients with and without depression. Individuals with T2DM (glycosylated hemoglobin [HbA1c] above 6.4 %, impaired fasting glucose or impaired glucose tolerance) were recruited and diagnosed with current depression using the Structured Clinical Interview for DSM-5, Research Version. Unesterified oxylipins were extracted from fasting serum through solid phase extraction and quantified by ultra-high-performance liquid chromatography tandem mass spectrometry. Serum BDNF was measured by enzyme-linked immunosorbent assay. Among 89 participants (mean age 62.6 ±9.7, 56 % women, mean HbA1c 7.4 ±1.2%), 24% were depressed (8 men, 13 women). In an analysis of covariance including age, sex, body mass index, glycemic control (HbA1c), depression, and antidepressant use, the 12,13 diol/epoxide ratio was associated with BDNF (F=2.674, p=0.012) but this relationship was specific to those without depression (depression×12,13 ratio interaction F=4.586, p=0.035). These findings suggest a relationship between sEH activity and circulating BDNF concentrations, that was disrupted in depressed people with T2DM, which may help to understand pathophysiological changes in depression.

A47

**REGULATION OF SODIUM-DEPENDENT GLUTAMATE UPTAKE BY PALMITOYLATION**

Alain Guillem Del Angel, Elizabeth Krizman, Michael Robinson

*Children's Hospital of Philadelphia, Pediatrics, Philadelphia, USA*

Glutamate is the main excitatory amino acid neurotransmitter in the central nervous system, it is required for different cognitive processes such as learning and memory, but if the concentration of glutamate is not kept low, it would induce neuronal death by excitotoxicity. Sodium-dependent glutamate uptake is essential to regulate glutamate concentration. The astroglial glutamate transporters glutamate transporter 1 (GLT-1) and glutamate aspartate transporter (GLAST) mediate the majority of forebrain and cerebellum glutamate uptake, respectively. These transporters are regulated by both transcriptional and post-translational processes. There is evidence that glutamate uptake is regulated in the plasma membrane (Murohy-Royal et al 2015). There is evidence that GLT-1 glutamate uptake is regulated by palmitoylation (Huang et al, 2010). The goal of this study was to explore this regulation. Palmitoylation is generally thought to be a reversible post-translational modification that regulates the activity of several different membrane proteins. We used crude synaptosomes prepared from cortex or cerebellum to determine if an inhibitor of palymitolylation (2-bromopalmitate, 2-BP) affects Na^+^-dependent glutamate uptake. As was reported earlier, we found that 2-BP (50 µM) inhibited uptake after 30min to 63±4% of control (N=14, p<0.001) in cortical synaptosomes. Unlike that found earlier, we also found that 2-BP reduced cerebellar uptake to 63±9% of control (N=8, p<0.005), but the methods for uptake were somewhat different. These effects were concentration-dependent (n=4). The effect of 2- BP was blocked by an inhibitor (PalmB) of depalmitotoylating enzymes (APT1 &2). These data are consistent with the hypothesis that both GLT-1 and GLAST activity are dependent upon palmito-ylation. We also found that 2-BP has an effect on cortical glutamate uptake even when the synaptosomes are not pre-incubated (to 74±7% of control, n=7, p <0.05), consistent with the notion that there is a pool of non-palmitoylated GLT-1 in vivo. Studies are underway to determine if these effects are associated with changes in palmito-ylated GLT-1 and GLAST.

A48

**CNS-PENETRATING THYROID HORMONE AGONISTS LOWER VERY LONG CHAIN FATTY ACIDS IN A MOUSE MODEL OF X-LINKED ADRENOLEUKODYSTROPHY**

Meredith Hartley^1,2^, Mitra Shokat^1^, Margaret DeBell^1^, Tania Banerji^1^, Lisa Kirkemo^1^, Thomas Scanlan^1^

*^1^ Oregon Health & Science University, Physiology and Pharmacology, Portland, USA*

*^2^ University of Kansas, Chemistry, Lawrence, USA*

X-linked adrenoleukodystrophy (X-ALD) is a rare, genetic disease in which increased very long chain fatty acids (VLCFAs, C22-C26) cause CNS demyelination and axonal degeneration, leading to severe neurological deficits. The elevation in VLFCAs is caused by mutations in *ABCD1*, which encodes a peroxisomal transporter responsible for the uptake of VLCFAs for degradation. Sobetirome, a potent thyroid hormone agonist, has been shown to lower VLCFA levels in the periphery and CNS of *Abcd1* knockout (KO) mice. In this study, we evaluated two pharmacological strategies for enhancing the effects of thyromimetics. First, we tested a CNS-selective prodrug of sobetirome, which significantly lowered total C26:0/C22:0 and C26:0-lysophosphatidylcholine in the brain and spinal cord of *Abcd1* KO mice. We also demonstrated that the pro-drug was better tolerated than the parent drug due to lower peripheral exposure. Second, we co-administered thyroid hormone with sobetirome to correct for thyromimetic-induced suppression of the hypothalamic-pituitary-thyroid axis. Addition of thyroid hormone enhanced VLCFA lowering in the periphery compared to sobetirome alone, but did not produce greater lowering in the CNS. This result suggested that the extent of lowering in the CNS was limited by a mechanistic threshold related to slow turnover kinetics. In conclusion, our data demonstrate that CNS-penetrating thyromimetics improve dosing tolerance and correct the lipid abnormality associated with X-ALD in peripheral organs, brain, and spinal cord.

A49

**HIV-1 TAT PROTEIN PROMOTES NEUROENDOCRINE DYSFUNCTION CONCURRENT WITH POTENTIATION OF OXYCODONE PSYCHOMOTOR EFFECTS IN FEMALE MICE**

Salahuddin Mohammed^1^, Fakhri Mahdi^1^, Jason Paris^1,2^

*^1^ University of Mississippi, Department of BioMolecular Sciences/Pharmacology Division, Oxford, USA*

*^2^ Research Institute of Pharmaceutical Sciences, University of Mississippi, Oxford, USA*

**INTRODUCTION** Human immunodeficiency virus (HIV) is associated with neuroendocrine dysfunction which may contribute to comorbid stress-sensitive disorders. The hypothalamic-pituitary-adrenal (HPA) and -gonadal (HPG) axes are perturbed in up to 50% of HIV patients, but the mechanisms are not known, but we find that transgenic expression of Tat protein recapitulates the clinical phenotype in male mice.

**PURPOSE** We hypothesized that HPA and/or HPG dysregulation contributes to Tat-mediated interactions with oxycodone, a clinically-used opioid often prescribed to HIV patients.

**METHODS** Mice that expressed the Tat_1-86_ protein [Tat(+) mice] or their control counterparts that did not [Tat(-) control mice] were exposed to forced swim stress (or not) and behaviorally-assessed for motor behavior in the open field task. Mice werepretreated with vehicle, RU-486, or antalarmin to block glucocorticoid receptors (GR) or CRF receptors (CRF-R), respectively and some mice were ovariectomized. Circulating corticosterone was assessed via enzyme-linked immunosorbent assay.

**RESULTS** In transgenic female mice, HIV Tat elevated corticosterone levels recapitulating the clinical HIV phenotype. We have found males to additionally express adrenal insufficiency in response to Tat exposure, however, this was not observed in females. When challenged with oxycodone, Tat-exposed females demonstrated a potentiated psychomotor response that was not attenuated by GR or CRF-R inhibition. However, ovariectomizing females demonstrated elevated hypothalamic allopregnanolone levels concurrent with significant attenuation of the psychomotor response revealing gonadal hormones for Tat-mediated potentiation of oxycodone. Irrespective of genotype, either diestrous cycle phase or exposure to oxycodone showed a significant increase in hypo-thalamic allopregnanolone in non-stressed or stressed paradigm which may indicate a central adaptive response to stress.

**CONCLUSIONS** Together, these effects support the notion that Tat exposure dysregulates the HPA and HPG axes, potentially raising vulnerability to stress-related substance use disorders in females. HPG activation may be necessary for the combined oxycodone-Tat interaction indicating the importance of maintained HPG feedback in this vulnerable population.

A50

**A NOVEL APPROACH FOR ELECTROCHEMICAL DETECTION OF OPIOID PEPTIDES**

Sineadh Conway^1,2^, Loc Thang^1,2^, Graydon Gereau^1,2^, John Cirrito^1^, Carla Yuede^1^, Ream Al-Hasani^2,1^

*^1^ Washington University in St. Louis, Anesthesiology, St. Louis, USA*

*^2^ St. Louis College of Pharmacy, CCP, St. Louis, USA*

The endogenous opioid peptide systems are critical for analgesia, reward processing, and negative affect, however research on their *in vivo* function in modulation of these behaviors has been challenging due to an inability to reliably detect dynamic changes in opioid peptides. There is little to no data directly measuring dynamic *in vivo* changes of endogenous opioids, in particular dynorphin. The ability to correlate changes in brain neuropeptide levels during circuit or behavioral manipulations will exponentially evolve our understanding of brain processing. Thus, our work aims to develop innovative approaches for rapid and sensitive detection of opioid peptide release *in vivo*. We developed microimmunoelectrodes (MIEs) for electro-chemical detection of opioid peptides. Because all opioid peptides contain an electroactive tyrosine residue as part of their N-terminus sequence, square-wave voltammetry can be used to measure their presence. Briefly, a voltage is applied to the electrode to cause oxidation of the tyrosine residue, which is detected as current. To provide specificity to these voltammetric measurements, the carbon fiber surface of the MIE is coated with antibody selective to the opioid peptide of interest and any remaining binding sites are blocked with bovine serum albumin. To test the sensitivity of the MIEs, electrodes are immersed in solutions containing different concentrations of opioid peptides, and oxidative current is measured. We show that dynorphin antibody-coated electrodes are sensitive to increasing concentrations of dynorphin in the fmol range. To confirm specificity, oxidative current is also measured to tyrosine and other opioid peptides. The dynorphin antibody-coated MIEs are sensitive to metenkephalin and detect a small oxidative current to tyrosine, and we are working to minimize these non-specific electrochemical signals. Future work aims to demonstrate the utility of these MIEs both *in vitro* via brain slice preparation and *in vivo* for real-time, rapid detection of endogenous opioid peptide release in awake and behaving animals.

A51

**IN VIVO DETECTION OF ENDOGENOUS OPIOID PEPTIDES DURING OPIOID WITHDRAWAL**

Marwa Mikati^1,2^, Kia Barclay^2^, Petra Erdmann-Gilmore^1^, Robert Sprung^1^, Reid Townsend^1^, Ream Al-Hasani^2,1^

*^1^ Washington University in St. Louis, School of Medicine, St. Louis, USA*

*^2^ St. Louis College of Pharmacy, CCP, St. Louis, USA*

Opioids are the primary treatment to relieve pain, however, their chronic use can lead to severe addiction. The withdrawal syndrome associated with abstinence from opioids often results in relapse, sometimes fatal and prevents long-term abstinence. Studies have shown that increased expression of dynorphin mRNA, the endogenous kappa opioid receptor ligand, increases during withdrawal in the nucleus accumbens. However, there is no reliable method to directly measure *in vivo* changes in opioid peptides. We describe a method coupling microdialysis and nano-liquid chromatography/ mass spectrometry to detect endogenous opioid peptides *in vivo*. The *in vivo* resolution not only advances our ability to reliably measure opioid peptides, but also allows us to correlate dynamic changes with behavioral outputs. We hypothesize that the kappa opioid system modulates the somatic and affective components of withdrawal leading to relapse. We use osmotic mini-pumps to establish a fentanyl dependent mouse model, and measure somatic and anxiety-like symptoms during withdrawal. During the 14 day infusion, mice are monitored for analgesic behavior, using the hot and cold plate tests, and for anxiety behavior using the Open Field Test(OFT), and Elevator Plus Maze(EPM). Additionally, we use DeepLabCut for pose estimation to assess the somatic signs of withdrawal. On day 14 of infusion, mice are implanted with a microdialysis probe in the nucleus accumbens to allow for continuous sample collection. We demonstrate that fentanyl is analgesic at one week, and that this effect is not reversible by naloxone at withdrawal. We do not detect anxiety behavior in OFT during fentanyl infusion, however, naloxone alone significantly decreases open-arm time in EPM. Additionally, the mice undergoing precipitated withdrawal show significantly greater somatic signs of withdrawal specifically grooming and foraging. Finally, we successfully detected met-enkephalin and leu-enkephalin *in vivo* during withdrawal. Our findings introduce a novel method of monitoring opioid peptide levels while simultaneously assessing withdrawal behaviors.

A52

**BIOENERGETIC STRESS DURING ALCOHOL INTOXICATION**

Li Zhang^1^, Zhe Zhang^2^, Tracey Singer^2^, Shinghua Ding^1,2^

*^1^ University of Missouri, Interdisciplinary Neuroscience, Columbia, USA*

*^2^ University of Missouri, Biological Engineering, Columbia, USA*

*^3^ University of Missouri, Dalton Cardiovascular Research Center, Columbia, USA*

Regulation of cellular energy metabolism is important to the central nervous system (CNS), where energy is in high demand. Alcohol in-toxication is a harmful condition induced by excessive consumption of alcohol. Alcohol diffuses through most cell membranes and can be metabolized by most tissues. The brain, particularly the cerebral cortex, is vulnerable to alcohol intoxication. Alcohol intoxication causes impairment of motor function. Nicotinamide adenine dinucleotide (NAD^+^) is known as an important co-enzyme for cellular energy metabolism and NAD^+^ is consumed during alcohol metabolism. Here we treated primary cortical neurons with various concentrations of alcohol, and studied cellular bioenergetic stress during alcohol intoxication using various assays. We found that alcohol caused an increase in neuronal injury and reductions of NAD^+^, NADH, and ATP levels in a dose dependent manner. We also found that alcohol intoxication decreased mitochondrial membrane potential and increased oxidative stress. Moreover, alcohol in-toxication impaired both mitochondrial respiration and glycolytic function and affected glycolysis less than mitochondrial respiration under mitochondrial stress. Finally, supplement of NAD^+^ ameliorated ATP reduction and neuronal death, and improved mitochondrial respiration and glycolytic function after alcohol intoxication. The current study provides new insights into neuronal bioenergetics after alcohol intoxication, and repletion of NAD^+^ and enhancing NAD^+^ biosynthesis might be a potential therapeutic strategy for reducing neuronal degeneration and ameliorating motor impairment caused by alcohol intoxication.

A53

**SYNAPTOJANIN1 DEFICIENCY UPREGULATES BASAL AUTOPHAGOSOME FORMATION IN ASTROCYTES**

Pingyue Pan, Justin Zhu, Asma Rizvi, Xinyu Zhu, Hikari Tanaka, Cheryl Dreyfus

*Rutgers, Robert Wood Johnson medical School, Neuroscience and Cell Biology, Piscataway, USA*

Macroautophagy dysregulation is implicated in multiple neuro-logical disorders, such as Parkinson's disease. While autophagy pathways are heavily researched in heterologous cells and neurons, regulation of autophagy in the astrocyte, the most abundant cell type in the mammalian brain, is less well understood. Missense mutations in the SYNJ1 gene encoding Synaptojanin1 (Synj1), a neuron-enriched lipid phosphatase, have been linked to Parkinsonism with seizures. Our previous study showed that the Synj1 haploinsufficient (Synj1+/-) mouse exhibits age-dependent autophagy impairment in multiple brain regions. Here, we used cultured astrocytes from Synj1-deficient mice to investigate its role in astrocyte autophagy. We report Synj1 is expressed in low levels in astrocytes and represses basal autophagosome formation. We demonstrate using cellular imaging that Synj1-deficient astrocytes exhibit hyperactive auto-phagosome formation, represented by an increase in the size and the number of GFP-LC3 structures. Interestingly, Synj1 deficiency is also associated with an impairment in stress-induced autophagy clearance. We show, for the first time, that the Parkinsonism-associated R839C mutation impacts autophagy in astrocytes. The impact of this mutation on Synj1's phosphatase function resulted in elevated basal autophagosome formation that mimics Synj1 deletion. We found that the membrane expression of the astrocyte-specific glucose transporter GluT-1 was reduced in Synj1-deficient astrocytes. Consistently, AMPK activity was elevated, suggesting altered glucose sensing in Synj1-deficient astrocytes. Expressing exogenous GluT-1 in Synj1-deficient astrocytes reversed the autophagy impairment, supporting a role for Synj1 in regulating astrocyte auto-phagy via disrupting glucose-sensing pathways. Thus, our work suggests a novel mechanism for Synj1-related Parkinsonism involving astrocyte dysfunction.

## Poster Session B

B01

**FINE STRUCTURE OF THE MYELIN AT AXON INITIAL SEGMENT IN SPINAL CORD**

Miki Furusho, Mark Terasaki

*Univ. Connecticut Health, Cell Biology, Farmington, USA*

The neuronal action potential initiates at the axon initial segment (AIS) and is rapidly and efficiently propagated along the myelinated axon by the Nodes of Ranvier. The AIS and Node of Ranvier have a similar high density of channels. While the nodes are specialized for reliable propagation, the AIS is involved in the initiation, and the neuron may have the capability to modify the properties of the AIS in order to fine-tune its excitation.

The fine structure of the myelin at the Node of Ranvier is well characterized but that of the myelin at the AIS is not. This is because it is difficult to locate the AIS of neurons by conventional EM techniques. We used serial sections from an ATUM tape collector to inspect by electron microscopy about 1000 images of 300um x 250um areas of the cervical spinal cord and found 9 AIS.

We compared these AIS with 8 Nodes of Ranvier which had similar axon diameters. We found three major differences. 1) There were two types of myelin structures at the AIS. One was similar to myelin at the Node of Ranvier. The other type had more than one compact myelin around the axon and myelin loops of outside compact myelin contact with the inside myelin sheath. 2) There were mitochondria in the loop at both Node of Ranvier and AIS.

Whereas at the AIS, the average number of mitochondria in the loop was 18.2±2.7, at the Node of Ranvier it was 11.2±2.2. 3) At the Node of Ranvier, there were 1-2 endoplasmic reticulum (ER) tubules in each myelin loop. At the AIS, however, most of the myelin loops contained 2-5 ER tubules.

It seems likely that the paranode at the AIS may have an effect on the initiation of action potentials. Possibly, the structural differences from the Nodes of Ranvier are reflective of the plasticity of this para-node.

B02

**CERTL REDUCES C16 CERAMIDE, AMYLOID-Β LEVELS AND INFLAMMATION IN A MODEL OF ALZHEIMER'S DISEASE**

Simone Crivelli^1,2^, Qian Luo^2^, Caterina Giovagnoni^2^, Jo Stevens^2^, Sandra den Hoedt^3^, Joost Verhaagen^4^, Anna-Lena Scheithauer^5^, Jochen Walter^5^, Monique Mulder^3^, Christopher Exley^6^, Michelle Mielke^7^, Helga De Vries^8^, Kristiaan Wouters^2^, Daniel van den Hove^2^, Dusan Berkes^9^, María-Dolores Ledesma^10^, Mario Losen^2^, Erhard Bieberich^1^, Pilar Martinez^2^

*^1^ University of Kentucky, Physiology, Lexington, USA*

*^2^ Maastricht University, Neuroscience, Maastricht, Netherlands*

*^3^ Erasmus MC, Internal Medicine, Rotterdam, Netherlands*

*^4^ NIH, Neuroscience, Amsterdam, Netherlands*

*^5^ University of Bonn, Membrane Biology and Lipid Biochemistry, Bonn, Germany*

*^6^ Keele University, The Birchall Centre, Staffordshire, UK*

*^7^ Mayo Clinic Rochester, Health science research, Minnesota, USA*

*^8^ VUmc, Molecular Cell Biology and Immunology, Amsterdam, Netherlands*

*^9^ Slovak University of Technology, Organic Chemistry, Bratislava, Slovak Republic*

*^10^ Centro de Biología Molecular Severo Ochoa, Molecular Neuropathology, Madrid, Spain*

Dysregulation of ceramide and sphingomyelin levels have been suggested to contribute to the pathogenesis of Alzheimer's disease (AD). Ceramide transfer proteins (CERTs) are ceramide carriers which are crucial for ceramide and sphingomyelin balance in cells. Extracellular forms of CERTs co-localize with amyloid-β (Aβ) plaques in AD brains. To date, the significance of these observations for the pathophysiology of AD remains uncertain. A plasmid expressing CERT_L_, the long isoform of CERTs, and the recombinant CERT_L_ protein were used to study the interaction of CERT_L_ with amyloid precursor protein (APP) and Aβ *in vitro*. CERT_L_ was overexpressed in neurons by adeno associated virus (AAV) in a mouse model of familial AD (5xFAD). Twelve weeks after transduction, brains were investigated for sphingolipid levels by mass spectrometry, plaques and neuroinflammation by immuno-histochemistry, gene expression and/or immunoassay. We report that CERT_L_, binds to APP, modifies Aβ aggregation and reduces Aβ neurotoxicity *in vitro*. Furthermore, we show that intracortical injection of AAV, mediating the expression of CERT_L_, decreases levels of ceramide d18:1/16:0, Aβ formation and microglia pro-inflammatory phenotype in the cortex of 5xFAD. Our results demonstrate a crucial role of CERT_L_ in regulating ceramide levels, amyloid plaque formation and neuroinflammation.

B03

**SUB-CHRONIC HYPERGLYCEMIA INCREASES OXIDATIVE STRESS LEVELS AND IMPAIRED SPATIAL MEMORY IN CD-1 MICE**

Diana Del moral^1^, César Mendoza-Calles^2^, Claudia Juárez-Portilla^3^, Mónica Flores-Muñoz^4^, Óscar López-Franco^4^, Gabriel Roldán-Roldán^5^, Rossana Zepeda^3^

*^1^ Programa de Doctorado en Ciencias Biomédicas, NA, Xalapa, México*

*^2^ Programa de Maestría en Ciencias de la Salud, NA, Xalapa, México*

*^3^ Centro de Investigaciones Biomédicas, Universidad Veracruzana, Laboratorio de Biomedicina Integral y Salud, Xalapa, México*

*^4^ Instituto de Ciencias de la Salud, Universidad Veracruzana, Laboratorio de Medicina Traslacional, Xalapa, México*

*^5^ Facultad de Medicina, Universidad Nacional Autónoma de Mexico, Fisiología, México, México*

Diabetes mellitus (DM) is a disease characterized by disrupted metabolism and high glucose levels, which complications include cognitive disfunction. In diabetic patients, functional and structural abnormalities have been shown by brain magnetic resonance imaging. Additionally, several studies have implied free radicals (FR) in the cognitive loss caused by DM, since high consumption of O2 and glucose in the CNS produces FR, which in turn, makes CNS vulnerable to the harmful effects of the reactive oxygen species. Various mechanisms of cognitive damage induced by hyperglycemia have been proposed. There is a wide discrepancy in the reported results, mainly because of methodological differences among studies (e.g., the hyperglycemic model, the time-lapse between hyper-glycemia induction, and the learning paradigms). Therefore, this study aimed to evaluate the effects of sub-chronic hyperglycemia on spatial memory paradigms and its correlation with oxidative stress markers. CD-1 mice were administered with STZ and fifteen days later, learning and memory capabilities were evaluated the in two explicit memory tasks: 1) eight-arm radial maze, or 2) buried food location test. The increase of oxidative stress was evaluated by the lipoperoxidation and antioxidant enzyme activity of superoxide dismutase and catalase in the hippocampus. Our results showed that DM is associated with increase lipid peroxidation and a significant decrease of antioxidant activity of enzymes. We conclude that hyper-glycemia induces FR, due to a decline of antioxidant enzymatic activity in the hippocampus, this may contribute to the cognitive impairment in diabetic mice. However, biochemical and molecular studies are needed to understand the mechanisms underlying these findings.

B04

**SYNTHESIS AND BIOLOGICAL EVALUATION OF ANALOGUES OF LANTHIONINE KETIMINE AS SMALL MOLECULE TREATMENTS FOR NEUROLOGICAL DISORDERS**

Travis Denton, Dunxin Shen, Elizabeth Everson, Jing Wang, Tristan Hilton

*Washington State University, College of Pharmacy and Pharmaceutical Sciences, Spokane, USA*

Lanthionine ketimine (LK) is a natural amino acid metabolite found at low concentrations in mammalian brain tissue. LK, and its synthetic ethyl ester derivative, LKE, have potent neuroprotective, neurotrophic and anti-neuroinflammatory properties and have been shown to increase autophagy in neurons and glia. LKE shows benefits in diverse preclinical models of Alzheimer's disease, amyo-trophic lateral sclerosis (ALS), glioma, multiple sclerosis, traumatic brain injury and stroke. Using LK and LKE as lead compounds, we have recently synthesized a series of 17 phosphonate or non-phos-phonate LK analogues. The synthesis incorporates multiple reactions in “one-pot.” The structures were confirmed by ^1^H, ^13^C and ^31^P NMR and liquid chromatography tandem UV spectrophotometry high-resolution mass spectrometry (UPLC/UV/HRMS). The new analogues were assayed for cytotoxicity, autophagy stimulation and the ability of the compounds to protect from hydrogen peroxide derived oxidative stress in a SH-SY5Y neuroblastoma cell line. All of the new compounds show low toxicity, stimulate autophagy and provide various levels of protection form oxidative stress indicating these compounds as potential drug leads for the treatment of neuro-degenerative disorders related to dysfunctional autophagy and or oxi-dative stress.

B05

**MODAFINIL MODULATES THE GLUTAMATE/GLUTAMINE SHUTTLE IN CULTURED BERGMANN GLIA CELLS**

Laura Mendez, Elizabeth Bejarano-Pérez, Luis Cid, Luisa Hernández-Kelly, Arturo Ortega

*CINVESTAV, Toxicology, CDMX, Mexico*

Glutamate is the major excitatory transmitter in the Central Nervous System of vertebrates and exerts its actions through the activation of specific membrane receptors and transporters. Over-stimulation of glutamatergic receptors results in neuronal death, a phenomenon known as excitotoxicity. Therefore, extracellular glu-tamate levels have to be tightly regulated through a family of high-affinity transporters expressed in neurons and glial cells. Most of the uptake process occurs in the glial compartment *via* a biochemical coupling known as glutamate/glutamine shuttle that is involved in the turnover of this excitatory neurotransmitter. Among the variety of freely accessible Central Nervous System stimulants, modafinil is widely used as a wakefulness agent although it has also been recommended for the treatment of excessive daytime sleepiness, fatigue, and impaired cognition. Due to these properties, nowadays it has become a popular drug among youngsters. Despite the fact that the mechanism of action of this drug is far from being understood, it increases glutamate extracellular levels. In order to characterize the involvement of glutamate/glutamine shuttle in the effects of modafinil, we used the well-established model of chick cerebellar Bergmann glia primary cultures. Acute treatment with modafinil results in a significant increase in [^3^H]-D-Aspartate uptake, apparently related to an augmentation of the affinity of the transport.

An expected decrease in [^3^H]-Glutamine uptake, result from a change in the affinity of the transporters, was also found. Long-term exposure to the stimulant is likely to affect the protein synthesis process. Our results suggest that glial cells are targets of modafinil through the modulation of the glutamate turnover process.

B06

**EFFECT OF THE EXPOSURE TO NPS-SIO2 ON EXTRACELLULAR-REGULATED KINASES 1/2 PHOSPHORYLATION IN HUMAN RETINA RADIAL GLIAL CELLS**

Fredy Sanchez, Ada Rodriguez, Arturo Ortega

*Center for research and advanced studies of the National Polytechnic Institute, Toxicology, Ciudad de México, Mexico*

Effect of the exposure to NPS-SiO2 on extracellular-regulated kinases 1/2 phosphorylation in human retina radial glial cells Sanchez Cano Fredy, Rodriguez Campuzano Ada G. and Ortega Arturo Laboratorio de Neurotoxiclogia, Departamento de Toxicologia, Cinvestav-IPN, Apartado Postal 14-740, Mexico D.F.m Mexico. the use of silica nanoparticles is increasingly frequent due to its applications in various biotechnological areas. the neurotoxicity of these particles has been based mainly, but not exclusively, on epide-miological studies in which exposure to NPS is associated with degenerative diseases such as Alzheimeŕs disease and some others in which there is a neuronal dysfunction. Taking into consideration that glial cells participate in the regulation of neuronal communication, in this work we evaluated the effect of NPS-SiO2 exposure on the function and signaling of glutamate plasma membrane transporters in radial glial cells from the human retina (MIO-M1 cells). Confluent monolayers were exposed to NPS-SiO2 at different concentrations (0.4 and 44.8 micrograms/ml for 3, 6, and 12 hours of exposure). No significant reduction os mitochondrial activity was found under these conditions. Therefore, the cells were exposed to different concentrations od silica NPS (0.4, 4.8, 8, 15, and 20 micrograms/ml for different time periods (15, 30, 60, and 90 minutes). The phosphorylation pattern of extracellular-regulated kinases (ERK 1 and 2) was determined. Phospho ERK 1/2 were evident at 30 minutes of the treatment with NPS at both concentrations (0.4, and 4.8 micrograms/ ml). Our results suggest that exposure to silica nanoparticles alters the proteome od glial cell by modulating the key signaling pathways in the transcriptional and translational control of gene expresión.

B07

**HUMAN BRAINSTEM ATLAS COMBINING IMMUNOCYTOCHEMISTRY AND MRI: TOWARDS A PRECISE DESCRIPTION OF THE PPN TARGETED BY DBS**

Vincent Coulombe^1^, Stephan Saikali^3^, Laurent Goetz^4^, Mohamad A. Takech^2^, Éric Philippe^2^, André Parent^1,2^, Martin Parent^1,2^

*^1^ CERVO Brain Research Center, Psychiatry and Neuroscience, Québec, Canada*

*^2^ Laval University, Surgery and Anatomy, Québec, Canada*

*^3^ Hôpital de l'Enfant-Jésus, Pathology, Québec, Canada*

*^4^ Hôpital Fondation Adolphe de Rothschild, Neurosurgery, Paris, France*

OBJECTIVES: Located in the brainstem, the pedunculopontine nucleus (PPN) is known to play important roles in numerous functions of the brain such as nociception and sleep. Being also involved in locomotion, this nucleus has recently generated a great interest since it can be targeted by deep brain stimulation (DBS) to alleviate postural instability and gait disturbances that affect many parkinsonian patients. Studies that assessed the exact anatomical position of the PPN remain controversial. This project aims to produce a detailed human brainstem atlas allowing a precise localization of the PPN, and to characterize the neurochemical content of the cells that compose this brainstem nucleus. METHODS: To achieve our goal, we performed a high-resolution post-mortem magnetic resonance imaging (MRI) scan of a human brainstem followed by histological staining. In addition, other sections taken through the PPN of human and cynomolgus monkey (*Macaca fascicularis*) brainstems were stained for choline acetyltransferase (ChAT) and for nicotinamide adenine dinucleotide-diaphorase (NADPH-δ). RESULTS: The MRI, combined with 45 anatomical plates presenting equally-spaced histological sections stained for Cresyl Violet and Luxol Fast Blue, allowed us to precisely localize the PPN at the ponto-mesencephalic junction. Our stereological studies have led us to estimate at 58 100 the number of ChAT+ neurons of the human PPN, contained in a volume of 145 mm^3^. Our 3D reconstructions indicate a significant degree of overlap between neurons of the PPN, laterodorsal tegmental nucleus, locus coeruleus and substantia nigra. CONCLUSIONS: Overall, our work provides a better anatomical and neurochemical description of the PPN and could help in a better placement of DBS electrodes, increasing the clinical outcomes for Parkinson's disease patients.

B08

**FLUORIDE EXPOSURE DISRUPTS GLUTAMINE TRANSPORT IN RETINAL MÜLLER GLIAL CELLS**

Ana García-López, Arturo Ortega

*Centro de Investigación y de Estudios Avanzados del Instituto Politécnico Nacional, Toxicología, Mexico, Mexico*

Glutamine is the most abundant extracellular amino acid in the Central Nervous System. It is the main precursor of the main excitatory and inhibitory neurotransmitters: glutamate and GABA, respectively. The GABA/glutamate/glutamine shuttle provides neurons with glutamine, which is transported through a family of sodium-dependent neutral amino acid transporters of the *slc38a* gene family. System N glutamine transporters, SNAT3 and SNAT5 are expressed in glial cells and supply neurons with glutamine in a Na^+^-dependent manner. Interestingly enough Li^+^ can replace Na^+^ albeit the efficiency of the transport diminishes. Fluoride, an environmental pollutant present in dental products, food, pesticides and water, severely impairs cognitive functions in exposed population, likely through a disruption of glutamatergic and/or GABAergic neurotrans-mission. Taking into consideration the pivotal role of glial SNATs in the recycling of these amino acid transmitters, we evaluated the effect of F^−^ acute exposure in the functional expression of SNAT3 and SNAT5 in a human retina Müller glia cell line. A roughly 20% reduction in [^3^H]glutamine uptake was present upon a 500 µM F^−^ treatment for 30 min. Michaelis-Menten analysis revealed a reduction in Vmax. As expected, Immunochemical experiments demonstrated a reduction of both SNAT3 and 5 protein levels in the plasma membrane of F^−^ exposed cultures. These results support the idea of glia cells as an important target of neurotoxicants.

B09

**ASTROCYTE-MEDIATED SYNAPSE FORMATION IS UNDER CONTROL OF NOGO-A SIGNALING**

Vinicius Coutinho Costa^1^, Sheila Espírito-Santo Araújo^1,3^, Romulo Dezonne^1^, Joice Stipursky^1^, Alexandre Rodrigues^4^, Carolina Batista^1^, Roberto Paes de Carvalho^4^, Babette Fuss^2^, Flávia Carvalho Alcantara Gomes^1^

*^1^ Universidade Federal do Rio de Janeiro, Instituto de Ciências Biomédicas, Rio de Janeiro, Brazil*

*^2^ Virginia Commonwealth University, Anatomy And Neurobiology, Richmond, USA*

*^3^ Universidade Estadual do Norte Fluminense Darcy Ribeiro, Biologia Tecidual, Campos dos Goytacazes, Brazil*

*^4^ Universidade Federal Fluminense, Departamento de Neurobiologia, Niterói, Brazil*

Synapse formation is a hallmark of central nervous system development and function, being regulated by a cluster of cellular, environmental and molecular cues. Among those, astrocytes are well known in contributing to synaptogenesis, providing both contact-dependent signals and soluble factors, such as Hevin and SPARC. The myelination process and its associated proteins, such as Nogo-A, are remarkable in acting as inhibitory cues on synapse formation and plasticity in the CNS. Although astrocytes express receptors for Nogo-A, it is still unknown whether Nogo-A signaling modulates astrocyte-mediated synapse formation. Therefore, here we investigated the effect of Nogo-A on synaptic regulation modulated by astrocytes, both in vitro and in a murine model of cuprizone induced demyelination. Our data in vitro show cortical astrocytes respond to Nogo-A through RhoA pathway activation, exhibiting stress fiber formation and decreased ramified morphology. These alterations are associated with decreased mRNA levels of synaptogenesis-associated genes Hevin, glypican-4, TGF-β1 and BDNF, and decreased and increased protein levels of Hevin and SPARC, respectively. Reduction of production and release of pro-synaptogenic soluble factors was confirmed by treatment of neuronal cultures with conditioned medium from astrocytes exposed to Nogo-A, which led to a decrease in the number of mature synapses in vitro. Those results were corroborated by data from our demyelination model. Under these conditions, reduced immunostaining for Nogo-A in the visual cortex was accompanied by higher levels of Hevin expression in astrocytes and increase in excitatory synapse density. Therefore, we suggest that interaction between astrocytes and Nogo-A is biologically relevant to synapse function, and may act as potential therapeutic targets in demyelinating diseases.

B10

**VULNERABILITIES OF THE IRON DEFICIENT BRAIN TO ENVIRONMENTAL TOXICANTS**

Janine Cubello^1^, Margot Mayer-Proschel^2^

*^1^ University of Rochester, Environmental Medicine, Rochester, USA*

*^2^ University of Rochester, Biomedical Genetics, Rochester, USA*

Iron deficiency (ID) is the most prevalent micronutrient deficiency in the world. Pregnant women are particularly susceptible to ID due to enhanced iron (Fe) requirements needed for fetal development. Lead (Pb), unlike Fe, is a nonessential divalent metal previously used in commercial products until it was linked to various pathologies and developmental deficits. Despite regulatory efforts, Pb is still a ubiquitous environmental toxin. The separate impacts of ID and Pb exposure on the developing brain have been studied extensively; however, there is a paucity of research considering co-exposure. Greater understanding of the neurodevelopmental outcome of co-exposure is essential as ID and Pb both impact individuals of lower socioeconomic status and have been associated with similar neurodevelopmental and behavioral deficits. Additionally, whether in the form of a deficiency of Fe or a presence of Pb, both Fe and Pb are divalent metals that can perturb the homeostasis of other essential metals. Some essential metals have been reported to respond to Fe levels and of particular interest to our lab, the contributions of regional disturbances in these other essential metals to later neuro-logical impairments observed under ID, Pb, or co-exposure have yet to be resolved. Using a dietary mouse model of ID combined with life-long Pb exposure, we conducted Inductively Coupled Plasma Mass Spectrometry (ICP-MS) analyses of various essential metals in the brain tissue of early postnatal mice. As early as postnatal day 6 (PND6), mild tissue ID significantly enhanced Pb accumulation in both the cerebral cortex and hippocampus and our exposures further led to region-specific changes in overall metal homeostasis. Interestingly, the accumulation of Pb in ID brain tissue was mirrored by accumulation of Pb in astrocytes *in vitro*, cells that have been described to play a role in metal handling and buffering. We are currently investigating the short and long-term cellular consequences of these profound disruptions in metal homeostasis. (Supported by R01HD094563)

B11

**SULFATIDE IS ESSENTIAL FOR NODAL STABILIZATION IN THE MATURE CNS**

Elizabeth Dustin^1,2,3^, Juan Palavicini^4^, Xianlin Han^4^, Rory McQuiston^1^, Jeffrey L Dupree^1,2^

*^1^ Virginia Commonwealth University, Anatomy and Neurobiology, Richmond, USA*

*^2^ HH McGuire VAMC, Research Service, Richmond, USA*

*^3^ Virginia Commonwealth University, Neuroscience Curriculum, Richmond, USA*

*^4^ UT Health San Antonio, Medicine, San Antonio, USA*

3-O-Sulfogalactosylceramide (sulfatide) is a sphingolipid that constitutes up to 4% of total myelin lipids in the central nervous system. Previously, our lab ultrastructurally characterized a mouse with a constitutively disrupted cerebroside sulfotransferase (CST) gene. CST catalyzes the final step in the production of sulfatide. Consequentially, the constitutive CST “knockout” mice are incapable of synthesizing sulfatide. Using this mouse, our lab has shown that sulfatide is required for proper establishment and maintenance the axoglial junctions and that provide stability to the nodal domains. In addition, we reported that sulfatide is involved in oligodendrocyte differentiation, proliferation, and may play a role in protein compartmentalization within the myelin sheath. Interestingly, some ultrastructural pathologies that we reported in the CST KO mice are consistent with structural abnormalities observed in Multiple Sclerosis (MS). Moreover, sulfatide has been reported to be reduced in regions of normal appearing white matter (NAWM) of MS patients. Reduction of this lipid in regions of NAWM suggests that sulfatide depletion is independent of demyelination and may be a driving force of disease pathology, not merely a consequence of disease progression. However, since MS is typically diagnosed in young adults, the constitutive CST KO mouse has limited clinical relevance since these mice develop in the absence of the lipid. In order to generate a more clinically relevant model, our lab has generated a “floxed” CST mouse, which provides both temporal and cell specific ablation of the CST gene. Using this mouse, mated against the PLP-creERT mouse we are investigating the structural and functional consequence that adult onset sulfatide depletion has on myelin sheath and axonal domain integrity and on oligodendrocyte function. Our data suggests that sulfatide depletion in the adult CNS causes nodal pathology and subsequent functional deficits to myelinated axons.

B12

**ASSESSING MITOCHONDRIAL FEATURES ACROSS MICROGLIAL LIFESPAN**

Katherine Espinoza, Ari Schaler, Lindsay De Biase

*UCLA, Physiology, Los Angeles, USA*

Microglia play essential roles in proper CNS development, tissue homeostasis, and responses to disease and injury. While performing such diverse functional roles, microglia show wide variation of key attributes such as morphology, and phagocytic behaviors across age, region, and disease/injury context. The mechanisms that regulate many of these dynamic changes in microglia are not well understood. In macrophages – peripheral immune cells like microglia – mito-chondria are central regulators of key cellular attributes like phagocytosis. Indeed, recent studies indicate that mitochondria carry out numerous intracellular signaling functions in addition to ATP-production and may have distinct functional roles in different cell types. Surprisingly, little is known about microglial mitochondria, including whether they can act as central regulators of key microglial attributes. In this study, we sought to map the mitochondrial landscape within microglia and to understand how mitochondrial status relates to differences in microglial attributes across brain region, development, and aging. Using a mouse that we generated with GFP targeted to the outer mitochondrial membrane in microglia, we analyzed microglial mitochondria in the ventral tegmental area (VTA) and nucleus accumbens (NAc), where microglia show distinct cellular phenotype and distinct expression of mitochondrial genes. Using confocal microscopy in fixed tissue and 3D reconstruction in Imaris, we analyzed microglial mitochondrial abundance, length, and formation of branched networks in these mice during development, adulthood and aging in VTA and NAc. We found that mitochondria in young adult mice make up around 4% and 5.5% of the total micro-glial volume in NAc and VTA, respectively. Qualitatively, microglial mitochondria seem less abundant and have less branched networks compared to macrophage mitochondria. We also observed prominent distinctions in microglial mitochondrial morphology between development, adulthood, and aging. Using FACS-based analyses and dyes sensitive to mitochondrial membrane potential, we observed regional and age-related differences in microglial mitochondrial membrane potential. Our results provide a previously unavailable map of microglial mitochondrial status and lay the foundation for understanding how these organelles regulate microglial function in diverse physiological and pathological contexts.

B13

**ANALOGS OF GELATINASE INHIBITOR ATTENUATE BRAIN DAMAGE AND IMPROVE SENSORIMOTOR FUNCTIONS IN MICE WITH CORTICAL IMPACT INJURY**

Shanyan Chen^1^, Zhenzhou Chen^1^, Jiankun Cui^1,2^, Heather Siedhoff^1^, Runting Li^1^, Wei Song^3^, Ashley Balderrama^1^, Shahriar Mobashery^3^, Mayland Chang^3^, Zezong Gu^1,2^

*^1^ University of Missouri-Columbia, Pathology &Anatomical Science, Columbia, USA*

*^2^ Truman VA Hospital, Research Service, Columbia, USA*

*^3^ University of Notre Dame, Chemistry & Biochemistry, Notre Dame, USA*

Traumatic brain injury (TBI) is a prevalent condition affecting approximately 1.7 million individuals annually in the United States.SB-3CT, a mechanism-based selective gelatinase inhibitor provides neuroprotection by ameliorating secondary injury after TBI. SB-3CT is poorly water-soluble and is metabolized to *p*-OH SB-3CT, a more potent gelatinase inhibitor than SB-3CT. The water-soluble *p*-NH-arginine prodrug (ND-478) is hydrolyzed to the active MMP-9 inhibitor *p*-amino SB-3CT (ND-322), which is *N*-acetylated to ND- 364; only the latter reaches therapeutic concentrations in the brain. In this study, we performed an electromagnetic controlled cortical impact TBI model in mice, and subsequently treated these animals with ND-478, ND-322 or ND-364 using different routes of administration. We found that mice administered with ND-364 intra-peritoneally (*i.p.*) at an early stage of post-injury, followed by subsequent subcutaneous (*s.c*.) doses of ND-364 inhibited MMP-9 gelatinolytic activity, reduced brain lesion volumes, and improved mouse complex motor coordination and sensorimotor function significantly. Treatment with other strategies only have showed partial functional improvement. The recently developed water soluble *p*- methylamino analog ((*R*)-ND-336) is>20-fold more potent MMP-9 inhibitor than SB-3CT. (*R*)-ND-336 was intravenously (*i.v.*) injected 2 hours post-injury and subsequent daily *s.c.* doses. We found that (*R*)-ND-336 effectively improved sensorimotor function short-term after TBI. The continuing studies are examining brain lesion, motor coordination defect, stress-induced welfare loss not only in short-term, but also long-term after TBI. These findings indicate that gelatinase inhibitor analogs of SB-3CT hold considerable promise as potential treatments of gelatinase-dependent injury after TBI. Support: AHA Grant 09NSDG2260983; Dana Foundation; MU PAS Research Fund (Z.G.); Indiana Traumatic Spinal Cord & Brain Injury Research Program; NFL Charities; ADA Pathway to Stop Diabetes Grant 1-15-ACN-06 (M.C.).

B14

**INTRANASAL LEUKEMIA INHIBITORY FACTOR AMELIORATES GLIOSIS AND IMPROVES MOTOR DYSFUNCTION AFTER PEDIATRIC TRAUMATIC BRAIN INJURY**

Veera D'Mello, Malini Subramaniam, Vincent Dodson, Aditya Paul Bhalla, Steven W. Levison

*Rutgers University, Pharmacology, Physiology and Neuroscience, Newark, USA*

Leukemia Inhibitory Factor (LIF) is an injury-induced cytokine and LIF haplodeficient mice show desynchronized glial responses, increased neurodegeneration and behavioral deficits after injury. Given the importance of LIF in recovery processes after traumatic brain injury (TBI), we sought to test the efficacy of intranasal (IN) LIF in a pediatric mouse model of mild closed head injury (mCHI). We assessed sensorimotor function and markers of neuroinflammation in wild type mice treated with IN-vehicle or IN-LIF. Two doses of LIF – 10 µl (20 ng) and 20 µl (40 ng) administered 2X daily for 3 days – were evaluated, of which 20 µl dose led to better recovery of sensorimotor function. Therefore, an intranasal dose of 20 µl was used for all subsequent studies. Mice treated with 20 µl of IN-LIF exhibited improved performance over IN-vehicle treated mice on the modified Neurological Severity Score, horizontal beam walk and horizontal ladder. At 5 days of recovery, astrogliosis (GFAP expression) and microgliosis (Iba-1 and CD-68 expression) were significantly reduced in the injured cortex of IN-LIF treated group over IN-vehicle but did not differ significantly from sham operated group. Punctate amyloid precursor protein accumulation, indicative of axonopathy, was observed in IN-vehicle group that was significantly reduced by IN-LIF. However, there was no evidence of cortical or white matter demyelination at this early time point as indicated by myelin basic protein (MBP) immunostaining. Western blot analyses of protein constituents of blood brain barrier (BBB) showed no significant differences implying that BBB integrity was largely intact. Altogether, these data further support the role of LIF as an important neuroprotective cytokine that modulates astroglial and microglial responses in the acute phase following injury. LIF also prevents early axonal damage. Our studies suggest that elevating levels of LIF *via* IN administration during the acute phase following mild TBI is a prospective therapeutic for improving neurological function.

B15

**CORTICAL BRAIN INJURY CAUSES RETROGRADE DEGENERATION OF AFFERENT BASAL FOREBRAIN CHOLINERGIC NEURONS VIA THE P75NTR**

Srestha Dasgupta, Laura Montroull, Wilma J Friedman

*RUTGERS UNIVERSITY, NEWARK, BIOLOGICAL SCIENCES, NEWARK, USA*

Basal forebrain cholinergic neurons (BFCNs) extend long projections to multiple targets in the brain to regulate cognitive functions and are compromised in numerous neurodegenerative disorders. To assess how injury to the target region of these neurons affects their viability *in vivo,* we are using the Fluid Percussion Injury (FPI) model to test the effects of injury at the cortex on the afferent BFCNs. Our preliminary studies show significantly fewer BFCNs ipsilateral to the injury compared to the contralateral side of the brain 7 and 14 days after the injury, an effect which is absent in p75 knock-out mice. These results suggest a retrograde degenerative effect of the cortical injury on the projecting BFCNs through p75NTR. Basal forebrain survival, growth, synaptic maintenance, and apoptosis is governed primarily by neurotrophins (NT). Treatment of BFCN neurons with mature NTs promote survival via the Trk family of receptors, while pro-neurotrophins (pro-NT) trigger apoptosis via p75NTR. Interestingly BFCNs express all the neurotrophin receptors throughout life and may access NTs locally or from their targets. To determine the effects of NT and proNT signaling on BFCN viability and function, we are using microfluidic and filter chamber cultures to segregate BFCN soma and axons *in vitro* allowing for compartmentalized treatment with pro/mature NTs. Our studies show that stimulation of BFCN axon terminals with proNGF which is a ligand for p75NTR, elicits retrograde degeneration of the axons and cell death of these neurons *in vitro.* Moreover, exposure of these neurons to mature or proNTs in the axonal or soma compartments may activate specific signaling mechanisms with different functional consequences. The knowledge of how pro or mature NTs affect axonal integrity and BFCN survival will shape our understanding of the role of NTs in BFCN development and in conditions of neuro-degeneration.

B16

**ASCORBIC ACID ENHANCES NICOTINE NEUROPROTECTIVE ROLES IN TRANSFERRIN-MEDIATED CORTICO-HIPPOCAMPAL NEUROTOXICITY**

Ismail Gbadamosi^1,2^, Olayemi Olajide^1^, Gabriel Omotoso^1^

*^1^ University of Ilorin, Anatomy, Ilorin, Nigeria*

*^2^ Nencki Institue of Experimental Biology, Translational Research in Neuropsychiatric Disorders, Warsaw, Poland*

The discovery of effective therapeutic strategies for the treatment of Alzheimer's disease could be advanced by studying the underlying disease biology vis-à-vis iron dyshomeostasis and molecules that modulate nAChRs. Nicotine, being an allosteric modulator of nAChRs, improves memory loss in AD without the withdrawal symptoms observed in orthosteric ligands of nAChRs. Ascorbic acid (AA), on the other hand, is a potent antioxidant molecule with known neuromodulatory effects. This study evaluated the neurotherapeutic advantages of combining Nicotine and ascorbic acid. Following institutional ethical approval (UERC/ASN/2018/1473), five groups (A-E) of male Wistar rats (n=8/group) were used for this study. Group A (control) was treated with distilled water daily for 8weeks. Transferrin-mediated neuroinflammation was induced in groups B-E through a daily oral infusion with 100 mg/kg of AlCl3 for four weeks. Groups C-E were then post-treated with ascorbic acid (100 mg/kg daily), nicotine (10 mg/kg daily) and nicotine (10mg/kg daily)+ascorbic acid (100mg/kg daily) respectively for four weeks. Following neurobehavioral tests to assess memory indices, the animals were euthanized for post-mortem studies. We observed that aluminium induced a reduction in long-and short-term memory indices in addition to perturbed cholinergic activities in the PFC and hippocampus. Atomic absorption spectrometric analysis of these brain regions revealed an increase in intracellular iron, which corresponded to overexpression of transferrin receptor protein (TRP). Nicotine-ascorbic acid treatment regimen reversed the reduction of long-and short-term memory indices through the restoration of the cholinergic system. These correlated with nicotine-dependent modulation of TRP expression, which was complemented by a significant attenuation of reactive oxygen species mediated by ascorbic acid. Summarily, our results have shown the role of ascorbic acid in enhancing nicotine neuromodulatory activities in transferrin-mediated behavioral decline and neuroinflammation in the PFC and hippocampus of Wistar rats.

B17

ANALYSIS OF SPHINGOLIPIDS PATTERN IN MICROGLIA AFTER TREATMENT WITH A REMYELINATING PROMOTING ANTIBODY

Chiara D'Aprile, Sara Grassi, Simona Prioni, Laura Mauri, Alessandro Prinetti

*University of Milan, Department of medical biotechnology and translational medicine, Segrate, Italy*

Multiple sclerosis (MS) is the most common demyelinating disease of the central nervous system and a major cause of neurological disability in young adults. The adult brain is able, up to a certain level, to repair damaged myelin prompting to the formation of new myelin sheath. However is a failing process that eventually leads to neuroin-flammation and neurodegeneration. For this reason, investigating possible strategies to promote remyelination could be a path to pursue. Microglia has a fundamental role in the processes of re-mye-lination: it activates after myelin damage and removes myelin debris, which is a limiting step for the remyelination process. Recombinant Human antibody IgM22 (rHIgM22) is able to successfully promote myelin repair in all mouse models of MS and, although little is known about its mechanism of action, it stimulates microglia clearance of myelin debris. Previously we observed deep changes in the sphingolipid pattern of cells belonging to the oligodendrocyte lineage after treatment with rHIgM22 and, since the antibody increases phagocytic activity in the microglial cell line BV-2, we decided to analyse this cell line through a steady-state metabolic labelling with ^3^H-sphingosine. Radiolabelled lipids were extracted, partially purified, separated by High Performance Thin Layer Chromatography and quantitatively analysed by digital auto-radiography. As a result, we observed significant changes in the lipid composition of BV-2 cells respect to control, mainly an increase of several gangliosides, whose exact identity is currently being determined through ESI mass spectrometry. Literature suggests that the mechanism of action of rHIgM22 might be mediated by the reorganization of sphingolipid-driven signalling complexes at cell surface, in cholesterol and sphingolipid enriched domains. After clarifying which lipids are involved in the rHIgM22 mediated signalling, we will investigate which proteins could be involved in the activity of these signalling complexes and if modulation of the composition of the lipid microenvironment could affect their activity, and thus microglia function.

B18

**EFFECT OF MICROGLIAL DEPLETION ON NEURODEGENERATION IN A MULTIPLE SCLEROSIS MODEL**

Anabella Di Pietro^1,2^, Victoria Wies Mancini^1,2^, Juana Pasquini^1,2^, Jorge Correale^3^, Laura Pasquini^1,2^

*^1^ Universidad de Buenos Aires. Facultad de Farmacia y Bioquímica, Departamento de Química Biológica, Buenos Aires, Argentina*

*^2^ Universidad de Buenos Aires-CONICET, Instituto de Química y Fisicoquímica Biológicas (IQUIFIB), Buenos Aires, Argentina*

*^3^ Instituto de Investigaciones Neurológicas Dr. Raúl Carrea, FLENI, Buenos Aires, Argentina*

Disability in patients with multiple sclerosis (MS) results from neu-ronal and axonal loss. Prolonged cuprizone (CPZ) intoxication is widely used as a MS model because it triggers chronic demyelination, neurodegeneration, astrogliosis and microgliosis. Exacerbated microglia (MG) activation is associated with impaired remyelination and neurodegeneration. As MG are physiologically dependent on colony-stimulating factor 1 receptor (CSF-1R) signaling, MG can be almost completely eliminated from the brain using CSF-1R inhibitors. Therefore, the present work aimed to evaluate the effects of CSF-1R inhibitor BLZ945 on myelin status, neurodegeneration and astrogliosis during chronic CPZ demyelination. Mice were fed either control or CPZ chow for 12 weeks and orally gavaged vehicle or BLZ945 from the 2nd week of CPZ treatment. BLZ945 induced a reduction in the microglial population in all structures evaluated. Astrogliosis was attenuated only in the cortex (CX), cerebellum (CB) and forceps major of the corpus callosum (fmjCC). A recovery in myelin basic protein (MBP) and Sudan black (SB) staining showed BLZ945 to protect myelin. The percentage of MBP-positive area increased in CX and throughout the CC, while SB intensity increased in the CB, fmjCC, anterior corpus callosum (aCC), fimbria of the hippocampus (FI) and striatum (ST). However, positive amino-cupric-silver staining was more prominent in axons traversing the ST and fibers throughout the CC with BLZ945 treatment. Axonal degeneration was accompanied by terminal axonal ovoids character-istic of inflammatory demyelination. These results indicate that neurodegeneration does not exclusively result from demyelination and that MG depletion could prevent demyelination but also exacerbate axonal degeneration.

B19

**THE DAAM2-VHL-NEDD4 AXIS GOVERNS DEVELOPMENTAL AND REGENERATIVE OLIGODENDROCYTE DIFFERENTIATION**

Xiaoyun Ding^1^, Juyeon Jo^2^, Chih-Yen Wang^2^, Carlo Cristobal^3^, Zhongyuan Zuo^4^, Qi Ye^2^, Marvin Wirianto^5^, Aaron Lindeke-Myers^2^, Jong Min Choi^6^, Carrie Mohila^7, 8^, Hiroshi Kawabe^9^, Sung Yun Jung^6^, Hugo Bellen^4, 10, 11^, Seung-Hee Yoo^5^, Hyun Kyoung Lee^2,11^

*^1^ Baylor College of Medicine, Graduate Program in Developmental Biology, Houston, USA*

*^2^ Baylor College of Medicine, Department of Pediatrics/Neurology, Houston, USA*

*^3^ Baylor College of Medicine, Graduate Program in Integrative Molecular and Biomedical Sciences, Houston, USA*

*^4^ Baylor College of Medicine, Molecular and Human Genetics, Houston, USA*

*^5^ The University of Texas Health Science Center, Department of Biochemistry and Molecular Biology, Houston, USA*

*^6^ Baylor College of Medicine, Center for Molecular Discovery, Houston, USA*

*^7^ Texas Children's Hospital, Department of Pathology, Houston, USA*

*^8^ Baylor College of Medicine, Department of Pathology and Immunology, Houston, USA*

*^9^ Max Planck Institute of Experimental Medicine, Department of Molecular Neurobiology, Goettingen, Germany*

*^10^ Baylor College of Medicine, Howard Hughes Medical Institute, Houston, USA*

*^11^ Texas Children's Hospital, Jan and Dan Duncan Neurological Research Institute, Houston, USA*

Dysregulation of the ubiquitin–proteasomal system (UPS) enables pathogenic accumulation of disease-driving proteins in neurons across a host of neurological disorders. However, whether and how the UPS contributes to oligodendrocyte dysfunction and repair after white matter injury (WMI) remains undefined. Here we show that the E3 ubiquitin ligase VHL interacts with Daam2 and their mutual antagonism regulates oligodendrocyte differentiation during development. Using proteomic analysis of the Daam2–VHL complex coupled with conditional genetic knockout mouse models, we further discovered that another E3 ligase Nedd4 is required for developmental myelination through stabilization of VHL via K63-linked ubiquitination. Furthermore, studies in mouse demyelination models and white matter lesions from patients with multiple sclerosis corroborate the function of this pathway during remyelination after WMI. Overall, these studies provide evidence that a signaling axis involving key UPS components contributes to oligodendrocyte development and repair and reveal a new role for Nedd4 in glial biology.

B20

**INFLAMMATION, MYELINATION, AND AXON DEGENERATION GENES ARE MODIFIED IN EXPERIMENTAL AUTOIMMUNE ENCEPHALOMYELITITIS OPTIC NERVES**

Micah Feri, Shane Desfor, Alyssa Anderson, Xinghao Shuai, Seema Tiwari-Woodruff

*University of California, Riverside, Biomedical Sciences, Riverside, USA*

Visual dysfunction is a prevalent feature in multiple sclerosis (MS), an autoimmune, inflammatory, demyelinating disease of the central nervous system (CNS). Visual dysfunction can be attributed to optic neuritis (ON) leading to vision loss, pain with eye movement, and deficiency in color vision. Considering ON is an early marker of MS, deterioration seen in the optic nerve may reflect pathology occurring in the CNS during disease. To explore potential targets for MS therapies, screening for proteins that mediate characteristic events in MS is imperative but difficult. Experimental autoimmune encephalo-myelitis (EAE) is one of the best models of MS and shows significant deficits in functional visual evoked potentials due to EAE-induced demyelination, inflammation, and axon degeneration. To investigate gene changes seen during MS optic neuritis, RNA from optic nerves of mice with EAE was extracted for NanoString nCounter gene analysis. Genes were categorized into three pathways that include neuroinflammation, myelination, and axon degeneration. A total of 760 genes were identified with 136 genes related to neuro-inflammation. In EAE, significant upregulation was observed in C1qa, C1qb, C1qc, and C3, all involved in the complement cascade within the innate immune system. Genes related to cytokines and chemokines demonstrate significant upregulation such as Ccl12, Ccl5, Cxcl10, Cxcl16, and Cxcr4. In addition, 13 oligodendrocyte-specific genes were identified, 6 of which are related to myelination. Genes include fatty acid 2-hydroxylase (Fa2h), galactose-3-O-sulfotransferase 1 (Gal3st1), gap junction protein 1 (Gjb1), myelin regulatory factor (Myrf), and UDP galactosyltransferase 8A (Ugt8a). Significant downregulation in myelination genes such as Gjb1, SRY-Box Transcription Factor 10 (Sox10), oligodendrocyte transcription factor 2 (Olig2), myelin oligodendrocyte glycoprotein (MOG), myelin basic protein (MBP), and Myrf were observed. Axon degeneration was evident with significant downregulation of PTEN Induced Kinase 1 (PINK1) and S100 Calcium Binding Protein B (S100B). These data demonstrate that specific genes related to inflammation, demyelination, and axon degeneration are modified in EAE. These genes can be possible targets for new treatments to alleviate optic neuritis in EAE and MS.

B21

**PLP1 IN THE ENS IS PREFERENTIALLY EXPRESSED DURING EARLY POSTNATAL DEVELOPMENT AS DM20 AND IS RELIANT ON AN INTRONIC ENHANCER**

Pankaj Patyal, Daniel Fil, Patricia Wight

*University of Arkansas for Medical Sciences, Physiology and Biophysics, Little Rock, USA*

Recently, the myelin proteolipid protein gene (*Plp1*) was shown to be expressed in glia of the enteric nervous system (ENS) in mouse.

However, beyond this, not much is known about its expression in intestine. To address this matter, we investigated *Plp1* expression at the mRNA and protein levels in intestine from mice at different ages (postnatal days 2, 9, 21 and 88). Here we show that *Plp1* expression preferentially occurs during early postnatal development, primarily as the DM20 isoform. Interestingly, the protein does not appear to be posttranslationally modified (i.e., lipidated) in intestine as *Plp1* products are in brain, based on differences in migration with SDS-PAGE. Moreover, by immunohistochemical analysis using cell type-specific markers, we demonstrate that the gene is expressed in neurons of the ENS, in addition to glia. Furthermore, through use of a couple of related *Plp1-lacZ* transgenic mouse lines, we show that the wmN1 enhancer region in *Plp1* intron 1 DNA is required for expression in intestine.

B22

**DNA METHYLATION DEPENDENT GLAST TRANSCRIPTIONAL CONTROL IN GLIA**

Ada Rodriguez, Arturo Ortega

*Cinvestav-IPN Mexico, Toxicologia, Ciudad de Mexico, Mexico*

Excitotoxicity is a form of neuronal death caused by hyperactivity of the main excitatory amino acid, glutamate (Glu). An uncontrolled receptor-meditated calcium influx triggers a plethora of signaling pathways resulting a death cascade. The constant exposure to several xenobiotics over the years can have a significant impact in the central nervous system (CNS), leading in the long-term run to an excitotoxic process. Hence, the extracellular levels of Glu must be tightly regulated in order to avoid prolonged receptor activation to this end, glial Glu transporters (GLAST, Glt-1) uptake Glu from the synaptic cleft after its release from the synaptic terminal, maintaining a homeo-static environment. The expression of these transporters under excitotoxic Glu concentrations has been widely studied, for example, the AMPA subtype of Glu receptors down regulates chglast (slc1A3) transcription via YY1 in a PKC-mediated signaling cascade. The YY1 transcription factor, as a member of the Polycomb group can be part of repressive and activating chromatin remodeling groups. Thus, YY-1 can target DNA methyltransferases or dioxygenases of methyl-ated cytosines to specific DNA sequences and by these means regulate the transcription of specific genes. Being the slc1A3 promoter an important target of YY1 involved its downregulation under excito-toxic conditions, we use here the well-characterized chick primary culture of Bergmann glia cells (BGC) and the human retina Muller MIO-M1 cells to address the epigenetic mechanisms associated to GLAST gene expression regulation. Thus far, we have been able to detect changes in YY1, DNMT3 and GLAST mRNA and protein levels in response to excitotoxic Glu levels. Likewise, global DNA methylation has been evaluated the same conditions. Moreover, in order to understand the effect of DNA methylation on GLAST function, 1 we evaluated the activity of this transporter after treatment with 5-Aza-2’-deoxycytidine an hypomethylation agent. These results favor the notion of a complex epigenetic regulation of GLAST expression.

B23

**AQP4 STOP CODON READTHROUGH FACILITATES AMYLOID-Β CLEARANCE FROM THE BRAIN**

Darshan Sapkota, Joseph D. Dougherty

*Washington University School of Medicine, Department of Genetics, St Louis, USA*

Alzheimer's disease (AD) is initiated by the toxic aggregation of Amyloid beta (Aβ). Immunotherapeutics aimed at reducing Aβ are in clinical trials but with very limited success to date. Identification of orthogonal approaches for clearing Aβ may complement these approaches for treating AD. In the brain, the astrocytic water channel Aquaporin 4 (AQP4) is involved in clearance of Aβ, and the fraction of AQP4 found perivascularly is decreased in AD. Further, an unusual stop codon readthrough event generates a conserved C-terminally elongated variant of AQP4 (AQP4X), which is exclusively perivascular. However, it is unclear if the AQP4X variant specifically mediates Aβ clearance. Here, using AQP4 readthrough-specific knockout mice that still express normal AQP4, we determine this isoform indeed mediates Aβ clearance. Further, with high-throughput screening and counterscreening, we identify small molecule compounds that enhance readthrough of the AQP4 sequence, and validate a subset on endogenous astrocyte AQP4. Finally, we demonstrate these compounds enhance brain Aβ clearance *in vivo,* which depends on AQP4X. This suggests derivatives of these compounds may provide a viable pharmaceutical approach to enhance clearance of Aβ and potentially other aggregating proteins in neuro-degenerative disease.

B24

**MURINE TOXOPLASMA GONDII INFECTION INDUCES MOLECULAR CHANGES IN DOPAMINERGIC NEURAL CIRCUITS**

Michael Shlossman^1^, Gabriela L. Carrillo^2,3^, Michael A. Fox^2,4^

*^1^ Fralin Biomedical Research Institute at Virginia Tech Carilion, School of Medicine, Roanoke, USA*

*^2^ Fralin Biomedical Research Institute at Virginia Tech Carilion, Center for Neurobiology Research, Roanoke, USA*

*^3^ Virginia Tech, Graduate Program in Translational Biology, Medicine, and Health, Blacksburg, USA*

*^4^ Virginia Tech, School of Neuroscience, Blacksburg, USA*

*Toxoplasma gondii* is a neurotropic protozoan parasite that infects ∼30% of the global population, and chronic infection has been linked to neuropathologies such as schizophrenia and seizure disorders. Evidence in mice suggests that chronic infection affects neuronal function in a variety of ways. In particular, murine and cell-based experiments have shown that *T. gondii* can increase both levels of dopamine and markers for dopamine synthesis localized to parasite cysts. In order to unravel how *T. gondii* impacts brain function, a more complete understanding of infection induced alterations in dopaminergic circuitry is needed. Our interest was to characterize how the parasite may alter the expression of host genes involved in neurotransmitter metabolism, transport, and signaling in several areas of the brain relevant to dopaminergic circuits. Adult C57BL/6J mice were chronically infected with Type II ME49 *T. gondii* cysts follwed by microdissection of tissue from prefrontal cortex, striatum, amygdala, and ventral tegmental area/substantia nigra. Gene expression analysis was then conducted in these 4 regions for 11 relevant genes normalized to actin. Our results show that chronically infected mice exhibit a consistent pattern of transcriptional down-regulation among genes involved in dopaminergic circuits, as well as glutamate signaling and metabolism. This pattern was most pro-nounced in the ventral tegmental area/substantia nigra. At this stage, no clear pattern has emerged reflecting possible functional consequences of these changes. Nonetheless, our results can aid in the understanding of mechanisms responsible for behavioral and neuropsychological changes in mice and humans. Moving forward, we aim to further investigate parasite induced derangements in neural circuits by analyzing levels of dopamine directly, as well as continuing preliminary immunostaining experiments to look for changes in expression and localization of proteins identified by our gene expression data.

B26

**FTY720 DECREASES CERAMIDE AND SPHINGOMYELIN LEVELS IN THE BRAIN AND PREVENTS MEMORY IMPAIRMENTS IN A AD MODEL**

Qian Luo^1^, Simone Crivelli^1,2^, Daan Kruining^1^, Caterina Giovagnoni^1^, Marina Damas^1^, Sandra Hoedt^3^, Dusan Berkes^4^, Etienne Waelkens^5^, Rita Derua^5^, Johannes Swinnen^5^, Jonas Dehairs^5^, Helga Vries^6^, Monique Mulder^3^, Jochen Walter^7^

*^1^ Maastricht University, Department of Psychiatry and Neuropsychology, Maastricht, Netherlands*

*^2^ University of Kentucky, Department of Physiology, Kentucky, USA*

*^3^ Erasmus MC University Medical Center, Department of Internal Medicine, Rotterdam, Netherlands*

*^4^ Slovak University of Technology, Department of Organic Chemistry, Bratislava, Slovak Republic*

*^5^ Leuven University, Laboratory of Lipid Metabolism and Cancer, Leuven, Belgium*

*^6^ Amsterdam UMC, Department of Molecular Cell Biology and Immunology, Amsterdam, the Netherlands*

*^7^ University of Bonn, Department of Neurology, Bonn, Germany*

A metabolic shift favoring ceramide production over sphingosine- 1-phosphate (S1P), contributes to Alzheimer's disease (AD) progression. Once the drug FTY720 phosphorylated, mimics S1P bio-activity but with an unclear underlying mechanism. In our study, Two doses of FTY720 (0.1 mg / kg and 0.5 mg / kg daily) were given by oral gavage to transgenic mouse models of familial AD carrying human apolipoprotein E. After 12 weeks of treatment, animals were challenged with behavioral tests for memory, locomotion and anxiety. Blood was withdrawn and brains were collected for sphingo-lipids analysis by mass spectrometry, RT-PCR and Aβ quantification also performed with the brain. The results showed that sphingosine kinase 1 was downregulated in E3FAD while S1P lyase was upre-gulated in E4FAD. In the cortex of E3FAD and E4FAD, Cer d18:1/ 16:0 and Cer d18:1/22:0 levels were elevated compared to litter controls. Low levels of S1P in the plasma were associated with higher probability of failing the memory test. FTY720 increased the likelihood of E4FAD to succeed in the memory test by 1.8-fold and reduced anxious behavior in APOE4 mice. Furthermore. FTY720 reduced ceramide and sphingomyelin levels, inflammation and Aβ concentration in E4FAD cortex. Our data indicates that FTY720 improves memory and anxious behavior in FAD mice, not only by antagonist effects on S1P receptors, but also by acting on the sphingolipid metabolism in the brain.

B27

**MODULATION OF THERMOGENESIS BY OMEGA 3 FATTY ACIDS IN THE HIBERNATING ARCTIC GROUND SQUIRREL**

Sarah Rice^1^, Evgeny Berdyshev^3^, Monica Mikes^1^, Doug Bibus^2^, Kelly Drew^1^

*^1^ University of Alaska Fairbanks, Chemistry/Biochemistry, Fairbanks, USA*

*^2^ Lipid Technologies, LLC, Austin, USA*

*^3^ National Jewish Health, Medicine, Denver, USA*

Omega 3 fatty acids have been shown to increase core body temperature (T_b_) in mammals and omega 6 polyunsaturated fatty acids (PUFAs) are known to influence T_b_ in hibernators. Hibernation, in part, is controlled by the central nervous system. T_b_ in hibernation is hypothesized to be regulated by the hypothalamic set-point. To the best of our knowledge, no one has investigated the influence of omega 3 fatty acids on the hibernating T_b_ of Arctic Ground Squirrel (AGS) or on hypothalamic fatty-acid derived neural signaling mechanisms. We hypothesized feeding omega 3 fatty acids would modulate T_b_ in hibernating AGS and influence PUFA-derived signaling lipids in the hypothalamus.

Wild juvenile AGS were fed either a diet high in omega 3 fatty acids or a standard chow control diet, high in omega 6 fatty acids, prior to hibernation. T_b_ was tracked with abdominal data loggers. Whole brain, white adipose tissue (WAT), brown adipose tissue (BAT) and plasma were collected during distinct stages of hibernation (torpor and arousal). Hypothalamic endocannabinoids and fatty acid amides and BAT, WAT and plasma fatty acids were measured.

Results show increased T_b_ during hibernation in animals fed a high omega 3 diet and increased BAT mass (p<0.05, t-test). Additionally, DHA and omega 3 fatty acids were increased in animals fed a high omega 3 diet (p<0.05, t-test). Results did not support our hypothesis that diet would alter major hypothalamic endocannabinoids, but we found hypothalamic palmitoylethanolamide (PEA), a fatty acid amide, and 2-AG, an endocannabinoid, increased during torpor compared to the euthermic phase of hibernation, arousal (p<0.05, t-test). In conclusion, our results indicate a dietary omega 3s modulate T_b_ of hibernating AGS. While diet did not influence hypothalamic endo-cannabinoids, hibernators showed seasonal trends in endo-cannabinoids.

This work was supported by NSF grant number 1258179 and NIH P20GM103395, UL1GM118991, TL4GM118992, and/or RL5GM118990.

B28

**MOLECULAR PHENOTYPING OF CERS2 RESCUE OF CERS1 MUTATION REVEALS GENES CRITICAL FOR PURKINJE CELL SURVIVAL**

Stefanka Spassieva^1^, Timothy McClintock^1^, Laura Goins^1^, Naazneen Khan^1^, Lihong Zhao^2^

*^1^ University of Kentucky, Physiology, Lexington, USA*

*^2^ Jackson Laboratory, Bar Harbor, USA*

Ceramide synthase 1 (CerS1) is the main neuronal ceramide synthase. CerS1 catalyzes the generation of C_18_ ceramide. We have previously reported a catalytically inactive mutation of CerS1 resulting in 50% reduction of brain C_18_ ceramide and significant increases in sphingoid bases causing Purkinje cell degradation and cerebellar ataxia. Ectopic expression of CerS2 in the neurons of the CerS1 mutant restores sphingoid bases to wild type levels, rescues Purkinje cells from degradation, and eliminates the ataxia phenotype. CerS2 isoform shares the same sphingoid base substrates as CerS1, but catalyzes the production of long-chain ceramides (C_22_-C_24_).

Therefore, the expression of CerS2 transgene in CerS1 mutant neurons did not restore wild type levels of C_18_ ceramides. In our current work, we performed RNA-seq analyses of the cerebella of the wild type, CerS1 mutant, and CerS1 mutant/CerS2 transgenic mice. The mRNAs reduced in CerS1 mutants were predominantly from genes expressed by Purkinje cells. 220 of these mRNAs were restored to wild type levels by ectopic expression of CerS2. Statistically overrepresented annotations linked to these genes include neuro-genesis, synaptic functions, calcium signaling, pleckstrin homology domain proteins, and SH3 domain proteins. In addition, we identified 110 mRNAs increased as a result of CerS1 mutation and normalized by CerS2 transgene expression. G-protein coupled receptor signaling and members of the complement system were overrepresented biological processes among these mRNAs. However, the molecular phenotype of rescued Cers1 mutants remained distinct from wild type mice, with 385 differentially abundant mRNAs showing overrepresentation of synapse function, ion transport, and transcriptional regulation annotations. Taken together, these results suggest that increased levels of sphingoid bases due to CerS1 deficiency affect the expression of genes critical for the survival and maintenance of cerebellar neurons. Moreover, our results predict that differences in the sphingolipids’ fatty acyl chain-length produce functionally distinct cerebellar neurons when Cers2 is used to rescue defects caused by CerS1 mutation.

B30

**INCREASING ACTIVATION OF THE SONIC HEDGEHOG PATHWAY NORMALIZES DYSREGULATION OF OLIG2 AND NKX2.2 IN TRISOMIC PRE-OPCS**

Jenny Klein^1,2^, Zhen Li^3^, Sanjeev Rampam^4^, Jack Cardini^4^, Ella Zeldich^2^, Tarik Haydar^3^

*^1^ Boston University, Graduate Program for Neuroscience, Boston, USA*

*^2^ Boston University, Department of Anatomy and Neurobiology, Boston, USA*

*^3^ Children's National Medical Center, Center for Neuroscience Research, Washington, DC, USA*

*^4^ Boston University, Department of Biomedical Engineering, Boston, USA*

Down syndrome (DS) is caused by triplication of chromosome 21. It is the most common genetic form of intellectual disability with a prevalence of 1 in 750 live births. Trisomy 21 results in global transcriptional dysregulation and previous work has shown that one of the alterations is the downregulation of a network of genes regulating the oligodendrocyte (OL) lineage. This suggests that perturbed OL development may be cause of the decrease in white matter in the brains of people with DS. To study the mechanism of this deficit, we differentiated two isogenic lines of iPSCs derived from people with DS into neural progenitor cells (NPCs) and pre-oligodendrocyte pro-genitor cells (pre-OPCs). We identified changes in the transition of trisomic cells from NPCs to pre-OPCs. This transition is tightly regulated by two transcription factors, OLIG2 and NKX2.2, which are essential to promote the commitment and differentiation of OLs. Upon application of a Sonic hedgehog pathway agonist (SAG), trisomic cells show a significant increase in OLIG2 expression at the RNA and protein level and a significant decrease in the expression of *Nkx2.2* at the RNA level. Hierarchal clustering and gene ontology identifies the hedgehog signaling pathway as the most enriched pathway driving the differentiation of the iPSCs from NPCs to pre-OPCs. Multiple genes within this pathway are dysregulated in trisomic pre-OPCs. Based on these findings, we increased the concentration of SAG during differentiation of the trisomic cultures and found that this treatment normalizes the level of expression of OLIG2 and NKX2.2 in the trisomic line. These observations imply that reduced responsiveness of trisomic cells to SHH signaling may drive the dysregulated expression underlying the first perturbed step of OL differentiation in DS.

B31

**DIFFERENTIAL RESPONSES OF BRAIN AND SPINAL CORD OLIGODENDROCYTE PRECURSOR CELLS TO EXTRACELLULAR LIPOPROTEIN PARTICLES**

Marie Mather, Luipa Khandker, Teresa Wood

*Rutgers University - New Jersey Medical School, Pharmacology, Physiology, and Neuroscience, Newark, USA*

Cholesterol comprises over 40% of oligodendrocyte lipid content and is often dysregulated in neurodegenerative diseases affecting myelin integrity. Progressive loss of myelin and impaired generation of new myelinating oligodendrocytes are hallmarks of diseases such as multiple sclerosis. Despite the prominence of potential promye-linating drugs which target sterol synthesis and our increasing knowledge of oligodendrocyte heterogeneity, few studies have explored cholesterol metabolism in both the brain and spinal cord. Therefore, understanding how cholesterol metabolism is regulated in different oligodendrocyte populations is essential to developing effective promyelinating therapies. Our previous study revealed that spinal cord oligodendrocyte precursor cells (OPCs) have higher expression of cholesterol synthesis enzymes than brain OPCs (Khandker et al, biorXiv), suggesting that OPCs of different regions may rely differently on intracellular cholesterol synthesis to support myelin production. Here we isolated O4+ oligodendroglia from the brain and spinal cord of mice to determine expression of low-density lipoprotein (LDL) receptors which facilitate uptake of extracellular cholesterol via lipoprotein particles.

We found that low density lipo-protein receptor related protein 1 (LRP1) expression is significantly higher in brain oligodendroglia compared to spinal cord oligodendro-glia throughout the peak of developmental myelination (P10-P18). Moreover, treatment of primary rat OPCs with varying concentrations of LDL resulted in an increase in myelin gene expression in brain OPCs whereas spinal cord OPCs showed no response to LDL treatment. These data suggest brain OPCs may have a greater dependence on extracellular cholesterol uptake rather than intracellular cholesterol synthesis.

B32

**HYPEROXIA INHIBITS THE GROWTH OF MOUSE FOREBRAIN OLIGODENDROCYTE PROGENITORS**

Lisamarie Moore^1^, Steven W. Levison^1^

*^1^ Rutgers Biomedical and Health Sciences, Pharmacology, Physiology & Neuroscience, Newark, USA*

*^2^ Rutgers University, Cancer Center, Newark, USA*

NG2 chondroitin sulfate proteoglycan positive oligodendrocyte progenitor cells (OPCs) reside throughout the brain. They divide asymmetrically and differentiate into myelinating oligodendrocytes throughout adulthood. OPCs have been successfully isolated from rodents using several techniques including magnetic beads, immuno-panning and exploiting differential centripetal adhesion. Whereas rat OPCs are relatively simple to propagate in vitro, it has been difficult to expand mouse OPCs in vitro and even more challenging to differentiate them once established in culture. In this study, we developed and characterized a simple and reproducible method to prepare large numbers of nearly homogenous cultures of primary mouse OPCs from postnatal day 0-2 mouse telencephala. Using the McCarthy and de Vellis mechanical separation method we separated OPCs from a mixture of glial cells and plated them onto fibronectin coated tissue culture plates in a biochemically defined medium containing fibroblast growth factor-2 (FGF-2) and platelet derived growth factor AA (PDGFAA). These cultures were maintained in a standard tissue culture incubator. However, they proliferated very slowly. By contrast, mouse OPCs doubled approximately every 7 days when grown in 30% B104 neuroblastoma conditioned medium supplemented with FGF-2 (B104CM+FGF-2) and in a 2% oxygen, nitrogen buffered environment. After 3 passages, greater than 99% of these OPCs were NG2+/PDGFRα+. In medium containing only FGF-2, mouse OPCs progressed to late stage OPCs whereupon A2B5 expression decreased and O4 expression increased. However, cells that remained in proliferation media, after repeated passages, eventually progressed to a late O 4+ OPC even with B104 present. When the mitogens were withdrawn and the medium was supplemented with T3 hormone as well as CNTF and Noggin, a subset of the OPCs differentiated into more mature Olig2+/O4 +/ MBP+ cells. Unlike rat OPCs, T3 or FGF2 alone were not sufficient to promote the differentiation and survival of mouse OPCs. These studies reveal significant differences between mouse and rat OPCs and an inhibitory role for oxygen in mouse OPC proliferation.

B34

**CANNABIDIOL INHIBITS MICROGLIAL INFLAMMATORY-TYPE REACTIONS THROUGH DECREASING GLUCOSE UPTAKE-DEPENDENT OXIDATIVE STRESS**

Maurício Dos-Santos-Pereira^1,2^, Francisco Guimarães^1^, Rita Raisman-Vozari^2^, Patrick Michel^2^, Elaine Del Bel^1^

*^1^ Universidade de São Paulo, Basic and Oral Biology, Ribeirão Preto, Brazil*

*^2^ Paris Brain Institute, Experimental Therapeutis of Parkinson's disease, Paris, France*

Here, we studied the anti-inflammatory potential of cannabidiol (CBD),the major non-psychoactlve component of cannabis. For that we used microglial cells in culture that were isolated from post-natal mouse brain through a procedure that relies on the adhesion preference of these cells to the polycation poly-ethyleneimine (Sepulveda-Díaz et aI, Glia, 2016; dos-Santos Pereira et aI, Glia, 2018). We established that CBD (1-10 µM) was highly efficient in reducing inflammatory-type responses triggered by the Toll-like receptor4 (TLR4) agonist, lipopolysaccharide (LPS, 10 ng/ ml). In particular, CBD strongly reduced the release of two pro-inflammatory cytokines TNF-a and IL-1ß and that of glutamate,a non-cytokine mediator of inflammation. Interestingly, CBD was also highly effective against other inflammatory signaling molecules,theTLR-2 agonist Pam3CSK-4 and the P2X7 agonist BzATP, indicating that CBD inhibitory effects were not restricted to a particular inflammatory pathway.The effects of CBD were pre-dominantly receptor independent; they were only marginally blunted by SCH336, a selective antagonist/inverse agonist at CB2 receptors and insensitive to antagonists of CB1 receptors and PPAR-y. Additional experiments revealed that CBD had the capacity to restrain LPS-Induced Inflammatory events by interfering with a signaling cascade involving the ROS producing enzyme NADPH oxidase and subsequently NF-xB dependent signaling. Importantly, we noticed that NF-KB inhibition by either CBD (1,10 µM) or TPCA-1, an IKB kinase inhibitor counteracted the rise in glucose uptake that is observed after exposure of microglial cells to LPS,suggesting that CBD occluded pro inflammatory events by lowering glucose consumption. Comforting this view, CBD anti-inflammatory effects were mimicked by 2-deoxy-D-glucose (2-DG), a synthetic non-metabollzable glucose analog. CBD and 2-DG led to a reduction of glucose-derived NADPH, a requisite factor for ROS production by NADPH oxidase. Altogether, our findings suggest that CBD possesses potent anti-inflammatory effects towards microglial cells through an antioxidant mechanism that restrains glucose utilization and NADPH synthesis.Present data also further confirm that CBD may have a therapeutic interest in conditions where neuro-inflammatory processes are prominent.

B35

**ISOLATING HETEROGENOUS REACTIVE ASTROCYTES DURING CNS INFECTION TO STUDY SUBSETS' UNIQUE ROLES DURING CHRONIC INFLAMMATION**

Zoe Figueroa^3^, Will Agnew^3^, Martin Riccomagno^2^, Todd Fiacco^2^, Emma WIlson^1^

*^1^ University of California, Riverside, Division of Biomedical Sciences, Riverside, USA*

*^2^ University of California, Riverside, Department of Neuroscience, Riverside, USA*

*^3^ University of California, Riverside, Neuroscience Graduate Program, Riverside, USA*

Neuroinflammation is common to neurodegenerative diseases and brain injuries, resulting in immune responses characterized by changes in astrocytes. Astrocytes ordinarily provide critical support for neurons but become reactive in response to brain disease, injury, or infection. Reactive astrocytes (RAs) are defined by their increased proliferation, enlarged cell bodies and processes, and change in function. A longstanding and unresolved issue is whether RAs contribute to or help alleviate disease progression, but overall, RAs have been discovered to be an important contributor to several neurological diseases. *Toxoplasma gondii* infects a third of the world's population and is asymptomatic except during times of immunocompromise when infection leads to severe neurological damage. *Toxoplasma* is a highly successful neurotropic parasite that causes persistent subclinical neuroinflammation due to cyst formation in neurons lasting for the lifetime of the host, making this a key resource for our studies. During chronic *Toxoplasma* infection, RAs demonstrate a neuroprotective function by inhibiting parasite replication via STAT1-mediated mechanisms, while simultaneously demonstrating detrimental effects by downregulating GLT-1, suggesting hetero-geneity exists among RAs. Utilizing flow cytometry, we have characterized surface integrin subsets found during chronic *Toxoplasma* infection. Experiments demonstrate during infection, potential chronic reactive astrocyte subsets are present. However, variability exists within these heterogeneous astrocytic naive and infected subsets. To further determine if astrocytes define a chronic inflammatory state, single cell RNA sequencing experiments on bulk astro-cytes at various stages of infection were conducted to determine if similar populations are expressed during acute compared to chronic infection. Furthermore, to address the issue of detecting a pure RA population, we have created a reporter mouse, *Lcn2 CreERT2*; *Rosa26 lsl-tdTomato*, that will allow for identification of RA subsets during infection for the first time, providing a novel tool for future use.

B36

**KETOGENIC DIET MODULATES MICROGLIAL MORPHOLOGY AT STEADY-STATE AND PROMOTES RESILIENCE TO REPEATED SOCIAL DEFEAT STRESS IN MICE**

Fernando González Ibáñez^1^, Kaushik Sharma^2^, Chloe McKee^3^, Katherine Picard^1^, Kanchan Bisht^2^, Haley A Vecchiarelli^3^, Nathalie Vernoux^1^, Jessica Deslauriers^1^, Marie-Ève Tremblay^3^

*^1^ CRCHU de Québec-Université Laval, Axe Neurosciences, Quebec, Canada*

*^2^ Center for Brain Immunology and Glia (BIG), University of Virginia, Department of Neuroscience, Charlottesville, USA*

*^3^ University of Victoria, Division of Medical Sciences, Victoria, Canada*

Ketogenic diet (KD; high-fat, low-carbohydrate) has emerged as a lifestyle change that may promote stress resilience. This study investigates the possible resilience-promoting properties of KD and aims to unravel underlying mechanisms by focusing on microglia specifically, the resident immune cells of the brain. Using 2 month-old adult male C57BL/6 mice, we studied the effects of KD *versus* normal diet (ND) exposure for 4 weeks. The consequences of chronic stress under KD *versus* ND were investigated by comparing non-stressed controls with animals undergoing 10 days of repeated social defeat (RSD). After RSD, mice underwent a social interaction test (SIT) to classify them as resilient or susceptible to stress. We are focusing on the ventral hippocampus CA1 *stratum radiatum*, previously shown to be affected by chronic stress. Our results show that after RSD, ketogenic diet increased the proportion of resilient animals, 57.14% of KD mice (n=28) *vs* 36.36% of ND (n=22). We studied the effect that ketogenic diet on its own might have on micro-glia. Using TMEM119/IBA1 double staining we have observed that KD does not affect microglia number and distribution. Nevertheless, the microglia of KD animals show increased soma and arborization area. This observation is very important given that changes in micro-glia morphology suggest changes in their function, suggesting. Further analyses of stress susceptible *versus* resilient animals, together with ultrastructural studies, will expand our understanding of KD on microglial function. Ongoing analysis using scanning electron microscopy will allow us to perform ultrastructural characterization of microglial function, their interactions with other cell types, synaptic elements, as well as provide valuable intracellular information regarding their phagocytic activity and markers of cellular stress.

B37

**EXPLORING THE ROLE OF B CELLS IN THE FORMATION OF ECTOPIC LYMPHOID TISSUE DURING NEUROINFLAMMATION**

Sravanthi Bandla^1^, Alexandra Lopez^2,1^, Angela Archambault^1^, Gregory Wu^1^

*^1^ Washington University School of Medicine, Department of Neurology, Saint Louis, USA*

*^2^ Wellesley College, Department of Neuroscience, Wellesley, USA*

Multiple sclerosis (MS) is an inflammatory demyelinating, auto-immune disorder of the central nervous system (CNS). In MS, B cells are thought to promote disease by driving antigen-specific CD4 T cell responses via cognate interaction dependent upon MHCII, producing pro-inflammatory cytokines, secreting antibody, and contributing to the formation of meningeal ectopic lymphoid tissues (ELT). ELTs are aggregates of leukocytes formed in non-lymphatic tissue under chronic inflammation and are associated with the progression of MS. We hypothesize that there are B cell-intrinsic properties critical for the infiltration and retention of B cells in the meninges during EAE. Using a murine system involving the expression of MHCII exclusively by myelin-specific B cells (B^APC^ mice), we studied the potential of Th1 lines to foster the development of ELT at different time points. B^APC^ mice receiving Th1 cells had a higher number of germinal center-like B cells (GL-7^+^) 14 days post-onset (DPO) compared to 7dpo and 21dpo. Single-cell RNA sequencing (scRNA-seq) was carried out to explore the extent, diversity and phenotype of B cells in the spinal meninges during neuroinflammation in comparison to deep cervical draining lymph nodes (DCLN). Distinct lymphoid clusters, including CD8 T, B, NK and myeloid lineage cells, were identified. Subcluster analysis was performed to identify the subtypes of B cells. We observed 5 subsets that express genes associated with memory or naive B cells such as Bcl2, Hhex, Mndal, and Cd38. These genes were higher in clusters 0, 1, and 3. Clusters 2 and 4 have some expression of these genes but are defined by their high expression of ribosomal or mitochondrial genes. Germinal center B cells were absent in B-cell clusters indicating that these clusters could be immature. We believe cluster 2 and 4 to be metabolically active B cells and this information needs to be further validated.

B38

**VIRTUAL REALITY TOOLS AND METHODS FOR TEACHING NEUROCHEMISTRY**

Randy Stout, Jerry Jose, Edward Piscitelli, Nicholas Frangella, Maddison Messmer, Chloe Bodden, Salonie Dave

*New York Institute of Technology College of Osteopathic Medicine, Department of Biomedical Sciences, Old Westbury, USA*

Virtual Reality (VR) technology allows users to visualize digital models in three dimensions. Many useful VR, Augmented Reality (AR), and screen-based applications have been developed to teach neuroanatomy. The newest-generation commercial versions of VR equipment allow the user to interact with the virtual environment in an intuitive way. Head, hand and gaze tracking combine with customizable user inputs to allow rich data collection on VR user performance. The developments described above allowed us to build teaching material that extends beyond immersive viewing of anatomical structures to enable constructive learning activities in VR that are customized the learner's experience with the aim of addressing personalized knowledge gaps. We will present a workflow and a prototype VR training tool to allow medical students to explore and interact with the molecular level anatomy and physiology of neural components including the blood-brain barrier, tripartite synapse, and hypothalamus/pituitary. We will demonstrate aspects of our training application that extend beyond immersive learning to include personalized interactive learning of neurobiological concepts.

B39

**ASSESSMENT OF GAD67 EXPRESSION IN THE PIRIFORM CORTEX OF CNTNAP2 KNOCKOUT MICE**

Lars Udo-Bellner^1,2^, Kimberly Fasciglione^1^, Himani Jani^1^, Yan Li^1^, Aaron Miller^1^, Yamini Nori^1^, Mac Josh Reandelar^1^, Violeta Roumenova^1^, William Shin^1^, Gonzalo Otazu Aldana^1^, Randy Stout^1,2^

*^1^ New York Institute of Technology, Biomedical Sciences, Old Westbury, USA*

*^2^ New York Institute of Technology, Center for Biomedical Innovation, Old Westbury, USA*

Olfactory deficiency (OD), a characteristic of Autism Spectrum Disorder (ASD), can lead to diminished appetite and malnutrition. Our group and others have established a link between ASD, OD and copy-number variations in the contactin associated protein 2 (Cntnap2) gene. Although wild-type (WT) and Cntnap2^−/-^ (KO) mice show similar olfactory discrimination of odors, odor discrimination by KO mice is severely impaired in the presence of novel background odors. Cntnap2 is expressed particularly in the nodes of Ranvier in several brain regions, including the olfactory tract. Impaired expression leads to neuronal migration abnormalities globally and reduced number of GABAergic inhibitory interneurons in the neocortex. We argued that this impaired neuronal function and density might be responsible for the observed deficit in odor detection in presence of background odors. Fluorescence imaging was used with immunolabelled coronal sections of perfused and fixed adult mouse brains. Glutamate decarboxylase, GAD67, anti-body labelling allowed quantitation of GABAergic inhibitory inter-neurons of WT and KO mice within the Piriform Cortex (PC), a region in the brain associated with olfactory function. Images were captured on a Nikon C2 inverted confocal microscope, then denoised using the image-denoising module in the NIS Elements software. The number of cell bodies of GAD67 positive neurons in the layer 1 of the PC were counted using the NIH Fiji-Image J software. Initial analysis revealed no difference of GAD67-positive neurons between WT and KO mice within layer 1 of the piriform cortex. Based on this we are gathering tiled z-stack images to include all the layers of the piriform cortex with the aim to also count the cell bodies of GABAergic neurons in the layers 2/3 of the PC that are responsible for feedback inhibition.

B40

**CONTRIBUTIONS OF VRAC TO CELL SWELLING, NEURONAL EXCITABILITY AND EPILEPTIFORM ACTIVITY**

Erin Walch^1,2^, Alex Bilas^3^, Murad Aldoghmi^3^, Devin Binder^1,2,3^, Todd Fiacco^2,3^

*^1^ UC Riverside, School of Medicine, Riverside, USA*

*^2^ UC Riverside, Division of Biomedical Sciences, Riverside, USA*

*^3^ UC Riverside, Department of Molecular, Cell and Systems Biology, Riverside, USA*

In this project, we aim to understand the role of volume regulated anion channels (VRAC) on brain tissue swelling and neuronal excitability. VRAC are activated in response to cell swelling, and release glutamate and other anions into the extracellular space in an effort to regulate cell volume. To study VRAC in the context of cell volume and excitability changes, we have generated multiple transgenic mouse lines using Cre-lox technology to selectively ablate VRAC in specific cell types (astrocytes, neurons, or most neural pro-genitors). Each Cre line is first being characterized by crossing to a Rosa26-lsl-TdTomato reporter line to assess cell-type specific expression. The role of VRAC in cell swelling is being evaluated in each transgenic line by recording real-time confocal volume responses of astrocytes and neurons in acute mouse hippocampal slices using two common models of tissue swelling - application of 40% hypoosmolar aCSF or elevated-potassium aCSF (10.5 mM). In complementary experiments, the impact of VRAC ablation on neuronal excitability is being measured by recording AMPA and NMDA receptor mediated EPSCs and action potential generation in CA1 pyramidal neurons. Preliminary data suggest that the ablation of VRAC does not produce significant changes in the swelling profiles of astrocytes, with neurons still yet to be measured. However, despite lack of major effects on astrocyte volume, swelling-induced neuronal activity was moderately impacted in astrocyte-targeted Cre lines. Neurons from VRAC cKO slices exhibited fewer slow inward currents in response to swelling in comparison to controls. Furthermore, to complement direct cell swelling models, we found that application of the pro-epileptic drug 4-AP resulted in less ictal activity in astrocyte VRAC cKO slices compared to controls. Overall our findings thus far suggest that astrocytic VRAC may play key roles in mediating excitability under both physiological and patho-logical conditions.

B41

**EXOSOMAL CERAMIDE MEDIATES NEUROTOXICITY OF AMYLOID BETA (AΒ) IN ALZHEIMER'S DISEASE**

Ahmed Elsherbini^1^, Alexander Kirov^2^, Michael Dinkins^2^, Sanjib Karki^1^, Simone Crivelli^1^, Guanghu Wang^1^, Haiyan Qin^1^, Zhihui Zhu^1^, Priyanka Tripathi^1^, Stefanka Spassieva^1^, erhard Bieberich^1^

*^1^ University of Kentucky, Physiology, Lexington, USA*

*^2^ Augusta University, Neuroscience, Augusta, USA*

Amyloid beta is a pathologic hallmark of Alzheimer's disease (AD), however, the mechanism of Aβ neurotoxicity is not fully understood. Exosomes associate with Aβ, but it is not clear how this association would affect Aβ neurotoxicity. We report that the sphingolipid ceramide mediates neurotoxicity of Aβ. We show that sera from AD transgenic mouse model (5xFAD) and AD patients, but not the WT or healthy controls, contain a subpopulation of astrocyte-derived exosomes that are enriched with ceramide and are prone to aggregation (termed astrosomes) as confirmed by nanoparticle tracking and cluster analyses. Upon being taken up by Neuro2A cells and human iPS cell-derived neurons, these astrosomes are shuttled to mitochondria where they induce mitochondria clustering, evident by elevation of expression of the fission protein dynamin related pro-tein1 (Drp1). Using proximity ligation assays, we show that Aβ forms a complex with voltage dependent anion channel 1 (VDA1), a protein that functions as gatekeeper for the entry and exit of mito-chondrial metabolites and is key player in mitochondria-mediated apoptosis. Complex formation colocalized with ceramide cotrans-ported with Aβ by astrosomes. The interaction between Aβ and VDAC1 leads to caspase3 activation and subsequently apoptosis. Aβ-associated astrosomes, but not Aβ alone, induced neurite frag-mentation and neuronal cell death, suggesting that association with astrosomes substantially enhances Aβ neurotoxicity in AD. Interestingly, the novel ceramide analog N-oleoyl serinol (S18) prevented the aggregation of exosomes, and Aβ association with astrosomes, and reduced Aβ interaction with VDAC1, indicating that exosomal ceramide mediated binding of Aβ to astrosomes and mitochondrial damage. Our data suggests that association of Aβ with ceramide in astrosomes enhances Aβ interaction with VDAC1 and mediates Aβ neurotoxicity in AD, which can be prevented by novel ceramide analogs. This work is supported by NIH R01AG034389 and R01NS095215, and VA 1 I01 BX003643.

B42

**PROBING AMYLOID-Β PROTOFIBRILS WITH A CONFORMATION-SELECTIVE ANTIBODY**

Shikha Grover^1^, Thao Pham^1^, Anna Jones^1^, Cristina Sinobas Pereira^1^, Michael R. Nichols^1^, Michael Gross^2^, Saketh Chemuru^2^

*^1^ University of Missouri-St. Louis, Department of Chemistry & Biochemistry, St. Louis, USA*

*^2^ Washington University, Department of Chemistry, St. Louis, USA*

Antibodies are critical tools throughout every aspect of Alz-heimer's disease (AD) including research, diagnostics, and therapy. Additionally, antibodies provide the only clues for the existence of various conformational species in tissue specimens from humans or mouse models. One important target of antibodies in AD is the amyloid-β peptide (Aβ), the primary component of senile plaques in the brain and the initial trigger for AD onset. Aβ, particularly Aβ42, is prone to aggregation, forming a variety of soluble and insoluble conformational species during the process. Our studies show that one soluble aggregated species, termed protofibrils, significantly interacts with microglia and is highly proinflammatory. Due to the importance of this Aβ42 species, we developed an antibody termed AbSL that selectively recognizes protofibrils over other Aβ forms. Immunization of rabbits with isolated Aβ42 protofibrils generated a high-titer antiserum with a high affinity and a strong selectivity for Aβ42 protofibrils over Aβ42 monomers and fibrils. AbSL did not react with amyloid precursor protein and recognized distinct patho-logical features in AD transgenic mouse brain slices. The serum was affinity-purified over an Aβ42 protofibril column to yield apAbSL and a monoclonal form of AbSL (mAbSL) was developed, cloned, sequenced, expressed and purified. Both apAbSL and mAbSL anti-bodies were integrated into numerous ELISA formats that are sensitive and selective for Aβ42 protofibrils. Initial experiments using both antibody competition and mass spectrometry techniques identified a conformational epitope on protofibrils that involved both the N-and C-terminal domains of Aβ42 in the pro-tofibril structure. Additional studies demonstrated a sub-stoichiometric inhibitory effect of mAbSL on Aβ42 aggregation dynamics. By targeting protofibrils, the AbSL antibody may have potential diagnostic and therapeutic uses in AD tissue and patients.

B43

**ENDOLYSOSOME FERROUS IRON ENHANCES MITOCHONDRIAL REACTIVE OXYGEN SPECIES, FRAGMENTATION, AND DYSFUNCTION**

Peter Halcrow, Jonathan Geiger*

*University of North Dakota School of Medicine and Health Sciences, Biomedical Sciences, Grand Forks, USA*

Endosomes and lysosomes (endolysosomes) are acidic organelles that are important both physiologically and pathologically. Implicated in the physiological and pathophysiological processes are readily releasable stores of cations including ferrous iron (Fe^2+^); an essential cofactor for various enzymes and the generation of reactive oxygen species (ROS). Because of the physiological and pathological relevance of Fe^2+^ in generating ROS via the Fenton reaction and because intra-mitochondrial iron originates from endocytosed ferric iron, it was important to specifically quantitate [Fe^2+^] in endo-lysosomes and explore the extent to which and mechanisms by which endolysosome iron is an upstream event of mitochondrial ROS generation, fragmentation, and dysfunction. First, using U87MG astro-cytoma cells and primary rat neurons, we determined the fluo-rescence dye FeRhoNox-1 was specific for Fe^2+^, that FeRhoNox-1 colocalized to endolysosomes, and levels of endolysosome [Fe^2+^] were 36.6±13.6 µM under normal conditions; [Fe^2+^] increased to 75±15.7 µM in cells treated with ferric ammonium citrate (FAC), and decreased to 0.08±0.05 µM in cells treated with the endolysosome-specific iron chelator deferoxamine (DFO). In control cells, 68% of endolysosomes contained [Fe^2+^] ranging from 0 to 50 µM while 32% contained [Fe^2+^] ranging from 51 to 150 µM. In FAC treated cells, 32% contained [Fe^2+^] between 0 to 50 µM and in DFO treated cells 100% contained [Fe^2+^] between 0 to 50 µM. Second, we found endo-lysosome de-acidification with bafilomycin A1 (Baf A1) or chloroquine (CQ) increased released iron from endolysosomes, which resulted in increased iron in the cytosol and in mitochondria. Baf A1 and CQ increased ROS levels in the cytosol and mito-chondria, which DFO blocked. We demonstrated endolysosome-resident two-pore channels released iron from endolysosomes, and mitochondrial permeability transition pores regulated iron uptake in-to mitochondria. Third, mitochondrial fragmentation was increased by CQ, which DFO blocked. Our findings suggest endolysosome iron release is an upstream event, sufficient to increase mitochondrial ROS, fragmentation and dysfunction, and chelating endolysosome iron might alleviate mitochondrial dysfunction that appears to play important roles in the pathogenesis of neurodegenerative diseases. (Supported by P30GM103329, R01MH100972, R01MH105329, R01MH119000 and 2R01DA032444)

B44

**CALPAIN ISOFORMS AND CD4+ T-CELL RESPONSES IN ANIMAL MODELS OF PARKINSON'S DISEASE**

Azizul Haque^1^, Supriti Samantaray^2^, Mollie Mollie Capone^1^, Azim Hossain^1^, Varduhi Knaryan^2^, Denise Matzelle^2,3^, Donald Shields^2^, Ariana Farrand^4^, Heather Boger^4^, Naren Banik^2,3^

*^1^ MUSC, Microbiology and Immunology, Charleston, USA*

*^2^ MUSC, Neurosurgery, Charleston, USA*

*^3^ Veterans Administration, VA, Charleston, USA*

*^4^ MUSC, Neuroscience, Charleston, USA*

Parkinson's disease (PD) is a neurodegenerative disorder characterized by a progressive loss of dopaminergic neurons in the substantia nigra (SN). In addition to midbrain dopaminergic nigrostriatal degeneration in PD, cell damage in extra-nigral sites such as spinal cord (SC) motor neurons has been observed. The mechanisms of this degenerative process in both brain and SC remain elusive, although inflammation is believed to be a common factor involved in many neurodegenerative diseases, including PD. The infiltration of inflammatory T cells, activation of microglia/astrocytes, and detrimental factors produced by these cells may be responsible for degeneration and disease progression in PD. Calpain, a calcium activated cysteine protease, plays a pivotal role in SN and SC (spinal cord) degeneration in PD; its role in α-synuclein aggregation, activation of microglia, and T cell migration/activation suggest calpain may be critical in promoting the inflammatory process and disease progression. While calpain-1 cleavage of α-synuclein promotes synuclein aggregation in PD and PD-like diseases, the precise involvement of the two major calpain isoforms, calpain-1 and calpain-2, in α-synuclein processing and immune activation in PD remains poorly understood. Studies in our lab identified a subtype of CD4+ T cells in MPTP (1- methyl-4-phenyl-1,2,3,6-tetrahydropyridine) mice, which was abolished by calpain inhibitor administration, suggesting activation of calpain plays a role in CD4+ T cell activation in PD. siRNA-mediated knockdown of calpain-2 inhibited activation of CD4+ T cells in vitro, indicating distinct calpain isoforms may be involved in the regulation of CD4+ T cells in PD. Importantly, the previously noted upregulation of CD4+ T cell subpopulation was markedly attenuated following calpain inhibition in MPTP mice, suggesting that calpain activation and generation of distinct CD4+ T cell subset(s) may have critical roles in the inflammatory and neuro-degenerative processes in PD.

PS-B-45

**MICRORNA MIR-21 MIMIC PROTECTS BRAIN AFTER EXPERIMENTAL STROKE IN RODENTS**

Raghu Vemuganti, Mary Lopez, Robert Dempsey

*University of Wisconsin, Neurological Surgery, Madison, USA*

Stroke is a leading cause of death and disability in adult humans that promotes significant motor, cognitive and neuropsychiatric dysfunction. However, there is no efficacious therapy to prevent post-stroke brain damage and neurologic deficits. Recent studies showed that modulating specific microRNAs (miRNAs) leads to neuroprotection and better functional recovery after stroke in rodents. As the reagents like miRNA mimics and antagomiRs are available to rapidly increase or decrease the levels of a specific miRNA, they became attractive targets for stroke therapeutic development. We pre-sently identified that miR-21 levels increase in a sustained manner when cerebral ischemic tolerance was induced in adult rodents. As bioinformatics analysis showed that miR-21 targets several pro-apoptotic and pro-inflammatory molecules that are known mediators of ischemic brain damage, we tested the efficacy of a miR-21 mimic to protect post-stroke mouse brain. Intracerebral injection of a miR- 21 mimic in adult mice increased the cerebral levels of miR-21 by >60 fold that sustained up to 2 days without any signs of toxicity. Treatment with miR-21 mimic induced significant neuroprotection (smaller infarcts by ∼56%) and motor function recovery compared to control mimic treatment in both male and female mice subjected to focal ischemia by transient middle cerebral artery occlusion. The miR-21 mimic treated mice didn't show any change in the rCBF before, during or after MCAO. The miR-21 mimic treatment also significantly decreased infarct volume and motor dysfunction in aged male and female mice compared to corresponding age/sex matched control mimic treated cohorts. Treatment with miR-21 mimic intra-venously at 30 min of reperfusion decreased the post-ischemic infarct volume and prevented splenic atrophy. In addition, IV miR-21 mimic treatment prevented the post-ischemic induction of several pro-apoptotic and pro-inflammatory genes. Thus, these studies indicate the therapeutic potential of miR-21 after focal cerebral ischemia.

B46

**PROLONGED ENVIRONMENTAL ENRICHMENT PROMOTES DEVELOPMENTAL MYELINATION**

Evan Goldstein^1^, Vera Pertsovskaya^2^, Thomas Forbes^1^, Jeffrey Dupree^3^, Vittorio Gallo^1^

*^1^ Children's National Hospital, Neuroscience, Washington, USA*

*^2^ The George Washington University, School of Medicine and Health Sciences, Washington, USA*

*^3^ Virginia Commonwealth University, Department of Anatomy and Neurobiology, Richmond, USA*

Postnatal neurodevelopment is profoundly influenced by environmental experiences. Environmental enrichment is a commonly used experimental paradigm that has uncovered numerous examples of experience-dependent plasticity in health and disease. However, the role of environmental enrichment in normal development, especially glial development, is largely unexplored. Oligodendrocytes, the myelin-forming glia in the central nervous system, provide metabolic support to axons and establish efficient saltatory conduction by producing myelin. Indeed, alterations in myelin are strongly correlated with sensory, cognitive, and motor function. The timing of developmental myelination is uniquely positioned to be influenced by environmental stimuli, as peak mye-lination occurs postnatally and continues into adulthood. To determine if developmental myelination is impacted by environmental experience, mice were housed in an enriched environment during peak myelination through early adulthood. Using translating ribosome affinity purification, oligodendrocyte specific RNAs were isolated from subcortical white matter at various post-natal ages. RNA-sequencing revealed that differences in the oligo-dendrocyte translatome were predominantly evident after prolonged and continuous environmental enrichment. These translational changes corresponded with altered oligodendrocyte lineage cell dynamics and enhanced myelination. Furthermore, consistent with increased developmental myelination, enriched mice displayed enhanced motor coordination on a beam walking task. These findings indicate that protracted environmental stimulation is sufficient to modulate developmental myelination and to promote behavioral function.

B47

**OLIGODENDROCYTES DEATH INDUCES SENSORIMOTOR AND COGNITIVE DEFICIT IN A L-NAME RAT-MODEL OF PRE-ECLAMPSIA**

Olayemi Ijomone^1,2^, Philemon Shallie^1,3^, Thajasvarie Naicker^1^

*^1^ University of KwaZulu-Natal, Department of Optics and Imaging, Doris Duke Medical Research Institute, College of Health Sciences, Durban, South Africa*

*^2^ University of Medical Sciences, Department of Anatomy, Ondo, Nigeria*

*^3^ Olabisi Onabanjo University, Department of Anatomy, Ago-Iwoye, Nigeria*

Pre-eclampsia (PE) is a pregnancy complicated syndrome that affects multiple organs including the brain that continue post-delivery in both mother and the offspring. We evaluated the expression of oligodendrocytes in the brain of PE rat model through development as well as the cognitive changes and other behavioural modifications that may occur later in the life of offspring of PE-like rat model. Pregnant rats divided into early-onset and late-onset groups were administered with N □ -nitro-L-arginine methyl (_L_-NAME) through drinking water at gestational days (GD) 8-17. Rats were allowed free access to water throughout the pregnancy. At GD 19, post-natal day (PND) 1 and 60, rats were sacrificed and brain excised for further analysis. The offspring were subjected to behavioural studies for cognitive and sensorimotor impairments before sacrificed at PND 60. Results showed significant down-regulation in the expression of OLIG2 in PE at GD 19 brain which persists till PND 60. Likewise, there was a significant increase in the latency to locate the platform in Morris water maze, time to traverse the balance beam and reduced hanging time on the wire test between the control and the PE treated. PE could lead to impaired neuronal signalling through demyelination which may contributes significantly to long-term sensorimotor and cognitive deficit.

B48

**NEURONS AND ENDOTHELIAL CELLS INDUCE ASTROCYTE MATURATION**

Zila Martinez-Lozada^1^, William Todd Farmer^2^, Keith K. Murai^2^, Michael B. Robinson^1^

*^1^ Children's Hospital of Philadelphia, Pediatrics, Philadelphia, USA*

*^2^ McGill University, Centre for Research in Neuroscience, Montreal, Canada*

Astrocytes are positioned between neurons and blood vessels with fine processes that oppose synapses and endfeet that enwrap the vasculature. This localization allows astrocytes to participate in several aspects of brain homeostasis, including glutamate clearance, but it also permits neurons and endothelia to influence astrocyte biology. Astrocytes express two subtypes of glutamate transporters. The expression of one of these transporters, GLT1/EAAT2, increases dramatically during synaptogenesis, providing a surrogate marker of astrocyte maturation. We and others have demonstrated that astro-cytes cultured alone express little or no GLT1 and that co-culturing with neurons or endothelia induces expression of GLT1 in astrocytes. While several signaling pathways have been implicated in neuron-dependent induction, we previously showed that Notch is required for the effect of endothelia. Our research has two goals: 1) determine which components of Notch signaling mediate the effect of endo-thelia, and 2) determine how neurons and endothelia synergistically affect the transcriptome of astrocytes. To address the first goal, we cultured cortical astrocytes in the presence or absence of the mouse endothelioma cell line bEND.3. Using recombinant Notch ligands, neutralizing antibodies, and shRNAs directed against Notch ligands in bEND.3 cells, we found that both delta-like Notch ligands, DLL1 and DLL4, are involved in endothelial-dependent induction of GLT1. Our studies also showed that astrocytes increase expression of DLL4 in endothelia. This now published study showed that reciprocal communication between astrocytes and endothelia contributed to the maturation of both cells (Martinez-Lozada & Robinson Neurochem Int 2020). To address the second goal, we cultured cortical astrocytes alone or in the presence of bEND.3 or rat cortical neurons, or both. We isolated astrocytes using fluorescence-activated cell sorting and performed RNA-seq and bioinformatic analysis. We found that neurons and endothelia have both complementary/additive and competitive effects on the astrocyte transcriptome. Overall, our study indicates that astrocytes receive an intricate combination of signals from neurons and endothelial cells which ultimately dictate astrocyte biology.

B49

**IL-1 REPROGRAMMING OF ADULT NSC LIMITS NEUROCOGNITIVE RECOVERY AFTER VIRAL ENCEPHALITIS BY MAINTAINING A PROINFLAMMATORY STATE**

Robyn Klein, Allison Soung, Charise Garber, Lauren Vollmer

*Washington University, Internal Medicine, St. Louis, USA*

Innate immune responses to emerging RNA viruses are increasingly recognized as having significant contributions to neurologic sequelae, especially memory disorders. Using a recovery model of West Nile virus (WNV) encephalitis, we show that, while macro-phages deliver the antiviral and anti-neurogenic cytokine IL-1β during acute infection; viral recovery is associated with continued astrocyte inflammasome-mediated production of inflammatory levels of IL-1β, which is maintained by hippocampal astrogenesis via IL- 1R1 signaling in neural stem cells (NSC). Accordingly, aberrant astrogenesis is prevented in the absence of IL-1 signaling in NSC, indicating that only newly generated astrocytes exert neurotoxic effects, preventing synapse repair and promoting spatial learning deficits. *Ex vivo* evaluation of IL-1β-treated adult hippocampal NSC revealed the upregulation of developmental differentiation pathways that derail adult neurogenesis in favor of astrogenesis, following viral infection. We conclude that NSC-specific IL-1 signaling within the hippocampus during viral encephalitis prevents synapse recovery and promotes spatial learning defects via altered fates of NSC progeny that maintain inflammation.

B50

ALTERATIONS TO GUT MICROBIOTA IN INDUCIBLE C1Q KNOCKOUT MICE IN ALZHEIMER'S MOUSE MODELS

Tiffany Petrisko, Andrew Oliver, Claudia Weihe, Katrine Whiteson, Andrea Tenner

*University of California, Irvine, Molecular Biology and Biochemistry, Irvine, USA*

The complement (C’) system, a critical component of the innate immune system, is activated in the context of Alzheimer's disease (AD) pathology. It has previously been demonstrated that genetic ablation or pharmacologic inhibition of the classical C’ initiator, C1q, provides protection from gliosis and neuronal loss in animal models of AD. To determine if deletion of C1q alters the gut microbiome and thereby possibly contributes to changes in disease progression, we assessed the microbiome of the Arctic mouse model of AD using a tamoxifen-inducible knockout of C1q. WT and Arctic C1qa^FL/FL^ mice containing the RosaCre^ERT2^ transgene were treated with tamoxifen or vehicle at 6 months of age, and housed according to treatment. Fecal samples were collected at 12-12.5 months and subjected to microbial focused Illumina sequencing. C1q deficiency (C1q-iKO) was confirmed by western blot. Deletion of C1q significantly increased alpha diversity in Arctic animals compared to their WT counterpart. Beta-diversity, as calculated by Bray-Curtis dissimilarities, indicated significant variation between WT and Arctic C1q-iKO animals, with a trend in variation between Arctic animals with and without C1q. *Alistipes* and *Turicibacter* were identified by random forest analysis as two genera that were critical for distinguishing the four groups.

*Alistipes* was significantly increased in C1q-iKO Arctic animals compared to Arctic. A trending increase was observed in C1q-iKO animals compared to C1q sufficient WT. No significant differences were observed in *Turicibacter*. However, Arc C1q-iKO mice displayed a trending decrease in *Turicibacte*r levels compared to Arctic animals. Both *Alistipes* and *Turicibacte*r have the potential to negatively impact gut serotonin levels.

Overall, this study demonstrates that C1q-iKO increases alpha diversity and greater diversity of the microbial communities in the context of AD like pathology. C1q deletion particularly affected serotonin regulating bacteria in Arctic animals. Future studies will assess cognitive function to determine if C1q-induced alterations to the gut microbiome may influence memory. Finally, these observations provide a foundation for assessing the impact of C1q on gut microbiome that may affect outcomes in mouse models of disease.

B51

**REVISITING THE MODEL OF THE BASAL GANGLIA THROUGH NEUROCHEMICAL CONTENT OF STRIATOFUGAL NEURONS**

Laetitia Reduron^1,2^, Martin Parent^1,2^

*^1^ Laval University, Faculty of Medicine, Dept of Psychiatry and Neuroscience, Quebec City, Canada*

*^2^ CERVO Brain Research Centre, Integrative Neuroscience & Experimental Therapies, Quebec City, Canada*

**Objectives.** The striatum (STR) is the main integrative component of the basal ganglia (BG) with only few efferent projections targeting mainly the pallidum (GP), the entopeduncular nucleus (ENT) and the substantia nigra pars reticulata (SNr). The current model of the BG relies on the segregation of striatal efferent projections into a direct and indirect pathway, originating respectively from GABAergic striatal neurons expressing D1 dopamine receptor, substance P (SP) and dynorphin (DYN) and projecting to the SNr and the ENT (direct pathway), and striatal neurons expressing D2 and enkephalin (ENK) and projecting to the GP (indirect pathway). Previous single-axon tracing studies in rats and monkeys have shown that most striatal neurons possess an axon that arborizes into all striatal targets, suggesting that striatal efferent projections are not as segregated as previously thought. Here, we present data that support this hypothesis by showing peptide distribution within D1 and D2 striatal neurons. We aim to provide single-axon reconstructions of D1 and D2 striatal neurons in mice and assess neuropeptide (SP, DYN, ENK) distribution within their axons.

**Methods**. *In vivo* injections of cre-dependent AAV were performed in the striatum of DRD1-cre and DRD2-cre transgenic mice. Immunohistochemical staining for SP, DYN and ENK allowed to visualize peptide distribution within infected neurons by using confocal microscopy.

**Results.** At the time of submission, experiments are still being conducted. Preliminary data has been acquired showing ENK, SP and DYN distribution within D1 and D2 infected axons, in all striatal targets.

**Conclusion.** Our preliminary data supports the hypothesis that the D1 and D2 striatal neurons carry GABA and different neuropeptides along their axons and arborize into all striatal targets, in which they differentially release peptides. Combined with single-axon reconstructions of D1 and D2 infected neurons, we aim to characterize the complex axonal trafficking of neuropeptide in the collateralized axons of striatofugal neurons.

B52

**INTRACELLULAR AMYLOID BETA OLIGOMERS INHIBIT LONG TERM DEPRESSION OF MEDIUM SPINEY NEURONS OF THE NUCLEUS ACCUMBENS**

Nicolas Riffo^1^, Odra San Martin^2^, Eduardo Fernandez^1^, Marco Fuenzalida^2^, Luis Aguayo^1^

*^1^ University of Concepcion, Physiology Department, Concepción, Chile*

*^2^ University of Valparaiso, Center of Neurobiology and Brain Plasticity, Valparaiso, Chile*

Alzheimer's Disease (AD) is a neurologic disorder causing dementia in an increasingly aging population. Recent evidence shows that the limbic system, critical to the regulation of emotional behaviors like motivation, reward, or anxiety, is early affected in AD progression, where the presence of intracellular Amyloid Beta (iAß) has been reported. The Nucleus Accumbens (NAc) is a central area of the mesolimbic system and is particularly affected in humans and animal transgenic mice models with AD, however, the effects that iAß could have in the medium spiny neuron (MSN) of the NAc have not been explored. It is recognized that synaptic plasticity mediated by long-term potentiation (LTP) and long-term depression (LTD), are mechanisms that control complex behaviors like memory, learning, and reward, being particularly affected in neurodegenerative diseases like AD, however, the possibility that iAß could inhibit LTD in MSN neurons of the NAc is largely unknown. In this work we characterize the iAß effects on LTD of MSN neurons of wild type mice brain slices through a electrophysiologic spike time-dependent plasticity protocol (STDP), and we found that iAß inhibit LTD without affecting neurotransmitter release probability. In addition, iAß increased the total post-synaptic event frequency and interestingly, the events amplitude was increased after LTD induction. These results bring new information about the effects of iAß on the plasticity of limbic areas like NAc to help to understand the early changes in AD like anhedonia, motivation, and memory.

## Poster Session C

C01

**HIV-1 TAT AND MORPHINE HAVE REGION-SPECIFIC EFFECTS ON MYRF GENE REGULATION IN CNS WHITE MATTER/OLIGODENDROCYTES**

Kelly Flounlacker^1^, Yun Hahn^1^, Kurt Hauser^2^, Pamela Knapp^1^

*^1^ Virginia Commonwealth University, Anatomy and Neurobiology, Richmond, USA*

*^2^ Virginia Commonwealth University, Pharmacology and Toxicology, Richmond, USA*

HIV-associated neurocognitive disorders (HAND) remain pervasive even with increased efficacy and use of antiretroviral therapies, and opiate use/abuse among HIV+ individuals has been documented to exacerbate CNS deficits. White matter (WM) alterations, including myelin pallor, and volume and structural alterations detected by diffusion tensor imaging (DTI), are a common observation in HIV+ individuals. A study using non-human primates infected with simian immunodeficiency virus suggests that WM may actually harbor latent virus, since antiretroviral treatment reduced levels of viral DNA in grey matter but not in WM (Perez et al, J. Neurovirology, 2018). Using a transgenic mouse that expresses the HIV-1 Tat protein, we examined in vivo effects of Tat and opiates (morphine) on WM, at times ranging from 2-6 wk co-exposure using genomic, biochemical, and morphological methods. After 6 wk chronic exposure, significant differences were observed in multiple myelin basic protein (MBP) isoforms. Outcomes were region-specific (hippocampus, striatum, corpus callosum, pre-frontal cortex), and included both individual and interactive effects. For example, HIV Tat exposure increased levels of the 18.5 kDa MBP isoform in hippo-campus but not striatum; both HIV Tat and morphine affected 21.5 kDa isoform levels in striatum; morphine interaction mitigated the effects of Tat on MBP levels in hippocampus and pre-frontal cortex, but not striatum. RNA sequencing of striatal tissue revealed several candidate genes associated with oligodendrocyte precursor populations and myelin integrity that may be related to WM pathology, including transferrin, atypical oligodendrocyte markers GPR17 and NDRG1, and the oligodendrocyte transcription factor Myrf. Myrf is implicated in numerous neurological disorders, and is critical for proper oligodendrocyte differentiation and maturation. Western blot analyses identified regional differences in the effect of Tat and morphine on Myrf protein levels, some of which coincided with changes in Myrf transcriptional targets MBP and MAG. Support: DA044939.

C02

**SPHINGOLIPID-DEPENDENT MEMBRANE ORGANIZATION AND SIGNALING ORCHESTRATING MYELIN REPAIR**

Sara Grassi, Simona Prioni, Chiara D'Aprile, Livia Cabitta, Paola Giussani, Laura Mauri, Alessandro Prinetti

*University of Milan, Department of Medical Biotechnology and Translational Medicine, Milan, Italy*

Recombinant human IgM22(rHIgM22) binds to myelin and oligo-dendrocytes(OLs) and promotes remyelination in mouse models of multiple sclerosis(MS). However, the target antigen and the signaling mechanisms through which rHIgM22 exerts its function are still unclear.

*In vitro* analysis revealed that rIHgM22 binds to sulfatide, but also to phosphatidylinositol, phosphatidylserine and phosphatidic acid. Moreover, the composition of the lipid microenvironment of its antigen can modulate the affinity of the antibody, suggesting that reorganization of lipid membrane might be relevant in its biological activity.

In rat mixed glial cells(MGC), rHIgM22 causes an increase in the levels of PDGFαR and induces a dose-dependent proliferative response in all the cells in the culture, with the most significant response associated with astrocytes. Moreover, rHIgM22 increases the production and release of sphingosine 1-phosphate(S1P) in MGC while total levels of ceramide remain unchanged. Furthermore, release of S1P is strongly reduced by a selective inhibitor of PDGFαR. Increased S1P production is not mediated by regulation of sphingosine kinase 1 and 2, instead, we observe a significant reduction of S1P lyase. Remarkably, rHIgM22 treatment does not induce changes in the production and/or release of S1P in astrocytes, but it increases its release in BV2 cells, suggesting that rHIgM22 indirectly influences the proliferation of astrocytes in MGCs, by affecting ceramide/S1P balance.

Analysis of the effect of rHIgM22 on glycosphingolipid metabolism in MGC and astrocytes revealed no significant effects on the lipid pattern, while in OPCs and OLs we observe an increase in the levels of different gangliosides, known for their ability to interact with and modulate the activity of different growth factor receptors.

Considering all this, we propose that rHIgM22 protective effects might be mediated by alterations of lipid-dependent membrane organization and/or signalling in different cell types present in the nice of MS lesions and that a complex cross talk between different cell types is underlying the ultimate repair effect elicited by this antibody.

C03

**SCHWANN CELLS WITH FIG4 DEFICIENCY ARE PREDISPOSED TO DEMYELINATION**

Bo Hu^1^, Daniil Moiseev^1^, Jun Li^1,2^

*^1^ Wayne State University School of Medicine, Neurology, Detroit, USA*

*^2^ John D. Dingell VA Medical Center, Neurology, Detroit, USA*

**Objectives:** Segmental demyelination consistently occurs in patients with Charcot-Marie-Tooth disease type-4J (CMT4J) caused by recessive mutations in *FIG4* gene. Our previous study has demonxstrated an increased intracellular Ca^2+^ in FIG4 deficient Schwann cells due to lysosomal Ca^2+^ channel dysfunction. In this study, we tested a hypothesis that whether FIG4 deficient Schwann cells are sensitized to demyelinate upon the challenge of extra Ca^2+^.

**Methods:** Surgically exposed sciatic nerve in 3-month-old *Fig4*^*f/f*^ and Schwann cell conditional knockout (*scFig4*^−*/*−^ ) mice was wrapped by gauze soaked with 10µM Ca^2+^ Ionophore A23187, while the contralateral sciatic nerve was treated with vehicle for 3 hours. The mice were allowed to recover for 10 days, followed by nerve conduction studies and teased nerve fiber analysis for counting segmental demyelination.

**Results:** A23187 significantly decreased conduction velocity (CV) in *scFig4* ^−/−^ nerves, compared with that in vehicle-treated *scFig4* ^−/−^nerves (A23187 8.8±0.8 m/s vs. vehicle 13.4±3.0 m/s; *p*<0.01, n=5 for each group). A23187 did not affect CV in *Fig4* ^f/f^ nerves (A23187 30.0±3.2 m/s vs. vehicle 28.8±4.0 m/s; *p*>0.05; n=5). Segmental demyelination was observed in 9.0±1.1% of nerve fibers from *scFig4* ^−/−^ nerves treated with A23187, 2.2±0.2% in *scFig4* ^−/−^nerves treated with vehicle, 2.3±0.4% in *Fig4* ^f/f^ nerves treated with A23187, and or 2.0±0.6% in *Fig4* ^f/f^ nerves treated with vehicle (*p*<0.01, 100-200 fibers counted/mouse, n=5 mice for each group). Segmental demyelination was associated with an increase of Schwann cell dedifferentiation factor c-Jun level.

**Conclusions:** FIG4-deficiency impairs the capacity of Schwann cells to “buffer” extra Ca^2+^, thereby sensitizing the cells to demyelination.

In a live animal, the extra Ca^2+^ in Schwann cells could be induced by axon excitation or cytokines released from macrophages.

**Supported by:** VA (IBX003385A) and NINDS (R01NS066927).

C04

**MODULATION OF LANOSTEROL SYNTHASE DRIVES 24,25-EPOXYSTEROL SYNTHESIS AND OLIGODENDROCYTE FORMATION**

Zita Hubler^1^, Ryan M. Friedrich^1^, Joel L. Sax^1^, Dharmaraja Allimuthu^1^, Farrah Gao^1^, Adrianna M. Rivera-Leon^1^, Matthew J. Pleshinger^2^, Ilya Bederman^1^, Drew J. Adams^1^

*^1^ Case Western Reserve University, Genetics and Genome Sciences, Cleveland, OH, USA*

*^2^ Case Western Reserve University, Pharmacology, Cleveland, OH, USA*

Small molecules that promote the formation of new myelinating oligodendrocytes from oligodendrocyte progenitor cells (OPCs) are potential therapeutics for demyelinating diseases. We recently established inhibition of specific cholesterol biosynthesis enzymes and resulting accumulation of 8,9-unsaturated sterols as a unifying mechanism through which many such molecules act. To identify more potent sterol enhancers of oligodendrocyte formation, we synthesized a collection of 8,9-unsaturated sterol derivatives and found that 24,25-epoxylanosterol potently promoted oligodendrocyte formation. In OPCs, 24,25-epoxylanosterol was metabolized to 24,25-epoxycholesterol via the epoxycholesterol shunt pathway. Increasing flux through the epoxycholesterol shunt using genetic manipulation or small-molecule inhibition of lanosterol synthase (LSS) increased endogenous 24,25-epoxycholesterol levels and OPC differentiation. Notably, exogenously supplied 24,25-epoxycholes-terol promoted oligodendrocyte formation despite lacking an 8,9- unsaturation. This work highlights epoxycholesterol shunt usage, controlled by inhibitors of LSS, as a target to promote oligodendro-cyte formation. Additionally, sterols beyond the 8,9-unsaturated sterols, including 24,25-epoxycholesterol, drive oligodendrocyte formation.

C05

**ARYL HYDROCARBON RECEPTOR: A NOVEL GLAST REGULATOR**

Janisse Silva, Arturo Ortega

*Cinvestav, Toxicology, Mexico City, Mexico*

Glutamate (Glu) is the main excitatory neurotransmitter of the Central Nervous System of mammals, exerts its effects by activating specific membrane receptors present in neurons and glia cells. Extracellular Glu levels are tightly regulated by a family of high-affinity sodium-dependent transporters known as excitatory amino acid transporters (EAAT), which remove Glu from the synaptic cleft, internalizing it to mostly to glial cells. When Glu is inefficiently removed, a phenomenon of neuronal death can occur due to the over activation of Glu receptors, it is known as excito-toxicity. Glu glial transporters are essential for glutamatergic neuro-transmission and to avoid excitotoxicity, and a deficiency in the expression of transporters can lead to the development of pathologies such as Alzheimer's disease, among others. Glu transporters are regulated at several levels, including transcription of their gene, mRNA stability and maturation, post-translational modifications and mem-brane traffic. In this work, we focused in the transcriptional regulation of the *slc1a3* gene. Using the well-characterized *in vitro* model of chick cerebellar Bergmann glial cells in which only the EAAT1 / GLAST transporter is expressed, we describe here the effect of 2,3,7,8-tetrachlorodibenzo-p-dioxin (TCDD) exposure in GLAST function and expression levels. Bioinformatic analysis of the *chglast* promoter revealed the presence of xenobiotic response (XRE) DNA binding sites. A time and dose dependent increase in GLAST activity and protein levels was found suggesting a new element that regulates *slc1A3* expression. These results strengthen the notion of the pivotal role of glia cells in the regulation of glutamatergic signaling.

C06

**FROM IPSCS TO MOTOR NEURONS: SPLICING, GROWTH, AND EXCITABILITY DEFICITS IN DARS2-RELATED LEUKODYSTROPHY**

Shiqi Guang^1^, Brett O'Brien^1^, Qian Li^1^, Philippe Hubo^1^, Ali Fatemi^1,2^, Mingyao Ying^1,2^, Christina Nemeth^1^

*^1^ Kennedy Krieger Institute, Moser Center for Leukodystrophies, Baltimore, USA*

*^2^ Johns Hopkins School of Medicine, Department of Neurology, Baltimore, USA*

*DARS2*-related leukodystrophy, also called Leukoencephalopathy with Brainstem and Spinal cord involvement and Lactate elevation (LBSL), is a rare disorder characterized by childhood-onset of slowly progressive spasticity, cerebellar ataxia, and dorsal column dysfunction with wide-ranging severity. Infantile-onset patients with rapid neurological deterioration, and adulthood-onset oligosymptomatic cases have also been reported. LBSL is caused by mutations in *DARS2*, which encodes the mitochondrial aspartyl-tRNA synthetase, an enzyme which charges aspartate to its cognate tRNA. Almost all patients are compound heterozygotes for *DARS2* mutations, most of which carry a missense mutation on one allele and a splice-site mutation in intron 2 on the other allele leading to exon 3 exclusion and a frameshift. Considering the exclusive complexity of pre-mRNA splicing mechanisms in human, conditional knockout of *Dars2* in mice does not address the contributions of specific variants; however, patient-derived induced pluripotent stem cells (iPSCs) have opened a new platform to investigate pathology in disease-relevant cells. IncuCyte live cell imaging reveals significant developmental differences between control and LBSL patient iPSC differentiated motor neurons (iMNs) and microelectrode arrays (MEA) show decreased electrical activity of LBSL iMNs throughout differentiation. Due to the high occurrence of intron 2 mutations, much focus has been placed on understanding the effects of increased production of mRNAs lacking exon 3. Human splicing software illustrates intronic splicing silencers (ISSs) at the end of intron 2 are relatively stronger than exonic splicing enhancers (ESEs) in exon 3, making the 3’ splice-site of exon 3 naturally weak. We verified by RT-qPCR that abnormally spliced transcripts exist in control iPSCs and iMNs and that the relative expression of aberrant transcripts increased in LBSL in a cell-type specific manner. Careful modeling and understanding of pathogenic variants will facilitate the testing of potential therapeutics, such as antisenseoligonucleotides (ASOs), which show promises to correct the exon 3 splicing pattern in LBSL.

C07

**UNDERSTANDING THE GENETIC REGULATION OF NEURONAL PLASTICITY WITHIN THE MATURE RAT CENTRAL NERVOUS SYSTEM**

Derick Thompson, Malak Alzidaneen, Abiodun Odufuwa, John Watt

*University of North Dakota, Biomedical Sciences, Grand Forks, USA*

The decline in neuronal plasticity within the mature mammalian central nervous system following injury is a well-known phenomenon that has yet to be elucidated. Our study utilizes the magnocellular neurosecretory system which is comprised of magnocellular neurons (MCN) of the supraoptic nucleus (SON) that produce and transport oxytocin and vasopressin along their axonal projections to the posterior pituitary for secretion into the bloodstream. In young rats (35 days), this system possesses an in-trinsic property for axonal regeneration following unilateral lesion of MCN axons that occurs within the contralateral uninjured SON. However, in mature rats (125 days), this capacity for regeneration is lost. Our study aims to identify key transcriptome and epigenome alterations that occur during aging to explain this loss in neuro-regeneration. In uninjured rats, we observed key changes in the transcriptome of the mature rat SON, including genes involved in the inhibition of axonal guidance and activation of immune system pathways. Furthermore, we observed a reduction in genes of key neurotrophic factors such as BDNF within our mature group. These data may suggest a mechanism for the age-related loss of axonal regeneration. We anticipate that such changes in gene expression will correlate to changes in DNA methylation and chromatin remodeling. Identifying these alterations that occur within our uninjured aging model will provide a fundamental baseline to assess our future findings within the axotomized SON.

C08

**COMPREHENSIVE ANALYSIS OF THE MICROGLIA TRANSCRIPTOME ACROSS THE HUMAN LIFESPAN USING SINGLE CELL RNA SEQUENCING**

Moein Yaqubi^1^, Jack Antel^1,2^, Luke Healy^1,2^

*^1^ McGill University, IPN, Montreal, Canada*

*^2^ Montreal Neurological Institute, Neuroimmunology Unit, Montreal, Canada*

Microglia as the main resident immune cells of the central nervous system (CNS) have critical, underappreciated roles in development and in the maintenance of brain homeostasis. Microglia also become highly reactive throughout the course of all neurological disease including multiple sclerosis. To gain a clear insight into the role of microglia in CNS pathology we first need to understand the molecular mechanisms underlying development of these cells.

Furthermore, it has been shown that animal model organisms consistently fail to mimic human physiological conditions. In the present study, we performed whole, single-cell RNA sequencing on 13,568 microglia isolated from surgical samples of second trimester fetal, pediatric (18 months to 2 years old), adolescent (10 to 14 years old) and adult (40 to 62 years old) brains. Using Seurat (v3) package to analyze the data we showed there are multiple subtypes of micro-glia during human development contributing to a continuum genes expression. We showed fetal microglia have a distinct transcriptomic expression profile compared to all post-natal samples. Based on the transcriptional signatures we predict fetal cells to have a higher phagocytic capacity than microglia from other ages while postnatal microglia have a greater proinflammatory gene signature and this increases with age. Moreover, we revealed the adolescent derived microglia were transcriptionally distinct from pediatric and adult cells, and they exhibited the most metabolically active transcriptomic profile. Finally, we correlated our findings about human microglia with mouse microglia datasets from the literature and observe that in spite of high correlation between human and mouse, microglia samples cluster according to their species and not according to developmental age. To understand the diverse function of human microglia during disease we must first gain insight into the gene expression programs that underlie each stage of normal development. This study is a valuable resource to explore transcriptional changes of human microglia during development at the single cell level.

C09

**VOLUME-REGULATED ANION CHANNELS FACILITATE PROLIFERATION OF THE PRIMARY BRAIN TUMOR GLIOBLASTOMA**

Shaina Orbeta^1^, Martin Bach^1^, Corinne Wilson^1^, Iskandar Abdullaev^2^, Yu-hung Kuo^3^, Alexander Mongin^1^

*^1^ Albany Medical College, Department of Neuroscience and Experimental Therapeutics, Albany,NY, USA*

*^2^ National University of Uzbekistan, Institute of Biophysics, Tashkent, Uzbekistan*

*^3^ University of California San Francisco, Department of Surgery, Fresno,CA, USA*

Glioblastoma (GBM) is the most common and deadly primary brain tumor. Recent work in the field identified volume-regulated anion channels (VRACs), as important players in progression of several cancers. The current study tested the role of VRAC in GBM cell proliferation by targeting the essential subunit of this channel, leucine-rich repeat-containing family 8 member A (LRRC8A). First, we analyzed the abundance of VRAC components, LRRC8A-E, using a qRT-PCR approach. Four out of five LRRC8 gene products, LRRC8A-D, were highly expressed in GBM tissue. Among LRRC8 isoforms, LRRC8A was upregulated up to 18-fold in 16 out 18 GBM tissue samples as compared to non-malignant brain tissue (commercial sources). Similar expression patterns were observed in primary GBM cell cultures: up to 25-fold upregulation compared to primary human astrocytes (commercial sources). Functional expression of VRAC in primary GBM cells was validated with a radiotracer assay, and its role in cell proliferation probed by an RNAi approach. siRNA knockdown of LRRC8A reduced cell growth by up to 60% in two primary GBM cell lines, as established by MTT assay and DAPI counting of adherent cells. Parallel LDH release measurements showed no evidence for increased cell death suggesting an effect on proliferation. The detailed FACS analysis of siRNA-treated cells did not reveal significant distortions in cell cycle progression. Western blot examination of cyclins and cyclin-dependent kinases did not identify consistent changes in cyclin-dependent machinery. In the ongoing work, we are exploring alternative mechanisms of LRRC8A knockdown on GBM cell growth. Overall, this work links LRRC8A-containing heteromeric VRAC to the proliferative potential of the primary brain tumor GBM. Targeting VRAC may be utilized as a new treatment modality.

Supported by Dr. Louis Skarlow Memorial Trust and NIH R01 NS111943

C10

**REDOX-FYN-C-CBL (RFC) PATHWAY REGULATES O-2A/OPC CELL CYCLE EXIT AND DIFFERENTIATION**

Yunpeng Pang^1,3^, Christopher Folts^2^, Ibro Ambeskovic^3^, Mark Noble^3^

*^1^ University of Rochester, Neuroscience Graduate Program, Rochester, USA*

*^2^ Vertex Pharmaceutical, Research & Development, Boston, USA*

*^3^ University of Rochester, Department of Biomedical Genetics, Rochester, USA*

Thyroid hormone (TH) plays an important role in the development of the central nervous system. In particular, TH signaling is critical for the differentiation of oligodendrocyte precursor cells (OPCs) into oligodendrocytes, which myelinate the CNS. Although gene regulations by TH and TH receptors have been well-studied, it is still not clear how TH initiates cell cycle exit in dividing OPCs. Previous work from our laboratory revealed that intracellular redox status is a central regulator of the balance between self-renewal and cell-cycle exit in dividing OPCs. Here, we report a critical role of hyper-activation of the c-Cbl tumor suppressor protein, via the redox-Fyn-c-Cbl (RFC) pathway, in inducing cell cycle exit of OPCs in response to TH. The effects of TH were prevented by knockdown of either Fyn kinase or c-Cbl, and also by preventing TH-induced increases in OPC oxidation. Moreover, these effects of TH are not mediated by classical TH receptors. We also found that other O-2A/OPC differentiation signals, including TGF-beta 1 and BMP4, converge on requiring ROS generation and activation of the RFC pathway to cause O-2A/OPC cell cycle exit. Taken together, our studies provide a new molecular pathway by which multiple distinct signaling molecules regulate O-2A/OPC cell cycle exit by convergence on ROS-mediated RFC pathway activation.

C11

**COMPLETE CASK LOSS CAUSES CEREBELLAR DEGENERATION IN HUMAN AND MOUSE**

Paras Patel^1^, Julia Hegert^2^, Ingrid Christian^2^, Alicia Kerr^1^, Leslie LaConte^1^, Michael Fox^1^, Sarika Srivastava^1^, Konark Mukherjee^1^

*^1^ Virginia Polytechnic Institute and State University, Fralin Biomedical Research Institute, Roanoke, USA*

*^2^ Orlando Health, Arnold Palmer Hospital for Children, Orlando, USA*

Mutations in the X-linked *CASK* gene in human have been associated with microcephaly with pontine and cerebellar hypoplasia (MICPCH) in heterozygous girls and epileptic encephalopathies in hemizygous boys. The precise molecular cause of this pathology is uncertain but interactions between CASK protein and the Tbr1- Reelin pathway have been implicated. Herein, we first present an autopsy of a boy hemizygous for an early truncation mutation of the *CASK* gene resulting in no functional protein production. Gross and fine histopathological analysis reveals severely diminished cerebellar volume; however no defect in lamination, neuronal differentiation, or migration is observed, precluding the role of the Tbr1-Reelin pathway in cerebellar pathology. Interestingly, molecular analysis reveals an upregulation of glial fibrillary acidic protein immuno-staining, indicating reactive astrogliosis common in neuro-degenerative disorders. This led us to hypothesize a degenerative, rather than a developmental cause for the observed diminishment of cerebellar volume. Further investigating this in a murine model, we utilize a *Calb2* driven Cre recombinase to delete *Cask* from the majority of cerebellar neurons. Ablation of CASK from post-migratory neurons of the cerebellum results in otherwise normal cerebellar development, lamination, and function up to approximately post-natal day 45 when degeneration begins to be observed. The properly developed cerebellum degenerates over several weeks to result in profound diminishment in volume and culminates in severe motor ataxia. We propose that the underlying etiology of MICPCH resultant from CASK-loss is degenerative rather than developmental and that this may extend to other X-linked disorders such as Rett Syndrome. Random X-chromosome inactivation results in 50% of cells in heterozygous girls having functional CASK and a plateau of cerebellar degeneration while hemizygous boys with no CASK continue to degenerate.

C12

**IN VIVO DELIVERY OF SYNTHETIC COLLAGEN XIX MATRICRYPTINS TRIGGERS INHIBITORY CIRCUIT ASSEMBLY IN NEOCORTEX**

Meredith Rahman^1,2^, Jianmin Su^2^, Michael Fox^2,3^

*^1^ Virginia Tech Carilion School of Medicine, Medicine, Roanoke, USA*

*^2^ Fralin Biomedical Research Institute at Virginia Tech Carilion, Center for Neurobiology Research, Roanoke, USA*

*^3^ Virginia Tech, Department of Biological Sciences, Blacksburg, USA*

Schizophrenia is a neuropsychiatric disorder associated with impaired formation or function of perisomatic inhibitory synapses formed by fast-spiking, Parvalbumin-expressing interneurons. Collagen XIX, a brain-derived extracellular matrix (ECM) protein associated with familial schizophrenia, contributes to the assembly of these same perisomatic inhibitory synapses. Proteolytic processing of ECM proteins in vivo leads to the release of bio-activie peptides known as matricryptins. In the case of Collagen XIX, such enzymatic processing leads to the shedding of a non-collagenous peptide (NC1 [XIX]) that is sufficient to trigger inhibitory synaptogenesis in vitro. Here, we investigated whether intracortical administration of NC1 [XIX] impacts the assembly of inhibitory perisomatic synapses in vivo. Synthetic NC1 [XIX] or control peptides were injected intra-cortically into one hemisphere of wild-type or Col19a1-/- mutant mice (which exhibit reduced perisomatic inhibitory synapses). Immunohistochemistry was used to evaluate synaptic architecture and sites, including antibodies against synaptotagmin 2 (Syt2) which selectively label inhibitory synapses. In line with in vitro studies, we found that NC1 significantly increased the distribution of Syt2+ inhibitory nerve terminals in wild type mice and rescued the loss of inhibitory synapses seen in Col19a1-/- mutant mice. These data reinforce the link Collagen XIX and inhibitory synaptogenesis and suggests that administration of this matricryptin may have thera-peutic potential.

C13

**FUNCTIONAL IMPLICATIONS OF CD8+ RESIDENT MEMORY T CELL POPULATIONS WITHIN THE CNS AFTER RECOVERY FROM ZIKA VIRUS INFECTION**

Jeremy Hill^1,2^, Shenjian Ai^1^, Allison Soung^1^, Sindhu Manivasagam^1^, Robyn Klein^1,2^

*^1^ Washington University School of Medicine, Division of Infectious Diseases, St. Louis, USA*

*^2^ Washington University School of Medicine, Department of Pathology and Immunology, St. Louis, USA*

Zika virus (ZIKV) is an emerging mosquito-borne flavivirus linked to neurological complications and memory impairment in the adult clinical population. In an adult mouse infection model, ZIKV pre-ferentially targets the hippocampus, a brain region essential for proper memory formation. In conjunction with these data, there is mounting evidence that ZIKV infection is not simply a transient infection in adult humans but rather induces a new, emerging syndrome: Zika virus-associated cognitive impairment. Virologic control of ZIKV within the central nervous system (CNS) is contingent upon proper host-viral interaction and CD8^+^ T cell during acute encephalitis. Only recently has it been established that antiviral CD8^+^ T cell populations remain in the CNS for prolonged periods of time following clearance of neurotropic viral infections. These long-lived memory T cells are termed tissue-resident memory T cells (Trm) and provide enhanced protection against reinfection. However, unbalanced local cytokine responses or re-activation of T cell populations can have serious negative cognitive consequences. To date, comprehensive analyses of specific T cell subset populations in the CNS after ZIKV infection and their functional implications in ZIKV-related cognitive impairment have not been performed. In the present study, we assessed CD8+ T cell populations after recovery from ZIKV infection by flow cytometry and correlated with outcomes on the Barnes Maze spatial memory task. Results showed ZIKV specific CD8^+^ T cell populations in poor learners are skewed to a CD103^−^ /CD69^+^ phenotype rather than CD103^+^/CD69^+^ as seen in good learners within the hippocampus after recovery. Data also suggest that within the hippocampus of poor learners, APCs have in-creased expression of B7 co-stimulatory molecules while CD8+ T cells have reduced inhibitory (PD-1, CTLA-4) molecules. These data suggest functional implications of CD8^+^ T cell interactions with local APC populations in ZIKV-related cognitive impairment post recovery.

C14

**QUERCETIN ATTENUATES LEAD-INDUCED TNF-Α AND IL-1Β NEUROINFLAMMATORY RESPONSES AND HISTOPATHOLOGY IN BRAIN OF WISTAR RATS**

Iliyasu Musa Omoyine^1^, Musa Sunday Abraham^2^, Oladele Sunday Blessing^3^, Iliya Ibrahim Abdullahi^4^

*^1^ Kogi State University, Anatomy Department, Anyigba, Nigeria*

*^2^ Ahmadu Bello University, Human Anatomy Department, Zaria, Nigeria*

*^3^ Ahmadu Bello University, Veterinary Pathology Department, Zaria, Nigeria*

*^4^ Federal University Dutse, Human Anatomy Department, Dutse, Nigeria*

The study was aimed at evaluating the attenuating effects of Quercetin on Lead-induced tumor necrosis factor-α (TNF-α) and in-terleukin-1β (IL-1β) neuroinflammatory responses and histopathology of the hippocampus and cerebellar cortex of Wistar rats. Thirty male Wistar rats of average weight of 119.00g were randomly divided into 6 groups (each, n=5). Groups I and II were administered distilled water (H_2_O: 1 ml/kg) and Lead (Pb: 125mg/kg), respectively for 42 days. Lead (Pb: 125mg/kg) was co-administered with quercetin (Q: 60 mg/kg) and Succimer (S: 10 mg/ kg) for Groups III and VI, respectively for 42 days. Groups IV and V were administered lead (Pb: 125 mg/kg) for 21 days and treated with Q: 60 mg/kg for Groups IV and S: 10 mg/kg for Group V for 21 days. The administrations were orally, once per day and lasted for 42 days. At the end of administrations, the rats were anaesthetized with Ketamine at 75mg/kg intraperitoneally and euthanized. The skull was opened, brain was harvested, weighed and divided into two halves. One half was used for histological slides and the other half was minced and homogenized in cold phosphate buffer (pH 7.4). The homogenates were centrifuged and the supernatant were obtained and used for the assessment of TNF-α and IL-1β. The results show that lead significantly increased the level of TNF-α and IL-1β in brain (p<0.05) and induced histopathology such as vacuolation and pyknotic of neurons in the hippocampus and cerebellar cortex of lead exposed rats. However, there were significant decreased in TNF-α and IL 1β and changes in histopathology of rats exposed to lead and treatment with quercetin (p<0.05). It was concluded that quercetin could be of importance in treatment and prevention against neuroin-flammation.

C15

**ELEVATED NEONATAL INTERLEUKIN-6 DRIVES BEHAVIORAL PHENOTYPES REMINISCENT OF ASD IN MICE**

Fernando Janczur Velloso, Anna Wadhwa, Ekta Kumari, Steven Levison

*Rutgers-New Jersey Medical School, Pharmacology, Physiology and Neuroscience, Newark, USA*

Epidemiologic studies have demonstrated that perinatal infections and other maternal immune challenges during pregnancy increase the risk of offspring developing neurodevelopmental disorders that include schizophrenia, autism (ASD), ADHD, Tourette disorder, depression, cerebral palsy and bipolar disorder. Animal and epidemi-ological studies point to the importance of inflammatory cytokines in inducing these behavioral changes. Notably, interleukin-6 (IL-6) injected mid-gestation can cross the placenta and the fetal blood-brain barrier, reaching the fetal CSF and reproducing behavioral deficits of MIA models. We have shown previously that IL-6 can directly affect neural stem cells (NSCs) and neural progenitors (NPs) of the subventricular and subgranular zones. Here, we have injected IL-6 into mouse pups twice daily, from P3 to P6, at a dose of 75 ng per injection, to increase IL-6 plasma levels 2-fold. This elicited a subtle but significant increase in body temperature that persisted for 2 months and was more prominent in males than females but had no effect on overall growth. Postnatal IL-6 treatment elicited behavior changes reminiscent of important symptoms in neurodevelopmental disorders. At 3 weeks of age, IL-6 injected male mice showed reduced nose-to-nose and urogenital sniffing during reciprocal juvenile play. At 6 weeks, the IL-6 injected mice were less social, as assessed by both social approach and novel social subject tests. As adults, these animals produced fewer ultrasonic vocalizations and showed reduced interaction (urogenital sniffing) during mating. Additionally, IL-6 injected male and female mice exhibited increased self-grooming and males had an increase in fear conditioning in the inhibitory avoidance task. At the cellular level, IL-6 stimulated the proliferation of a multipotential progenitor and decreased the pro-liferation of two glial restricted progenitors. Fate mapping studies revealed decreased neurogenesis in the dorsal hippocampus, reduced astrogliogenesis in the prefrontal cortex and amygdala and decreased oligodendrogenesis in the body and splenium of the corpus callosum. Altogether, these studies provide new insights into the biological mechanisms that contribute to complex neurodevelopmental brain disorders. Supported by a grant from the Governor's Council for Medical Research and Treatment of Autism awarded to SWL

C16

**T CELLS ARE CRITICAL FOR BACTERIAL CONTAINMENT DURING STAPHYLOCOCCUS AUREUS CRANIOTOMY INFECTION**

Gunjan Kak, Christopher M. Horn, Cortney E. Heim, Tammy Kielian

*University of Nebraska Medical Center, Pathology and Microbiology, Omaha, USA*

Craniotomy is a neurosurgical procedure required to gain access to the brain for tumor resection or epilepsy treatment. Infectious complications occur at a frequency of 1-3%, with approximately half caused by *Staphylococcus aureus* (*S. aureus*) that forms a biofilm on the bone flap. Our recent scRNA-seq study revealed the transcriptional heterogeneity of infiltrating leukocyte populations during *S. aureus* craniotomy infection, which included T cells and NKT cells. In the current study, intracellular cytokine staining revealed an equivalent number of Th1 and Th17 cells infiltrating the brain at days 7 and 14 post-infection, suggesting that T cells may influence inflammatory responses during *S. aureus* craniotomy infection. Bacterial burden in the brain, subcutaneous galea, and bone flap was significantly higher in RAG1 knockout (KO) compared to wild type (WT) mice at days 3, 7, and 14 post-infection, suggesting a critical role for T cells in infection containment. This was supported by findings in IFN-γR KO mice, where *S. aureus* titers were significantly elevated out to day 28 post-infection. The similar phenotypes of IFN- γR and RAG1 KO mice suggested that T cells are critical for regulating the antibacterial properties of other leukocyte and microglial subsets. To investigate this possibility, we utilized a sc-RNAseq approach, which confirmed the progressive increase in T cell recruitment into the brain during the course of infection. Hallmark pathway analysis identified a significant increase in hypoxia-and glycolysis-associated genes in the brain of RAG1 KO mice, particularly in granulocytes and microglia, with a concomitant decrease in Type I and II Interferon responsive genes (ISG, IRF, IFIT, GBP etc.). These results are suggestive of T cell crosstalk with other immune cell populations in the brain by altering their activation and metabolic states. Collectively, these findings reveal the importance of adaptive immunity in dictating the outcome of craniotomy infection by regulating the activity of infiltrating granulocytes and resident microglia.

C17

**AGGREGATION AND RELEASE OF AMYLOID-Β IN VIVO**

Rachel Hendrix^1^, Carla Yuede^2^, Woodrow Gardiner^1^, Hannah Edwards^1^, Kevin McBrearty^1^, John Cirrito^1^

*^1^ Washington University in St. Louis, Department of Neurology, St. Louis, USA*

*^2^ Washington University in St. Louis, Department of Psychiatry, St. Louis, USA*

The cleavage of the amyloid precursor protein and corresponding extracellular release of various amyloid-β (Aβ) species is a key feature of early stage Alzheimers disease (AD). It is well established that elevated neuronal activity increases release of soluble Aβ; however, a fundamental question regarding this process remains: does activity drive aggregation? The hippocampus has both a high degree of neuronal activity and is an early site of amyloid plaque pathology. Soluble and insoluble Aβ pathology spreads along specific neuronal networks in both mice and humans, such as the perforant pathway connecting the entorhinal cortex to the hippocampus. Sustained increase in excitatory synaptic strength, i.e. long-term potentiation (LTP), is impaired in the AD hippocampus. We hypo-thesize that a threshold of neuronal activity exists in which monomeric release of Aβ reaches a pro-aggregative level. Using a novel micro-immunoelectrode (MIE) allows for detection of Aβ species every 60 seconds for approximately three hours in vivo, or in living acute brain slices. MIEs utilize an antibody-attached electrode to measure the oxidation of tyrosine residues, such as those in human Aβ_40_, Aβ_42_, and Aβ dimers. The amount of current detected is proportional to the concentration of target molecule present. By altering the degree of excitation in acute brain slices and measuring the release of amyloid, kinetics can be defined on a more precise time scale. Greater understanding of these processes may enable interventions aimed at homeostatic control of hyperexcitability for prevention of the formation and spread of Aβ and downstream pathologies.

C18

**NASAL ADMINISTRATION OF GANGLIOSIDES ALLEVIATES PARKINSON'S DISEASE PATHOLOGIES IN MOUSE BRAIN**

YUTAKA ITOKAZU, TAKAHIRO FUCHIGAMI, ROBERT YU

*Augusta University, Department of Neuroscience and Regenerative Medicine, Augusta, USA*

At present, patients with Parkinson's disease (PD), and other neuro-degenerative diseases suffer from disease progression without any satisfying clinical intervention. The aggregation and accumulation of neurotoxic alpha-synuclein (aSyn) and loss of dopaminergic neurons are the major pathological hallmarks of PD. Thus, to effectively treat PD, it is necessary to eliminate cytotoxic aSyn and sustain the function of dopaminergic neurons. We harness the bioactivity of ganglio-sides, abundant in the nervous tissues and critically important in brain functional development. Gangliosides are sialic acid-containing glycosphingolipids and capable of interacting with aSyn with high affinity to stabilize an alpha-helical state and inhibit fibrillation and aggregation. Ganglioside expression profiles are known to be associated with pathogenic mechanisms in many neurological disorders. Deficiency in major brain type gangliosides (including GM1) are particularly noticeable in the brain of PD patients. In this study, intra-nasal spray of ganglioside GD3 and GM1 could reduce aSyn level and restore functional dopaminergic neurons in an A53T aSyn-overexpressing mouse (PD mouse). Furthermore, intranasal infusion of GM1 significantly enhanced expression of tyrosine hydroxylase (TH) in the substantia nigra pars compacta of the PD mouse, following the restorations of the expression and nuclear localization of, Nurr1 (also known as NR4A2), a dopaminergic neuron-associated transcription factor. GM1 induces activation of the *TH* gene transcription, including augmentation of acetylated histones and recruitment of Nurr1 to the *TH* promoter region. In this way, GM1 epigenetically regulates dopaminergic neuron specific gene expression. Our present study demonstrates that intranasal administration of gangliosides could effectively reduce neurotoxic proteins and restore functional neurons via modulating chromatin status by nuclear gangliosides. In further studies, we will apply curative gangliosides to restore functional neurons in other similar neuro-degenerative diseases that result from accumulation of misfolded toxic proteins (e.g., Alzheimer's and Huntington's diseases). Since many of these age-related neurodegenerative diseases affect an increasing number of patients, our research will benefit millions of senior citizens, their families and world society.

C19

**ISOMERIZATION OF ASP387 IN TAU IS DIAGNOSTIC FOR ALZHEIMER'S DISEASE AND REFLECTS IMPAIRED AUTOPHAGIC FLUX**

Ryan Julian^1^, Evan Hubbard^1^, Michael MacCoss^2^, Thomas Montine^3^, Lilian Heil^2^, Gennifer Merrihew^2^

*^1^ UC Riverside, Dept of Chemistry, Riverside, USA*

*^2^ University of Washington, Genome Science, Seattle, USA*

*^3^ Stanford University, Pathology, Stanford, USA*

Examination of 66 brain samples with a modified proteomics protocol revealed a striking increase in the isomerization of tau for both sporadic and autosomal dominant Alzheimer's disease (AD, ADAD) relative to controls. Importantly, roughly half of the control group exhibited high levels of amyloid plaque and neurofibrillary tangle pathology, yet isomerization of tau was statistically indistinguishable in these samples from clean controls. Further analysis revealed that tau isomerization is greater in ADAD when compared to sporadic AD. We hypothesize that autophagic flux in AD/ADAD brains is lower than that found in healthy controls. Additional data strongly support this hypothesis, including quantitative analysis of protein abundance showing that isomerization of tau positively correlates with levels of p62, a recognized indicator of autophagic inhibition. Furthermore, other proteins involved in autophagy were found to be in lower abundance in AD samples relative to controls. In conclusion, the data reveal that isomerization of tau is strongly connected with AD status and suggest strong ties between isomerization and autophagic flux, which may therefore represent a promising target for future investigations into the therapy and prevention of AD.

C20

**TAT CONJUGATED NANOPARTICLE OF ROSIGLITAZONE AND VORINOSTAT ON REVERSAL OF COGNITIVE DEFICITS IN ANIMAL MODEL OF ALZHEIMER**

Sarathlal K C, Violina Kakoty, Rajeev Taliyan

*Birla Institute of Technology and Science, Pilani (BITS Pilani), Neuropsychopharmacology, Pilani, India*

Many recent studies have investigated the role of insulin resistance in cognitive dysfunction and Alzheimer type of dementia. The transcription factor peroxisome proliferator activated receptor gamma (PPAR-γ) agonists and histone deacetylase inhibitors (HDACi) have been accounted as neuroprotective. Hence in this study we have focused to evaluate the efficacy of brain targeted poly(ethylene glycol)-poly(ε-caprolactone) (PEG-PCL) based nanoparticle of rosiglitazone (PPAR-γ agnost) and vorinostat (HDACi) in neuro-protection along with its effect on various genes implicated in memory and cognition.

Mice model of Alzheimer's type of dementia was developed with intra-hippocampal injection of beta-amyloid_1-42_. The PEG-PCL based nanoparticle of rosiglitazone (5 mg/kg) and vorinostat (25 mg/kg) was prepared by emulsion-diffusion-solvent evaporation method further the nanoparticles were linked with TAT peptide. The nano-particle and free drug combination were administered for 28 days. Neuroprotective effect of rosiglitazone and vorinostat was assessed by evaluating the expression of genes which are implicated in cognitive function, such as CREB, BDNF, GDNF and NGF with respect to internal control gene by RT-PCR method, the behavioral parameters was assessed by Morris water maze test and neuronal degeneration was estimated by histochemical analysis.

We found that combination of rosiglitazone and vorinostat up-regulated the mRNA expression of CREB, BDNF, GDNF, NGF with respect to internal control. However the nanoformulation markedly up regulated the mRNA expression than that of its free form. Further, the behavioral study and the histochemical analysis revealed significant improvement on cognitive deficits and neuronal degeneration after nanoformulation treatment.

In view of our study outcomes it is concluded that rosiglitazone and vorinostat in its nanoformulation have efficiently attenuated the cognitive deficits and neuronal degeneration in beta-amyloid induced mice model of Alzheimer type of dementia along with up-regulation of genes implicated for memory.

C21

**DISSECTING THE ROLE OF ADGRG1/GPR56 IN PERIPHERAL MYELIN DEVELOPMENT AND REPAIR**

Amit Mogha^1^, Sarah Ackerman^2^, Xianhua Piao^3^, Kelly Monk^1^

*^1^ Oregon Health and Science University, Vollum Institute, Portland, USA*

*^2^ University of Oregon, Institute of Neuroscience, Eugene, USA*

*^3^ University of California at San Francisco, School of Medicine, San Francisco, USA*

Adhesion G protein-coupled receptors (aGPCRs) are a unique sub class of GPCRs; each aGPCR possesses a large extracellular region that contains various domains potentially involved in cell-cell and/or cell-matrix adhesion in addition to the classical 7-transmembrane (7TM) region that is involved in cell signaling. aGPCRs represent the second largest GPCR family, but remain significantly understudied; however, mounting evidence demonstrates that aGPCRs are critical regulators of nervous system development and repair. We previously demonstrated that one such receptor, ADGRG1 (previously Gpr56), plays important roles in central and peripheral nervous system myelin development and maintenance [Ackerman *et al.,* 2015, 2018*;* Giera *et al.,* 2015, 2018]. In the peripheral nervous system (PNS), global *Adgrg1* knockout mice develop various defects during development and adulthood including radial sorting defects, defects in myelin ultrastructure and domain organization, and progressive neuropathy-like symptoms with age. It is unclear if Adgrg1 functions in Schwann cells, axons, or both cell types; thus, it is critical to investigate cell type-specific functions of ADGRG1. To this end, we have generated Schwann cell and sensory neuron specific conditional knock out mouse lines of *Adgrg1* (*Adgrg1*^*Floxed*^*; P0*^*Cre*^ and *Adgrg1*^*Floxed*^*; Nav1.8*^*Cre*^, respectively). Our preliminary data suggest that ADGRG1 has distinct functions in Schwann cells and sensory neurons. Our ongoing efforts to dissect cell-specific functions and to investigate possible ligands for ADGRG1 in the PNS will be discussed.

C22

**CHANGE IN LEVELS OF EXPRESSION AND FUNCTION OF EAAT1 IN GLIAL CELLS BY EXPOSURE TO METHYLMERCURY**

Andrea Ocharan, Arturo Ortega

*Centro de Investigación y de Estudios Avanzados del Instituto Politécnico Nacional, Toxicología, Ciudad de Mexico, Mexico*

Glutamate (Glu) is the main excitatory neurotransmitter in the central nervous system (CNS) and exerts its actions by activating specific plasma membrane receptors and transporters. Under physiological conditions, extracellular levels of glutamate are regulated by a family of sodium-dependent excitatory amino acid transporters (EAAT). Most of glutamate uptake is carried out by glia glutamate transporters in order to recycle this neurotransmitter through the so-called glutamate/glutamine shuttle. Exposure to environmental pollutants such as methylmercury (MeHg), has been linked to cognitive deficits in children. Taking into consideration that MeHg readily permeates the blood brain barrier (BBB) and that glia cells outnumber neurons and their end feet is part of the the referred barrier, in this contribution we explored the effect a MeHg acute exposure in the functional expression of GLAST. To this end, we used the established model of chick cerebellar Bergmann glia cultures. A substantial increase of [3H]-D-aspartate (used as a non-metabolizable glutamate analog) uptake was present upon the exposure of the cultured cells to non-cytotoxic nanomolar MeHg concentrations. These results suggest that MeHg neurotoxicity has been underestimated and provide a biochemical support to the reduction in the World Health Organization (WHO) permissive exposure limit.

C23

**A PANEL OF MICE DEMONSTRATING WIDELY DIFFERENT BUT STABLE LEVELS OF CNS MYELIN.**

Alan Peterson^1^, Hooman Bagheri^1^, Celia Jarweh^1^, Amanda Hadwen^1^, Jiarui Ao^1^, Lawrentiu Oprea^1^, Anmar Khadra^1^, Ellis Cooper^1^, Vladimir Grouza^1^, David Rudko^1^, Rachel Pryce^2^, Pierre Chaurand^2^, Amanda Young^3^, Marie Bechler^3^, Matthew Swire^4^, Andrew Jarjour^4^, Lise Cougnaud^5^, Dajana Vuckovic^5^, Amin Sherafat^10^, Akiko Nishiyama^10^, Anouk Benmamar-Badel^6^, Trevor Owens^6^, Ahdeah Pajoohesh-ganji^7^, Robert Miller^7^, Jinyi Zhang^8^, Katherine Siminovitch^8^, Claire Guerrier^9^, Hana Friedman^1^

*^1^ McGill University, Human Genetics, Oncology, Neurology & Neurosurgery, Montreal, Canada*

*^2^ Universite de Montreal, Chemistry, Montreal, Canada*

*^3^ SUNY Upstate Medical University, Cell and Developmental Biology, Saracuse, USA*

*^4^ University of Edinburgh, Regenerative Medicine, Edinburgh, Scotland*

*^5^ Concordia University, Chemistry and Biochemistry, Montreal, Canada*

*^6^ University of Southern Denmark, Neurobiology Research, Odense, Denmark*

*^7^ George Washington University, Anatomy and Cell Biology, Washington DC, USA*

*^8^ University of Toronto, Lunenfeld-Tanenbaum Research Institue, Toronto, Canada*

*^9^ Universite Cote d'Azur, NeuroMod Institue, Nice, France*

*^10^ University of Connecticut, Phyisiology and Neurobiology, Storrs, USA*

To investigate the functional organization of *Myelin Basic Protein (Mbp) r*egulatory sequence, we mutated *Mbp* enhancers and derived 7 different mouse lines. These mutants demonstrate stable, variably reduced or augmented *Mbp* mRNA accumulation^1^. As MBP is necessary and limiting for CNS myelin formation and maintenance^2,3,4^, such mice demonstrate a range of stable hypomyelination thus providing opportunities to investigate MBP functions during myelin sheath formation and secondary changes arising from CNS hypomyelination. Axons of appropriate diameter are ensheathed, albeit with thin myelin, but in one line, myelinated fibres demonstrate anomalous profiles while small to medium caliber axons remain amyelinated. Secondary consequences extend to myelin proteins and lipids and an altered density and state of multiple glial populations. The MBP Hypomyelination Consortium hopes that the characterization of the models reported here will facilitate their application to diverse questions in myelin biology. 1: Bagheri *et al*., 2020 PlosGenetics; 2: Roach et al., Cell. 1983; 3: Meschkat *et al.,* 2020, BioRxiv; 4: Shine et al., J Neurochem. 1992

C24

**COMPARISON OF COGNITIVE, SENSORY AND MOTOR FUNCTION BETWEEN WILD-TYPE AND SYSTEM XC-NULL MICE**

Shannon Pitt, Sandra Hewett, Carla Frare

*Syracuse University, Biology, Syracuse, USA*

Alterations in Excitatory/Inhibitory (E/I) balance contribute to the pathobiology of neurological diseases (e.g. stroke, epilepsy and traumatic brain injury) and neurodevelopmental disorders (e.g. autism and schizophrenia) all of which are associated with cognitive and motor impairments. Recent evidence from our lab demonstrates that loss of the heterodimeric plasma membrane amino acid cystine/glutamate antiporter, system x^−^ (Sx^−^), leads to E/I imbalance.

Specifically, we find that both male and female Sx_c_^−^ null mice (SLC7a11^sut/sut^ C3H/HeSnJ) have a reduction in their convulsive seizure threshold upon acute systemic administration of the chemo-convulsant kainate, as compared to their wild-type littermates (SLC7A11^+/+^ C3H/HeSnJ). Whether this shift towards excitation is accompanied by cognitive, sensory, and/or motor behavioral deficits remains unknown. To determine this, we compared 8-9 week old male and female SLC7a11^sut/sut^ mice with their age-and sex-matched SLC7A11^+/+^ littermates using the accelerating rotor-rod to test motor strength/coordination and motor learning as well as pre-pulse Inhibition (PPI) of the acoustic startle response, a measure of sensorimotor gating theorized to contribute to cognitive organization. With respect to rotor-rod, there was no statistical genotypic difference between male or female SLC7A11^+/+^ (n=13M,16F) and SLC7a11^sut/sut^ (n=18M,10F) mice with respect to motor coordination or motor learning. Specifically, the latencies to fall on acceleration of the rod did not differ nor did the increase in time to fall demonstrated over 4 days of testing performed every other day [two-way repeated measures ANOVA]. Likewise, pre-pulse inhibition was similar between male SLC7A11^+/+^ and SLC7a11^sut/sut^ (n=8/genotype; two-way repeated measures ANOVA), indicating that mice null for system x_c_^−^ had no deficit in sensorimotor gating. Despite this, the acoustic startle amplitude of male SLC7a11^sut/sut^mice was statistically higher than that of their sex-matched SLC7A11^+/+^ littermates (two-tailed t-test, p<0.0413). Analysis of females is on-going. Since, E/I balance regulates the threshold of startle responses, the shift toward excitation by the system x_c_^−^ mutation may explain this enhancement of startle. Supported by R01NS105767-03 to SJH.

C25

**ACUTE LOSS OF PROLIFERATING NG2+ CELLS AFTER SPINAL CORD INJURY CHRONICALLY ALTERS SCAR FORMATION AND AXON GROWTH**

Christina Marion^1^, Ping Wei^1^, Dana McTigue^1,2^

*^1^ Wexner Medical Center, Ohio State University, Department of Neuroscience, Columbus, USA*

*^2^ Ohio State University, Belford Center for Spinal Cord Injury, Columbus, USA*

The extent to which NG2^+^cells contribute to glial and fibrotic scar formation after spinal cord injury (SCI) is poorly understood. To selectively ablate dividing NG2^+^ cells responding to SCI, we utilized a novel transgenic mouse line in which cells expressing NG2 also express a thymidine kinase from herpes simplex virus (NG2-tk mice). Intraventricular administration of antiviral agent ganciclovier (GCV) causes apoptosis in dividing NG2^+^cells (including oligodendrocyte progenitors and pericytes). Immediately following unilateral white matter-specific C5 SCI in NG2-tk mice, a drug delivery pump was placed subcutaneously with attached catheter inserted into the ventricle to administer GCV or saline for 14 days. Our prior work showed ablation of NG2^+^ cells significantly altered density and distribution of glial and fibrotic scars through 21d post-injury. Compared to controls, these alterations prolonged hemorrhage, enhanced edema, and impaired forelimb recovery, but also increased axons entering the lesion area (Hesp et al., 2018). The present study assessed long-term consequences of acute NG2^+^cell ablation. Drug pumps were removed after 14 days, allowing a recovery period before assessment at six-or eight-weeks post-SCI. In contrast to earlier time points, overall density of GFAP in the glial scar and laminin within the fibrotic scar did not significantly differ between NG2^+^ cell-ablated and non-ablated mice. However, there were differences in scar density and patterning, suggesting altered recovery after GCV cessation. A unique pattern emerged in the gray matter adjacent to the lesion, with significantly increased laminin profiles resembling blood vessels in NG2^+^ cell-ablated mice at both time points. Consistent with acute findings, more axonal profiles were maintained within lesions in GCV-treated mice. Iimpaired motor recovery also persisted through 8w post-injury. Clarifying the roles of NG2+ oligodendrocyte progenitors versus pericytes will be important to further define the contributions of each cell type to beneficial and deleterious cellular responses to CNS injury.

C26

**THE ROLE OF NOS3 EXPRESSION IN MICROGLIA RESPONSE DURING ISCHEMIA IN WHITE MATTER**

Hung Nguyen, Selva Baltan

*Oregon Health & Science University, Anesthesiology & Perioperative Medicine, PORTLAND, USA*

White matter (WM) damage after ischemic injury contributes significantly to neurological impairments. In grey matter, microglia play a dual function after ischemic injury: exacerbating injury through pro-inflammatory markers and promoting neuroprotection. Nitric oxide synthase (NOS) activation contributes to oxidative damage in ischemia. The expression of NOS isoforms in WM is cell-specific and age-dependent. Particularly, microglia express NOS3 in young WM, but both NOS2 and NOS3 isoforms in aging WM. Inhibition of NOS3 exerts functional protection against ischemia in young and aging axons. However, whether this protective effect is mediated through microglial activation has not been investigated. We hypothesize that ischemic injury alters microglial activity and morphology in WM, and that NOS3 inhibition preserves microglial activity and function, which contribute to axonal protection against ischemic injury.

We used isolated Cx3Cr1-GFP mouse optic nerves (MONs), a pure WM tract, to visualize and quantify the morphological changes of GFP (+) microglia in a model of *in vitro* ischemia. Confocal live imaging of green fluorescent microglia was captured and quantified at baseline and during 1h of oxygen-glucose deprivation (OGD) with or without L-NIO, a NOS3 inhibitor. In males, OGD altered ∼90% of microglial dynamics as quantified by a series of morphological parameters, including the complexity of processes, size, shape, and area covered by microglia. Application of L-NIO attenuated the percentage of microglial changes to ∼25%. Interestingly, in females OGD caused alterations in only ∼43% of microglia, and application of L-NIO did not improve these changes.

Our results show that microglia in WM undergo significant morphological changes during ischemia, but the percentage of microglia that responds to ischemia is higher in males compared to females. Subsequently, NOS3 inhibition attenuated responsive microglia more in males. This study raises the question of how microglial activation participates in ischemic injury and axon function recovery in male vs. female WM, and whether NOS isoform expression in microglia of male and female WM regulates axon function recovery.

C27

**NEUROENDOCRINE, COGNITIVE, AND BEHAVIORAL CO-MORBIDITIES IN HIV-1 TAT TRANSGENIC MALE MICE ARE AUGMENTED BY AGING**

Alaa Qrareya^1^, Fakhri Mahdi^1^, Nicole Ashpole^1^, Jason Paris^1^, Marc Kaufman^2^

*^1^ University of Mississippi, Department of Biomolecular Sciences, University, USA*

*^2^ McLean Imaging Center, Department of Psychiatry, McLean Hospital/Harvard Medical School, Belmont, USA*

In the era of antiretroviral therapies, secondary hypogonadism remains a common problem among young and middle-aged HIV-1- infected men (∼25%) and its prevalence increases with age. In the U.S, ∼51% of HIV-infected individuals are 50 years of age or older. Although the underlying mechanisms are poorly understood, HIV-1 proteins, such as the trans-activator of transcription (Tat), may contribute to hypothalamic-pituitary-gonadal axis dysfunction partly via the promotion of mitochondrial toxicity and disrupted steroidogenesis. In this study, we hypothesized that conditional Tat expression in middle-aged male transgenic mice [Tat(+)] would promote cognitive impairment, affective-like and neuromuscular dysfunction, and mechanical allodynia, compared to age-matched controls [Tat(-)]. We further expected that Tat expression would alter the steroid hormone milieu resulting in abnormalities that would correlate with behavioral deficits. Age and/or Tat exposure altered steroid hormones wherein middle-aged Tat(+) mice exhibited greater circulating corticosterone, lower circulating testosterone (T), and increased allopregnanolone in the midbrain and hippocampus. In young adults, Tat exposure accelerated the progesterone increase observed in middle-age and also increased circulating estradiol (E_2_) and the E_2_:T ratio. Older age or Tat exposure increased anxiety-like behavior in an open field and an elevated plus-maze, increased numbers of radial arm water maze errors, and reduced grip strength compared to young adult and Tat(-) males. Young adult Tat(+) or middle-aged Tat(-) males had higher mechanical nociceptive thresholds than their age-matched counterparts. Levels of several steroids (corticosterone, allopregnanolone, and testosterone) correlated with behavioral endpoints. Thus, HIV-1 Tat appears to be sufficient to amplify age-related disease states.

C28

**LOW-INTENSITY BLAST INDUCES MULTI-FOCAL NEURONAL AND SYNAPTIC ULTRASTRUCTURAL ABNORMALITIES WITH BEHAVIORAL DEFICITS IN MICE**

Heather Siedhoff^1^, Shanyan Chen^1^, Ashley Balderrama^1^, Runting Li^1^, Landry Konan^1^, Hailong Song^1^, Catherine E. Johnson^2^, DeAna Grant^3^, Tommi White^3^, Timothy Hoffman^6^, Jiankun Cui^1,6^, Ralph G. DePalma^5^, Zezong Gu^1,6^

*^1^ University of Missouri School of Medicine, Pathology and Anatomical Sciences, Columbia, USA*

*^2^ Missouri University of Science and Technology, Mining and Nuclear Engineering, Rolla, USA*

*^3^ University of Missouri, Electron Microscopy Core Facility, Columbia, USA*

*^4^ Mercer University, Biomedical Sciences, Macon, USA*

*^5^ Uniformed Services University of the Health Sciences, Surgery, Bethesda, USA*

*^6^ Truman VA Hospital, Research Service, Columbia, USA*

Blast-induced mild traumatic brain injury (mTBI) represents a majority of military-related neurotrauma. We report provisional insights into mechanisms of low-intensity blast (LIB)-induced patho-biology and long-term neurobehavioral deficits in mice, using the military-relevant “*Missouri Blast*” model with open-field detonation of high-energy explosive C4 (350 grams). We report effects to date of incident and ground reflected shockwaves, generating a static peak overpressure of 46.7 kPa, with maximal impulse of 60 kPa x ms at 3- m distance from 1-m above ground detonation. Transmission electron microscopy delineated LIB effects at the ultrastructural level for the neuron perikaryon, axons, and synapses of mice at 7- and 30-days post-injury (DPI). A significant increase of PSD95 and synaptophysin protein levels 7 DPI suggest potential synaptic reorganization. Alternation of vGluTs, EAATs, and glutamine synthetase expression indicate dysregulated glutamate transmission due to circuit excitability. Blast-exposed mice demonstrated significantly decreased contact duration in Social Interaction test and increased anxiety-like activity in Open Field test 3 months after LIB exposure, suggesting delayed effects of mTBI simulating blast-related human behavior. These results indicate single LIB exposure causes ultrastructural brain injury, accompanied with multi-focal subcellular alterations and long-term behavioral deficits. The “*Missouri Blast*” model provides a useful paradigm, elucidating pathogenic mechanisms of primary blast exposures in the absence of head impact or acceleration. **Support:** VA ORD BLR&D Director Service program (UFR-002-18F), Collaborative Merit TBI (I01 BX004313-01A1), DOD/CDMRP PRARP-CSRA (AZ180043), MU research funds (ZG) and MU EMC Award.

C29

**ASTROGLIOSIS AND NETWORK ALTERATIONS IN FRONTAL AND TEMPORAL CORTICES FOLLOWING DEMYELINATION-INDUCED SEIZURES**

Czarina Juan-Sing, Carrie R. Jonak, Xinghao S. Shuai, Devin K. Binder, Seema Tiwari-Woodruff

*UC Riverside, Biomedical Sciences, Riverside, USA*

Multiple sclerosis (MS) characteristically presents as a relapsing and remitting disease. There are several factors that can signal onset or progression of symptoms, notably including seizures. MS patients are three to six times more likely to develop epileptic seizures and seizure incidence can indicate sudden and severe onset and progression. Despite this, demyelination-associated seizures and hyper-excitability are poorly understood. To address this, we aim to determine the spatial and temporal anatomy of EEG changes following chronic demyelination and assess cellular markers in identified regions of seizure activity. Male mice were subjected to a normal or 12-week cuprizone (CPZ) diet in order to induce chronic demyelination. These same animals were implanted with planar 30- contact multi-electrode arrays (MEA) and monitored for spontaneous electrographic seizure activity. The left temporal and left frontal cortices demonstrated synchronized seizure activity in 12-week CPZ animals. We then assessed neuronal activation, inhibitory networks and glial involvement in these left temporal and prefrontal cortex using immunohistochemistry to determine whether changes in cell population and circuitry were correlated with epileptic hyperactivity. Chronically demyelinated animals demonstrated increased astrogliosis and c-fos activation in areas of seizure activity. Additionally, while parvalbumin neuron populations were decreased in the cortex, the existing parvalbumin neurons showed increased fiber projection, suggestive of cortical remodeling. Our data suggests that increased cellular activity and alterations in inhibitory and glialnetworks may be associated with demyelination-associated seizures. By understanding how these inhibitory and excitatory networks change in response to demyelination, we can better understand seizure generation in MS.

C31

**EXTRACELLULAR VESICLES AS NANOCARRIERS OF APOTRANSFERRIN IN DEMYELINATING DISEASES**

Vanesa Mattera^1^, Pehuen Pereyra Gerber^2^, Matias Ostrowski^2^, Juana Pasquini^1^, Jorge Correale^3^

*^1^ Instituto de Química y Fisicoquímica Biológica(IQUIFIB), Química Biológica Patológica, Buenos Aires, Argentina*

*^2^ Instituto de Investigaciones Biomédicas en Retrovirus y SIDA (INBIRS), Departamento de Microbiología, Buenos Aires, Argentina*

*^3^ Instituto de Investigaciones Neurológicas Dr. Raúl Carrea (FLENI), Neuroinmunología y enfermedades desmielinizantes, Buenos Aires, Argentina*

Extracellular vesicles (EVs) are lipid bilayer-delimited particles which are fundamental for intercellular communication. EVs capacity for biologic information transfer makes them an attractive tool as therapeutic agents for nanodelivery of different molecules to target cells. They are able to cross the blood brain barrier, their structure is biocompatible, and the cargo is protected from degradation, which increases molecule stability, solubility and bio-availability.

Previous work by our group has shown the pro-differentiating effects of apoTransferrin (aTf) on oligodendroglial cells in vivo and in vitro. The remyelinating effect of aTf was also observed in a model of hypoxia/ischemia where aTf was intranasally administered. In this scenario, the purpose of this work is to establish the effect of nano-particles loaded with human aTf in a model of demyelination induced by cuprizone (CPZ).

EVs were isolated from mouse plasma and characterized by Western blot, dynamic light scattering and scanning electron microscopy. Isolated EVs containing the Tf receptor 1 (TfR1) were loaded with aTf, specifically through binding to TfR1, using two passive cargo-loading strategies which rendered EV-Tf loaded particles. In this work, we show the promaturation effect of the EV-Tf system on oligodendroglial primary cultures and its remyelinating effect through intranasal administration in CPZ-demyelinated mice, as assessed through different cell markers and myelin staining.

These results show EVs as potential nanovehicles of aTf to be delivered into the CNS parenchyma and pave the way for further studies into their possible clinical application in the treatment of demyelinating diseases.

C32

**DEGENERATIVE AND ADAPTIVE CHANGES IN THE RESPIRATORY SYSTEM OF NEUROPATHIC MICE**

Malavika Nair, Jie Zhao, Hannah Bazick, Allison Franklin, Alexa Mealy, Lucia Notterpek

*University of Florida, Department of Neuroscience, Gainesville, USA*

Though peripheral neuropathies normally refer to weakness of the extremities, respiratory complications have also been documented in Charcot-Marie-Tooth disease type 1A (CMT1A) and Dejerine-Sottas disease (DSS) patients. Heterozygous Trembler J (TrJ) mice are an established model of severe hereditary demyelinating neuropathies as they carry the same Leu16Pro mutation in PMP22 which is found in DSS patients. Our previous studies of 2-9 month old TrJ mice indicate severe phrenic nerve degeneration with significant demye-lination, and a concurrent hypertrophy of diaphragm muscle fibers. To investigate the underlying mechanism for this unexpected phenotype, we performed immunohistochemistry and biochemical analyses of diaphragm tissues from 5-9 month male and female Wt and TrJ mice. Immunolabeling with anti-actinin antibodies showed intact Z-lines in affected diaphragms from 2 month old mice, suggesting the preservation of functional contractile units at this age. Western blot analyses of whole diaphragm tissue lysates demonstrated significant (p<0.01) upregulation of the ubiquitin-proteasome pathway and an increase in the autophagy marker LC3II. Immuno-histochemistry on frozen diaphragm tissue sections confirmed the biochemical results, as samples from neuropathic mice displayed increased reactivity with antibodies against ubiquitin and the lysosomal membrane protein 1 (LAMP1). As a follow up to the phrenic nerve degeneration, we also examined spinal cord cross sections from vertebrae C3-C5 of 4-month old male and female Wt and TrJ mice. Double immunolabeling with cell type specific markers indicated astrogliosis in samples from TrJ mice. Atypical neurofilament whirls were also seen in occasional ventral motor neurons, demonstrating ongoing CNS pathology in phrenic neuropathy. Together, our results indicate pathological degenerative events in both PNS and CNS of neuropathic mice, co-occurring with adaptive changes in the diaphragm. Further investigation of the respiratory complications in neuropathic rodent models will contribute towards identifying the appropriate tissue targets to improve human patient quality of life.

C33

**VITAMIN D SUPPLEMENTATION RESCUES ABERRANT NF-KB PATHWAY ACTIVATION AND AMELIORATES RETT SYNDROME NEURONAL MORPHOLOGY AND BEHAVIOR**

Mayara Ribeiro, Jessica MacDonald

*Syracuse University, Biology, Syracuse, USA*

Rett syndrome (RTT) is an X-linked, severe neurodevelopmental disorder caused by mutations in the transcriptional regulator *MECP2*. Previous research found that in the absence of *Mecp2*, NF-kB signaling is increased in the CNS of mice. Genetically attenuating the aberrant NF-kB activation ameliorates several hallmark phenotypes of RTT, including neuronal morphology, identifying the NF-kB pathway as a potential therapeutic target for RTT. Among the known inhibitors of NF-kB signaling is vitamin D (VitD). Interestingly, VitD deficiency is prevalent in RTT patients, and we have found that male *Mecp2*-null mice have significantly lower serum levels of VitD (25(OH)D). Thus, we aimed to investigate whether VitD supplementation can reduce NF-kB signaling and improve RTT phenotypes. We find that VitD lowers NF-kB activity and promotes neurite outgrowth of *Mecp2*-knockdown cortical neurons *in vitro*. To investigate whether VitD has therapeutic benefit *in vivo*, we placed *Mecp2*-null male and *Mecp2* heterozygous female (*Mecp2*+/-) mice, and their wildtype littermates, on custom chow containing 1IU/g (control), 10IU/g, or 50IU/g VitD at 4 weeks of age. Both *Mecp2*- null male and heterozygous female mice on the supplemented diet display increased dendritic complexity and soma area when compared to *Mecp2* mutant mice on the control chow at symptomatic ages, 4 and 20 weeks of age, respectively. Additionally, *Mecp2*+/- mice on 10IU/g VitD supplementation demonstrate improved motor coordination and decreased anxiety-like behavior compared to *Mecp2*+/- mice on control 1IU/g VitD. To investigate how VitD ameliorates RTT phenotypes, we performed RNA-seq analysis on total cortex of a subset of female mice that underwent behavior assays. We identified over 200 genes that are differentially expressed in *Mecp2*+/- mice, compared to wildtype, on 1IU/g VitD but that are no longer significantly different in *Mecp2*+/- mice on 10IU/g VitD. These rescued differentially expressed genes are involved, among other things, in neuronal morphology, and thus could underpin the behavioral and morphological rescue observed with VitD. Thus, our data indicate that VitD could be a simple and cost-effective partial therapeutic avenue for RTT.

C34

**DIVERSE GABAERGIC NEURONS ORGANIZE INTO SUBTYPE-SPECIFIC LAMINAE IN THE VENTRAL LGN**

Ubadah Sabbagh^1,2^, Jessica Wei^3^, Ryan Ha^1^, Rachana Somaiya^1,2^, Michael Fox^1,4,5^

*^1^ Fralin Biomedical Research Institute at VTC, Center for Neurobiology Research, Roanoke, VA, USA*

*^2^ Virginia Tech, Graduate Program in Translational Biology, Medicine, and Health, Blacksburg, VA, USA*

*^3^ Fralin Biomedical Research Institute at VTC, neuroSURF program, Roanoke, VA, USA*

*^4^ Virginia Tech, Biological Sciences, Blacksburg, VA, USA*

*^5^ VTC School of Medicine, Pediatrics, Roanoke, VA, USA*

In the visual system, retinal axons convey visual information from the outside world to dozens of distinct retinorecipient brain regions. In rodents, two major areas that are densely innervated by this retinal input are the dorsal lateral geniculate nucleus (dLGN) and ventral lateral geniculate nucleus (vLGN), both of which reside in the thalamus. The dLGN is well-studied and known to be important for classical image-forming vision. The vLGN, on the other hand, is associated with non-image-forming vision and its neurochemistry, cytoarchitecture, and retinothalamic connectivity all remain unresolved, raising fundamental questions of its role within the visual system. Here, we sought to shed light on these important questions by studying the cellular landscape of the vLGN and map its connectivity with the retina. Using *in situ* hybridization, immuno-histochemistry, electrophysiology, and genetic reporter lines, we discovered at least six transcriptionally distinct subtypes of inhibitory neurons that are distributed into distinct adjacent sublaminae. Using trans-synaptic viral tracing and in vitro electrophysiology, we found that cells in each these sublaminae receive direct inputs from retina. Lastly, by genetically removing this visual input to the vLGN, we found that the organization of these sublaminae is dramatically disrupted, suggesting a crucial role for sensory input in the cyto-architectural maintenance of the vLGN. Taken together, these results not only identify novel subtypes of vLGN cells, but they also point to new means of organizing visual information into parallel pathways – by anatomically creating distinct sensory channels. This subtype-specific organization may be key to understanding how the vLGN receives, processes, and transmits light-derived signals in the subcortical visual system.

C35

**16P11.2 DELETION AND GESTATIONAL IRON DEFICIENCY ALTER DEVELOPMENT OF HUMAN VENTRAL FOREBRAIN ORGANOIDS**

Garrick Salois, Margot Mayer-Proschel

*University of Rochester, Neuroscience, Rochester, USA*

The prevalence of Autism Spectrum Disorder (ASD) diagnoses has been rapidly increasing over the last several decades. Numerous genetic and environmental risk factors for ASD have been discovered, however it remains unclear how these risk factors interact and converge upon common developmental pathways to generate aberrant phenotypes. Heterozygous deletion of the 16p11.2 copy number variant is associated with increased risk for ASD diagnosis as well as iron deficiency anemia, potentially due to a reduction in BolA2, a protein involved in Fe-S cluster formation. In order to investigate how iron deficiency and 16p11.2 deletion affect early neurodevelopmental events in a human system, we cultured 16p11.2 deletion patient-derived induced pluripotent stem cells, and an Nkx2.1:GFP embryonic stem cell line to generate ventral forebrain organoids (VFOs). Using immunofluorescence, we have demonstrated the establishment of cells expressing markers of mature inhibitory neurons, as well as transcription factors associated with the development of these cell types, which maintain a spatiotemporal organization which closely models that seen in the developing human brain. Preliminary results demonstrate that this organization is modulated by iron deficiency in Nkx2.1:GFP VFOs. Furthermore, we demonstrate that early embryoid bodies maintain equivalent size across conditions at early timepoints but begin to diverge when region-specific morphogens are applied, at which point 16p11.2 deletion organoids demonstrate an overall gross increase in organoid volume. This result contradicts findings previously reported from animal models of 16p11.2 but is consistent with observations of macrocephaly in human 16p11.2 deletion patients.

C36

**ALLOPREGNANOLONE EFFECTS ON INHIBITION IN INTERNEURONS**

Xinguo Lu^1^, Charles Zorumski^1,2,3^, Steven Mennerick^1,2,3^

*^1^ Washington University in St. Louis, Psychiatry, St Louis, USA*

*^2^ Washington University in St. Louis, Neuroscience, St Louis, USA*

*^3^ Washington University in St. Louis, Taylor Family Institute for Psychiatry Research, St Louis, USA*

Inhibition sculpts neurophysiological excitability and modulates brain oscillations that give rise to sensation, perception, and cognition. Inhibitory neurotransmission is largely mediated by GABA_A_ receptors in mammalian central nervous system. GABA_A_ receptors are heteropentameric and composed of two α, two β, and a fifth subunit, usually either δ (less common) or γ2 (most common). Besides releasing GABA to activate GABA_A_ receptors on projection cells, GABAergic interneurons themselves express GABA_A_ receptors and receive phasic and tonic inhibition from other interneurons. Allopregnanolone (AlloP) is a neurosteroid recently approved for treating postpartum depression in women. Past evidence suggests that AlloP may selectively target δ-containing GABA_A_ receptors.

Previously, we utilized novel pharmacoresistant GABA_A_ receptor subunits to test the role of α4δ-containing receptors, a dentate granule cell (DGC) subunit combination, in mediating AlloP effects. In contrast to more extensively studied DGCs, hippocampal fast-spiking parvalbumin-positive (PV+) interneurons express an unusual variant partnership of δ subunits with α1 subunits. Here we hypo-thesized that native α1δ receptors may provide a strong substrate for AlloP, resulting in potentiation of tonic current. Contrary to the hypo-thesis, electrophysiology from genetically tagged PV+ interneurons in hippocampal slices showed that 100 nM AlloP promoted phasic inhibition by increasing the sIPSC decay, but tonic inhibition was not detectably altered. Co-application of 100 nM AlloP with 5 µM GABA did result in tonic current augmentation. Our results indicate that AlloP principally promotes phasic current but may disinhibit in-terneurons through tonic inhibition under conditions of high ambient GABA. This differs from effects on nearby DGCs in which AlloP significantly promoted tonic current even in the absence of exogenous ambient GABA.

C37

**CELLULAR AND SYNAPTIC THALAMIC ALTERATIONS IN A MOUSE MODEL OF DRAVET SYNDROME**

Carleigh Studtmann, Sharon Swanger

*Virginia Tech, Fralin Biomedical Research Institute, Roanoke, USA*

Dravet Syndrome (DS) is an infantile epileptic encephalopathy caused by mutations in the voltage-gated sodium channel Na_v_1.1, which leads to hyperexcitable brain activity during development. This altered excitability causes persistent dysfunction across brain circuits, resulting in a broad phenotypic profile including non-convulsive (absence) seizures, attention deficits, and sleep disruption. Corticothalamic (CT) circuits are responsible for diverse informa-tional processing and play a particularly important role in regulating attention and sleep. Disrupted CT circuit activity contributes to a variety of phenotypes in DS models and thus offers a promising therapeutic target. However, the precise cellular and synaptic mechanisms underlying CT circuit dysfunction remain elusive. Here, we sought to identify the cellular and synaptic mechanisms underlying pathological somatosensory thalamic circuit activity in a DS mouse model, thereby revealing therapeutic targets to correct circuit function. Electrophysiology results reveal the first evidence of synaptic-level alterations in the somatosensory thalamus of a DS mouse model, including a significant reduction in the glutamatergic synaptic input to the ventrobasal (VB) thalamus and a significant increase in gabaergic input to the same region. Interestingly, synaptic input to the reticular nucleus of the thalamus (nRT), an inhibitory neuron population that forms reciprocal connections with the VB, remains unaltered. Furthermore, nRT neuron intrinsic excitability was altered in this DS mouse model including significant changes in resting membrane potential and depolarization-induced firing response. These data indicate that diverse synaptic and cellular-level changes in multiple neuron populations may contribute to somatosensory thalamic dysfunction in this DS model, providing ample opportunity for therapeutic intervention.

C38

**FUNCTIONAL ANALYSIS OF VALENCE AND VARIANCE IN UBE3A IN THE CONTEXT OF NEURODEVELOPMENTAL DISEASE**

Kellan Weston, Xiaoyi Gao, Jinghan Zhao, Jason J. Yi

*Washington University in St. Louis, Neuroscience, St. Louis, USA*

UBE3A is a HECT (homologous to E6AP C-terminus) domain E3 ubiquitin ligase that targets substrate proteins for degradation through the ubiquitin-proteasome pathway. The UBE3A gene is imprinted exclusively in neurons such that only the maternal allele is expressed. Precise deletion or null mutation of the maternal copy of UBE3A causes a severe form of intellectual disability known as Angelman syndrome. In contrast, duplication or triplication of maternal 15q11- 13, the chromosomal location within which UBE3A resides, is linked to a prevalent syndromic form of autism known as Dup15q syn-drome. This observation suggests that excessive UBE3A activity may be causative for autism phenotypes in Dup15q syndrome; however, evidence for this hypothesis remains sparse. In order to identify if precise, hyperactivating mutations in UBE3A are sufficient to drive disease, we devised a high-throughput assay to screen the functional consequence of UBE3A missense variants. We screened over 150 variants and identified distinct functional classes of UBE3A mutants based on their effect on enzymatic activity. Importantly, we identified over a dozen novel gain-of-function variants that aberrantly hyperactivate UBE3A enzyme activity. We confirm that UBE3A hyperactivation is sufficient to drive neurode-velopmental disease phenotypes in humans, and mice carrying a specific hyperactivating mutation in UBE3A exhibited aberrant motor and communication defects. Finally, mapping the results of our screen to the UBE3A protein structure revealed an unknown allosteric regulatory site within the catalytic domain that acts as a charge-dependent regulator of enzyme activity. Altogether, our study provides conclusive evidence that bi-directional changes in UBE3A activity are sufficient to cause distinct neurodevelopmental outcomes, and moreover, sheds new light on the mechanisms that regulate UBE3A activity.

C39

**ANTICANCER ACTIVITY OF CARDIAC GLYCOSIDES AGAINST HUMAN GLIOBLASTOMA CELL LINE U87**

Sumbul Zehra, Shabana Usman SIMJEE

*International Center for Chemical and Biological Sciences, Molecular Medicine, Karachi, Pakistan*

Glioblastoma multiforme (GBM) is considered as most invasive cancer which accounts for approximately 54% among different types of brain cancers. It is extremely aggressive, with poor prognosis and highly resistant to many anticancer drugs. The median overall survival of only 11 months has been reported in the general GBM population with standard GBM therapies. GBM relapse is very common and a challenge for its treatment. Therefore, there is dire need to develop new drug having effective therapeutic potential for GBM. The purpose of the present study was to evaluate cardiac glycosides on human glioblastoma cells U87. Cardiac glycosides are present in plants and used for their important medicinal properties. They have been formerly used as diuretic, abortifacient, emetic and as a heart tonic. Earlier we have found a potent anticancer activity of these compounds on human oral cancer cell line cal27. Since GBM is considered as a resistant tumor with low rate of remediation, therefore we decided to evaluate the activity of these compounds on human U87 cell line. MTT assay was used to determine the cytotoxic effect of these compounds. The calculated % growth inhibition revealed a significant inhibition of the U87 cells following the treatment in a dose dependent manner. Treatment of U87 cells with these compounds was done for 24h. The IC_50_ of the compounds was found to be at 17µM and 2µM dose. Reactive oxygen species were also observed at these IC_50_ doses. Moreover, wound healing assay was performed which further evaluated their role as anticancer agents. These observations showed the potential of these compounds as potent anticancer agents against U87 cells and suggest further research for the development of anticancer drug using these cardiac glyco-sides for the treatment of human glioblastoma.

C40

**DECIPHERING INFLAMMATORY CHANGES ELICITED BY CRANIOTOMY SURGICAL DAMAGE THAT PROMOTE STAPHYLOCOCCUS AUREUS BIOFILM FORMATION**

Lee Korshoj, Tammy Kielian

*University of Nebraska Medical Center, Pathology and Microbiology, Omaha, USA*

Craniotomy is a neurosurgical procedure involving the removal and replacement of a skull fragment (bone flap) to access the brain. Despite precautions, infection occurs at rates of 1-3% with about half due to *Staphylococcus aureus* forming a biofilm on the bone flap. Infections carry significant morbidity, often requiring the bone flap to be discarded concomitant with long-term antibiotic therapy. This study aimed to understand how the release of damage-associated molecular patterns (DAMPs) from tissue following a craniotomy contributes to biofilm formation and infection persistence. A mouse craniotomy model was used to compare spatial and temporal attributes after sterile sham surgery vs. *S. aureus* infection. Flow cytometry revealed the differential influx of leukocyte subsets into the brain, subcutaneous galea, and bone flap. Monocytes were the major leukocyte population recruited to the brain, whereas granulocytic myeloid-derived suppressor cells (G-MDSCs) and neutrophils were most numerous in the galea and bone flap. These patterns were similar for sham and infected mice at three days post-surgery, even in the presence of significant bacterial burdens (10^5^-10^6^ cfu) suggesting that surgical damage alone is a major driver of the acute immune response. However, at later time points (days 7-28) there was a divergence between groups, notably in the galea, where monocytes were enriched in sham animals but G-MDSCs remained elevated in the infected group. Quantification of inflammatory mediators revealed significant increases in pro-inflammatory cyto-kines and chemokines in the brain and galea of infected mice including IL-1β, TNF-α, IL-6, CXCL9, and CXCL10. As a followup, *in vitro* metabolic analyses of microglia co-cultured with live *S. aureus* biofilm showed preferential upregulation of oxidative phos-phorylation with minimal change in glycolysis, indicating that bio-film elicits an anti-inflammatory phenotype supporting infection persistence. Collectively, these results suggest DAMPs released following craniotomy mask the infection by inducing a wound healing response promoting biofilm formation. Once formed, the biofilm can metabolically reprogram microglia leading to its persistence. Supported by the NIH National Institute of Neurological Disorders and Stroke (R01NS107369).

C41

**CEREBELLAR MICROGLIAL ACTIVATION BY AN ACUTE EXPOSURE TO MANGANESE CHLORIDE (MNCL2)**

María Isabel Hernández, Luisa Hernandez-Kelly, Libia Vega, Arturo Ortega

*Centro de Investigación y de Estudios Avanzados del Instituto Politécnico Nacional, Toxicología, Gustavo A Madero, Mexico*

Microglia plays an important role in the neurotoxicity of various xenobiotics, within which highlights the metals, a clear example is the toxicity caused by manganese. Acute intoxication with manganese leads to a clinical feature similar to Parkinson's disease. This condition is called “*manganism*”. This phenomenon alters the motor coordination due to an accumulation of manganese in the *globus pallidus*, an area that forms part of the basal ganglia. This effect can be observed mainly in people who are occupationally exposed, for example welders and workers of the steel industry. It should be noted that all the world population has already been exposed to manganese, since this metal is present in most of the food consumed, moreover it is also present in the drinking-water, air, gasoline additives and some other sources. Epidemiological studies demonstrate the relationship of manganese exposure and cognitive deficits in children. Other *in vivo* and *in vitro* studies have shown that exposure to different manganese chemical forms, time periods, and concentrations is linked to an increase in the activation of microglia. In the present study, we assessed the activation of cerebellar micro-glia in mice exposed to either 100 mg/kg of MnCl_2_, 5 mg/kg of lipo-polysaccharide (LPS) or vehicle, *via* a single intraperitoneal injection. Twenty-four hours later, the *cerebellum* was removed and after extensive washing was cryoprotected and sagittally sectioned with a cryostat. Activated microglia was evidenced with the iba-1 antibody. A significant increase in activated microglia was evident in mice treated with LPS and MnCl_2_. These results suggest that MnCl_2_ exposure results in an augmented release of pro-inflammatory cyto-kines (IL-6, TNF-α, IL-1β) and reactive oxygen species leading to microglia activation.

C42

**RETINAL DEGENERATION REFLECTS SPINAL CORD NEURONAL LOSS DURING EXPERIMENTAL AUTOIMMUNE ENCEPHALOMYELITIS**

Gabrielle Mey^1^, Kirsten Evonuk^1^, Laura Wolfe^1^, Rupesh Singh^2^, Julia Batoki^2^, Minzhong Yu^2^, Neal Peachey^2^, Bela Anand-Apte^2^, Tara DeSilva^1^

*^1^ Cleveland Clinic Lerner Research Institute, Neurosciences, Cleveland, USA*

*^2^ Cleveland Clinic Cole Eye Institute, Ophthalmic Research, Cleveland, USA*

Multiple sclerosis (MS) is an inflammatory demyelinating disease of the central nervous system (CNS) which ultimately leads to neuro-degeneration. Although immunomodulatory therapies reduce relapse rate, there is no effective indicator or treatment for the degenerative phase of MS. Visual system impairments are evident in MS, presenting a uniquely accessible system to monitor disease pathology. However, the underlying cellular pathology driving functional and structural visual assessments and how it relates to deficits in the rest of the CNS remains ill-defined. The purpose of this study was to explore the relationship between myelin/axonal injury and neuro-degeneration in the visual system and spinal cord during experimental autoimmune encephalomyelitis (EAE), a chronic model of autoimmune demyelination used for preclinical testing of MS pharmacological therapies. Histopathological assessments were coupled with optical coherence tomography (OCT) to track retinal structure and visually evoked potentials (VEP) to monitor neuronal responses to visual stimuli. Our results show comparable T cell infiltration, demyelination, and axonal loss 15 days post-induction in spinal cord white matter during peak of motor dysfunction and optic nerve when VEPs were delayed. Neither spinal cord neuron nor retinal ganglion cell loss was observed until the chronic phase of disease (35 days post-induction), consistent with OCT imaging showing thinning of the retinal nerve fiber layer. These data suggest a retrograde degeneration of neuronal cell bodies following damage to their respective axons in the white matter. In further support, post-synaptic neuronal loss was not observed anterograde to axonal injury in the spinal cord or visual system (i.e. ventral posterolateral nucleus or lateral geniculate nucleus, respectively). Our results indicate that the visual system and spinal cord have similar time courses for demyelination, axonal injury, and neurodegeneration, and suggest that OCT imaging of the retina may serve as an important indicator of CNS neurodegeneration.

C43

**ROLE OF BLOOD-BRAIN BARRIER TRANSPORT IN STRESS-INDUCED IMMUNE RESPONSE AND DEPRESSION**

Fernanda Neutzling Kaufmann, Katarzyna Dudek, Laurence Dion-Albert, Manon Lebel, Caroline Menard

*Universite Laval, CERVO Brain Research Center, Quebec City, Canada*

Depression affects 322 million people worldwide. Chronic stress promotes immune system dysregulation and recruitment of inflam-matory mediators to the brain promoting depression. The blood-brain-barrier (BBB) restricts peripheral inflammatory cytokines and immune cells from the brain, however, its integrity is compromised in depression. Interestingly, most individuals exposed to stressful events of life remain resilient not developing depression. Accordingly, stress-resilient mice display intact BBB integrity and low inflammatory profile when compared to stress-susceptible animals. Physiology of the brain endothelial cells that line the BBB includes transporters involved in the bidirectional flow of macro-molecules through this barrier, a mechanism named transcytosis. These transporters maintain BBB integrity and control transcytosis keeping it at physiological level, a mechanism that could be disrupted by chronic stress leading to increased passage of inflammatory markers into the brain. Here, we evaluate stress-induced endothelial cell transporters changes in the nucleus accumbens (NAc) of C57Bl6 mice, an important brain area involved in mood regulation and hedonic behavior, and its association with peripheral inflammatory marker. Male and female mice were subjected to 10-day chronic social defeat stress followed by social interaction test 24h later to determine behavioral phenotype. Then, we evaluated transporters gene expression by qPCR and protein levels are currently being quantified by immunostaining and confocal microscopy in the NAc. In addition, we collected mice serum before and after the stress protocol to evaluate the peripheral inflammatory makers by milliplex assay. We observed specific gene expression level patterns in stress-susceptible and resilient male and female mice associated with peripheral inflammatory markers. We are also confirming validity of our mouse findings in post-mortem tissue from depressed patients which will add translational value to this research. The BBB is perfectly located to bridge peripheral immune communication to the brain neuronal circuits. My project sheds new light on the emotions` biology by deciphering how the peripheral immune system interacts with the brain neurovasculature to modify neuronal circuits involved in emotional processing.

C44

**CORONIN 1A FACILITATES CALCIUM MOBILIZATION AND PROMOTES ASTROCYTE REACTIVITY IN HIV-1 NEUROPATHOGENESIS**

Hriday Shanker Pandey, Rishabh Kapoor, Bindu ., Pankaj Seth

*National Brain Research Centre, Cellular and Molecular Neuroscience, Gurgaon, India*

In most neurodegenerative disorders, including neuroAIDS, reactive astrocytes are detrimental to the neuronal population. Calcium and its downstream regulators play a central role in mediating astrocyte reactivity. Coronin 1A, an actin-binding protein, majorly reported in hematopoietic cells, regulates cell activity in a calcium-dependent manner, but its role in astrocyte physiology and reactivity is largely unknown. Using a well-characterized primary culture of human astroglia and neurons, we explored the roles of Coronin 1A in astrocyte physiology and its involvement in facilitating astrocyte reactivity. In this study, we report Coronin 1A expression in human primary astrocytes and autopsy brain sections, and that it plays activity-dependent roles by facilitating Calcium mobilization from the intracellular stores. HIV-1 Tat, a potent neuro-toxicant that turns astrocytes reactive, augments Coronin 1A expression, apart from affecting GFAP and pro-inflammatory mole-cules. Also, the autopsy brain tissue of HIV-1 infected individuals has higher Coronin 1A expression. Downregulation of Coronin 1A attenuated the HIV-1 Tat-induced deleterious effects of reactive astrocytes, measured as upregulated GFAP expression, enhanced release of IL-6, and Glutamate and hence reduced astrocyte-mediated neurodegeneration. Our findings also suggest that out of a pool of dysregulated miRNAs studied by us, hsa-miR-92b-5p regulates Coronin 1A expression under the effect of HIV-1 Tat. These findings highlight the novel roles of Coronin 1A in regulating astrocyte activity in stimulated conditions and astrocyte reactivity observed in HIV-1 neuropathogenesis.

C45

**NEUROPROTECTIVE ROLE OF AUTOPHAGY AND HISTONE ACETYLATION IN Α-SYN PREFORMED FIBRIL ADMINISTERED RAT MODEL OF PARKINSON'S DISEASE**

Violina Kakoty, Sarathlal K C, Rajeev Taliyan

*Birla Institute of Technology and Science, Pilani (BITS Pilani), Neuropsychopharmacology, Pilani, India*

Parkinson's disease (PD) is a devastating, chronic, progressive neurodegenerative disorder characterized both motor and non-motor features. In recent past most researches have been paid attention to autophagy modulation and epigenetic alterations such as histone acetylation in neuroprotection. Hence in this study we have focused to evaluate the neuroprotective efficacy of rapamycin, an autophagy inducer and vorinostat, an histone deacetylase inhibitor in α-syn induced rat model of PD.

PD rat model was developed by unilateral injection preformed fibrillar form (PFF) of α-syn into striatum. A combination of rapamycin and vorinostat was administered intraperitonealy for period of 4 weeks at a dose of 0.5 mg/ kg and 25 mg/ kg respectively. Further, behavioral parameters were assessed by Morris water maze, novel object recognition task and narrow beam walk test. The bio-chemical estimations for IL-6, IL-1, TNF-α CRP, BDNF and dopamine level were performed using standard ELISA kits. The level of different genes involved in autophagy such as BECN1, LC3-II, LAMP-2 were assessed by real time polymerase chain reaction analysis and the histopathologic examination was done by H&E staining.

Intra-striatal PFF α-syn administered rat showed significant characteristic features PD. However, treatment with rapamycin and vorinostat in combination showed significant improvement in the motor and memory deficits in comparison with disease group and their treatment in alone. The level of IL-6, IL-1, TNF-α CRP were significantly reduced whereas level of BECN1, LC3-II, LAMP2, BDNF and dopamine were found increased on combination treatment than that of its treatment in alone. Further, the histopathologic analysis revealed marked reduction in neurodegeneartion in combination treated group with respect to treatment in alone.

Outcomes of the present study indicate that the synergistic action of autophagy induction by rapamycin and histone deacetyalse inhibition by vorinostat elicits marked neuroprotection and attenuates motor and cognitive deficits in PFF α-syn induced PD model of rat.

C46

**TAUOPATHY-INDUCED GLIOVASCULAR DYSFUNCTION IDENTIFIED BY CELL-SPECIFIC VIRAL TRAP-SEQ**

Yutaro Komuro, Mitchell Rudd, Michal Machnicki, Bianca Yugar, S. Thomas Carmichael, Jason Hinman

*UCLA, Neurology, Los Angeles, USA*

Increasing evidence suggests that the inciting pathogenic events in Alzheimer's disease may involve disruption of the multicellular interactions within the neurovascular unit. The interruption of specific molecular pathways within the neurovascular unit occurs antecedent to classic pathology and therefore is an attractive target to modulate neurodegeneration. However, the majority of molecular pathways regulating neural and gliovascular signaling remain unknown. Single-cell and whole tissue gene expression approaches have been used in various animal models of dementia to discern new pathways but are limited by sequencing depth and spatiotemporal accuracy of transcriptional profiling. We developed an approach for identifying relevant and novel pathways within the multicellular environment of the neurovascular unit interactions in Alzheimer's disease. Cell-specific viral constructs coding for antigen-tagged ribosomes (TRAP) were used to study variance in spatial and temporal gene expression patterns within and between cell types in the PS19 (P301S) transgenic model of tauopathy. The engineered lentiviruses and adeno-associated viruses express an HA-tagged ribosomal protein (Rpl10a-HA) driven by a cell-type specific promoter (Synapsin, GFAP, or PDGFRa) for the major cell types of the neurovascular unit. We demonstrated the ability of the viruses to target specific cell types under particular spatiotemporal conditions and confirmed cell-specificity *in vivo* using both known cell-specific markers and novel markers identified by sequencing. We then combined this cell-specific vTRAP system with the PS19 mouse model of tauopathy to generate multiple cell-type specific transcriptomic databases of the neurovascular unit across several pathologically-relevant time points. Gene ontology analysis indicates a prodromal drive by gliovascular cells including pericytes and astrocytes to support injured neurons. This occurs through specific and temporally regulated coordination of multiple molecular programs driving neuronal differentiation, neurite outgrowth, and synaptogenesis. This suggests a paradigm for gliovascular rescue of neurodegeneration phenomena and implicates numerous novel perivascular molecular pathways in the pathogenesis of tauopathy and Alzheimer's disease.

C47

**POTENTIAL ROLE OF LATENT HUMAN HERPES VIRUS 6 (HHV6) INFECTIONS IN NEURON-DEGENERATIVE DISEASES**

Margot Mayer-Proschel, Jessica Hogestyn, Garrick Salois, Li Xie, Chris Proschel

*University of Rochester, Biomedical Genetics, Rochester, USA*

Alzheimer's Disease (AD) is thought to be caused by a combination of multiple genetic and environmental factors, and the role of infectious agents has been debated for decades. A recent multiomic, epidemiological study of large Alzheimer patient cohorts revealed a specific and significant association of human herpesvirus 6A (HHV6A) with AD. However, these studies could not resolve which HHV6A specific genes were involved or how HHV6A might affect cells of the central nervous system (CNS) to exacerbate AD. As HHV6A is mainly present in the brain in its latent form, *we asked the question of whether the major latency associated gene **U94A****,** affects host cells functions that are relevant to Alzheimer disease pathology* Using a human cell system, we found that U94A expression impairs the migration and maturation of human glial progenitor cells (OPCs) and leads to synapse loss in human neurons. We present transcriptomic and proteomic analysis of U94A infected cells that show dysregulation of genes involved in cytoskeletal functions, myelination and synaptic maturation. These phenotypes are particularly relevant for the early stages of Alzheimer disease, as mounting evidence suggests that synapse and neurite loss, associated with diffuse demyelination precede cognitive impairment. This work will provide insight into the potential role of infectious agents in Alz-heimer's pathology and will establish that HHV6A viral latency is not merely a benign state of viral infection, but an important disease modifying factor.

C48

**MULTIPLEX FLOW CYTOMETRY ANALYSIS OF SINGLE EXTRACELLULAR VESICLES SPONTANEOUSLY RELEASED BY BRAIN TISSUE**

Carlos Nogueras-Ortiz^1^, Christopher Dunn^2^, Ana Amorim^3^, Ioannis Sotiropoulus^3^, Dimitrios Kapogiannis^1,4^

*^1^ National Institute on Aging, Laboratory of Clinical Investigation, Baltimore, USA*

*^2^ National Institute on Aging, Flow Cytometry Unit, Baltimore, USA*

*^3^ University of Minho Gualtar, Life and Health Sciences Research Institute, Braga, Portugal*

*^4^ Johns Hopkins School of Medicine, Neurology, Baltimore, USA*

**INTRODUCTION**: Extracellular vesicles (EVs) released by brain cells are known to regulate neuronal and glial function and are emerging as a source of biomarkers for neurodegenerative diseases due to their presence in blood and pathological cargo. These findings underlie the importance of phenotyping single brain derived EVs to properly characterize EV subpopulations and identify disease discriminators. Although several single-particle characterization platforms have been proposed, their nanoscale multiplex capabilities remain elusive.

**METHODOLOGY**: To explore the nanoscale multiplex capabilities of flow cytometry, EVs spontaneously released by mouse brain tissue were subjected to high sensitivity flow cytometry analysis of validated EV membrane tetraspanins, combining violet side scatter-and fluorescence-based particle detection.

**RESULTS**: To explore the relative abundance of tetraspanins, we assessed EV subpopulations positive for either CD9 or CD81, and both, in EVs simultaneously labelled with APC-CD9 and PE-CD81 antibodies. First, we confirmed the capacity of our flow cytometry analysis to distinguish between single-and double-positive events by comparing mixed EVs individually labelled with APC-CD9 and PE-CD81, and simultaneously labelled EVs. APC/PE double-positive events were only detected when simultaneously labelling EVs, and not when individually labelled EVs are mixed prior to analysis, indicating the detection of double-positive single nanoparticles in the absence of coincidental events due to swarming. Further analysis of simultaneo-usly labelled EVs showed that 98% of APC-CD9+ events are PE-CD81+, whereas 42% of PE-CD81+ events are APC-CD9+.

**CONCLUSIONS**: High sensitivity nanoscale flow cytometry analysis allowed quantification of multiple surface markers in single brain derived EVs. This technology holds enormous potential to categorize EV subpopulations and identify surface molecular signatures of cell specific EVs for sorting and subsequent assessment of disease bio-markers.

C49

**TAU IS UPREGULATED IN CROHN'S DISEASE THROUGH A NRF2/NDP52 PATHWAY**

Malvyne Rolli-Derkinderen, Guillaume Chapelet, Alice Prigent, Emilie Durieu, Thibauld Oullier, Michel Neunlist, Pascal Derkinderen

*Inserm U1235, Faculté de Médecine, Nantes, France*

A sizeable body of evidence has recently emerged to suggest that gastrointestinal inflammation might be involved in the development of Parkinson's disease. There is now strong epidemiological and genetical evidence linking Parkinson's disease to inflammatory bowel diseases and we recently demonstrated that the neuronal protein alpha-synuclein, which is critically involved in Parkinson's disease pathophysiology, is upregulated in inflamed segments of Crohn's colon. The microtubule associated protein tau is another neuronal protein critically involved in neurodegenerative disorders but, by contrast to alpha-synuclein, no data are available about its expression and phosphorylation patterns in inflammatory bowel diseases. Here, we examined the expression levels of tau isoforms, their phos-phorylation profile and truncation in colon biopsy specimens from 17 Crohn's disease and 6 ulcerative colitis patients and compared them to samples from 12 controls. Additional pharmacological experiments were performed in primary cultures of rat enteric neurons. Our results show the upregulation of two main human tau isoforms in the enteric nervous system in Crohn's disease but not in ulcerative colitis. This upregulation was not transcriptionally regulated but instead resulted from a decrease in protein clearance via an Nrf2/NDP52 pathway. Our findings, which provide the first detailed characterization of tau in Crohn's disease, suggest that the key proteins involved in neurodegenerative disorders such as alpha-synu-clein and tau, might also play a role in Crohn's disease.

C50

**HOUSE DUST MITE-INDUCED ASTHMA POTENTIATES ALZHEIMER'S DISEASE IN APPNL-G-F MICE**

Bijayani Sahu, Suba Nookala, Angela Floden, Colin Combs

*University of North Dakota, Biomedical Sciences, Grand Forks, USA*

Alzheimer's disease (AD) is a progressive age-related neuro-degenerative disorder affecting approximately 35 million individuals worldwide. AD is a leading cause of death in the USA, with an estimated 5.8 million affected and, by mid-century, the number is expected to grow to 13.8 million. There are several well-established genetic and environmental factors hypothesized to contribute to AD pathology and progression. Besides aging, various comorbid factors also increase the risk of AD, including obesity, diabetes, air pollution, and asthma. Epidemiological studies have reported a 2.17- fold higher risk of dementia in asthmatic patients >45 years old and 1.27-fold in asthmatic patients ≥20 years old. However, the molecular mechanism(s) underlying this asthma-associated AD exacerbation is not known. This study was designed to explore the effects of house dust mite-induced asthma on AD-related brain changes using the *App*^*NL-G-F*^ transgenic mouse model of disease. Male and female C57BL/6 wild type and *App*^*NL-G-F*^mice (8-9 months old) were exposed to either saline vehicle or house dust mite at a dose of 833µg/kg every alternate day for 16 weeks. Mice were sacrificed at the end of the experiment to collect bronchoalveolar lavage fluid (BALF) and brains. BALF was analyzed for immune cell markers and inflammatory mediators by flow cytometry and cytokine arrays, respectively. Aβ ELISAs were performed on frozen hippocampal lysates. As predicted, asthma increased BALF inflammatory cells and several cytokine levels compared to the vehicle controls. Interestingly, asthmatic *App*^*NL-G-F*^ mouse hippocampi had higher levels of soluble Aβ 1-40/42 and insoluble Aβ 1-40. Understanding a mechanistic relationship tying AD and asthma progression may provide novel therapeutic interventions for both of these chronic diseases.

C51

**OVEREXPRESSING LOW-DENSITY LIPOPROTEIN RECEPTOR REDUCES TAU-ASSOCIATED NEURODEGENERATION IN RELATION TO APOE-LINKED MECHANISMS**

Yang Shi^1^, Prabhakar Sairam Andhey^2^, Christina Ising^3^, Kairuo Wang^1^, Lisa L. Snipes^2^, Kevin Boyer^2^, Stephanie Lawson^2^, Kaoru Yamada^4^, Wei Qin^5^, Melissa Manis^1^, Javier Remolina Serrano^1^, Bruno A. Benitez^5^, Robert E. Schmidt^2^, Maxim Artyomov^2^, Jason D. Ulrich^1^, David M. Holtzman^1^

*^1^ Washington University in St. Louis, Neurology, St. Louis, USA*

*^2^ Washington University in St. Louis, Pathology and Immunology, St.Louis, USA*

*^3^ German Center for Neurodegenerative Diseases, Neurodegenerative Diseases and Geriatric Psychiatry, Bonn, Germany*

*^4^ The University of Tokyo, Neuropathology, Tokyo, Japan*

*^5^ Washington University in St. Louis, Psychiatry, St.Louis, USA*

*APOE* is the strongest genetic risk factor for late-onset Alzheimer's disease. ApoE exacerbates tau-associated neurodegeneration by driving microglial activation. However, how apoE regulates microglial activation and whether targeting apoE is therapeutically beneficial in tauopathy is unclear. Here we show that overexpressing an apoE metabolic receptor LDLR (low-density lipoprotein receptor) in P301S tauopathy mice markedly reduces brain apoE, and ameliorates tau pathology and neurodegeneration. LDLR overex-pression in microglia cell-autonomously downregulates microglial *Apoe* expression, and is associated with suppressed microglial activation as is in apoE-deficient microglia. Both apoE-deficiency and LDLR-overexpression strongly drive microglial immunometa-bolism towards enhanced catabolism over anabolism, whereas LDLR-overexpressing microglia also uniquely upregulate specific ion channels and neurotransmitter receptors upon activation. ApoE-deficient and LDLR-overexpressing mice harbor enlarged pools of oligodendrocyte progenitor cells (OPCs), and show greater preservation of myelin integrity under neurodegenerative conditions. They also show less “disease-associated astrocyte” activation in the setting of tauopathy.

C52

**LEUKOTRIENE RECEPTOR ANTAGONIST USE IS ASSOCIATED WITH SLOWER COGNITIVE DECLINE IN ALZHEIMER'S DISEASE**

Lisa Xiong^1,2^, Michael Ouk^2^, Che-Yuan Wu^1,2^, Jennifer Rabin^2^, Krista Lanctôt^1,2^, Nathan Herrmann^2^, Sandra Black^2^, Jodi Edwards^3^, Walter Swardfager^1,2^

*^1^ University of Toronto, Department of Pharmacology & Toxicology, Toronto, Canada*

*^2^ Sunnybrook Research Institute, Hurvitz Brain Sciences Program, Toronto, Canada*

*^3^ University of Ottawa, University of Ottawa Heart Institute, Ottawa, Canada*

Leukotrienes are inflammatory mediators that may contribute to the development and progression of neurodegenerative diseases. Repurposing of leukotriene receptor antagonists (LTRAs), originally developed for asthma, is being explored in the treatment of Alz-heimer's disease (AD); however, there is little evidence supporting its neuroprotective effects in humans. Participants with AD dementia were identified in the National Alzheimer's Coordinating Center database. LTRA users were propensity-matched 1:3 to non-users on age, sex, education, body mass index, smoking history, concomitant use of medications for dementia and other respiratory conditions, ApoE ε4 carrier status, Clinical Dementia Rating (CDR) global score, and vascular brain disease. Cognitive domains including delayed memory (Wechsler Memory Scale-Revised – Logical Memory IIA), psychomotor processing speed (Digit Symbol Coding), and semantic verbal fluency (Animal Naming) were compared between users and non-users in mixed-effects linear or Poisson regression models, as appropriate for the data distribution, yielding unstandardized regression coefficients (B) and rate ratios (RR), respectively. Among n=584 people with AD dementia (mean age 72.2±9.8 years, 59.8% female, mean education 14.0±3.9 years, median CDR global score=1, 55.0% ε4 carriers, mean follow-up 2.35 years), LTRA use was associated with a slower decline in processing speed, as measured by the Digit Symbol Coding test (Β=1.18 [0.090,2.26] symbols/year), and a slower decline in verbal fluency, as measured by the Animal Naming test (Β=0.488 [0.150,0.827] animals/year), but not with a change in logical memory performance (RR=1.06 [0.940,1.20]). These results indicate that use of LTRAs may have benefits for preserving function for select cognitive domains in individuals with AD. The leukotriene pathway may represent a target for the treatment of AD and the role of leukotrienes and their receptors in cognitive decline warrants further investigation.

C53

**LIPID BIOCHEMISTRY PROBED WITH NILE RED SPECTRAL MICROSCOPY REVEALS NOVEL MYELIN DEFECTS IN PMP22+/-**

Wulin Teo^1^, Bo Hu^2^, Jun Li^2^, Peter K. Stys^1^

*^1^ University of Calgary, Dept. of Clinical Neurosciences, Calgary, Canada*

*^2^ Wayne State University School of Medicine, Dept. of Neurology, Detroit, USA*

We have previously developed a spectroscopy method based on Nile Red, a polarity-sensitive lipophilic dye for detecting myelin changes at the earliest stages of myelin injury in the cuprizone mouse model and normal appearing white matter in multiple sclerosis(MS) patients. We proposed that increases in the measured dielectric constant of myelin, in still-myelinated axons, may underpin physiologically important conduction block in MS and other deor dysmye-linating disorders. Here we explore this question in heterozygous knockout of the Pmp22 myelin gene, an established mouse model of hereditary neuropathy with liability to pressure palsies(HNPP). Action potential conduction abnormalities, including conduction block, are a hallmark of HNPP. Spinal roots from Pmp22+/- mice and age-matched wild-type mice were labeled with NR and imaged on a spectral confocal microscope. In still-myelinated axons, there was a significant (p<0.001, N=9 mice) increase in myelin polarity in Pmp22+/- mice. NR spectroscopy revealed a significant polarity shift to higher values as early as postnatal day 8 in Pmp22+/- nerves (p<0.001, N=4 mice). Tomacula, thickenings of myelin segments characteristic of HNPP, showed an unexpected polarity gradient in-creasing radially from outer to inner mesaxon. Lastly, we examined the role of non-polar cholesterol in myelin polarity changes. We stained the nerves with filipin, a cholesterol-specific dye, and found that filipin signal was reduced in myelin regions with increased polarity, this lipid plays a key role in establishing normal physical properties of myelin. CONCLUSION: NR spectroscopy reports early physicochemical changes in myelin in the PNS of Pmp22+/- mice, with cholesterol playing a key role. Along with previously observed myelin junction disruption and abnormally increased myelin perme-ability in HNPP mouse, the sensitive measures of polarity using this method indicate that increases of myelin dielectric constant in still-myelinated fibers reveal a new mechanism that may underpin clinically important abnormalities of conduction. Supported by CIHR and NINDS R01NS115748

C54

**PALMITOYLATION OF ACETYLATED TUBULIN AND ASSOCIATION WITH CERAMIDE-RICH PLATFORMS IS CRITICAL FOR CILIOGENESIS**

Priyanka Tripathi^1,2^, Zhihui Zhu^1^, Haiyan Qin^1^, Ahmed Elsherbini^1^, Simone M. Crivelli^1,2^, Emily Roush^1^, Guanghu Wang^1^, Stefka D. Spassieva^1,2^, Erhard Bieberich^1,2^

*^1^ University of Kentucky, Department of Physiology, Lexington, United States*

*^2^ Veterans Affairs Medical Center, Lexington, United States*

Microtubules are polymers composed of αβ-tubulin subunits that provide structure to cells and play a crucial role in the development and function of neuronal processes and cilia, microtubule-driven extensions of the plasma membrane that have sensory (primary cilia) or motor (motile cilia) functions. To stabilize microtubules in neuronal processes and cilia, α tubulin is modified by the posttranslational addition of an acetyl group, or acetylation. We discovered that acetylated tubulin in microtubules interacts with the membrane sphingolipid, ceramide. However, the molecular mechanism and function of this interaction are not understood. Here, we show that in human iPS cell-derived neurons, ceramide stabilizes microtubules, which indicates a similar function in cilia. Using proximity ligation assays, we detected complex formation of ceramide with acetylated tubulin in *C. reinhardtii* flagella and cilia of human embryonic kidney (HEK293T) cells, primary cultured mouse astrocytes, and ependymal cells. Using incorporation of palmitic azide and click chemistry-mediated addition of fluorophores, we show that a portion of acetylated tubulin is S-palmitoylated. S-palmitoylated acetylated tubulin is colocalized with ceramide-rich platforms (CRPs) in the ciliary membrane, and it is coimmunoprecipitated with Arl13b, a GTPase that mediates transport of proteins into cilia. Inhibition of S-palmitoylation with 2-bromo palmitic acid or inhibition of ceramide biosynthesis with fumonisin B1 reduces formation of the Arl13b-acetylated tubulin complex and its transport into cilia, concurrent with impairment of ciliogenesis. Together, these data show, for the first time, that CRPs mediate membrane anchoring and interaction of S-palmitoylated proteins that are critical for cilium formation, stabilization, and function. This work was supported by the NIH grants R01NS095215, R01AG034389, and R01AG064234 the NSF grant NSF1615874, and the VA grant I01BX003643.

C55

**AN INTRODUCTION TO THE NIH SHARED INSTRUMENTATION PROGRAMS**

Guanghu Wang, Alena Horska, Ashley Barnes, Malgorzata Klosek

*National Institutes of Health, Office of Research Infrastructure Program/DPCPSI/OD, BETHESDA, USA*

The NIH shared instrumentation (S10) programs, managed by the Office of Research Infrastructure Programs (ORIP), support acquisition of state-of-the-art commercially available scientific instruments for NIH-funded research. The S10 programs have served the biomedical research community for more than 25 years. In the last five years, close to 600 S10 grants were awarded to 177 institutions across the United States. Imaging and mass spectrometry were among the top-funded technologies requested. These awards have contributed to almost all areas of NIH-supported research, including neuroscience.

There are three S10 funding mechanisms in FY2021, the High-End Instrumentation (PAR-21-126) program, the Shared Instrumentation Grant (PAR-21-127) program, and a newly-introduced Basic Instrumentation Grant (PAR-21-125) program. We will describe their eligibility and program requirements, the most important of which are shared-usage and justification of scientific need. We will also share insights on application preparation and how to locate S10 instruments adjacent to you.

C56

**REGULATION OF BRAIN AGING BY NEUTRAL SPHINGOMYELINASE**

Zhihui Zhu^1^, Haiyan Qin^1^, Simone M. Crivelli^1^, Ahmed Elsherbini^1^, Emily Roush^1^, Priyanka Tripathi^1,2^, Mariana Nikolova-Karakashian^1^, Timothy McClintock^1^, Liping Zhang^1,2^, Zainuddin Quadri^1,2^, Stefanka D. Spassieva^1^, Erhard Bieberich^1,2^

*^1^ University of Kentucky, Department of Physiology, Lexington, USA*

*^2^ Veterans Affairs Medical Center, Medical Center, Lexington, USA*

We showed that deficiency of neutral sphingomyelinase 2 (nSMase2) improves memory in Alzheimer's disease model (5xFAD) and wildtype mice, indicating that nSMase2 regulates neural function in the aging brain. To understand the underlying molecular mechanism, targeted mass spectrometry (sphingolipidomics) and RNAseq analysis was performed on the cortex from aging (10 months-old) nSMase2-deficient (fro/fro) and heterozygous (+/fro) mouse brain. fro/fro brains showed reduced levels of ceramide, particularly in astrocytes. Gene cluster (KEGG) analysis predicted that genes related to oxidative phosphorylation and astrocyte activation were down-regulated. Genes in axon formation and synaptic signal transduction such as the glutamate receptor subunit 2B (NR2b/Grin2B) were upre-gulated, indicating a role of nSMase2 in the regulation of oxidative stress and cognition. In primary astrocytes, oxidative stress decreased the level of glutathione (GSH) and increased immunolabeling for ceramide in +/fro astrocytes, but not in fro/fro astrocytes. β- glucosidase activity was lower in fro/fro astrocytes, indicating reduced senescence due to nSMase2 deficiency. In fro/fro cortex, levels of the senescence markers C3b and p27, and the pro-inflammatory cytokines interleukin 1β, (1L-1β), interleukin 6 (IL-6), and tumor necrosis factor α (TNF-α) were significant reduced, concurrent with 2-fold decreased phosphorylation of their downstream target, protein kinase Stat3. Consistent with RNAseq analysis, protein levels of NR2b/Grin2b were elevated by 2-fold. In summary, our data indicate that nSMase2 deficiency and reduction of ceramide levels ameliorates oxidative stress, neuroinflammation, and cognition in the aging brain. Funding: This work was supported by the NIH grants R01AG034389 and R01AG064234, and the VA grant I01BX003643.

## Poster Session D

D01

**DEGENERATIVE AND ADAPTIVE CHANGES IN THE RESPIRATORY SYSTEM OF NEUROPATHIC MICE**

Lucia Notterpek, Malavika Nair, Jie Zhao, Hannah Bazick, Allison Franklin, Alexa Mealy

*Univ. of Florida Neuroscience Dept., McKnight Brain Institute, Gainesville, USA*

Though peripheral neuropathies normally refer to weakness of the extremities, respiratory complications have also been documented in Charcot-Marie-Tooth disease type 1A (CMT1A) and Dejerine-Sottas disease (DSS) patients. Heterozygous Trembler J (TrJ) mice are an established model of severe hereditary demyelinating neuropathies as they carry the same Leu16Pro mutation in PMP22 which is found in DSS patients. Our previous studies of 2-9-month-old TrJ mice indicate severe phrenic nerve degeneration with significant demye-lination, and a concurrent hypertrophy of diaphragm muscle fibers. To investigate the underlying mechanism for this unexpected phenotype, we performed immunohistochemistry and biochemical analyses of diaphragm tissues from 2-9-month-old male and female Wt and TrJ mice. Immunolabeling with anti-actinin antibodies showed intact Z-lines in affected diaphragms from 2-month-old mice, suggesting the preservation of functional contractile units at this age. Western blot analyses of whole diaphragm tissue lysates demon-strated significant (p<0.01) upregulation of the ubiquitin-proteasome pathway and an increase in the autophagy marker LC3II. Immuno-histochemistry on frozen diaphragm tissue sections confirmed the biochemical results, as samples from neuropathic mice displayed in-creased reactivity with antibodies against ubiquitin and the lysosomal membrane protein 1 (LAMP1). As a follow up to the phrenic nerve degeneration, we also examined spinal cord cross sections from vertebrae C3-C5 of 4-month-old male and female Wt and TrJ mice. Double immunolabeling with cell type specific markers indicated astrogliosis in samples from TrJ mice. Atypical neurofilament whirls were also seen in occasional ventral motor neurons, demonstrating ongoing CNS pathology in phrenic neuropathy. Together, our results indicate pathological degenerative events in both the PNS and CNS of neuropathic mice, co-occurring with adaptive changes in the diaphragm. Further investigation of the respiratory complications in neuropathic rodent models will contribute towards identifying the appropriate tissue targets to improve human patient quality of life.

D02

**TFEB REGULATES MYELIN HOMEOSTASIS IN SCHWANN CELLS**

Akash Patel, Imran Khan, Corey Heffernan, Patrice Maurel, Radek Dobrowolski, Haesun Kim

*Rutgers University, Biology, Piscataway, USA*

Transcription factor EB (TFEB) is a master regulator for lysosomal and autophagosomal genes. TFEB is a member of the MiTF-TFE family, which also includes TFE3, a similarly regulated transcription factor that has overlapping targets. Recently, TFEB has been shown to be a negative regulator of oligodendrocyte myelination and has been suggested to play a role in myelin degradation in Schwann cells after nerve injury. The role of TFEB in regulating myelination has not been thoroughly examined in the PNS. To investigate the role of TFEB in Schwann cell myelination, we used both an *in vitro* gain of function of TFEB and an *in vivo* dual loss of function of TFEB and TFE3 approaches. We generated Schwann cells in which a constitutively active, Flag-tagged TFEB mutant (S211A) is expressed in a doxycycline-dependent manner. In Schwann cell-dorsal root ganglion co-cultures, Flag-TFEB^S211A^ expression inhibited myelin initiation and early myelin growth. In mature myelin cultures, inducing Flag-TFEB^S211A^ expression reduced the number of existing myelin segments indicating a disruption in myelin maintenance. Schwann cell proliferation was not significantly affected by Flag-TFEB^S211A^ expression. *In vivo*, preliminary analysis of Schwann cell specific TFEB KO (Dhh-Cre;TFEB^fl/fl^) on a TFE3 global KO background (dKO) mouse sciatic nerves show a mild increase in in-foldings and abnormal thickness by P90. Our results suggest that aberrant activation or inactivation of TFEB in Schwann cell disrupt myelin homeostasis.

D03

**THE PROTECTIVE EFFECTS OF CHPG IN THE EAE MOUSE MODEL THROUGH METABOTROPIC GLUTAMATE RECEPTORS**

Talia Planas-Fontánez^1^, Yangyang Huang^2^, Swetha Sathi^2^, April De Stefano^2^, Cheryl Dreyfus^2^

*^1^ Rutgers University, Joint Graduate Program in Toxicology, Piscataway, USA*

*^2^ Robert Wood Johnson Medical School,Department of Neuroscience and Cell Biology, Piscataway, USA*

Most treatments for demyelinating diseases, such as Multiple Sclerosis, focus on targeting focal inflammatory lesions. However, treatments to enhance myelin repair after lesion are still needed. Previous studies in our laboratory demonstrated that the metabotropic glutamate receptor (mGluR) agonists, 1-amino-1,3-dicarbo-xycyclopentane and (R,S)-2-chloro-5-hydroxyphenylglycine (CHPG) enhance myelin proteins in a lesion site through release of the growth factor BDNF from astrocytes, mediated through mGluR5 (Fulmer et al, 2014; Saitta et al, 2021). Here, we assess the effects of CHPG in the MOG-induced EAE mouse model. Studies demonstrate that CHPG injections (20mg/kg; i.p.; every other day) from day 12 post induction, significantly reduce clinical scores from day 20 to 30. CHPG-treated EAE mice present tail paralysis as opposed to saline-injected controls that exhibit hindlimb weakness. Western blot analysis of the lumbar spinal cord on day 21 demonstrates that CHPG elevates myelin proteins and GFAP while BDNF is unaffected. Immunohistochemistry reveals an increase in GFAP+ astrocytic profiles and a decrease in numbers of Ly-6g+ neutrophils in the ventral white matter. CHPG treatment of EAE mice at late stages of disease significantly attenuate disease progression between day 23 and 30. The reduction, though, is of less magnitude. This effect was accompanied by reversal of the loss in BDNF and PLP in the lumbar spinal cord to unlesioned control levels. Colocalization of mGluR5 with GFAP+ astrocytes, Ly-6g+ neutrophils and rarely Iba1+ activated microglia is noted within the lesion, indicating that these cells may be targets of CHPG. To define the role of astrocyte-derived mGluR5, effects of CHPG were examined in inducible-conditional hGFAP-CreERT2-mGluR5 fl/fl ROSA26 mice. The deletion of mGluR5 inhibits effects of CHPG. The data suggest that targeting astrocyte-derived mGluRs might provide a new therapeutic opportunity, although other immune cells may be involved.

Supp. NMSS RG4257B4/1, NIH RO1 NS03664, T32ES007148, BMS Toxicology Fellowship, SGS Biomedical Acceleration and Completion Fellowship.

D04

**CHRONIC DEMYELINATION AND REMYELINATION OF SPARED AXONS AFTER SPINAL CORD INJURY**

Nicole Pukos^1,2^, Dana McTigue^1,2^

*^1^ The Ohio State University, Neuroscience, Columbus, USA*

*^2^ Belford Center for Spinal Cord Injury, Neuroscience, Columbus, USA*

Spinal cord injury (SCI) renders motor and sensory deficits below the level of injury, in part, due to severed axons and extensive axon demyelination. Demyelination blocks or slows down conduction through spared axons, leading to functional impairments. This is in part due to node Ranvier disruption as highly organized proteins around the node, like contactin associated protein (Caspr) and voltage-gated potassium channels (Kv1.2), spread along axons. After SCI, some demyelinated axons are spontaneously remyelinated by newly formed oligodendrocytes acutely, which improves neuronal communication. Because it is thought to be incomplete, remyelina-tion is often pursued as a therapeutic strategy to restore function after SCI; however, the target and duration for remyelination remains unknown. Here, **we tested the hypothesis that new myelin forms on demyelinated axons chronically after SCI.** For this, reporter mice were used to quantify the amount of new myelin formed during specific periods of recovery. In naïve mice, only ∼5% of axons were wrapped in new myelin. However, after SCI, remyelination significantly increased and peaked during the 3rd month post-injury (mpi) when over 15% of axons were remyelinated. Interestingly, spared axons continued to be remyelinated for at least 26wpi, although at a lower rate. To determine if chronic remyelination repaired all demye-linated axons, the number of intact nodes of Ranvier were quantified. Intact nodes were 6.5x lower after SCI compared to naïve, and there was marked spreading of Caspr and Kv1.2 along axons, which persisted for at least 26wpi. The presence of demyelinated axons at chronic timepoints was confirmed with electron microscopy. Overall this work shows that demyelination persists chronically post-SCI, but that spontaneous remyelination of a subset of axons continues chronically.This reveals that the chronic spinal cord injury site remains a surprisingly dynamic environment even 6mpi, and raises hope that endogenous progenitor cells remain responsive to environmental cues and may be a feasible target to promote more complete remyelination chronically after SCI.

D06

**TGFBETA1 DEPENDENT GPNMB EXPRESSION IN NEURAL STEM CELLS INHIBITS OLIGODENDROGENESIS**

Daniel Radecki, Jayshree Samanta

*University of Wisconsin-Madison, Comparative Biosciences, Madison, USA*

Demyelination of the central nervous system (CNS) is a patho-logical feature of many neurodegenerative diseases, and leads to altered signaling, axonal stress, and neurodegeneration. Multiple Sclerosis (MS) presents with widespread demyelination, which is refractory to current treatments and interventions. Recent research identified a population of adult neural stem cells (NSCs) that express the transcription factor Gli1 in the adult subventricular zone (SVZ) and are capable of generating new oligodendrocytes for remye-linating the CNS. Loss of Gli1 in these NSCs further increases their recruitment to the site of demyelination and differentiation into mye-linating oligodendrocytes, with functional improvement in an Experimental Autoimmune Encephalomyelitis (EAE) model of MS. To understand the molecular underpinnings of enhanced remyelina-tion, we performed an RNAseq screen in NSCs with loss of Gli1 from demyelinated brain and identified Glycoprotein non-metastatic melanoma b (Gpnmb) as the most differentially expressed gene with ∼6 fold higher expression in wildtype NSCs. In the healthy brain, Gpnmb is expressed in subsets of NSCs, microglia, astrocytes, oligo-dendrocyte precursors (OPCs), and neurons but not mature oligo-dendrocytes. Global knockout of Gpnmb results in an increase in mature oligodendrocyte generation during remyelination, while over-expression of Gpnmb *in vitro* suppresses oligodendrocyte gene expression; thus indicating that Gpnmb inhibits remyelination. We also found that TGFß1 secreted from the site of demyelination increases Gpnmb expression in NSCs. In addition, increased Gpnmb levels stimulate TGFß-R2 expression, the ligand binding subunit of the TGFß1 receptor dimer, indicating Gpnmb may function in a feed-forward loop to sensitize NSCs to TGFß1. Taken together, our data strongly implicate Gpnmb as a negative regulator of remyelination that is regulated by and feeds forward to amplify TGFß1 signaling.

D07

**REPURPOSING CARDIAC GLYCOSIDE DIGOXIN FOR MYELIN REPAIR/REGENERATION FOR MULTIPLE SCLEROSIS IN RODENT PRE-CLINICAL TRIALS**

Haley Titus^1^, Huan Xu^2^, Andrew Robinson^1^, Priyam Patel^1^, Damiano Fantini^1^, Molly Karl^3^, Eric Garrison^3^, Indigo Rose^4^, Shane Liddelow^4^, Robert Miller^3^, Brian Popko^2^, Stephen Miller^1^

*^1^ Northwestern University Feinberg School of Medicine, Microbiology & Immunology, Chicago, USA*

*^2^ Northwestern University Feinberg School of Medicine, Neurology, Chicago, USA*

*^3^ George Washington University, Anatomy & Cell Biology, Washington DC, USA*

*^4^ New York University Grossman School of Medicine, Neuroscience & Physiology, New York, USA*

The CNS autoimmune disease Multiple Sclerosis (MS) is characterized by demyelination and neurodegeneration. Currently, there are no available therapies marketed for myelin repair/regeneration in MS. As MS is a chronic disease that often presents in young adulthood, there is a need for both halting progression and repairing existing damage. In an effort to repurpose FDA approved medication to expedite therapies to patients, we tested the cardiac glycoside (Na^+^/K^+^ ATPase inhibitor) digoxin and revealed it promoted differentiation of the oligodendrocyte cell lineage *in vitro and in vivo* in *C57BL/6J* mice. Digoxin stimulated a more robust recovery of mye-linated axons in the LPC spinal cord model and a quicker restoration of myelin integrity in the corpus callosum in the non-T cell-mediated Cuprizone model of demyelination/remyelination as well as improved clinical score throughout the autoreactive Th1/Th17 driven *C57BL/6J* chronic experimental autoimmune encephalomyelitis (EAE) time course. Available FDA-approved disease modifying therapies for MS are global immunosuppressants with limited efficiency. Our lab is able to induce immune tolerance to selectively target the immune system in relapsing-remitting (RR-EAE) and chronic progressive (C-EAE) experimental autoimmune encephalo-myelitis murine models of MS using an i.v. infusion of nanoparticles coupled with or encapsulating myelin peptides (Ag-PLG) that pro-phylactically prevent disease and therapeutically stop disease progression without compromising the entire adaptive immune system. Combination therapy with selective immune regulation (PLG-MOG) and digoxin at peak of C-EAE disease completely ameliorated clinical disease severity and restored the oligodendrocyte cell lineage. These findings provide pre-clinical evidence for future clinical trials using combination therapy in MS patients.

D08

**ROLE OF TIM-2 IN CNS MYELINATION**

Quinn Wade, Elizabeth Neely, James Connor

*Penn State College of Medicine, Neurosurgery, Hershey, USA*

Myelin, which is formed by oligodendrocytes (OLs) in the central nervous system (CNS), is crucial for efficient signal transduction and neuronal function. Iron is a critical micronutrient for OLs due to its role as a cofactor for myelin synthesis, and it is well-known that OLs are the highest iron staining cells in the brain. Inadequate iron delivery to OLs frequently results in severe and long-lasting neuro-logical deficits that are attributed to hypomyelination. Our laboratory and others have identified H-ferritin (Fth), traditionally considered solely an iron storage protein, as the primary iron delivery protein to OLs. We have also found that H-ferritin utilizes a novel receptor on rodent OLs, T-cell immunoglobulin and mucin domain-containing protein-2 (Tim-2). To ascertain the importance of H-ferritin-mediated iron delivery through Tim-2 on OLs for myelination, we have generated *Timd2*^*fl/fl*^ *Plp1-Cre/ERT* conditional knockout mice to eliminate Tim-2 expression specifically from OLs following tamoxifen injections. We hypothesize that removal of Tim-2 from OLs will lead to decreased H-ferritin uptake leading to insufficient myelination. In this study, we observed that *Timd2*^*fl/fl*^ *Plp1-Cre/ERT* mice injected with 75 mg/kg tamoxifen on post-natal days 7-9 to induce the knockout displayed a trend towards poorer motor function on the rotarod as measured by increased number of falls and decreased latency to fall. In addition, there is a statistically significant reduction in myelin basic protein (MBP) in animals that received tamoxifen compared to controls. Essentially, the data from this study indicates that myelin is negatively affected following Tim- 2 knockout from OLs.

D09

**"A1" ASTROCYTES ARE NEUROTOXIC AFTER STROKE**

Todd Peterson^1^, Evan Brahms^2^, Alex Munch^3^, Maya Weigel^3^, Kenya Inoue^1^, Ben Barres^3^, Marion Buckwalter^2^, Shane Liddelow^4^

*^1^ University of North Carolina - Wilm, Psychology, Wilmington, USA*

*^2^ Stanford University, Neurology and Neurological Sciencese, Stanford, USA*

*^3^ Stanford University, Neurobiology, Stanford, USA*

*^4^ New York University, Neuroscience and Physiology, New York, USA*

Multiple different neurological disorders, like neurodegenerative disorders or neural insults such as stroke create a neuroinflammatory response that leads to glial cell activation. These cells change their conformation and take on multiple different phenotypes, causing them to lose much of their normal functions and take on an inflammatory role. Activation of microglia following injury leads to the conversion of astrocytes into an “A1” phenotype that is more neurotoxic in (Parkinson's Disease and optic nerve crush models). Three cytokines, tumor necrosis factor (TNF), interleukin 1a (Il-1a), and complement component 1q (C1q) are released from microglia and have been determined to induce the A1 astrocyte phenotype. We prevented “A1” neurotoxic astrocyte formation after stroke with triple knockout (TNF-, Il-1a-, and C1q-) mice, and compared their neuroinflammatory response and stroke size to wildtype mice. We hypothesized that inhibition of “A1” astrocytes in triple knockout mice would lead to less astrogliosis and reduced infarct size. Following distal middle cerebral artery occlusion, the infarct size of triple knockout mice was significantly less than wildtype mice at both 1 (*p*<0.05) and 7 days (*p*<0.0001) post-ischemia. We also found a reduction in astrogliosis in the triple knockout animals 7 days post-stroke (*p*<0.05). No significant reduction of infarct size (*p*= 0.4343) was found 28 days post-dMCAO, but there was a reduction in astrogliosis (*p*<0.05) in the triple knockout animals. Removing “A1” neurotoxic astrocytes from the neuroinflammatory response to stroke led to a reduction in the glial response to stroke and reduced infarct volume. Understanding the neurotoxic phenotype of this astrocyte activation and its feedback role on other resident and infiltrating cells could lead to treatments to reduce an exacerbated neuroinflammatory response.

D10

**MPGES1 GLIAL EXPRESSION IS PART OF THE SEX RISK FACTOR OF INFLAMMATORY BOWEL DISEASE**

Malvyne Rolli-Derkinderen, Emilie Durieu, Anne Bessard, Thibauld Oulier, Emilie Duchalais, Michel Neunlist

*Inserm U1235, Faculté de Médecine, Nantes, France*

The aim of this study was to investigate the role of PGE2 glial production by mPGES1 on intestinal permeability, motility and inflammation in vivo in mouse model of colitis, and ex vivo on human samples, and to associate these data with the expression level of mPGES1 in EGC from IBD patients. We generated mice expressing the tamoxifen-inducible Cre recombinase under control of the S100bpromoter and carrying the mPGES1^fl-fl^ gene (S100b^Cre-ERT2^–mPGES1^fl-fl^). The effects of mPGES1 deletion were analyzed in 10- 20 weeks old male or female (mPGES1 DEGC mice) and compared to 10-20 weeks old male or female S100b^Cre-ERT2^ or mPGES1^fl-fl^(control mice), treated with tamoxifen too. Two weeks after the onset of the induction of deletion, we induced, or not, the development of colitis by adding dextran sulfate sodium (DSS 4%) in the drinking water during 4 days. Paracellular permeability, gut motility, total transit time and Disease Activity Index (DAI) were evaluated. PGE2 and estradiol impact on glia and/or intestinal epithelial cells were assessed in vitro. IHC analyses were also assessed on human control and IBD samples.The increase in mPGES1 glial expression observed after exposure to DSS in the myenteric ganglia of male and female wild control mice is absent in mPGES1^KO^EGC mice. mPGES1 deletion had no impact on male intestinal function, in control or DSS conditions. The female mice deleted for glial mPGES1 presented comparable permeability and DAI, and transit time as male. Remarkably, wild type female had higher control permeability and higher mPGES1-glial expression. This work represents the first in vivo evidence of direct involvement of glial-derived PGE2 in the pathophysiology of the intestine. It can regulate intestinal permeability and motility and sensitize the development of colitis in female mice.

D11

**ENDOPLASMIC RETICULUM STRESS INITIATES JANUS KINASE (JAK) 1-DEPENDENT GENE EXPRESSION IN ASTROCYTES**

Savannah Sims^1^, Gordon Meares^1,2^

*^1^ West Virginia University, Immunology, Microbiology, and Cell Biology, Morgantown, USA*

*^2^ West Virginia University, Neuroscience, Morgantown, WV, USA*

Neurodegenerative diseases are associated with misfolded proteins in the endoplasmic reticulum (ER) and dysregulated immune signaling. ER stress occurs when the protein folding capacity of the ER is overwhelmed, resulting in initiation of the unfolded protein response (UPR) to restore homeostasis. Unresolved UPR activation leads to cell death and inflammation. We have described an ER-stress induced Janus Kinase (JAK) 1 - Signal Transducer and Activator of Transcription (STAT) 3-dependent mechanism that promotes expression of inflammatory mediators including Interleukin-6 (IL-6). JAK1 is initiated by cytokine stimulation to promote inflammatory gene expression, and additionally, using RNA-seq, we have shown JAK1 controls many ER stress-induced genes. We found that less than 10% of the ER stress-induced JAK1-dependent genes are also induced by cytokine stimulation, demonstrating ER stress and cyto-kine stimulation induce distinct JAK1-dependent transcriptional pro-files in astrocytes, a nonneuronal brain cell that responds to insult by producing soluble immune mediators. We found that JAK1 controls expression of genes that are not regulated by STATs, but by activating transcription factor (ATF) 4. We have shown that JAK1 and ATF4 physically interact and, via chromatin immunopre-cipitation (ChIP), in response to ER stress, ATF4 is effectively recruited to these promoters of these genes in a JAK1-dependent manner suggesting that JAK1 is involved in directing ATF4-dependent gene expression in a gene specific manner. These findings suggest JAK1 is a major driver of transcription in response to cellular stress, and JAK1 exhibits novel signaling mechanisms to regulate gene expression through ATF4.

D12

**COMPLEMENT ACTIVATION DURING STAPHYLOCOCCUS EPIDERMIDIS CNS CATHETER INFECTION**

Gwenn Skar^1^, Matthew Beaver^1^, Lara Bergdolt^2^, Anna Dunaevsky^2^, Tammy Kielian^3^

*^1^ University of Nebraska Medical Center, Pediatric Infectious Disease, Omaha, USA*

*^2^ University of Nebraska Medical Center, Neurological Sciences, Omaha, USA*

*^3^ University of Nebraska Medical Center, Pathology and Microbiology, Omaha, USA*

Cerebrospinal fluid (CSF) shunt infection is a common and devastating complication of the treatment of hydrocephalus. The majority of these infections are cause by *S. epidermidis* and can lead to long term neurologic consequences such as seizures and decreased IQ. The mechanisms by which these consequences occur are unknown. Our preliminary studies in a rat model of CSF revealed elevated levels of many complement components at late time points when bacterial burdens were low suggesting a role for complement beyond bacterial opsonization. Complement has been associated with the pathogenesis of neurodegenerative diseases such as MS and Alz-heimer's as well as CNS infections such as HIV and bacterial meningitis. More recently there is evidence to support the role of complement components in directing developmentally appropriate synaptic pruning. This literature and our preliminary studies suggest that complement may be responsible for the neurologic damage that occurs in response to shunt infection. We hypothesize that complement creates these deleterious neurologic effects through pathologic pruning of neural synapses. To evaluate this hypothesis we used a standardized mouse model of *S. epidermidis* CNS catheter infections, allowing for the evaluation of host response to infection. Early data demonstrated elevated levels of the complement components C3 and C5 at day 5 post-infection when bacterial burdens are low suggesting a role beyond control of the infection. Additional studies comparing WT and C3 KO mice demonstrate a trend towards increased synaptic number in C3 KO mice compared to WT which supports our hypothesis that complement is responsible for pathologic synaptic pruning in *S. epidermidis* shunt infection. Interestingly, Factor B is elevated at all time points in animals with *S. epidermidis* infected CNS catheters indicating that the alternative pathway may be major mode of complement activation during these infections and a potential target for therapeutic interventions.

D13

**CXCR3 AND CAVEOLIN-1 PROMOTE T CELL CNS INFILTRATION IN EAE**

Troy Trevino, Deanna Keen, Andrea Ochoa-Raya, Sarah Lutz

*University of Illinois at Chicago, Anatomy & Cell Biology, Chicago, USA*

Entry of pathogenic T cells into the central nervous system (CNS) causes disease in the multiple sclerosis (MS) animal model experimental autoimmune encephalomyelitis (EAE). Extravasation across the blood-brain barrier (BBB) occurs via transcellular trafficking within endocytic vesicles or by tight junction dissolution. Our previous data suggested that encephalitogenic Th1 cells utilize Caveolin-1 (Cav-1) to cross the BBB in MOG_35-55_ EAE. However, Cav-1 is expressed on T cells, endothelial cells, and neurons. Additionally, the molecular signals targeting migratory T cells to trans-cellular, instead of paracellular, BBB migration remain unclear. Th1 cells highly express the chemokine receptor CXCR3, and its ligand CXCL10 is upregulated in the CNS in MS and EAE. Here, we have tested the hypothesis that CXCR3 promotes migration of T cells across the BBB dependent on endothelial Cav-1. CXCR3+ T cells were reduced in the spinal cords of Cav-1 null mice with EAE. CXCL10 enhanced transcellular chemotaxis across primary BBB endothelial cells. We utilized novel endothelial cell conditional Cav-1 knockout mice to further probe this hypothesis. Our findings suggest that chemokine-mediated caveolar transmigration may be a target for modulating BBB permeability.

D14

**VESICULAR TRANSLOCATION AND RETENTION OF MHC CLASS II MOLECULES AT THE SURFACE OF INTERFERON Γ – ACTIVATED ASTROCYTES**

Robert Zorec^1,2^, Mićo Božić^1^, Matjaž Stenovec^1,2^

*^1^ University of Ljubljana, Medical Faculty, LN-MCP, Institute of Pathophysiology, Ljubljana, Slovenia*

*^2^ Celica Biomedical, Leb Cell Engineering, Ljubljana, Slovenia*

Astrocytes are heterogeneous glial cells that maintain homeostasis and provide for defense of the central nervous system (CNS). In response to CNS damage they undergo morphological and functional alterations collectively termed reactive astrogliosis. However, the capacity of astrocytes to participate in immune interactions is still incompletely understood. Interferon γ (IFNγ), an inflammatory cyto-kine, induces expression and plasmalemmal translocation of major histocompatibility type II molecules (MHCII) that present antigens on cell surface. To identify principal vesicles involved in MHCII delivery and retention at the plasmalemma of cultured rat astrocytes, we investigated the subcellular localization of MHCII by confocal and structured illumination microscopy (SIM), and examined exo-/ endocytotic interactions at a single vesicle level by high-resolution cell-attached membrane capacitance measurements. Astrocyte treatment with IFNγ increased the expression of MHCII, which upon immunolabelling strongly co-localized with LAMP1-EGFP, a marker of lysosomes, but only scarcely with immunolabelled Rab4A, EEA1 and TPC1, markers of early and recycling endosomes. As revealed by SIM, numerous MHCII-positive vesicles were positioned at the plasmalemma and displayed larger diameters in IFNγ-treated astrocytes as compared to non-treated controls. Furthermore, MHCII localized to the plasmalemma exclusively in IFNγ-treated astrocytes. Membrane capacitance measurements revealed reversible and full exo-/endocytotic vesicle interactions with the plasmalemma. Following IFNγ treatment, reversible exocytosis of vesicles with larger diameter was observed. Additionally, IFNγ treatment reduced the frequency of full endocytotic events. In IFNγ-treated astrocytes, exocytosis of predominately larger lysosomes accompanied by concomitant inhibition of endocytosis mediates translocation and prolonged retention of MHCII on cell surface.

D15

**ANTIBODIES-BASED APPROACH TO REDUCE INTRACELLULAR TDP-43 PROTEINOPATHY**

Silvia Pozzi^1,3^, Sai Sampath Thammisetty^1^, Philippe Codron^2^, Geneviève Soucy^1^, Reza Rahimian^1^, Karine Valérie Plourde^1^, Laurence Renaud^1^, Pierre Junior Cordeau^1^, Kallol Dutta^1^, Christine Bareil^1^, Daniel Phaneuf^1^, Jasna Kriz^1,3^, Claude Gravel^1,3^, Jean-Pierre Julien^1,3^

*^1^ CERVO Brain Research Centre, Integrative Neuroscience and Experimental Therapies, Québec, Canada*

*^2^ University of Angers, UMR CNRS 6015, INSERM U1083, Angers, France*

*^3^ University Laval, Department of Psychiatry and Neuroscience, Québec, Canada*

TDP-43 proteinopathy is characterized by a consistent cytoplasmic mislocalization and aggregation of the protein TDP-43. This event is specific for Amyotrophic Lateral Sclerosis (ALS) and Frontotemporal Dementia (FTD) but has also been observed in other neurodegenerative disorders (Arai et al. 2014). Different studies highlighted the sensitivity of the RRM1 domain of the protein in TDP-43 proteinopathy induction (Chang et al. 2013; Shodai et al. 2013) and NF-kB pathway activation (Swarup et al. 2011).

We recently developed two antibody-based approaches, to target and overcome TDP-43 proteinopathy. In particular, a monoclonal full-length antibody (Pozzi et al. 2020) and a single chain antibody (Pozzi et al. 2019), both specifically directed against the RRM1 domain of TDP-43, were generated and tested in ALS/FTLD mouse models.

The full-length antibody recognized specifically the cytoplasmic fraction of TDP-43 in cells, animal models and ALS human tissues. In neuronal cells the antibody reduced the cytoplasmic TDP-43 by activating the TRIM-21/proteasome degradative pathway. In tissues of treated mice, the antibody decreased the levels of cytoplasmic TDP-43 and nuclear p65, the active subunit of NF-kB. The single chain antibody demonstrated to rescue motor and cognitive impairments in animal models. It reduced the amount of cytoplasmic accumulated TDP-43 by targeting the protein to degradative pathways and it blocked the interaction between TDP-43 and p65, decreasing neuroinflammation in mice.

With these studies we demonstrated the efficacy of two antibody-based approaches against the RRM1-domain of TDP-43 in reducing the intracellular TDP-43 proteinopathy and rescuing deficits in ALS/ FTLD mouse models.

D16

**NEW INSIGHTS INTO THE DOWN SYNDROME-RELATED BRAIN CELLS PHENOTYPES STUDIED IN IPS-DERIVED OLIGOCORTICAL SPHEROIDS**

Sanjeev Rampam^1^, Aneka Pradhan^1^, Jack Cardini^1^, Ronni Kurzion^2^, Zhen Li^3^, Jenny Klein^2^, Shai Shimoni^2^, Tarik Haydar^3, 2^, Ella Zeldich^2^

*^1^ Boston University, Biomedical Engineering, Boston, USA*

*^2^ Boston University School of Medicine, Anatomy and Neurobiology, Boston, USA*

*^3^ Children's National Research Center, Center for Neuroscience Research, Washington DC, USA*

Down Syndrome (DS), a neurodevelopmental disorder which arises from the presence of an extra copy of chromosome 21, is linked with an increased risk of developing early-onset Alzheimer's disease (AD). Three dimensional (3D) oligocortical spheroids (OLS) generated from patient-derived induced pluripotent stem (iPS) cells permit innovatory observation of brain development; they have previously been validated to recapitulate the cortical architecture of the human fetal brain along with the presence of neurons, oligodendro-cytes and astrocytes. Herein we present a thorough analysis of the DS-derived OLS utilizing immunohistochemistry and single-cell RNA sequencing (scRNA-seq) comparing cellular diversity, cell lineage development, and the AD-related pathological features in isogenic trisomic and euploid OLS. We have recently reported myelin-related abnormalities in DS human brain and Ts65Dn mouse model. Using this novel in vitro 3D system, we evaluated oligo-dendrocyte development and myelin production in trisomic and euploid OLS. We have specially developed a MATLAB application to quantify fate-and stage-specific markers. Trisomic OLS faithfully recapitulated the development of AD-related pathology including depositions of amyloid-beta and activation of caspase 3 accompanied by the profoundly reduced spheroid volume. Our scRNA-seq analysis identified 15 different cell clusters with diverse transcriptional signatures. Subsequent gene ontology analysis revealed that the excitatory neuron clusters demonstrate significant enrichment for processes related to enhanced transcription and translation as well as for the genes implicated previously in AD. We uncovered a profound dysregulation of the pathways mediating axonal guidance, neuron migration, cell-to-cell adhesion and nervous system development in trisomic excitatory neurons. This implies that these pathways can be implicated in neuronal networks, connectivity, and synaptogenesis, and contribute to the intellectual deficits observed in DS individuals. Our translational, human-directed study contributes to the understanding of DS-associated cell pathology and comorbidity with AD.

D18

**LIPOXYGENASE MEDIATORS AND MICROSTRUCTURAL WHITE MATTER INTEGRITY IN ALZHEIMER'S DISEASE AND SUBCORTICAL ISCHEMIC VASCULAR DISEASE**

Julia Zebarth^1,2^, Di Yu^1,2^, Fenghua You^1,2^, Joel Ramirez^1^, Pak Cheung Chan^1,2^, Mario Masellis^1,2^, Richard Swartz^1,2^, Krista L. Lanctôt^1,2^, Nathan Herrmann^1,2^, Demitrios J. Sahlas^3^, Jacqueline A. Pettersen^4^, Sandra E. Black^1,2^, Ameer Taha^5^, Walter Swardfager^1,2^

*^1^ Sunnybrook Research Institute, Hurvitz Brain Sciences Program, Toronto, Canada*

*^2^ University of Toronto, Pharmacology and Toxicology, Toronto, Canada*

*^3^ McMaster Univeristy, Medicine, Hamilton, Canada*

*^4^ University of Northern British Columbia, Medicine, Prince George, Canada*

*^5^ UC Davis, Food Science and Technology, Davis, USA*

Linoleic acid derived lipoxygenase hydroxyoctadecadienoic acid metabolites (9-HODE and 13-HODE) have anti-inflammatory properties but their clinical associations with Alzheimer's disease (AD) and subcortical ischemic vascular disease (SIVD) have not been established. We aim to examine these metabolites with respect to microstructural white matter integrity and vascular brain lesion characteristics. Patients from memory and stroke prevention clinics (n=69) were stratified based on SIVD (minimal vs. abundant white matter hyperintensities [WMH] based on 3.0T structural MRI), and AD (clinical diagnosis). 9-HODE and 13-HODE were extracted from serum using solid phase extraction and concentrations measured by ultra high-performance liquid chromatography mass spectrometry. Microstructural brain tissue integrity was examined as fractional anisotropy (FA) and mean diffusivity (MD) using diffusion tensor imaging. Using multivariate analysis of covariance controlling for age and sex, 13-HODE was lower in people with AD (F_1,68_=6.52, p=0.013, n=23) but not with SIVD (F_1,68_=0.14, p=0.7, n=38). 13-HODE was associated with higher FA in normal appearing white matter (β=0.303, p=0.017), negatively associated with periventricular WMH-FA (β=-0.381, p=0.048), and positively associated with with periventricular WMH-MD (β=0.257, p=0.048). In patients without AD, 9-HODE and 13-HODE were negatively associated with WMH volume (β=-0.322, p=0.018 and β=-0.324, p=0.019). Additionally, 9- HODE was positively associated with FA in normal appearing white matter (β=0.341, p=0.041) and periventricular WMH-MD (β=0.381, p=0.025). Anti-inflammatory linoleic acid oxylipins were associated with preserved white matter integrity, smaller vascular brain lesion volumes, and vascular brain lesion microstructural characteristics. AD was associated with a relative deficit in these mediators.

D19

**LINKING MOLECULAR ABNORMALITIES TO BEHAVIORAL DEFICITS USING A ZEBRAFISH MODEL FOR TAUOPATHIES**

Yunlu Zhu^1^, Paige Leary^1,2,3^, Qing Bai^4^, Edward Burton^4,5^, David Schoppik^1,2,3^

*^1^ New York University School of Medicine, Neuroscience Institute, New York, USA*

*^2^ New York University School of Medicine, Department of Otolaryngology, New York, USA*

*^3^ New York University School of Medicine, Department of Neuroscience & Physiology, New York, USA*

*^4^ University of Pittsburgh School of Medicine, Department of Neurology, Pittsburgh, USA*

*^5^ Pittsburgh VA Healthcare System, Geriatric Research Education and Clinical Center, Pittsburgh, USA*

Tauopathies are neurodegenerative diseases characterized patho-logically by accumulation of abnormal Tau in the brain. However, it remains unclear how these molecular and cellular dysfunctions lead to behavioral deficits, especially during the early stages of patho-genesis. To dissect disease mechanisms across multiple biological scales, we generated a zebrafish model of progressive supranuclear palsy (PSP), a primary tauopathy causing unexpected falls in patients early in disease progression, by expressing human 0N/4R-Tau in the evolutionarily conserved vestibulospinal (VS) nucleus. Human Tau-expressing zebrafish exhibit impaired balance control during free-swimming while maintaining normal locomotor ability compared to their siblings. Functional imaging of the VS nucleus shows decreased calcium signals in Tau-expressing neurons in response to tilt stimulus. This altered neuronal activity correlates with Tau phos-phorylation in VS neurons. Interestingly, we also observed ectopic accumulation of acidic organelles in the cell bodies of Tau-positive neurons, suggesting abnormal lysosomal function. Taken together, our zebrafish PSP model allows us to understand molecular and cellular mechanisms of balance deficits in tauopathies and can be a powerful system for preclinical drug screening and evaluation of potential therapeutic targets.

D20

**ROLE OF ASTROCYTE-DERIVED GDNF IN NEURONAL PROTECTION AND BRAIN RECOVERY AFTER FOCAL ISCHEMIC STROKE**

Zhe ZHANG^1^, Nannan Zhang^2^, Shinghua Ding^1,2^

*^1^ University of Missouri-Columbia, Bioengineering, Columbia, USA*

*^2^ University of Missouri-Columbia, Dalton Cardiovascular Research Center, Columbia, USA*

Focal ischemic stroke (FIS) is a leading cause of human death. Reactive gliosis is a hallmark of FIS characterized by dramatic spatial and temporal changes of morphology of reactive astrocytes, gene expression in reactive astrocytes and glial scar formation in peri-infarct region (PIR). Glial cell-derived neurotrophic factor (GDNF) was originally isolated from a rat glioma cell-line supernatant and is a potent survival neurotrophic factor. In our previous study, we found reactive astrocytes expressed enhanced GDNF after photothrombosis (PT)-induced FIS, and deletion of GDNF in astro-cytes using inducible and conditional knockout mice lead to increased neuronal death and brain infarction after PT. Moreover, deletion of GDNF reduced proliferation of reactive astrocytes compared based on Brdu and Ki67 staining in the PIR, indicating that astrocytic GDNF can promote neural regeneration. Furthermore, behavioral tests showed that deletion of GDNF increased motor function impairment after PT. Our study indicate that endogenous GDNF in reactive astrocytes has beneficial effect on poststroke brain repair. Here we overexpress GDNF in reactive astrocytes using AAV virus injection for gain-of-function study. We found astrocyte-specific GDNF overexpression can significantly decreased infarct volume and promoted motor function recovery after PT. In summary, our study suggests that reactive astrocytes-derived GDNF plays important roles in reducing neuronal death and brain damage through a non-cell autonomous effect after FIS, and promoting endogenous neurotrophic factor release from reactive astrocytes might be a potential approach in stroke therapy.

D21

**PRO-SURVIVAL F-ACTIN REORGANIZATION IN RESPONSE TO NEURONAL ISCHEMIC INJURY**

Barbara Calabrese^1^, Michael Lingelbach^2^, Steven Jones^3^, Uri Manor^2^, Tatjana Svitkina^3^, Henry Higgs^4^, Andy Shih^5^, Shelley Halpain^1^

*^1^ UCSD, Department of Biology, La Jolla, USA*

*^2^ The Salk Institute, Department of Biology, La Jolla, USA*

*^3^ University of Pennsylvania, Department of Biology, Philadelphia, USA*

*^4^ Geisel School of Medicine, Department of Biochemistry, Hanover, USA*

*^5^ Seattle Children's Research Institute, Center for Dev. Biology and Reg. Medicine, Seattle, USA*

One of the early consequences of ischemic injury and glutamate receptor hyperactivation is neuronal swelling or cytotoxic edema, caused by sodium and chloride entry and followed by irreversible damage to the plasma membrane.

Here we show that ischemia and excess NMDA receptor activation cause actin filaments to rapidly reorganize within the somatoden-dritic compartment. Normally F-actin is concentrated within den-dritic spines, with little F-actin in the dendrite shaft. However, after incubation of neurons with NMDA, F-actin depolymerizes within dendritic spines and polymerizes into long filament bundles within the dendrite shaft and soma. A similar “actinification” of the somatodendritic compartment occurs after oxygen/glucose deprivation *in vitro*, and in mouse brain after photothrombotic stroke *in vivo*. These actin changes spontaneously reverse within 1-2 hours, if the exposure to NMDA is transient. We find that actinification is triggered by neuronal swelling, which depends on Na^+^ and Cl^−^ influx.

However, it requires also Ca^2+^ entry from the extracellular space, rather than from the intracellular stores. It is mediated by activation of the F-actin elongation factor inverted formin-2 (INF2), modulated by acetylation and dependent on the disassembly of F-actin in den-dritic spines. Silencing of INF2 renders neurons more vulnerable to NMDA-induced membrane leakage and cell death. Inhibition of formin activity markedly increases ischemic infarct severity *in vivo*. Overall, these results uncover a novel neuron-specific pro-survival response that protects neurons from ischemic death.

D22

**PROTECTIVE EFFECTS OF ESTROGEN AND NANOPARTICLE DELIVERY TO ATTENUATE MYELIN LOSS AND NEURONAL DEATH AFTER SPINAL CORD INJURY**

Kelsey Drasites^1^, April Cox^1^, Mollie Capone^1,2^, Ramsha Shams^1,2,3^, Denise Matzelle^1,4^, Giovanna Leone^1,2,3^, Alexandra Myatich^1,2,3^, Dena Garner^3^, Mikhail Bredikhin^5^, Alexey Vertegel^5^, Naren Banik^1,2,4^, Azizul Haque^2^

*^1^ The Medical University of South Carolina, Neurosurgery, Charleston, USA*

*^2^ The Medical University of South Carolina, Microbiology and Immunology, Charleston, USA*

*^3^ The Citadel Military College of South Carolina, Health and Human Performance, Charleston, USA*

*^4^ Ralph H. Johnson Veterans Administration Medical Center, VA, Charleston, USA*

*^5^ Clemson University, Bioengineering, Clemson, USA*

Spinal cord injury (SCI) is associated with devastating neurological problems affecting more than 11,000 Americans each year. Although several treatment agents have been proposed and tested, no FDA-approved pharmacotherapy is available for treating SCI. We have recently demonstrated that estrogen (E2) acts as an antioxidant and anti-inflammatory agent, attenuating gliosis in SCI. We have also demonstrated that nanoparticle-mediated focal delivery of E2 to the injured spinal cord decreases lesion size, reactive gliosis, and glial scar formation. The current study tested *in vitro* effects of E2 on reactive oxygen species (ROS) and calpain activity in microglia, astroglia, macrophages, and fibroblasts, which are believed to participate in the inflammatory events and glial scar formation after SCI. E2 treatment decreased ROS production and calpain activity in these glial cells, macrophages, and fibroblast cells *in vitro*. This study also designed and tested the efficacy of fast and slow release nano-particle-E2 constructs in a rat model of SCI. Focal delivery of E2 via nanoparticles increased tissue distribution of E2 over time, attenuated cell death, and improved myelin preservation in injured spinal cord. Specifically, the fast release nanoparticle-E2 construct (PLGA-E2) reduced the Bax/Bcl-2 ratio in injured spinal cord tissues, and the slow release nanoparticle-E2 construct (PLA-E2) protected myelina-tion below the lesion site in penumbra. These data suggest that this novel delivery strategy of E2 to the lesion site of spinal cord may decrease inflammation and improve functional outcomes in SCI.

D23

**MODULATING MOLECULAR CHAPERONES: A POTENTIAL THERAPEUTIC APPROACH FOR X-LINKED CHARCOT-MARIE-TOOTH (CMT1X) DISEASE**

Sukhmanjit Kaur^1^, Allison Zhang^1^, Brian Blagg^2^, Charles Abrams^3^, Rick Dobrowsky^1^

*^1^ University of Kansas, Department of Pharmacology and Toxicology, Lawrence, USA*

*^2^ University of Notre Dame, Chemistry & Biochemistry, Notre Dame, USA*

*^3^ University of Illinois, Neurology, Chicago, USA*

CMT1X is an inherited peripheral neuropathy caused by mutations in the GJB-1 gene that encodes for connexin 32 (Cx32). Despite being the second most common form of CMT neuropathies, there are no pharmacologic treatments for CMT1X. We have developed “novologues” as orally bioavailable novobiocin analogues that manifest neuroprotective activity by modulating the expression of heat shock protein 70 (Hsp70). The novologue, KU-596, is in clinical trials for treating a metabolic neuropathy and we examined if it may improve neuropathic symptoms in Cx32 deficient (Cx32def) mice, an authentic mouse model of human CMT1X. Cx32def mice develop a significant reduction in motor nerve conduction velocity (MNCV, ∼45 m/sec) and compound muscle action potential (CMAP,∼20- 25mV) compared to wild-type mice (MNCV, ∼60 m/sec; CMAP,∼40 mV). Beginning at 4 months of age, 5 months of daily KU-596 therapy significantly improved MNCV (∼ 55-60 m/sec) and CMAP (∼ 30 mV). To investigate whether these effects were Hsp70 dependent, Cx32def x Hsp70 knockout mice were treated with KU-596. While the deletion of Hsp70 did not affect the development of peripheral neuropathy, the therapeutic efficacy of KU-596 was Hsp70 dependent since, there were no improvements in MNCV and CMAP. To determine if KU-596 may be effective in other models of CMT1X, we utilized T55I x Cx32def mice. T55I is a common Cx32 mutation in human CMT1X patients, which leads to accumulation of Cx32 in the endoplasmic reticulum. These mice develop similar deficits in MNCV (∼45-50 m/sec), CMAP (∼20 mV). Five months of daily treatment with KU-596 improved MNCV (∼ 60 m/sec) but did not significantly improve CMAP. Collectively, our data suggests that modulating Hsp70 with KU-596 may be beneficial for treating CMT1X and that efficacy may not be limited by the nature of the underlying genetic mutation in the GJB-1 gene.

D24

**TRANSCRIPTIONAL PROGRAMS PROTECTING RETINAL GANGLION CELLS FROM INJURY-INDUCED DEGENERATION**

Aboozar Monavarfeshani^1,2^, Songlin Zhou^1^, Feng Tian^1^, Chen Wang^1^, Joshua Sanes^2^, Zhigang He^1^

*^1^ BCH, Neurology, 1F.M. Kirby Neurobiology Center, Boston, USA*

*^2^ Harvard University, Department of Molecular and Cellular Biology, and Center for Brain Science, Cambridge, USA*

Similar to other central nervous system neurons, retinal ganglion cells (RGCs), the projection neurons of the retina, fail to regenerate after injury. In glaucoma, as the second leading cause of blindness, degeneration of RGC axons and subsequent death of their soma are two key pathological events that lead to irreversible loss of vision. However, no effective treatment targeting RGC vulnerability is yet available. A key obstacle toward developing novel therapeutics is the lack of sufficient understanding of mechanisms that regulate RGC survival and axon regeneration. Here, we leveraged the mouse optic nerve crush model – in which ∼80% of RGCs die within 2 weeks after insult to their axon – and undertook a large-scale in vivo CRISPR screen to identify key regulators of such mechanisms. Our screen revealed multiple genes whose removal from retinal cells promoted RGC survival and/or axon regeneration. One of the strongest protective phenotypes belongs to the knockout of the gene encoding c-Jun N-terminal kinases-Interacting Protein 3 (JIP3; Also known as MAPK8IP3). Then, we performed transcriptomic analysis of RGCs with or without JIP3 at different time points after optic nerve crush to further investigate molecular programs underlying JIP3-dependent RGC survival, and identified candidate genes that regulate survival of RGCs.

D25

**VISION AND MOTOR DEFICITS DUE TO LOSS OF VPS11 FUNCTION IN A ZEBRAFISH MODEL OF GENETIC LEUKOENCEPHALOPATHY**

Shreya Banerjee^1^, Lillian Ranspach^1^, Xixia Luo^1^, Lauren Cianciolo^2^, Joseph Fogerty^2^, Brian Perkins^2^, Ryan Thummel^1^

*^1^ Wayne State University School of Medicine, Ophthalmology, Visual and Anatomical Sciences, Detroit, USA*

*^2^ Cole Eye Institute, Cleveland Clinic, Ophthalmic Research, Cleveland, USA*

Purpose: Genetic Leukoencephalopathies (gLEs) are white matter disorders affecting the central nervous system, causing progressive abnormalities in the visual and motor systems. A mutation in *VPS11* has been identified as a causative allele of gLE in Ashkenazi Jewish individuals, with a high carrier rate of 1:250. VPS11 forms mem-brane-tethering complexes with three additional VPS proteins to control vesicle fusion within the endolysosomal and autophagy pathways. Here, we are characterizing a zebrafish *vps11* mutant as a potential model for gLE. Methods: Behavioral responses to visual and acoustic cues was performed at 5- and 7-days post-fertilization (dpf) using the DanioVision Noldus tracking system. In addition, optokinetic response (OKR) analysis was performed at 5dpf to test visual acuity. Results: Behavioral analysis showed that *vps11* mutant fish could visualize changes in light and dark backgrounds, but OKR analysis indicated the animals were functionally blind and not able to make out an image. Regarding motor movement, no difference in response to alternating light-dark backgrounds was observed between the mutant and wild-type larvae at 5dpf, but a significant reduction in movement of the mutants at 7dpf. Mutants also showed a progressive reduction in movement to non-visual, acoustic stimuli. Together, these results suggest that loss of Vps11 function has a progressive adverse effect on visuomotor system development in zebrafish. Conclusions: Our findings support the use of zebrafish to further characterize the vision and motor defects associated with loss of Vps11 function.

D26

**REDUCED HIPPOCAMPAL INHIBITION AND ENHANCED AUTISM-EPILEPSY COMORBIDITY IN MICE LACKING NEUROPILIN 2**

Carol Eisenberg^1^, Deepak Subramanian^2,3^, Milad Afrasiabi^2^, Patryk Ziobro^1,4^, Jack Delucia^1^, Michael Shiflett^4^, Vijayalakshmi Santhakumar^2,3^, Tracy Tran^1^

*^1^ Rutgers University, Biological Sciences¹, Psychology^4^, Newark, USA*

*^2^ Rutgers NJ Medical School, Pharmacology, Physiology and Neuroscience, Newark, USA*

*^3^ UCR, Molecular, Cell and Systems Biology, Riverside, USA*

The neuropilin receptors and their semaphorin ligands play key roles in brain circuit development by regulating numerous crucial neuronal processes, including synapse maturation and migration of GABAergic interneurons. Consistent with its developmental roles, the neuropilin 2 (Nrp2) locus contains polymorphisms in patients with autism spectrum disorder (ASD). Nrp2 deficient mice show autism-like behavioral deficits and propensity to develop seizures. In order to determine the pathophysiology in Nrp2 deficiency, we examined the hippocampal interneuron subtypes and inhibitory regulation of CA1 pyramidal neurons in mice lacking one or both copies of Nrp2. Immunostaining for interneurons revealed that Nrp2-/- mice have reduced number of parvalbumin, somatostatin and Neuro-peptide Y cells, mainly in CA1. Whole cell recordings identified reduced firing and hyperpolarized shift in resting membrane potential in CA1 pyramidal neurons from Nrp2+/- and Nrp2-/- mice compared to Nrp2+/+ indicating decrease in intrinsic excitability. Simultaneously, the frequency and amplitude of spontaneous inhibitory post-synaptic currents (sIPSCs) are reduced in Nrp2 deficient mice. A convulsive dose of kainic acid evoked electrographic and behavioral seizures with significantly shorter latency, longer duration and higher severity in Nrp2-/- compared to Nrp2+/+ animals. Finally, Nrp2+/- and Nrp2-/-, but not Nrp2+/+, mice have impaired cognitive flexibility demonstrated by reward-based reversal learning, a task associated with hippocampal function. Together these results demonstrate a broad reduction in interneuron subtypes and compromised inhibition in CA1 of Nrp2-/- mice, which could contribute to the heightened seizure susceptibility and behavioral deficits consistent with an ASD/epilepsy phenotype.

This work is supported by the NJ Governor's Council for Medical Research and Treatment of Autism: CAUT17BSP011 to VS, TST; CAUT17BSP022 to TST, MWS; Rutgers BHI Pilot Grants to TST, VS; NIH R01 NS069861, NS097750 to VS; NSF/IOS1556968 to TST.

D27

**TRANSCRIPTOMIC ANALYSIS ACROSS POSTNATAL DEVELOPMENT IN HEALTHY AND MECP2 DEFICIENT MICE REVEALS ABERRANT ASTROCYTE MATURATION**

Raymundo Hernandez^1,2^, Leanne Holt^3^, Natasha Pacheco^4^, Michelle Olsen^2^

*^1^ Virginia Polytechnic Institute and State University, Translational Biology, Medicine, and Health, Blacksburg, USA*

*^2^ Virginia Polytechnic Institute and State University, School of Neuroscience, Blacksburg, USA*

*^3^ Icahn School of Medicine at Mount Sinai, Nash Family Department of Neuroscience, Friedman Brain Institute, New York, USA*

*^4^ National Institute on Aging, Laboratory of Epidemiology and Population Science, Baltimore, USA*

Rett Syndrome (RTT) is an X-linked neurodevelopmental disorder caused by mutations in the MECP2 gene. The MeCP2 protein is a critical transcriptional regulator in the CNS responsible for neuro-typical function and maturation. Persons with RTT experience symptom onset after a period of typical development between 6 to 18 months of age, causing regressions in motor abilities, development of breathing abnormalities, and intellectual impairments requiring life-long care. Mecp2-/y Mouse models similarly recapitulate these symptoms and provide a basis for understanding cellular contribution to disease progression. Previous studies have demonstrated that astrocytes contribute to RTT pathogenesis, but the exact mechanisms are not well understood. Astrocytes undergo postnatal maturation alongside neurons, indicated by changes in cellular function, and development of a complex morphological phenotype. Surprisingly little is understood regarding astrocyte gene expression during this period of morphological maturation. Using cell-specific isolation of cortical astrocytes, we evaluated the gene expression in wilde-type (WT) and Mecp2-/y mice from early postnatal development through adulthood.

We found the highest number of differentially expressed genes (DEGs) during the period of murine astrocyte morphogenesis (P14 – P28) in WT animals. While WT astrocytes have enrichment of pathways associated with cellular morphology and maturation, these pathways are disrupted in Mecp2-/y astrocytes. We also used an AAV construct to drive astrocyte-specific expression of fluorescent mem-brane markers, determining that Mecp2-/y animals have deficits in astrocyte morphogenesis at the end of this developmental period at P28, but not prior to it at P14. These results suggest that disruption of astrocyte gene pathways in RTT may have functional relevance.

D28

**AUGMENTING THE PROCESS OF NEUROGENESIS USING NATURAL AND SYNTHETIC COMPOUNDS**

Kanwal Iftikhar, Maryam Niaz, Shabana Simjee

*International Center for Chemical and Biological Sciences, Pharmacology, Karachi, Pakistan*

The adult mammalian brain nurtures neural stem cells (NSCs) that participate in the neurogenesis—the phenomenon refers to the origin of new and functional neurons. This event involves multiple complex processes that include proliferation, differentiation and fate determination of NSCs and the maturation, migration, survival and functional integration of developing neurons in the adult brain.

These NSCs reside in the brain as specialized micro-environment called neurogenic niche. The most studied and accepted neurogenic niches are the sub-ventricular zone [SVZ] of the lateral ventricles and the sub-granular zone [SGZ] of the hippocampal dentate gyrus.

A growing body of evidence supports the link between the variable levels of neurogenesis and brain function in the normal and affected brain. The existence of neural progenitor cells in adult CNS has opened a novel dimension of research to explore the potential of these cells for treatment of neurological disorders.

The present study is designed to evaluate the effects of natural and synthetic compounds on neuronal proliferation, maturation, survival and differentiation. This study might help in discovery of new era in medical science for improving the treatment of various neuro-degenerative diseases through the mechanism of neurogenesis.

D29

**SONIC HEDGEHOG SIGNALING AS AN INTERMEDIARY BETWEEN RETINAL INPUTS AND ASTROCYTES FOR THE RECRUITMENT OF GABAERGIC INTERNEURONS**

Rachana Deven Somaiya^1,2^, A. Denise R. Garcia^3,4^, Michael A. Fox

2,5,6

*^1^ Virginia Tech, Translational Biology, Medicine, and Health Graduate Program, Blacksburg, USA*

*^2^ Fralin Biomedical Research Institute at Virginia Tech Carilion, Center for Neurobiology Research, Roanoke, USA*

*^3^ Drexel University, Department of Biology, Philadelphia, USA*

*^4^ Drexel University, Department of Neurobiology and Anatomy, Philadelphia, USA*

*^5^ Virginia Tech, School of Neuroscience, College of Science, Blacksburg, USA*

*^6^ Virginia Tech, Department of Biological Sciences, College of Science, Blacksburg, USA*

Visual thalamus receives direct inputs from retinal ganglion cell (RGC) axons and is important for both image-forming and non-image-forming visual functions. Recent studies have shown that these RGC inputs play important roles in the development of cell types and circuits in these regions. For example, retinal inputs regulate the long-distance recruitment of GABAergic interneurons into two regions of visual thalamus – the dorsal and ventral lateral geniculate nuclei. Our lab recently discovered that these retinal inputs induce astrocytes to generate fibroblast growth factor 15 (FGF15), a potent motogen that is essential for interneuron migration into these regions. However, how retinal inputs induce the astrocytic expression of FGF15 remains unresolved. Here, we tested the role of RGC-derived Sonic Hedgehog (SHH) in this process since RGCs are known to release SHH in the brain and SHH is known to induce FGF15 expression in other parts of the embryonic brain. Using trans-criptomic analysis, *in situ* hybridization, and reporter line, we observed that thalamus-projecting RGCs express SHH, and SHH receptors and signaling pathway components are expressed in the developing visual thalamus. Specifically, we discovered thalamic astrocytes express Patched-1, Smoothened, and Glioma-associated oncogene transcription factors. Our data revealed a significant decrease in the thalamic expression of *Fgf15* mRNA and GABAergic interneuron number in the mice lacking SHH specifically from RGCs, suggesting the importance of this axo-glial signaling pathway in the development of visual thalamus. Overall, our results shed light on novel ways in which astrocytes mediate the recruitment of inter-neurons into neonatal visual thalamus.

D30

**EXPLORING THE ROLE OF ASTROCYTE SYSTEM XC-IN ACUTE SEIZURE GENERATION AND EPILEPTOGENESIS**

Samantha Sutton, Audrey Mellan, Sheila Sears, Sandra Hewett

*Syracuse University, Biology/Neuroscience, Syracuse, USA*

System x_c_^−^ (Sx_c_^−^) is a heteromeric amino acid antiporter composed of a substrate-specific light chain (xCT) and a heavy chain (4f2hc), the latter of which tethers it to the plasma membrane. It plays a vital role in maintaining both intracellular glutathione (via import of cystine) and extracellular glutamate (via export of glutamate) levels in the brain. Previously, we demonstrated that mice harboring a natural null mutation in the gene that encodes for xCT (Slc7a11^sut/sut^), and thus have no functional Sx_c_^−^ expression in any of their cells (Sx_c_^−^ global null mice), have an excitatory/inhibitory (E/I) imbalance in brain. Specifically, we found that Slc7a11^sut/sut^ mice are more excitable (i.e., have lower convulsive seizure thresholds) than their wild type (Slc7a11^+/+^) littermates after acute challenge with the chemoconvulsant pentylenetetrazol (PTZ). Despite this, Slc7a11^sut/sut^ mice show behavioral signs of hypoexcitability following a sub-chronic, sub-convulsant PTZ kindling paradigm. To wit, the percent of *SLC7a11*^*sut/sut*^ mice that kindle — defined as exhibiting a convulsive seizure on three consecutive days of injection — is significantly lower than their *SLC7a11*^+*/*+^littermate controls. However, since system x_c_^−^ is predominantly expressed in cells known as astro-cytes in the brain, the goal of this study was to test whether mice with targeted loss of Sx_c_^−^ in astrocytes only (astrocyte conditional knockout mice, AcKO) will recapitulate the results seen in our global null mice. Toward this end, we have begun to measure the acute seizure and kindling thresholds of AcKO mice in comparison to their wild-type littermate controls. Preliminary data, to be presented, support a role for astrocyte Sx_c_^−^ in the maintenance of E/I balance in brain. Supported by NINDS R01 NS051445 and R01 NS105767.

D31

**MYELINATION DISTURBANCES IN THE CORPUS CALLOSUM OF RATS SUBJECTED TO THE VALPROIC ACID MODEL OF AUTISM SPECTRUM DISORDER**

Nonthué Uccelli^1^, Marianela Traetta^1,2^, Martin Codagnone^1,2^, Einav Litvak^1^, Juana Pasquini^3^, Analía Reinés^1,2^

*^1^ UBA-CONICET, Instituto de Biología Celular y Neurociencias (IBCN), Buenos Aires, Argentina*

*^2^ Facultad de Farmacia y Bioquímica, UBA, Cátedra de Farmacología, Buenos Aires, Argentina*

*^3^ UBA-CONICET, Departamento de Química Biológica, IQUIFIB, Buenos Aires, Argentina*

Autism Spectrum Disorder (ASD) is a group of neurodevelopmental disabilities characterized by behavioral impairments and atypical brain connectivity. Imaging studies reported altered structure of the corpus callosum (CC) in patients. However, the underlying ultrastructural and cellular changes remain elusive. The valproic acid (VPA) model is a valuable strategy to study ASD neurobiology. In this model, we have previously demonstrated hypomyelination in the CC of juvenile VPA rats. As ASD has an early onset, the aim of this work was to compare the ultrastructural and cellular characteristics of the CC in infant and juvenile VPA rats. Pregnant Wistar rats were administrated with VPA (450mg/kg) or saline on gestation day 10.5. At PND15 (infant) and 36 (juvenile), CC ultrastructure and oligo-dendroglial lineage were evaluated by transmission electron micro-scopy and immunofluorescence, respectively. Oligodendroglial lineage was studied with PDGFαR (for precursors) and CC1 (for myelinating oligodendrocytes) markers. The CC of infant VPA rats showed a reduced percentage of myelinated axons and similar myelin sheath ultrastructure when compared with controls. Both precursor and mature oligodendrocyte densities were preserved in the CC of infant VPA rats. The CC of juvenile VPA rats evidenced a reduced percentage of myelinated axons and disturbed myelin compaction; while oligodendrocyte precursors were increased, mature oligo-dendrocytes decreased. Our results indicate that myelin alterations are present in the CC of infant VPA rats, preceding the disturbances in myelin sheath compaction and the disbalance in the oligodendro-glial lineage observed in juvenile rats. Our work suggests that an impaired axon-oligodendroglia communication could underlie CC connectivity deficits in ASD.

D32

**STRIATIN-3 IS A NOVEL RAC1 INTERACTOR IN SCHWANN CELLS**

Michael Weaver^1^, Marta Pellegatta^2^, Luciana Frick^1^, Caterina Berti^3^, Marilena Palmisano^4^, Scott Ferguson^5^, Clementine Namba^6^, Matthias Selbach^7^, Florian Paul^7^, Lawrence Wrabetz^1^, Yannick Poitelon^8^, M. Laura Feltri^1^

*^1^ SUNY Buffalo, HJKRI, Buffalo, USA*

*^2^ San Raffaele Scientific Institute, AGIU, Milan, Italy*

*^3^ NYU, Pathology, NYC, USA*

*^4^ AstraZeneca, Biologics, Basiglio, Italy*

*^5^ SUNY Buffalo, Pharmaceutical Sciences, Buffalo, USA*

*^6^ Meharry Medical College, Medicine, Nashville, USA*

*^7^ Max Delbrück Center, Molecular Medicine, Berlin, Germany*

*^8^ Albany Medical Center, Neuroscience, Albany, USA*

During development, Schwann cells (SCs) undergo extensive cyto-skeletal reorganization as they insert cytoplasmic extensions into axon bundles to sort, ensheath, and myelinate individual axons. Similarly, following peripheral nerve injury there is extensive actin polymerization around Schmidt-Lantermann incisures (SLIs) as Schwann cells differentiate into a repair phenotype. Both of these processes are regulated by Rac1. Our laboratory previously demon-strated that Rac1 activation in SCs is driven by engagement of α6β1 integrin with laminins, and that this is essential for peripheral nerve development. We then performed a proteomic screen to look for novel Rac1 interactors in peripheral nerves and identified striatin-3 (Strn3) as a candidate. Strn family proteins (Strn1/3/4) function as the core scaffolding proteins of STRIPAK (STRiatin-Interacting Phosphatase And Kinase) complexes, which are capable of regulating the Hippo pathway. Our group previously demonstrated that the downstream effectors of the Hippo pathway, Yap/Taz, are critical for myelin development. Knockdown of Strn3 in primary rat SCs reduces proliferation and disrupts their association with axons. Using Strn3 floxed mice expressing P0-Cre, we have specifically ablated Strn3 in SCs (Strn3^SCKO^). Sciatic nerves of Strn3^SCKO^ mice demonstrate mild radial sorting defects and hypomyelination. Strn3 null SCs isolated from these animals have impaired cell elongation, process extension, and lamellipodia formation, similar to SCs deficient in Rac1. Our work will investigate mechanisms linking the STRIPAK complex with Rac1 and the Hippo pathway in SCs. Additionally, this work seeks to define the role of Strn3 and the STRIPAK complex in SC development and injury response.

D34

**LONG TERM CONSEQUENCES OF MILD TBI/ CONCUSSION IN ASTROCYTES AND NEURONS**

Dzenis Mahmutovic^1,2^, Oleksii Shandra^1,2^, Owen Leitzel^1,2^, Stefanie Robel^1,2^

*^1^ Virginia Polytechnic Institute and State University, Neuroscience, Blacksburg, United States*

*^2^ Fralin Biomedical Research Institute, Glial Center, Roanoke, United States*

Traumatic brain injury (TBI) affects 1.7-3.7 million people in the US alone every year. Neurons transmit and receive signals and astro-cytes carry out many homeostatic and structural functions which are critical to support neurons. Previously, we have demonstrated that within minutes of mild TBI/ concussion (mTBI), expression of critical homeostatic proteins including those involved in glutamate and potassium buffering (e.g. Glutamate transporter 1, Glutamine synthetase, Kir4.1) is reduced. Additionally, there are signs of early damage to neuronal nuclear protein NeuN, which is a regulator of mRNA splicing. We hypothesized that early astrocyte dysfunction after mTBI will lead to subsequent neuronal dysfunction. To test this, we used C57Bl/6J mice of either sex in a model of repeated mTBI. We used qualitative and quantitative semi-automated analysis to evaluate astrocyte and neuronal expression in the cortex in mice days, weeks, and up to 6-months post TBI. We observed Kir4.1 and Glt-1 downregulation at all time points including 6 months after mTBI. As early as 1-day post-injury, in areas of Glt1 and Kir4.1 downregulation, subsets of cortical neurons lacked or downregulated NeuN. However, using fluorescent Nissl staining we found that these neurons are present. Future studies will determine if NeuN-lacking neurons are functionally impaired.

D35

**THE ROLE OF ASTROCYTIC NAMPT IN BRAIN PROTECTION AND REACTIVE ASTROGLIOSIS AFTER ISCHEMIC STROKE**

Nannan Zhang

*University of Missouri-Columbia, Dalton Cardiovascular Research Center, Columbia, USA*

We previously demonstrated that nicotinamide phos-phoribosyltransferase (NAMPT), a rate limiting enzyme in the salvage pathway of NAD^+^ biosynthesis, is primarily expressed in neurons under normal conditions and is brain protective after ischemic stroke in a mouse model of photothrombosis (PT). In the present study, we showed that NAMPT is largely upregulated in reactive astrocytes at peri-infarct region (PIR) after PT, suggesting astrocytic Nampt is a stress responsive gene. To test the role of astro-cytic NAMPT in neuronal and brain protection, we generated astro-cyte specific NAMPT conditional knockout (cKO) mice by crossing GFAP-Cre with floxed Nampt (Nampt^f/f^) mice, i.e., GFAP-Nampt^+/-^ and GFAP-Nampt^−/-^ cKO mice. When subject to PT, Nampt cKO mice exhibit significant increases in infarct volume and neuronal death in the PIR as compared with wild type (WT) mice. Using immunostaining, we further found that the deletion of Nampt in reactive astrocytes also reduces cell proliferation and reactive astro-gliosis in the PIR. Overexpression of NAMPT in astrocytes using AAV vector reduced brain infarction after PT and increased Sox2+ neuronal progenitor cells. Our study thus demonstrate that astrocytic NAMPT is brain protective and contributes to reactive gliosis and cell proliferation in focal ischemic stroke.

D39

**SHORT-TERM EXPOSURE TO MANGANESE MODIFIES PROTEIN TRANSLATION VIA PI3K/AKT SIGNALING IN GLIAL CELLS**

Jazmin Soto Verdugo, Mireya Castillo-Montesinos, Luis Mario Sánchez-Palestino, Jessica Gabriela Tovar-Ramírez, Luisa Clara Regina Hernández-Kelly, Arturo Ortega

*Centro de Investigación y de Estudios Avanzados del Instituto Politécnico Nacional, Toxicology, Mexico City, Mexico*

Glutamate (Glu), the major excitatory neurotransmitter in the nervous system, activates a wide variety of signal transduction cascades involved in the regulation of protein synthesis. Although protein translation is an exquisitely regulated process, translational dysregulation has been observed in many neurodegenerative disorders. Manganese (Mn) is an essential trace element, that in high doses exerts serious oxidative and neurotoxic effects. An established consequence of Mn neurotoxicity is the disruption of the glutamate/ glutamine (Glu/Gln) cycle, leading to an excitotoxic insult. The molecular mechanisms mediating Mn-induced neurotoxicity, particularly in the context of the Glu/Gln cycle, are not yet fully understood. Hence, we decided to investigate the effect of Mn short-term exposure in signaling pathways involved in protein synthesis, such as the phosphatidylinositol 3 kinase (PI3K)/protein kinase B (Akt) cascade that regulates the Glu/Gln shuttle. To this end, we decided to use Bergmann glial cells (BGC) primary cultures, a well-established model of glial/neuronal interactions. Confluent BGC monolayers were exposed to MnCl_2_ 50-500 µM for different periods and the phosphorylation patterns of Akt, the eukaryotic translation initiation factor 4E-binding protein 1 (4E-BP1), as well as the AMP-activated protein kinase (AMPK), were measured. A time and dose-dependent increase in the phosphorylation status of these proteins was found. An increase in Akt phosphorylation was observed as early as 5 min in a concentration-dependent manner. Pharmacological inhibition of PI3K and the sodium/calcium exchanger blocked these effects. Furthermore, Mn treatment augmented 4E-BP1 phos-phorylation up to 15 min of exposure, and this effect was depleted by inhibition of mTORC1. The Mn-induced increase of AMPK phos-phorylation suggests that Mn could exert a biphasic effect in protein synthesis that might be linked to a reduction in ATP levels and the resulting change in the protein repertoire of these cells. These findings strengthen the idea of the critical role that glial cells have in neurotoxicity development.

D40

**MULTI-SCALE ANALYSIS OF ASTROCYTE MORPHOLOGY AND ULTRASTRUCTURE IN HEALTHY AND ALZHEIMER'S DISEASE MODEL MICE**

Alexandra Schober^1^, Amy Zhou^1^, Yasmina Richa^1^, Tatiana Tibuleac^1^, Braxton Phillips^1^, Rachel Tooth^1^, Maxine Wu^1^, Gavin Frame^1^, J. Benjamin Kacerovsky^1^, Christopher K. Salmon^1^, Tabish A. Syed^2^, Kaleem Siddiqi^2^, Keith K. Murai^1^

*^1^ RI-MUHC, Centre for Research in Neuroscience, Montreal, Canada*

*^2^ McGill University, School of Computer Science & Centre for Intelligent Machines, Montreal, Canada*

Astrocytes comprise a highly complex cell population with diverse structural and functional properties in both the healthy and diseased central nervous system. Astrocytic pathology has been implicated in numerous neurodegenerative diseases, however, their structural rearrangement in brain diseases such as Alzheimer's disease (AD) remain poorly understood. Here, we applied super-resolution imaging and 3-dimensional (3D) electron microscopy approaches to better understand the structural changes of astrocytes in AD model (hAPP/ PS1) mice. Structured illumination microscopy (SIM) of genetically-labeled astrocytes allowed us to capture global modifications to individual astrocyte shape while resolving certain aspects of their subcellular organelle distribution. We observed perturbations to the complex branching pattern of whole astrocytes and additionally, mapped the organization of mitochondria throughout the cell in AD model samples. Results from SIM imaging were complemented with analysis using focused ion beam scanning electron microscopy (FIB-SEM) that enabled 3D serial reconstruction of astrocyte ultrastructure. Using FIB-SEM, we found dramatic restructuring of astrocyte subcompartments in neuropil and astrocytic endfeet, including major alterations of astrocytic process shape and the restructuring/redistribution of mitochondria. Quantitative analysis of SIM and FIB-SEM approaches allowed us to directly compare the astrocytic surface structure and the organelle distribution/ morphology within astrocyte sub-compartments using custom MATLAB scripts, in healthy and AD model tissue. This multi-level structural analysis of astrocytes provides an important understanding of how astrocytes are constructed in the healthy brain and altered with brain disease.

D41

**ORAL MICROBIOME DIVERGENCE BETWEEN MONOZYGOTIC TWINS DISCORDANT FOR MULTIPLE SCLEROSIS SEVERITY**

Anne Boullerne^1^, Demetrios Skias^2^, Mark Maienschein-Cline^3^, Douglas Feinstein^1^

*^1^ University of Illinois at Chicago, Dept Anesthesiology, Chicago, USA*

*^2^ University of Illinois at Chicago, Dept Neurology & Rehabilitation, Chicago, USA*

*^3^ University of Illinois at Chicago, Research Informatics Core, Chicago, USA*

The etiology of multiple sclerosis (MS) remains largely unknown but it is now clear that both genetic and environmental factors play a role. A major factor contributing to the environmental component is the microbiome which can influence disease progression in a variety of autoimmune diseases. While changes in the gut microbiome have been most often characterized, changes in the oral biome, a more easily accessible source, are limited. In the current study we examined the oral microbiome in a pair of monozygotic twins discordant for MS to minimize the genetic contribution to disease. One twin (MSF1) had clinically definite MS; the second (MSF2) was diagnosed with clinically isolated syndrome. Shot-gun sequencing of DNA isolated from saliva was mapped to the NCBI non-redundant DNA database, and identified 7,418 unique bacterial species in the twins. Taxonomic analysis shows that the relative abundance of 3 phyla were higher, and 3 were lower in MSF1 compared to MSF2. Of the 26 species that were present at 1% or greater abundance, 8 were higher in MSF1 compared to MSF2. Pathway analysis identified 116 level 3 BRITE (Bright Target Explorer) functional hierarchies that differed by at least 50% between twins, of which 21 were present at 0.005% or greater abundance. These data demonstrate differences in the oral biome of MS patients with distinct disease severity, suggesting that oral biome analysis may be useful for diagnostic use or assessment of therapeutic interventions. This work was supported by grants BX002625 and 14S-RCS-003 from the Department of Veterans Affairs (DLF), and the UIC Research Informatics Core supported by NCATS through Grant UL1TR002003.

D42

**ROLES FOR ASTROCYTIC RIPK3 SIGNALING IN PARKINSON'S DISEASE PATHOGENESIS**

Nydia Chang, Medha Krishnagiri, Colm Atkins, Brian Daniels

*Rutgers University-New Brunswick/Piscataway, Cell Biology & Neuroscience, Piscataway, USA*

Parkinson's disease (PD) is a neurodegenerative disorder of global concern, imposing an estimated cost of $52 billion per year in the United States, alone. The pathological hallmarks of PD include debilitating motor deficits, driven by progressive degeneration of dopaminergic neurons in the nigrostriatal pathway, an essential circuit for motor function. Emerging evidence suggests that neuroin-flammation is a key player in the pathophysiology of this degenerative process. Astrocytes are the most abundant glial cells in the central nervous system (CNS), where they serve diverse homeo-static functions. However, following inflammatory stimulation, astro-cytes enter a reactive state that can be neurotoxic, resulting in neu-ronal cell death. Numerous studies have now revealed that reactive astrocytes can contribute to clinical neurodegenerative diseases. However, the mechanism through which homeostatic astrocytes become reactive requires further investigation. We have identified damage-associated molecular patterns (DAMPs) released by neurons undergoing cell death as potential drivers of inflammatory astrocyte activation. DAMPs are important “alarm” molecules that initiate several downstream inflammatory responses. Recent work from our laboratory and others has identified receptor-interacting protein kinase-3 (RIPK3) as a central mediator of neuroinflammation. Here, we show that DAMPs activate RIPK3 signaling in astrocytes, resulting in inflammatory astrocyte activation, followed by neuro-toxic effects and neuron loss in the midbrain. Together, these experiments identify novel mechanisms of neurotoxic astrocyte activation with implications for the pathophysiology of PD.

D43

**RETINAL MICROGLIA DEPLETION TO DOWNREGULATE MICROGLIA-MEDIATED INFLAMMATION IN THE DIABETIC RETINA**

Kaira Church, Derek Rodriguez, Difernando Vanegas, Sandra Cardona, Astrid Cardona

*University of Texas at San Antonio, Biology, San Antonio, USA*

**Diabetic retinopathy (DR)**, an incurable eye disease caused by prolonged high glucose levels in the retina, is a leading complication of diabetes mellitus and the leading cause of blindness amongst working age adults. Prolonged high glucose levels damage retinal blood vessels leading to hemorrhages, ischemia and ultimately vision loss. Microglia, the resident immune cells of the central nervous system (CNS), are believed to contribute to the development of DR as they are rapidly activated and respond to transient hyperglycemia, and reset the homeostatic threshold of the retina. As prolonged hyperglycemia persists, micro-angiography occurs resulting in serum proteins and DAMPs from the periphery leaking into the retina. This results in microgliosis and pro-inflammatory cytokine production. Altogether, the peripheral components and microglia-mediated inflammation due to hyperglycemia results in angiogenesis and vascular damage, microglial clustering around vascular lesions, fibrinogen leakage, and further up-regulation of proinflammatory mediators in the diabetic retina. Therefore, to understand the role of microglia in DR progression we depleted microglia to determine if strategies to downregulate microglia mediated inflammation serves clinically relevant to prevent neuronal damage and hence vision loss. Utilizing a genetic model using mice expressing an inducible *Cre* under the *CX3CR1* promoter and the DTR gene under the Rosa 26 promoter (CX3CR1^CreER^:R26^iDTR^), expression of DTR by *CX3CR1*- expressing cells only occurs upon tamoxifen (TAM) treatment, rendering microglia susceptible to the effects of diphtheria toxin (DTx). The overall goal of this study was to determine if the removal of reactive microglia in the diabetic retina will ameliorate vascular damage, fibrinogen deposition, and the neuronal degeneration that give rise to the robust neuroinflammation and retinal degradation in the diabetic murine retina. Our findings revealed that transient depletion of CX3CR1^CreER^:R26^iDTR^ microglia during the course of disease when microglia receive the early-stage environmental cues of retinal inflammation due to hyperglycemia, induced neuroprotective cues to upregulate Tuj1**^+^** neurons in the diabetic retina.

D44

**NOVEL C1Q-RECEPTOR INTERACTION MEDIATES CHEMOTAXIS IN NEURAL STEM CELLS**

Dana Creasman, Francisca Benavente, Mitra Hooshmand, Katja Piltti, Pooja Sakthivel, Aileen Anderson

*University of California at Irvine, Anatomy and Neurobiology, Irvine, USA*

The use of transplanted neural stem cells (NSC) to treat neuro-degenerative conditions such as spinal cord injury (SCI) holds exciting potential. However, the complex interactions between NSC and their microenvironment must be better understood to ensure the transplanted cells develop and integrate properly in the host CNS. A critical component of understanding how NSC communicate with their microenvironment is elucidating mechanisms of neuroimmune signaling. We have shown that the complement cascade, part of the innate immune system, has non-traditional roles in signaling to trans-planted NSC. Particularly, C1q, the recognition molecule of the classical cascade, signals to NSC and affects NSC migration, pro-liferation, and differentiation both *in vitro* and in an in vivo SCI model. Critically, SCI involves disruption of the blood spinal-cord barrier occurs with an acute, robust influx of serum proteins, including C1q. We have published data showing that this influx of C1q serves as chemoattractant to NSC, causing them to migrate towards the injury epicenter. Recruited NSC then add to the astroglial scar, and fail to contribute to the animal's locomotor recovery. Here, we identify a novel C1q receptor expressed by NSC, and show that it specifically mediates the chemotactic effects of C1q on NSC. Using receptor-knockout NSC, we found that receptor expression is required for C1q-induced chemotaxis, and. Investigating the molecular mechanisms and signaling pathways through which this chemo-taxis occurs, may elucidate the *in vivo* relevance of these findings and allow for the development of strategies to control NSC migration in a therapeutic context, potentially expanding the clinical SCI trans-plantation window.

D45

**DO BLOOD-BORNE FACTORS INDUCE ATYPICAL ASTROCYTES AFTER CONCUSSIVE TRAUMATIC BRAIN INJURY?**

Kijana George^1,2^, Carmen Munoz-Ballester^1^, Ben Heithoff^1,3^, Stefanie Robel^1,2,3^

*^1^ Virginia Tech Fralin Biomedical Research Institute, Glial Biology Center for Health, Disease, and Cancer, Roanoke, USA*

*^2^ Virginia Tech Fralin Biomedical Research Institute, Translational Biology, Medicine, and Health, Roanoke, USA*

*^3^ Virginia Tech, Department of Biological Sciences, Blacksburg, USA*

Increasing blood-brain barrier (BBB) leakage has been correlated with the progression of long-term neurological deficits but the underlying mechanisms that occur after BBB leakage in concussive traumatic brain injury (TBI) have not been revealed. In a mouse model of concussive TBI, we observed downregulation of nearly all astrocytic proteins, including those involved in brain homeostasis and astrogliosis as early as 10 minutes. This response is sustained for several months, presenting a major challenge for normal brain function. Notably, these areas with atypical astrocytes overlapped with areas of BBB leakage. This suggests that the leakage of blood-borne factors triggers atypical astrocytes after concussive TBI. To test the hypothesis that leakage of blood-borne factors induces atypical astrocytes after concussive TBI**,** we chose two approaches: 1) a genetic approach ablating some endothelial cells in the adult mouse brain to enable BBB leakage in the absence of mechanical injury to determine if exposure to blood-borne factors is sufficient to cause atypical astrocytes. I observed Glt1 downregulation in the cortex six hours after induced BBB damage. 2) Screening of blood-borne factors was performed using cultures from postnatal day 3-5 C57B/6 mouse pups maintained in serum-free media for seven days or 14 days. While there were no significant changes in Glt-1 and Kir4.1 expression 2h after plasma treatment, 24h plasma treatment reduced both proteins in cultures maintained in media for seven days. Also, 24h plasma treatment reduced expression of both proteins in cultures maintained in media for 14 days. To determine if the responsible blood-borne factors are proteins or immune cells, I heat-denature the plasma before treating the cultures and found that Glt-1 and Kir4.1 were rescued. Overall, my data suggest that blood-borne proteins or immune cells cause atypical astrocytes.

D46

**PROFILING ASTROCYTE AND MICROGLIA-SPECIFIC TRANSCRIPTIONAL CHANGES AFTER ISCHEMIC STROKE**

Victoria Hernandez, Kendra Lechtenberg, Marion Buckwalter

*Stanford School of Medicine, Neurology, STANFORD, USA*

Ischemic stroke is a leading cause of death and long-term disability. Neuroinflammation after stroke can significantly affect stroke outcomes–it can induce tissue repair but also exacerbate cell death. In the acute period after stroke, microglia and astrocytes enter a reactive state of gliosis. Microglia, the brain's resident immune cells, are the major producers of inflammatory molecules in the hours following stroke but also have neuroprotective functions such as clearance of harmful debris and release of anti-inflammatory factors. Similarly, astrocytes release pro-inflammatory cytokines but also mediate neuroprotection through the formation of the astrocytic scar surrounding the infarct core. However, the exact astrocytic and microglial signaling pathways regulating neuroinflammation after stroke are unresolved. A barrier to understanding this has been the challenge of parsing the astrocytic and microglial response from that of infiltrating inflammatory cell types in the brain after stroke. To address this, we used the RiboTag technique to separately obtain astrocyte and microglia-derived transcripts after stroke. By crossing the RiboTag with Aldh1l1-CreER or Cx3cr1-CreER mice, we expressed a hemagglutinin tag on ribosomes only in astrocytes or microglia, respectively. This enables immunoprecipitation and isolation of astrocytic and microglial ribosomes with their attached actively translating mRNAs. We performed RNA-sequencing on astrocyte or microglia-specific transcripts obtained from male and female mice 3 days after distal middle cerebral artery occlusion or sham surgery. In astrocytes, we found 1891 genes were upregulated and 46 genes were downregulated between stroke and sham animals. In microglia, we found 1048 genes were upregulated and 753 genes were downregulated between stroke and sham animals. Of note, many of the genes upregulated in both microglia and astrocytes at this time point are involved in immune cell recruitment and activation, suggesting microglia and astrocytes are particularly involved in attracting peripheral immune cells to the injury site at this time point. Using this technique, we will comprehensively define the astrocyte and microglia specific translatome response in the acute period after stroke.

D47

**CHOLINERGIC, DAERGIC AND GABAERGIC DEGENERATION, ALTERED BEHAVIOUR, AND INCREASED SKN-1 ACTIVITY IN C. ELEGANS MODEL OF NICKEL**

Omamuyovwi Ijomone^1,3^, Mahfuzur Miah^2,1^, Grace Akingbade^3^, Hana Bucinca^1^, Michael Aschner^1,2^

*^1^ Albert Einstein College of Medicine, Department of Molecular Pharmacology, New York, USA*

*^2^ Albert Einstein College of Medicine, Department of Neuroscience, New York, USA*

*^3^ Federal University of Technology Akure, Department of Human Anatomy, School of Health and Health Technology, Akure, Nigeria*

Nickel (Ni) is a ubiquitous metal in the environment with increasing industrial application. While environmental and occupational exposure to Ni compounds has been known to result in toxicities to several organs, including liver, kidney, lungs, skin, and gonads, neurotoxic effects have not been extensively investigated. In this present study, we investigated specific neuronal susceptibility in a *C. elegans* model of acute Ni neurotoxicity. Wild-type worms and worms expressing green fluorescent protein (GFP) in either cholinergic, dopaminergic or GABAergic neurons were treated with NiCl_2_ for 1h at the first larval (L1) stage. The median lethal dose (LD_50_) was calculated to be 5.88 mM in this paradigm. Morphology studies of GFP-expressing worms showed significantly increasing degeneration of cholinergic, dopaminergic and GABAergic neurons with increasing Ni concentration. Significant functional changes in locomotion and basal slowing response assays reflected that cholinergic and dopaminergic neuronal function, respectively, were impaired due to Ni treatment. Interestingly, a small but significant number of worms exhibited shrinker phenotype upon Ni exposure but no loopy head foraging behaviour was observed suggesting that function of D-type GABAergic neurons of C elegans may be specifically attenuated while the RME subset of GABAergic neurons are not. GFP expression due to induction of glutathione S-transferase 4 (*gst-4*), a target of Nrf2 homolog *skn-1*, was increased in a P_*gst-4*_::GFP worm highlighting Ni-induced oxidative stress. RT-qPCR verified upregulation of this expression of *gst-4* immediately after exposure. These data suggest that oxidative stress is associated with neuronal damage and altered behaviour due to developmental Ni exposure.

D48

**METABOLOMIC AND TRANSCRIPTIONAL PROFILING REVEALS BIOENERGETIC STRESS AND INFLAMMATION IN VIVO AFTER NEURONAL DELETION OF NAMPT**

Samuel Lundt^1,2^, Nannan Zhang^1^, Jun-Liszt Li^3,4^, Zhe Zhang^1,5^, Li Zhang^1,2^, Xiaowan Wang^1,5^, Ruisi Bao^2^, Feng Cai^6^, Wenzhi Sun^4,7^, Woo-Ping Ge^4^, Shinghua Ding^1,2,5^

*^1^ University of Missouri, Dalton Cardiovascular Research Center, Columbia, USA*

*^2^ University of MIssouri, Interdisciplinary Neuroscience Program, Columbia, USA*

*^3^ Peking University, Academy for Advanced Interdisciplinary Studies, Beijing, China*

*^4^ Chinese Institute for Brain Research, Chinese Institute for Brain Research, Beijing, China*

*^5^ University of Missouri, Biomedical, Biological, and Chemical Engineering, Columbia, USA*

*^6^ University of Texas Southwestern Medical Center, Children's Medical Center Research Institute, Dallas, USA*

*^7^ Capital Medical University, School of Basic Medical Research, Beijing, China*

Nicotinamide phosphoribosyltransferase (NAMPT) is the rate-limiting enzyme in the NAD^+^ salvage pathway. Our previous study demonstrated that deletion of NAMPT gene in projection neurons using Thy1-NAMPT^−/−^conditional knockout (cKO) mice causes neu-ronal degeneration, muscle atrophy, neuromuscular junction abnormalities, paralysis and eventually death. Here we conducted a combined metabolomic and transcriptional profiling study in vivo in an attempt to further investigate the mechanism of neuronal degeneration at metabolite and mRNA levels after NAMPT deletion. Here using steady-state metabolomics, we demonstrate that deletion of NAMPT causes a significant decrease of NAD^+^ metabolome and bio-energetics, a buildup of metabolic intermediates upstream of glyceraldehyde 3-phosphate dehydrogenase (GAPDH) in glycolysis, and an increase of oxidative stress. RNA-seq shows that NAMPT deletion leads to the increase of mRNA levels of enzymes in NAD metabolism, in particular PARP family of NAD^+^ consumption enzymes, as well as glycolytic genes Glut1, Hk2 and PFBFK3 before GAPDH. GO, KEGG and GSEA analyses show the activations of apoptosis, inflammation and immune responsive pathways and the inhibition of neuronal/synaptic function in the cKO mice. The current study suggests that increased oxidative stress, apoptosis and neuroin-flammation contribute to neurodegeneration and mouse death as a direct consequence of bioenergetic stress after NAMPT deletion.

D49

**JUVENILE OLIGODENDROCYTES TRANSIENTLY UTILIZE THE GLUTATHIONE ANTIOXIDANT PATHWAY**

Dylan Verden, Mikaela Neal, Wendy Macklin

*University of Colorado Denver - Anschutz Medical Campus, Cell and Developmental Biology, Aurora, USA*

Oligodendrocytes, the myelinating cells of the central nervous system, undergo massive metabolic changes during myelin synthesis - a single oligodendrocyte can produce over 500 times its soma surface area in myelin. This massive lipid and protein synthesis creates harmful byproducts like hydrogen peroxide, leading to oxi-dative stress and damage if not managed. The mechanisms by which oligodendrocytes cope with the unique metabolic demands of mye-lination remain unknown; in fact, oligodendrocytes have been shown to have low tolerance for oxidative stress, attributed to high iron concentrations and low antioxidant activity. However previous work from our lab has shown that GSTpi, a protein that catalyzes anti-oxidant reactions by glutathione, is highly expressed in juvenile, but not adult, oligodendrocytes. Notably, the juvenile period is the peak of developmental myelination in mice - one possibility is that active myelination is a period of transient antioxidant activity that offsets the metabolic stress of lipid and protein synthesis. To test this hypo-thesis, we analyzed the glutathione pathway in the oligodendrocytes of juvenile (P21-P25) and adult mice (P60-90) by qPCR and immunohistochemistry, and found that the glutathione pathway is upregulated during juvenile development. *In vitro,* we found that inducing antioxidant signaling by melatonin increased oligodendro-cyte differentiation; surprisingly, oxidative treatments (H_2_O_2_ and buthione sulfoximine) similarly increased differentiation at sublethal doses, indicating that paired oxidation and antioxidant function may be required during oligodendrocyte development. Additional time course experiments will determine when oligodendrocytes are most sensitive to oxidative and antioxidative treatments. Together, these results indicate that glutathione signaling may play an important role during oligodendrocyte development, and may underly juvenile white matter's resistance to ischemic injury.

D50

**PROTECTIVE ROLE OF SLC4A4 IN ASTROCYTE-BLOOD-BRAIN-BARRIER (BBB) INTEGRITY IN HEALTHY AND ISCHEMIC STROKE BRAIN**

Qi Ye^1,2^, Juyeon Jo^1,2^, Chih-Yen Wang^1,2^, Hyun Kyoung Lee^1,2^

*^1^ Baylor College of Medicine, Pediatric Neurology, Houston, USA*

*^2^ Jan and Dan Duncan Neurological Research Institute at Texas Children's Hospital, Pediatric Neurology, Houston, USA*

Astrocytes provide diverse support for brain function by maintaining essential interactions with endothelial cells to form the BBB. Pathological astrocyte-BBB interactions contribute to ischemic stroke, which is the 5^th^ leading cause of death in the US. However, the mechanisms underlying maintenance of BBB integrity both in health and diseases such as stroke remain poorly defined. our study focuses on an astrocyte-enriched sodium-bicarbonate cotransporter, Slc4a4, which was previously identified as an astrocyte-specific regulator of both intracellular and extracellular pH. While pH homeostasis is essential for metabolic activity in the brain, the role of Slc4a4 in astrocyte-BBB integrity remains unknown. To address the role of Slc4a4 in this context, we generated new transgenic mouse lines that temporally ablate Slc4a4 in astrocytes. Using this genetic mouse model, we show loss of Slc4a4 in adult significantly dampens astrocyte endfeet Ca^2+^ signaling and generates enlarged blood vessels with disrupted endothelial junctions. Combination of transcriptome, proteomic and metabolomic profiling of Slc4a4-ablated astrocytes and conditioned media (CM) of Slc4a4-ablated astrocytes reveal dysregulation of genes associated with vasculature-BBB maintenance, inflammatory chemo-cytokines, and glial metabolism, further supporting a crucial role for Slc4a4 in BBB integrity by governing astrocyte-endothelia cell crosstalk. Among differentially expressed genes associated with Slc4a4 deletion, we confirm the upregulation of Ccl2 (C-C Motif Chemokine Ligand 2) in the CM of Slc4a4-ablated astrocytes and Slc4a4-deficient brain. We further validate the Slc4a4-Ccl2 axis using *in vitro* BBB model and find that blocking Ccl2 pathway restores endothelial junction and permeability. Using a mouse model of stroke, we found loss of Slc4a4 exacerbates stroke-induced motor dysfunction, mortality and BBB disruption coupled with impaired reactive gliosis. Together, our study indicates the indispensable role of Slc4a4 mediated astrocyte-BBB interaction, providing insights for the potential of glia metabolism regulators as novel therapeutic approach for BBB-related CNS disorders.

D51

**SERUM SOLUBLE EPOXIDE HYDROLASE DERIVED OXYLIPINS AND SMALL VESSEL STROKE**

Di Yu^1,2^, Ameer Taha^3^, Theresa Pedersen^3^, Joel Ramirez^2^, Maged Goubran^2^, Miracle Ozzoude^2^, Fuqiang Gao^2^, Leanne Casaubon^4^, Robert Bartha^5^, Sean Symons^2^, Donna Kwan^6^, Brian Tan^7^, Krista Lanctot^1,2^, Mario Masellis^1,2^, Sandra Black^1,2^, Rick Swartz^1,2^, Walter Swardfager^1,2^

*^1^ University of Toronto, Pharmacology and Toxicology, Toronto, Canada*

*^2^ Sunnybrook Research Intitute, Evaluative Clinical Science, Toronto, Canada*

*^3^ University of California, Davis, Food Science and Technology, Davis, US*

*^4^ University Health Network, Krembil Research Institute, Toronto, Canada*

*^5^ Western University, Department of Medical Biophysics, London, Canada*

*^6^ Queen's Unversity, Center for Neuroscience Studies, Kingston, Canada*

*^7^ Baycrest Health Sciences Center, Rotman Research Institute, Toronto, Canada*

Soluble epoxide hydrolase (sEH) inactivates vasoactive p450 derived polyunsaturated fatty acid epoxides, converting them into cytotoxic diols. We found that the ratios of linoleic acid (LA) diols to epoxides were associated with subcortical ischemic vascular disease (SVD) in patients with transient ischemic attack; however, the roles of these oxylipins remain unclear in clinical stroke of large or small vessel lacunar aetiology. Patients from ONDRI with clinical stroke of either large or small vessel etiology were included. Four plasma LA oxylipins (12,13-dihydroxyoctadecamonoenoic acid (12,13- DiHOME), 12,13-epoxyoctadecenoic acid (12,13-EpOME), 9,10-DiHOME, 9,10-EpOME) were measured with UPLC/MS-MS. White matter hyperintensities (WMH; an SVD marker) were quantified from structural MRI using Lesion Explorer. Cerebral free water (resultant from vasogenic edema or neurodegeneration) was calculated from diffusion tensor imaging. Among 80 stroke patients (n=50 large vessel occlusion, n=30 lacunar infarcts), the ratio of 12,13-DiHOME to its epoxide sEH substrate 12,13-EpOME was higher among patients with lacunar stroke (F_1,78_=5.06, p=0.028).

Controlling for age, sex, hyperlipidemia, waist hip ratio, and fasting glucose, the 12,13 oxylipin ratio, and the 9,10 oxylipin ratio were positively associated with volumes of deep (β=0.335, p=0.005; β=0.288, p=0.012, respectively) and periventricular (β=0.331, p=0.002; β=0.404, p<0.001, respectively) WMH. In the lacunar stroke subgroup, the 12,13-ratio was associated with white matter free water volume (β=0.339, p=0.027). Oxylipins derived from sEH activity may contribute to clinical stroke of small vessel etiology, potentially via blood brain barrier disruption and vasogenic edema.

D52

**VULNERABILITY OF WHITE MATTER FUNCTION IN ALZHEIMER'S DISEASE**

Sarah Zerimech^1^, Joseph F. Quinn^2^, Selva Baltan^1^

*^1^ OHSU, Anesthesiology and Peri-Operative Medicine, Portland, USA*

*^2^ OHSU, Department of Neurology, Portland, USA*

Alzheimer's disease (AD) is a global health issue that affects disproportionally females. AD is a neurodegenerative disorder of gray matter, characterized by synaptic loss, neuronal death, and the presence of neurofibrillary tangles. White matter (WM) lesions observed in elderly subjects increase the vulnerability of WM to injury, such as ischemic stroke. Increasing evidence has revealed a connection between WM changes (WMCs) and the pathophysiology of AD. Interestingly, human patients and studies from animal models of AD suggest that WM dysfunction precedes AD pathology. However, direct assessments of WM function in AD have not been documented. Because AD generally associates with aging brain, it is important to distinguish age-related changes from neurodegenerative pathophysiology. Using Tg2576, a well-established AD mouse model that overexpressed human APP, we investigated axon function properties and the response to metabolic challenges with respect to age.

Isolated mouse optic nerves (MONs), a pure myelinated WM tract, were obtained from female Tg2576^+^ mice and age-match littermate controls at 7 and 18 months old. Axon function was quantified as area under the evoked compound action potentials. Ischemia was induced by switching to oxygen-glucose deprivation (OGD) for 1h, followed by five hours recovery. Under baseline conditions, axon conduction properties and excitability remained the same among the different groups. Young Tg2576^+^ females showed a similar recovery to control female group after OGD. There was a robust effect of aging among control females such that aging group recovered substantially less compared to young control group. However, unexpectedly, aging Tg2576^+^ females recovered similar to young Tg2576^+^ group. Correspondingly, aging Tg2576^+^ females recovered better than aging control females suggesting that Tg2576^+^ aged females do not have impaired axon function.

In conclusion, unexpectedly, Tg2576 aging females were less affected by ischemic injury compared to aged control females. A potential explanation for this resilience against ischemic injury could be alternative cleavage of APP or compensatory mechanisms in WM that might need further investigation.

## Author Index

Abdullaev, I. C09

Abisambra, J. A28

Abrams, C. D23

Abushalbaq, O. OR-03-03, A02

Acharya, M. A14

Ackerman, S. C21

Adams, A. A26

Adams, D. S-15-01, C04

Adams, K. OR-03-02, A34

Adebiyi, O. A33

Afrasiabi, M. D26

Agnew, W. B35

Agnew-Svoboda, W. S-03-04

Aguayo, L. B52

Ai, S. C13

Ain-uddin, W. A10

Akingbade, G. D47

Al-Hasani, R. A50, A51

Al-Sammarraie, N. A03, A27

Aldoghmi, M. B40

Alikhani, L. A14

Allan, K. S-15-01, A04

Allimuthu, D. S-15-01, C04

Alluis, A. A35

Almad, A. C-05-03

Aluko, O. A31

Alzidaneen, M. A37, C07

Ambeskovic, I. C10

Ammassari-Teule, M. A31

Amorim, A. C48

Anand-Apte, B. C42

Anderson, A. B20, D44

Anderson, T. A19

Andhey, P. OR-01-01, C51

Anita, N. A46

Antel, J. OR-04-04, C08

Antikainen, H. A22

Ao, J. C23

Archambault, A. B37

Armstrong, R. S-11-01

Artyomov, M. OR-01-01, C51

Aschner, M. D47

Ashpole, N. C27

Ates, G. A15

Atkins, C. S-14-02, D42

Atkinson, K. A35

Audet, M. S-04-03

Bach, M. C09

Bachstetter, A. A28

Baddour, A. A14

Baggeroer, C. A06

Bagheri, H. C23

Bai, Q. D19

Balderrama, A. B13, C28

Ballard, V. A24

Baltan, S. C26, D52

Bandla, S. B37

Banerjee, P. OR-03-02, A34

Banerjee, S. A32, D25

Banerji, T. A48

Banik, N. A12, B44, D22

Bao, R. D48

Bar-Or, A. A36

Barclay, K. A51

Bareil, C. D15

Barger, S. A18

Barnes, A. C55

Barres, B. OR-01-03, D09

Bartels, C. A04

Bartha, R. D51

Batista, C. B09

Batoki, J. C42

Baulch, J. A14

Bazick, H. C32, D01

Beaver, M. D12

Bechler, M. C23

Beckman, D. A17

Bederman, I. S-15-01, A04, C04

Bejarano-Pérez, E. B05

Bell, L. A23

Bellen, H. B19

Benavente, F. D44

Benbarka, T. A35

Benitez, B. OR-01-01, C51

Benjamins, J. A36

Benmamar-Badel, A. C23

Bentea, E. A15

Berdyshev, E. B27

Bergdolt, L. D12

Bergersen, K. S-03-04

Bergles, D. C-05-02

Berkes, D. B02, B26

Berti, C. D32

Bessard, A. D10

Bettelli, E. C-06-04

Bhalla, A. B14

Bibus, D. B27

Bieberich, E. S-13-04, B02, B41, C54, C56

Bilas, A. B40

Binder, D. B40, C29

Bindu C44

Bisagno, V. S-06-03

Bisht, K. OR-04-03, B36

Black, S. OR-02-04, C52, D18, D51

Blader, I. A24

Blagg, B. D23

Blommer, J. A19

Bodden, C. B38

Bodnar, C. A28

Boecker, A. C-05-03

Boger, H. B44

Boison, D. A14

Boisvert, M. OR-04-05, A16

Bonkowsky, J. C-02-02

Boone, Z. A24

Borchelt, D. C-02-03

Bose, R. A32

Boullerne, A. D41

Boyer, K. OR-01-01, C51

Božić, M. D14

Bracher, F. S-15-01

Brahms, E. OR-01-03, D09

Bredikhin, M. D22

Briana, F. C-02-02

Bucinca, H. D47

Buckwalter, M. OR-01-03, D09, D46

Bugiani, M. OR-03-02, A34

Bukau, B. S-01-03

Bunner, K. S-10-03

Burton, E. D19

Bynoe, M. A33

Cabitta, L. OR-01-04, C02

Cadet, J. S-06-01, S-06-04

Cai, F. D48

Cain, M. A29

Calabrese, B. D21

Calabresi, P. C-05-02

Call, C. C-05-02

Capone, M. A12, D22

Carbajal, K. C-06-03

Cardini, J. B30, D16

Cardona, A. D43

Cardona, S. D43

Carey, L. S-10-03

Carmichael, S. OR-02-02, C46

Carrillo, G. A24, B24

Carruthers, N. A36

Carvalho Alcantara Gomes, F. B09

Casaubon, L. D51

Castillo-Montesinos, M. D39

Catague, R. A06

Cazalla, D. A25

Chakrabarty, P. A17

Chan, P. OR-02-04, D18

Chang, M. B13

Chang, N. D42

Chapelet, G. C49

Chaurand, P. C23

Chemuru, S. B42

Chen, A. S-14-01

Chen, J. S-08-03

Chen, S. B13, C28

Chen, Z. B13

Cheng, Y. S-03-01

Chiou, B. S-07-01

Choi, J. B19

Choudhary, M. A10

Christian, I. C11

Chu, J. S-08-03

Church, K. D43

Cianciolo, L. D25

Cibelli, A. A44

Cicardi, M. A20

Cid, L. B05

Cirrito, J. A50, C17

Clayton, B. A04

Codagnone, M. D31

Codron, P. D15

Cohn, E. A04

Combs, C. C50

Connor, J. S-07-01, D08

Conway, S. A50

Cooper, E. C23

Cordeau, P. D15

Correale, J. S-07-03, B18, C31

Cougnaud, L. C23

Coulombe, V. B07

Coutinho Costa, V. B09

Covey, D. S-15-02

Cox, A. D22

Creasman, D. D44

Cristobal, C. B19

Crivelli, S. B02, B26, B41, C54, C56

Crockett, D. A09

Crystal, J. S-10-03

Cubello, J. B10

Cui, D. C-01-04

Cui, J. B13, C28

D'Aprile, C. OR-01-04, C02

D'Mello, V. B14

Damas, M. B26

Daniels, B. S-14-02, D42

Dao, A. A17

Dasgupta, S. B15

Dave, S. B38

De Biase, L. C-04-01, B12

De Stefano, A. D03

De Vries, H. B02

DeBell, M. A48

Dehairs, J. B26

Del Bel, E. OR-04-01, B34

Del moral, D. B03

Delucia, J. D26

Demcheko, A. A07

Dempsey, R. PS-B-45

Den Hoedt, S. B02

Denton, T. B04

DePalma, R. C28

DePaula-Silva, A. A25

Derkinderen, P. S-16-02, C49

Derua, R. B26

Deschiffart, A. C-02-02

Desfor, S. B20

DeSilva, T. C42

Deslauriers, J. OR-04-03, B36

Dezonne, R. B09

Dhami, K. A07

Di Pietro, A. B18

Diaz, V. A45

DiBona, V. A09

Ding, S. A52, D20, D48

Ding, X. B19

Dinkins, M. B41

Dion-Albert, L. S-04-01, C43

Dobrowolski, R. A22, D02

Dobrowsky, R. D23

Dodson, V. B14

Dohare, P. S-08-04, A01

Dominova, I. A38

Doney, E. S-04-01

Donis-Cox, K. A17

Dos-Santos-Pereira, M. OR-04-01, B34

Dougherty, J. OR-01-02, B23

Doyle, T. S-10-04

Drasites, K. D22

Drew, K. A13, B27

Dreyfus, C. A53, D03

Duchalais, E. D10

Dudek, K. S-04-01, C43

Dunaevsky, A. D12

Dunbar, G. A32

Dunn, C. C48

Dupree, J. S-05-04, B11, B46

Durieu, E. C49, D10

Dustin, E. B11

Dutta, K. D15

D'Aprile, C. B17

Edwards, H. C17

Edwards, J. C52

Eells, J. A45

Eisenberg, C. D26

Ekiz, A. S-03-01

Elitt, M. S-15-01

Ellis, J. A45

Elsherbini, A. B41, C54, C56

Eltzschig, H. S-16-03

Erdmann-Gilmore, P. A51

Erika, S. C-02-02

Escalante, M. S-05-04

Espinoza, K. B12

Espírito-Santo Araújo, S. B09

Estevez, R. S-08-01

Ethell, I. S-09-04

Everson, E. B04

Evonuk, K. C42

Exley, C. B02

Factor, D. S-15-01

Fantini, D. D07

Farmer, W. OR-03-01, B48

Farrand, A. B44

Fasciglione, K. B39

Fatemi, A. C06

Fedorov, Y. S-15-01

Feinstein, D. C-03-01, D41

Feltri, M. D32

Ferguson, S. D32

Feri, M. A35, B20

Ferland, R. S-08-04, A01

Fernandez Fernandez, R. A45

Fernandez, E. B52

Ferreira, S. PRES-04

Fiacco, T. S-03-04, B35, B40

Figueroa, Z. B35

Fil, D. B21

Flint, D. C-01-01

Floden, A. C50

Flores-Muñoz, M. B03

Flounlacker, K. C01

Fogerty, J. D25

Folts, C. C10

Forbes, T. B46

Foreman, O. S-15-04

Fox, M. S-09-01, OR-03-05, A24, B24, C11, C12, C34, D29

Frame, G. OR-02-01, D40

Frangella, N. B38

Franklin, A. C32, D01

Frare, C. A13, C24

Frick, L. D32

Friedman, B. S-15-04

Friedman, H. C23

Friedman, W. A05, B15

Friedrich, R. C04

Frydman, J. S-01-04

Fuchigami, T. C18

Fuenzalida, M. B52

Fujinami, R. A25

Furusho, M. B01

Fuss, B. S-05-04, B09

Gallo, V. OR-03-02, A34, B46

Gao, F. C04, D51

Gao, X. C38

Garber, C. B49

Garcia, A. S-09-02

Garcia, L. C-05-03

García-López, A. B08

Gardiner, W. C17

Garner, D. D22

Garrison, E. S-15-01, D07

Gbadamosi, I. B16

Ge, W. D48

Geiger, J. B43

George, K. D45

Gereau, G. A50

Gevorgyan, A. A04

Giera, M. S-15-01

Giovagnoni, C. B02, B26

Giussani, P. OR-01-04, C02

Glatigny, S. C-06-04

Glausen, T. A24

Godoy, M. S-14-01

Goetz, L. B07

Goins, L. B28

Goldstein, E. B46

González Ibáñez, F. OR-04-03, B36

Gorbea, C. A25

Goubran, M. D51

Grant, D. C28

Grassi, S. OR-01-04, B17, C02

Gravel, C. D15

Green, K. S-03-01

Greig, N. C-04-01

Grinspan, J. C-02-01

Gross, M. B42

Grouza, V. C23

Grover, S. B42

Grubisic, V. S-16-03

Gu, Z. B13, C28

Guang, S. C06

Guerrier, C. C23

Guillem Del Angel, A. A47

Guimarães, F. OR-04-01, B34

Guitart, M. S-07-03

Gulbransen, B. S-16-03

Gunasekaran, S. OR-04-02, A39

Ha, R. OR-03-05, C34

Hadwen, A. C23

Hahn, Y. C01

Halcrow, P. B43

Halpain, S. D21

Han, X. B11

Haque Chowdhury, H. C-03-03

Haque, A. A12, B44, D22

Hartley, M. A48

Hauser, K. C01

Hayakawa, K. S-14-04

Haydar, T. B30, D16

He, Z. D24

Healy, L. OR-04-04, C08

Heffernan, C. D02

Hegert, J. C11

Heifets, B. S-12-02

Heil, L. C19

Heim, C. C16

Heithoff, B. D45

Hendrix, R. C17

Hermann, B. A45

Hernandez, R. S-09-03, D27

Hernandez, V. D46

Hernández, M. C41

Hernández-Kelly, L. B05, C41, D39

Herrmann, N. OR-02-04, C52, D18

Herzog, E. A40

Hewett, S. A06, C24, D30

Higgs, H. D21

Hill, J. C13

Hilton, T. B04

Hinkson, C. A24

Hinman, J. OR-02-02, C46

Ho, M. C-01-03

Hobert, O. PRES-01

Hodes, G. S-04-02

Hoedt, S. B26

Hoffman, T. C28

Hogestyn, J. C47

Hohmann, A. S-10-03

Holt, L. S-09-03, A41, D27

Holt, M. C-03-02

Holtzman, D. OR-01-01, C51

Holzbaur, E. C-05-03

Hooshmand, M. D44

Hope, K. C-04-01

Horn, C. C16

Horska, A. C55

Horvat, A. C-03-03

Hossain, A. A12, B44

Hsieh, H. C-05-02

Hu, B. C03, C53

Hu, L. A04

Huang, Y. S-08-04, A01, D03

Hubbard, E. C19

Hubler, Z. S-15-01, C04

Hubo, P. C06

Hung, S. A04

Ibrahim Abdullahi, I. C14

Iftikhar, K. D28

Ijomone, O. A31, B47, D47

Inoue, K. OR-01-03, D09

Ising, C. OR-01-01, C51

Itokazu, Y. C18

Iyer, V. S-10-03

Jackson, S. A45

Janczur Velloso, F. C15

Jani, H. B39

Jansen, W. A17

Jarjour, A. C23

Jarweh, C. C23

Jayanthi, S. S-06-02, S-06-04

Jenrow, K. A32

Jin, J. S-15-01

Jo, J. OR-02-03, B19, D50

Johnson, C. C28

Jonak, C. C29

Jones, A. B42

Jones, S. D21

Jordan-Sciutto, K. C-02-01

Jose, J. B38

Joshi, S. A05

Juan-Sing, C. C29

Julian, R. C19

Julien, J. D15

Jung, S. B19

Juárez-Portilla, C. B03

Kacerovsky, J. OR-02-01, D40

Kafle, S. A07

Kak, G. C16

Kakoty, V. C20, C45

Kanack, A. S-15-03

Kapogiannis, D. S-13-02, A19, C48

Kapoor, R. C44

Karki, S. B41

Karl, M. S-15-01, D07

Katritch, I. A40

Kaufman, M. C27

Kaur, S. D23

Kawabe, H. B19

Keen, D. D13

Kelly, J. S-01-01

Kelly-Castro, E. A09

Kerr, A. C11

Khadra, A. C23

Khakh, B. A21

Khan, I. D02

Khan, N. B28

Khandker, L. B31

Khanna, R. S-10-02, C-07-01

Kielian, T. S-03-02, C16, C40, D12

Kim, H. S-11-03, A22, A26, D02

Kim, J. A26

Kim, S. C-02-01, A45

Kirkemo, L. A48

Kirov, A. B41

Klein, J. B30, D16

Klein, R. A29, B49, C13

Klosek, M. C55

Knapp, P. S-05-03, C01

Knaryan, V. B44

Komuro, Y. OR-02-02, C46

Konan, L. C28

Kordower, J. A17

Korshoj, L. C40

Kosman, D. S-07-02

Krattli, R. A14

Kreft, M. C-03-03

Krishnagiri, M. D42

Kriz, J. D15

Krizman, E. A47

Kruining, D. B26

Kumari, E. C15

Kuo, Y. C09

Kurzion, R. D16

Khushboo, A30

Kwan, A. S-12-03

Kwan, D. D51

Kyauk, R. S-15-04

LaConte, L. C11

Lai, Y. S-10-03

Lanctot, K. D51

Lanctôt, K. OR-02-04, C52, D18

Lane, T. S-03-01, C-06-02

Lawson, S. OR-01-01, C51

Leary, P. D19

Lebel, M. S-04-01, C43

Lecca, D. C-04-01

Lechtenberg, K. D46

Ledesma, M. B02

Lee, H. OR-02-03, B19, D50

Lee, W. S-10-03

Leitzel, O. D34

Leone, G. D22

Levison, S. S-11-04, B14, B32, C15

Li, D. C-01-04, A42

Li, J. S-14-01, C03, C53, D48

Li, Q. C06

Li, R. B13, C28

Li, T. C-01-04

Li, Y. B39

Li, Z. B30, D16

Liddelow, S. OR-01-03, D07, D09

Lindeke-Myers, A. B19

Lindman, M. S-14-02

Lingelbach, M. D21

Lisak, R. A36

Litvak, E. D31

Liu, Y. C-01-04

Logan, M. OR-04-05, A16

Lopez, A. B37

Lopez, M. PS-B-45

Losen, M. B02

Lu, X. C36

Lundt, S. D48

Luo, Q. B02, B26

Luo, X. D25

Lutz, S. C-06-01, D13

López-Franco, Ó. B03

Mabandla, M. A43

MacCoss, M. C19

MacDonald, J. C33

Machnicki, M. OR-02-02, C46

Macklin, W. PRES-02, D49

Madhavan, M. S-15-01, A04

Madlala, T. A43

Magistretti, P. C-03-04

Mahdi, F. A49, C27

Mahmutovic, D. D34

Maienschein-Cline, M. D41

Major-Orfao, C. A46

Malenka, R. S-12-01

Malykhina, A. A08

Mangale, V. S-03-01

Manis, M. OR-01-01, C51

Manivasagam, S. C13

Manor, U. D21

Marion, C. OR-01-05, C25

Martinez, P. B02

Martinez-Lozada, Z. OR-03-01, B48

Martinez-Martinez, P. S-13-03

Martino Adami, P. S-07-03

Masellis, M. OR-02-04, D18, D51

Massie, A. A15

Masukawa, L. C-04-01

Mather, M. B31

Mattera, V. C31

Matzelle, D. A12, B44, D22

Maurel, P. D02

Mauri, L. OR-01-04, B17, C02

Mayer-Proschel, M. B10, C35, C47

McBrearty, K. C17

McCarthy, M. S-05-01

McClintock, T. B28, C56

McCoy, M. S-06-04

McGavern, D. S-03-03

McKee, C. OR-04-03, B36

McQuiston, R. B11

McTigue, D. OR-01-05, C25, D04

Mealy, A. C32, D01

Meares, G. OR-02-05, D11

Mehndiratta, K. A09

Meissner, W. A19

Mellan, A. D30

Menard, C. S-04-01, C43

Mendez, L. B05

Mendoza-Calles, C. B03

Mennerick, S. C36

Merrihew, G. C19

Messmer, M. B38

Mey, G. C42

Miah, M. D47

Michel, P. OR-04-01, B34

Mielke, M. B02

Mikati, M. A51

Mikes, M. B27

Miller, A. B39

Miller, M. A29

Miller, R. S-15-01, C23, D07

Miller, S. D07

Miranda, S. C-02-02

Mobashery, S. B13

Moca, E. C-04-01

Modrusan, Z. S-15-04

Mogha, A. C21

Mohammed, S. A49

Mohila, C. B19

Moiseev, D. C03

Mokgokong, M. A43

Molina-Castro, G. C-05-02

Mollie Capone, M. B44

Monavarfeshani, A. D24

Mongin, A. S-08-04, A01, C09

Monk, K. C21

Monnerie, H. C-02-01

Montana, V. C-01-01

Montine, T. C19

Montroull, L. B15

Moore, L. B32

Morelli, L. S-07-03

Morganti, J. A28

Morrison, J. A17

Morton, A. A04

Mukherjee, K. C11

Mukhtar, I. A10

Mulder, M. B02, B26

Muller, S. A17

Munch, A. OR-01-03, D09

Munoz-Ballester, C. D45

Murai, K. OR-02-01, OR-03-01, B48, D40

Murphy, E. S-02-01

Musa Omoyine, I. C14

Mustapic, M. A19

Myatich, A. D22

Myo, Y. S-05-04

Nabekura, J. C-04-02

Naicker, T. B47

Nair, M. C32, D01

Nalwalk, J. S-08-04, A01

Namba, C. D32

Naveilhan, P. S-16-02

Neal, M. D49

Nedelkoska, L. A36

Neely, E. S-07-01, D08

Nemeth, C. C06

Neunlist, M. S-16-02, C49, D10

Neutzling Kaufmann, F. S-04-01, C43

Nevin, Z. S-15-01

Newman, K. S-14-02

Ngoupaye Temkou, G. A43

Ngu, H. S-15-04

Nguyen, H. C26

Nguyen, M. A46

Niaz, M. D28

Nichols, M. S-13-01, A07, B42

Nikolova-Karakashian, M. C56

Nisar, U. A10

Nishiyama, A. C23

Noble, M. C10

Nogueras-Ortiz, C. C48

Nookala, S. C50

Noori, S. A35

Nori, Y. B39

Notterpek, L. C-02-03, C32, D01

O'Brien, B. C06

O'Connell, R. S-03-01

Obenaus, A. S-11-02

Ocharan, A. C22

Ochoa-Raya, A. D13

Odufuwa, A. A37, C07

Olajide, O. B16

Olivarria, G. C-06-02

Oliver, A. B50

Olp, M. S-15-03

Olsen, M. S-09-03, A41, D27

Olson, D. S-12-04

Omkumar, R. OR-04-02, A39

Omotoso, G. B16

Oprea, L. C23

Orbeta, S. S-08-04, A01, C09

Ortega, A. S-05-04, B05, B06, B08, B22, C05, C22, C41, D39

Orthmann-Murphy, J. C-05-02

Osei-Owusu, J. S-08-03

Ostrowski, M. C31

Osunde, M. A35

Otazu Aldana, G. B39

Ott, S. A17

Ou, Y. A18

Ouk, M. C52

Oulier, T. D10

Oullier, T. C49

Owens, T. C23

Ozzoude, M. D51

Pacheco, C. A18

Pacheco, N. D27

Paes de Carvalho, R. B09

Paez, P. S-07-04

Pajoohesh-ganji, A. C23

Palavicini, J. S-02-04, B11

Palmisano, M. D32

Pan, P. A53

Pandey, H. C44

Pang, Y. C10

Parent, A. B07

Parent, M. B07, B51

Paris, J. A49, C27

Parpura, V. C-01-01

Pasquini, J. S-07-03, B18, C31, D31

Pasquini, L. B18

Patel, A. D02

Patel, P. C11, D07

Patil, A. A09

Patyal, P. B21

Paul, F. D32

Peachey, N. C42

Pedersen, T. A46, D51

Pellegatta, M. D32

Pereyra Gerber, P. C31

Perkins, B. D25

Pertsovskaya, V. B46

Peterson, A. C23

Peterson, T. OR-01-03, D09

Petratos, S. C-07-03

Petrisko, T. B50

Pettersen, J. OR-02-04, D18

Pfister, B. A26

Pham, T. B42

Phaneuf, D. D15

Philippe, É. B07

Phillips, B. OR-02-01, D40

Piao, X. C21

Picard, K. OR-04-03, B36

Pignataro, A. A31

Piltti, K. D44

Piscitelli, E. B38

Pitcher, T. A19

Pitt, S. C24

Planas-Fontánez, T. D03

Pleshinger, M. C04

Plourde, K. D15

Podar, I. A09

Poitelon, Y. D32

Polcyn, R. A12

Popko, B. D07

Pozzi, S. D15

Pradhan, A. D16

Prigent, A. C49

Prinetti, A. OR-01-04, B17, C02

Prioni, S. OR-01-04, B17, C02

Proschel, C. C47

Pryce, R. C23

Pukos, N. D04

Qin, H. B41, C54, C56

Qin, W. OR-01-01, C51

Qiu, Z. S-08-03

Qrareya, A. C27

Quadri, Z. C56

Quinn, J. D52

R. Garcia, A. D29

Raas, Q. C-02-02

Rabin, J. C52

Radecki, D. OR-03-04, D06

Rahimian, R. D15

Rahman, M. C12

Raisman-Vozari, R. OR-04-01, B34

Ramirez, J. OR-02-04, D18, D51

Rampam, S. B30, D16

Rangel-Barajas, C. S-10-03

Ranspach, L. D25

Rao, M. S-16-01

Ray, S. A03, A27

Reandelar, M. B39

Rebec, G. S-10-03

Reduron, L. B51

Reichelt, M. S-15-04

Reinés, A. D31

Renaud, L. D15

Ribeiro, M. C33

Riccomagno, M. S-03-04, B35

Rice, S. A13, B27

Richa, Y. OR-02-01, D40

Riffo, N. B52

Rincon, W. A35

Rivera-Leon, A. C04

Rizvi, A. A53

Robel, S. D34, D45

Robinson, A. D07

Robinson, M. OR-03-01, A47, B48

Rodrigues, A. B09

Rodriguez, A. B06, B22

Rodriguez, D. D43

Roldán-Roldán, G. B03

Rolli-Derkinderen, M. S-16-02, C49, D10

Romer, M. C-02-01

Rosato Siri, V. S-07-03

Rose, I. D07

Rosenberg, P. S-05-04

Rossignol, J. A32

Roumenova, V. B39

Roush, E. C54, C56

Rudd, M. OR-02-02, C46

Rudko, D. C23

Saban, D. C-04-03

Sabbagh, U. OR-03-05, C34

Saberi, S. S-10-03

Sah, R. S-08-04, A01

Sahlas, D. OR-02-04, D18

Sahu, B. C50

Saikali, S. B07

Sakthivel, P. D44

Salmon, C. OR-02-01, D40

Salois, G. C35, C47

Salvemini, D. S-10-01, S-10-04

Samanta, J. OR-03-04, D06

Samantaray, S. B44

San Martin, O. B52

Sanchez, F. B06

Sanes, J. D24

Santhakumar, V. D26

Sapkota, D. OR-01-02, B23

Sarathlal, K. C. C20, C45

Sas, A. C-06-03

Sase, S. C-05-03

Sathi, S. D03

Sato, H. A15

Sax, J. S-15-01, C04

Scaglione, J. S-15-03

Scaglione, M. S-15-03

Scanlan, T. A48

Scavuzzo, M. A04

Schaler, A. B12

Scheithauer, A. B02

Scherer, S. C-05-03

Schick, E. S-15-01

Schmidt, R. OR-01-01, C51

Schober, A. OR-02-01, D40

Schoppik, D. D19

Schousboe, A. C-01-01

Scimemi, A. S-08-04

Sears, S. D30

Segal, B. C-06-03

Sekyi, M. A35

Selbach, M. D32

Serrano, J. OR-01-01, C51

Seth, P. C44

Shah, K. A09

Shaheen, F. A10

Shahid, M. A10

Shalgi, R. S-01-02

Shallie, P. B47

Shams, R. D22

Shandra, O. D34

Sharma, K. OR-04-03, B36

Shen, D. B04

Shen, K. S-15-04

Shen, Y. S-15-04

Sheng, M. S-15-04

Sherafat, A. C23

Shi, Y. OR-01-01, C51

Shields, D. A12, B44

Shiflett, M. D26

Shih, A. D21

Shimoni, S. D16

Shin, W. B39

Shlossman, M. B24

Shokat, M. A48

Shrestha, G. A07

Shuai, X. B20, C29

Siddiqi, K. OR-02-01, D40

Siedhoff, H. B13, C28

Silva, J. C05

Siminovitch, K. C23

Simjee, S. C39, D28

Sims, S. OR-02-05, D11

Singer, T. A52

Singh, R. C42

Sinobas Pereira, C. B42

Sitoy, H. C-04-01

Skar, G. D12

Skias, D. D41

Skinner, D. S-03-01, C-06-02

Smith, B. S-15-03

Snipes, L. OR-01-01, C51

Somaiya, R. OR-03-05, C34, D29

Song, H. C28

Song, W. B13

Sotiropoulus, I. C48

Soto Verdugo, J. D39

Soucy, G. D15

Soung, A. B49, C13

Spassieva, S. B28, B41, C54, C56

Spilling, C. A07

Spray, D. A44

Sprung, R. A51

Sriram, S. A35

Srivastava, S. C11

Stemmer, P. A36

Stenovec, M. D14

Stevens, J. B02

Stiles, L. S-14-01

Stipursky, J. B09

Stout, R. A44, B38, B39

Studtmann, C. C37

Stys, P. C53

Su, J. C12

Subramaniam, M. B14

Subramanian, D. D26

Sun, D. S-08-02

Sun, G. S-02-02

Sun, W. D48

Sunday Abraham, M. C14

Sunday Blessing, O. C14

Sung, J. A18

Sutton, S. D30

Suárez-Pozos, E. S-05-04

Svitkina, T. D21

Swanger, S. C37

Swardfager, W. OR-02-04, A46, C52, D18, D51

Swartz, R. OR-02-04, D18, D51

Swinnen, J. B26

Swire, M. C23

Syage, A. S-03-01, C-06-02

Syed, T. OR-02-01, D40

Symons, S. D51

Sánchez-Palestino, L. D39

Taha, A. S-02-03, OR-02-04, A46, D18, D51

Takech, M. B07

Taliyan, R. C20, C45

Tammy, S. C-02-02

Tan, B. D51

Tanaka, H. A53

Tannis, D. A44

Teamer, J. A24

Tenner, A. B50

Teo, W. C53

Terasaki, M. B01

Tesar, P. S-15-01, C-05-01, A04

Thammisetty, S. D15

Thang, L. A50

Thomason, E. S-05-04

Thompson, D. A37, C07

Thompson, M. S-15-01

Thummel, R. D25

Tian, F. D24

Tibuleac, T. OR-02-01, D40

Titus, H. D07

Tiwari-Woodruff, S. A35, B20, C29

Tooth, R. OR-02-01, D40

Torres, B. S-09-03, A41

Torres, O. S-06-04

Touil, H. A36

Tovar-Ramírez, J. D39

Townsend, R. A51

Traetta, M. D31

Trager, N. A12

Tran, T. OR-03-03, A02, D26

Tremblay, M. S-05-02, OR-04-03, B36

Trevino, T. D13

Tripathi, P. B41, C54, C56

Tuck, E. S-04-01

Tweedie, D. C-04-01

Uccelli, N. D31

Udo-Bellner, L. B39

Ulrich, J. OR-01-01, C51

Umukoro, S. A31

ur-Rahman, A. A10

Usman Simjee, S. A10

Van den Hove, D. B02

Vanderver, A. C-05-03

Vanegas, D. D43

Vardjan, N. C-03-03

Vecchiarelli, H. OR-04-03, B36

Vega, L. C41

Vemuganti, R. PS-B-45

Verbruggen, L. A15

Verden, D. D49

Verhaagen, J. B02

Vernoux, N. OR-04-03, B36

Vertegel, A. D22

Vizcarra, E. S-03-04

Voglewede, M. A09

Vollmer, L. B49

Vries, H. B26

Vuckovic, D. C23

Waagepetersen, H. C-01-01

Wade, Q. S-07-01, D08

Wadhwa, A. C15

Waelkens, E. B26

Walch, E. B40

Wallis, G. A23

Walter, J. B02, B26

Wang, C. OR-02-03, B19, D24, D50

Wang, G. B41, C54, C55

Wang, J. B04

Wang, K. OR-01-01, C51

Wang, P. C-01-04

Wang, X. D48

Wang, Y. C-01-04

Wanner, I. S-14-01

Watt, J. A37, C07

Weaver, M. D32

Wei, J. OR-03-05, C34

Wei, P. OR-01-05, C25

Weigel, M. OR-01-03, D09

Weihe, C. B50

Weimer, J. C-07-02

Weston, K. C38

White, T. C28

Whiteson, K. B50

Wies Mancini, V. B18

Wight, P. B21

Wilcox, K. A23, A25

Williams, J. S-14-03

Wilson, C. S-08-04, A01, C09

Wilson, E. S-03-04, B35

Wilson, W. S-15-01

Winckler, B. PRES-03

Wirianto, M. B19

Wohlfert, E. A24

Wolfe, L. C42

Wong, S. A46

Wood, A. A06

Wood, T. B31

Wouters, K. B02

Wrabetz, L. D32

Wu, C. C52

Wu, G. B37

Wu, L. C-04-04

Wu, M. OR-02-01, D40

Xie, A. A08

Xie, L. C47

Xiong, L. C52

Xu, H. D07

Xu, Z. S-10-03

Yamada, K. OR-01-01, C51

Yang, J. S-08-03

Yaqubi, M. OR-04-04, C08

Ye, Q. OR-02-03, B19, D50

Yi, J. C38

Ying, M. C06

Yoo, S. B19

You, F. OR-02-04, D18

Young, A. C23

Yu, D. OR-02-04, A46, D18, D51

Yu, J. C-01-04

Yu, M. C42

Yu, R. C18

Yu, X. C-01-02

Yuede, C. A50, C17

Yuen, T. S-15-04

Yugar, B. OR-02-02, C46

Zebarth, J. OR-02-04, D18

Zehra, S. C39

Zeldich, E. B30, D16

Zepeda, R. B03

Zerimech, S. D52

Zhang, A. D23

Zhang, H. A09

Zhang, J. C23

Zhang, L. A52, C56, D48

Zhang, N. D20, D35, D48

Zhang, Y. S-14-01

Zhang, Z. A52, D20, D48

Zhao, J. C32, C38, D01

Zhao, L. B28

Zheng, P. A42

Zhou, A. OR-02-01, D40

Zhou, E. A17

Zhou, S. D24

Zhou, Y. C-02-03

Zhu, J. A53

Zhu, X. A53

Zhu, Y. D19

Zhu, Z. B41, C54, C56

Ziobro, P. D26

Zorec, R. C-03-03, D14

Zorumski, C. C36

Zuo, Z. B19

## Keyword Index

Abeta S-13-01, B41, B52, C17, C50

Actin D21

Activated astrocytes D14

Acyl-CoA A45

Addiction and drugs of abuse S-06-01, S-06-02, S-06-03, S-06-04, A49

Adenosine S-10-01, A13, A14, A33

Adenosine kinase A13, A14

Adenosine receptor S-10-01, A33

Adhesion C21

Adrenergic C-03-03

Aggregation S-01-02, S-01-04, B42, C17, D15

Aging C-04-01, A15, A32, A37, C27, C56

Alcohol A52

Allopregnanolone C36

Allosteric S-15-03

Alpha-synuclein S-01-03, A19, C18, C49

Als-pdc A20, B04

Alzheimer's disease PRES-04, S-02-03, S-02-04, S-13-01, OR-01-02, A18, B02, B23, C50, C52, D16, D52

AMPK D39

Amygdala S-05-01

Amyloid PRES-04, S-01-03, S-01-04, S-13-04, OR-01-02, A07, A19, B02, B23, B41, B42, B52, C17

Amyloid beta B41, B52, C50

Amyotrophic Lateral Sclerosis D15

Animal models A17, B44

Animal models of neurological disease C-02-02, C-06-01, A17, A33, C17, C33, C42

Animal models of psychiatric disease S-06-01

Anti-inflammatory D10

Antidepressants S-12-02, S-12-04, A31

Antioxidant A30, D49

ApoE OR-01-01, C51

Apolipoprotein OR-01-01, C51

Apoptosis A27, B15, PS-B-45

Arachidonic S-02-01, S-02-02

Astrocyte S-03-04, S-05-03, S-07-04, S-08-01, S-08-03, S-09-01, S-09-02, S-09-03, S-09-04, S-11-04, S-13-04, S-14-01, S-14-02, S-14-03, S-14-04, C-01-01, C-01-02, C-01-04, C-03-01, C-03-02, C-03-03, OR-01-02, OR-01-03, OR-02-01, OR-02-03, OR-02-05, OR-03-01, A06, A09, A14, A18, A21, A22, A37, A38, A40, A41, A44, A53, B09, B10, B23, B35, B40, B48, B49, C29, C44, C54, C56, D09, D11, D14, D20, D27, D29, D30, D34, D39, D40, D42, D43, D45, D46, D50

Astrogliosis A14, B14, C29, D35

Ataxia B28

Autism S-09-04, S-12-01, B39, C15, C35, C38, D26, D27, D31

Autism spectrum disorder C35, D31

Autoimmune C-07-03, A35, B20, C42, D13

Autoimmunity S-14-03, C-06-01, B37, D07

Autophagy C-01-03, A03, A53, B04, C19, C45

Axon S-11-01, C-06-03, C-07-02, C-07-03, OR-01-05, B01, B15, B20, B51, C25, C28, D04, D24, D52

Axon degeneration B20

Axon function D52

Axon growth and guidance OR-01-05, A37, C07, C25

Axon initial segment B01

Axonal C-07-03, OR-04-05, A16, C42

Axonal degeneration C-07-03

Axonal injury OR-04-05, A16, C42

Axonal transport C-07-03

Bacteria S-04-03, C16, C40, D41

Basal ganglia C-04-01, B51

BDNF S-06-04, S-09-03, C-03-01, A41, A46, D03

Behavior OR-04-03, A32, A43, A45, A49, B36, PS-B-45, C15, C24, C27, C28, C33, D19, D25, D47

Bergmann glia B05, D39

Bergmann glia cells B05

Beta-amyloid S-13-02, B42

Biogenic amines A30

Bioinformatics C56, D16, D41, D46

Biological Rhythms A40

Blood S-07-02, OR-02-03, OR-03-01, B48, D50, D51

Blood-brain barrier S-04-01, S-07-02, C-06-01, OR-01-02, OR-02-03, B23, C43, D13, D45, D50

Brain Development S-05-01

Brain protection D35

C9orf72 A20

Calcium C-03-02, C-03-03, C-07-01, A23, A26, A27, A42, C03, C44, D19

Callosum D31

Calpain A12, B44, D22

CAMP C-03-03

Cardiac C39, D07

Caveolin-1 D13

Cell death S-14-02, A27

Cell death and survival D21

Ceramide S-02-04, S-13-03, S-13-04, B02, B26, B28, B41, C54, C56

Cerebellum C11, C14, C41

Cerebral S-11-02, A18

Cerebrovascular S-11-02, OR-02-04, D18

Chaperone S-01-02, S-01-03, S-01-04, D23

Charcot C32

Chemokine C-06-01, C40, D13

Chemotaxis D44

Cholesterol S-15-01, C-02-01, C-02-03, B31, C53

Cholinergic B15, D47

Cilia C54

Circadian Rhythms A40

Circuits B24

CNS myelination S-05-04, C-02-01, D08

Cognition S-05-03, A15, A32, A33, C20, C52

Cognitive S-05-03, A14, A19, A28, A43, B03, B47, C13, C20, C24, C27, C52

Collagen XIX C12

Communication OR-03-01, B48

Complement S-13-02, OR-04-05, A16, A24, B50, D12, D44

Conditional knockout A18

Connexin A44, D23

Cortical control of movement A40

CRMP2

S-10-02, C-07-01, C-07-02, C-

07-03

Cuprizone C-05-02, A33, B09, B18, C29, C31

Cx43 A44

Cytokine S-04-02, S-04-03, S-11-04, OR-02-03, A29, B14, B49, C16, C44, D50

Cytoskeleton S-05-04, C47

Degradation PRES-03, C32

Delivery S-07-01, C12, D22

Dementia C52

Demyelination S-03-01, S-07-01, C-02-02, C-02-03, C-05-02, C-06-02, C-07-03, OR-03-02, OR-03-04, A12, A26, A33, A34, A35, A36, A48, B17, B18, B20, B47, C03, C29, C31, C32, C42, C47, D01, D03, D04, D06, D23

Dendritic S-12-03, OR-03-03, A02, A31, C33

Depression S-04-01, S-04-02, S-04-03, S-12-02, S-12-03, A30, A31, A43, A46, B52, C43

Development S-05-01, S-05-02, S-07-01, S-09-03, S-09-04, OR-03-03, OR-03-05, OR-04-04, A02, A05, A41, B12, B19, B21, B31, B46, C08, C21, C34, C35, C38, D27, D32

Diet OR-04-03, B36, C33

Differentiation PRES-02, S-05-04, A05, B19, B30, B31, C10, C15, D28, D49

Diseases S-01-01, S-16-02, C31, C47, C49

Disorder S-06-01, C-02-01, C35, D31

DM20 B21

DNMT3 B22

Docosahexaenoic S-02-02, A45

Dominant C19

Dopamine S-06-03, B24

Dopaminergic B24

Drosophila OR-04-05, A16

Drug discovery B04

Dysfunction OR-02-02, A14, A49, B14, B43, PS-B-45, C46, D34

EAAT2

OR-03-01, B48

Elegans D47

Encephalomyelitis A25, A35, B37, C42, D13

Encephalopathy A29

Endocannabinoids S-02-01, OR-04-01, B27, B34

Endoplasmic OR-02-05, B01, D11

Endosome PRES-03

Endothelium OR-03-01, B48

Energy S-07-03

Enhancer B21

Ensheathment A24

Environment B46, D47

Environmental B10, B46, D47

Ephrin S-09-04

Epigenetic S-06-01, S-06-02, S-06-03

Epilepsy A10, A23, B40, C29, C37, D30

ERK1/2 A26

Estrogen A35, D22Excitatory S-09-04, C22, C24

Excitotoxicity S-08-03, A10, B22

Exocytosis C-01-01

Exosome S-13-01

Extracellular matrix A38, C12

Fibroblast S-09-01, D29

Fluoride B08

Forebrain B15, B32, C35

GABA S-15-02, B39, C36, D29

GAD67 B39

Ganglioside C18

GDNF D20

Gene expression, regulation S-06-04, OR-02-05, OR-04-02, OR-04-04, A25, A37, A39, B21, B24, B28, C05, C07, C08, C18, D11, D27

Gene Knock-Down A14

Genetics C11

GFAP S-16-02, C-01-04, A08

GLAST A47, B22, C05

Glial cells S-16-02, S-16-03, OR-01-03, A37, B06, B08, C05, C22, D09, D39

Glioblastoma A03, C09, C39

Gliogenesis D35

Gliosis OR-04-05, A14, A16, A23, B14, D10, D22, D35

GLT-1

OR-03-01, A47, A53, B48, D34

Glucose C-01-01, OR-04-01, A18, B34

Glutamate S-05-04, S-08-03, C-01-01, OR-03-01, A47, B05, B06, B08, B22, B39, B48, C05, C22, D03, D30

Glutamate transport S-05-04, OR-03-01, A15, B48

Glutamate Transporter S-05-04

Glutamate uptake A47

Glutamate/Glutamine shuttle B05

Glutamatergic OR-04-02, A39

Glutamine B05, B08

Glutathione A06, D49

Glycolipid C18

Glycosphingolipids OR-01-04, C02

Growth factor S-09-01

Herpes C47

Hibernation A13, B27

HIF-1 A04

Hippocampal A15, A43, B03, C36, D26

Hippocampus S-06-01, S-09-04, A15, A24, C14, D26

HIV-1

S-05-03, A49, C01, C27, C44

Homeostasis S-01-01, S-16-01, A22, D02

Hormone A48, C27

Hsp70

S-01-035-HT1A A43

Human S-01-03, S-12-02, S-14-01, OR-04-04, B06, B07, C08, C11, C35, C39, C47

Human oligodendrocyte progenitor cells C47

Huntington S-01-02

Huntington's disease C-01-02

Hydrolase A46, D51

Hypomyelination C23

Hypoxia S-14-01, A04, B32

IL-6 C15, C44

Imaging S-12-02, S-12-03, C-04-02, C-04-04, A21, A45, C55

Immune system S-05-01

Immunocytochemistry B07

Immunohistochemistry B51

Immunoregulation S-04-01, S-07-01, B44, C16

Inhibition A08, A27, C36, D26

Inhibitor B13, B26

Inhibitory S-09-04, S-11-04, A24, B14, B39, C12, D26

Insulin A19

Insulin signaling A19

Integrin C-06-04

Intercellular A44

Interferon-gamma C16

Interleukin C15

Intracellular S-11-03, C-03-02, OR-03-03, A02, B52, C03, D15

Intracellular signaling pathways C-03-02, OR-03-03, A02, A19

Ion channels S-08-01, S-08-04, S-10-02, S-15-02, C-07-01, A01, C09

Ion transporter S-08-02

IPSC C06

Iron S-07-01, S-07-02, S-07-03, S-07-04, B10, B43, C35, D08

Iron deficiency S-07-03, C35

Ischemia B04, PS-B-45, C26, D20, D21, D52

Ischemic OR-02-03, OR-02-04, D18, D20, D21, D35, D46, D50, D52

Jak/stat OR-02-05, D11

KAINIC C24

Ketogenic OR-04-03, B36

Kinase OR-02-05, A13, A14, D11

Kinesin C-07-03

Knockdown A14

Knockout B39, B50

Lactate C-01-01, C-03-03

Laminin A38

Lanthionine C-07-02, B04

Learning S-09-04, B49

Learning and memory S-09-04, A43, B49

Leukemia inhibitory factor S-11-04, B14

Leukocyte C-06-01, C-06-04, C40

Leukodystrophy S-08-01, C-02-02, C-05-01, C-05-03, C06

Linoleic acid A46

Lipidomics S-02-03, C55

Lipoxygenase OR-02-04, D18

Lithium S-11-02

Locus Coeruleus C-03-01

Long-term potentiation PRES-04, A32

Lymphocyte C-06-04

Lysosomal PRES-03, D19

Lysosome B43

Macrophage S-03-02, C-04-03, A07, A25

Manganese C41, D39

MAPK B06

Mapping D41

Mass spectrometry S-15-02

Matricryptin C12

Matrix A38, C12

Maturation OR-03-01, B48, D27

MeCP2 D27

Membrane-associated PRES-03, C-01-04, D14

Metabolic C-01-01, C-03-03, C-03-04

Metabolism S-07-04, S-14-01, C-01-01, C-02-02, C-03-03, C-03-04, OR-01-01, OR-02-03, A15, A45, C51, D50

Metabotropic D03

Metabotropic glutamate receptor D03

Metals B10, D47

Methods in neurochemistry S-15-02

Microbiome B50, D41

Microglia S-03-01, S-03-02, S-03-03, S-05-01, S-05-02, S-05-03, S-13-01, S-15-04, C-04-01, C-04-02, C-04-03, C-04-04, OR-01-01, OR-01-03, OR-04-01, OR-04-03, OR-04-04, A09, A12, A17, A24, A25, B12, B17, B18, B34, B36, C08, C26, C40, C41, C51, D09, D43, D46

Microgliosis B14

MicroRNA PS-B-45

Microtubule C-07-02

Mitochondria S-07-03, S-13-04, S-14-04, OR-02-01, A35, B01, B12, B43, D40

Mitochondrial dynamics A35

Mog-eae C42, D03

Motoneuron D48

Mouse S-09-02, S-11-04, S-12-02, S-14-01, S-16-01, C-03-02, C-04-03, OR-02-02, A01, A08, A31, A33, A48, B11, B32, B50, C11, C18, C37, C46, D03, D08

Mutagenesis C-02-03

Myelin PRES-02, S-05-04, S-07-03, S-07-04, S-08-01, S-11-01, S-11-03, S-15-01, S-15-04, C-02-01, C-02-02, C-02-03, C-05-01, C-05-02, C-07-03, OR-01-01, OR-01-04, A12, A26, A33, B01, B11, B17, B18, B20, B21, B31, B46, C01, C02, C21, C23, C29, C31, C51, C53, D02, D03, D04, D07, D08, D16, D22, D25, D31, D32, D49, D52

Myelin basic protein C23

Myelin proteolipid protein B21

Myelin repair OR-01-04, C02, C21, D07

NADH D48

NADPH OR-04-01, B34

Nanoparticle C20, D22

Neonatal A05, C12, C15, D28

Nerve B20, D24

Neural Stem Cells OR-03-02, OR-03-04, A34, B49, D06, D28, D44

Neurochemical Anatomy B38

Neurodegeneration S-01-02, S-01-04, S-02-03, S-02-04, S-08-02, S-11-01, S-15-03, S-16-02, C-01-03, C-04-03, OR-01-01, OR-02-04, A12, A27, A28, A35, A52, B15, B16, B18, B20, B43, B44, C42, C44, C48, C49, C51, D18, D20, D24, D47, D48, D51

Neurodegeneration S-01-02, S-01-04, S-02-03, S-02-04, S-08-02, S-11-01, S-15-03, S-16-02, C-01-03, C-04-03, OR-01-01, OR-02-04, A12, A27, A28, A35, A52, B15, B16, B18, B20, B43, B44, C42, C44, C48, C49, C51, D18, D20, D24, D47, D48, D51

Neurodegenerative S-01-04, S-05-03, C-01-03, B03, B20, B44, C50, C52, D19

Neurodegenerative disease S-01-04

Neuroendocrine Regulation C-01-04, A42, A48

Neuroimmunology S-03-02, S-03-03, S-04-02, S-14-02, C-04-03, C-06-02, C-06-03, OR-04-05, A16, A36, C13, D13

Neuroinflammation S-10-01, S-10-04, S-13-02, S-13-03, S-14-02, S-14-03, S-16-01, S-16-03, C-06-02, C-06-03, OR-01-03, OR-02-05, OR-04-01, A09, A10, A12, A14, A17, A22, A23, A45, B02, B34, B35, B37, B44, PS-B-45, C13, C14, C15, C40, C41, C43, C56, D09, D11, D14, D44

Neuronal activity C-04-04

Neuronal death D20, D22

Neuronal morphology C33

Neuronal-glial interaction S-05-03, S-08-04, S-11-01, S-16-01, S-16-03, C-03-04, C-04-01, C-04-04, C-05-03, OR-04-05, A08, A16, A24, C07, C23, C55, D20

Neuropathology C-06-02, B24

Neuropathy C-02-03, D01, D23

Neuropeptides A50, A51

Neuropilin C-07-01, D26

Neuroplasticity C-03-04, A32, B52

Neuroprotection S-14-03, S-14-04, A45, PS-B-45, C45

Neuroprotective S-11-01, S-11-04, A29, A30, B16, PS-B-45, C45, D24, D35

Neuropsychiatric Diseases and Models C-03-04, OR-04-02, A39, A43

Neurosteroids S-15-02, A32, C36

Neurotoxicity B06, B10, B16, B41, C22, D47

Neurotransmitter receptors A42

Neurotransmitter storage+release B51

Neurotrauma S-11-04, B14

Neurotrophic S-11-04

Neurotrophin B15

Neurovirology S-03-03, A25

NF-kB OR-04-01, B34, C33

Nitric Oxide S-10-03, C26

NMDA S-10-03, OR-04-02, A39, A42, D21

NMDA receptors OR-04-02, A39

NMDAR S-10-03, S-12-02, OR-04-02, A39

Nogo C-07-03, B09

Noradrenaline C-03-03

Norepinephrine C-03-01, C-03-02, C-04-04

Nrf2 D49

Olfaction B39

Oligodendrocyte PRES-02, S-05-04, S-07-01, S-07-03, S-07-04, S-11-03, S-15-01, S-15-04, C-02-01, C-05-03, OR-01-01, OR-01-04, A04, A05, A26, A33, B11, B19, B30, B31, B32, B46, B47, C01, C02, C04, C23, C51, D07, D08, D49

Oligodendrocyte differentiation PRES-02, B19

Oligodendrocyte progenitor A04, B32

Oligodendrogenesis OR-01-05, OR-03-02, OR-03-04, A05, A34, C25, D06

Oligodendroglial A36, C10, D31

Opioid S-10-03, S-10-04, S-12-02, A49, A50, A51

Optic B20, D24

Oxidative S-14-01, OR-04-01, A06, B03, B10, B34, D47, D49

Oxidative stress S-14-01, A06, A30, B03, D49

P75NTR A05, B15

Pain and inflammation S-10-03

Paranode B11

Parkinson's disease C-01-03, A19, A53, B44, C45, D42

Parvalbumin C12, C29

Pathogen-associated D41

Pathogenesis B43, D42

Pelizaeus-Merzbacher Disease C-05-01

Peroxidation S-02-02

Phagocytosis OR-04-05, A16

Pharmacology C52

Phospholipids S-02-01, A45

Phosphorylation B062-photon S-03-02, S-03-03, C-04-04, C-05-02

Plasma S-13-02, A19

PMP22 C-02-03, C53, D01

Potential S-10-02, C47, D23

Precursor B31

Presenilin C19

Primate A17

Primates A17

Profiling D46, D48

Progenitor A04, A05, B32, C15, C47

Progesterone A32

Proliferation OR-03-02, A05, A34, C09

Prostaglandin D10

Proteasome S-01-04

Proteolipid C-05-01, B21

Proteolipid protein C-05-01, B21

Proteolysis C19

Proteomic C19

Protofibrils A07, B42

PUFA B27

Purinergic A13

Purines S-10-01

Purkinje A35, B28

Rac1 D32

Radial B06

Reactive astrocyte B35

Reactive oxygen species B43, D22

Redox B03, C10

Remyelination S-03-01, S-07-04, S-15-01, S-15-04, C-05-01, C-05-02, OR-01-04, OR-03-04, A33, B17, B19, C02, C04, C21, C31, D04, D06

Respiration S-07-03, C32

Reticulum OR-02-05, B01, D11

Retina S-09-01, C-04-03, B06, B08, D24, D29, D43

Rett C33

Rett syndrome C33, D27

Risk S-15-04, D10

S100B D10

Scar OR-01-05, C25

Schizophrenia OR-04-02, A39, B24, C12

Schwann C-02-03, C03, C21, D01, D02, D32

Sclerosis S-02-03, S-14-03, S-15-04, C-03-01, C-06-01, C-06-04, OR-03-02, A12, A33, A34, A36, B11, B18, B19, D07, D13, D15, D41

Screening A04, A07

Seizure threshold D30

Seizures S-08-04, A01, C24, C29, D26, D30

Semaphorin OR-03-03, A02

Serotonin S-12-01, S-12-04, A30

Serum D51

Sex difference S-05-03

Shock D23

Signal transduction S-10-02, S-11-03, C-07-01, D39

Social S-12-01, OR-04-03, B36

Social behavior S-05-01

Social defeat stress OR-04-03, B36

Sonic Hedgehog S-09-02, B30, D29

Space PRES-03

Species S-02-02, B43, D22

Spectrometry S-15-02

Spectroscopy C53

Sphingomyelin B02, B26

Sphingosine S-13-03, OR-01-04, B28, C02

Sphingosine-1-phosphate S-10-04, OR-01-04, C02

Spinal OR-01-05, A27, B01, B31, B37, C25, C42, D01, D04, D22, D44

Spinal cord injury OR-01-05, A27, C25, D04, D22, D44

Spine S-09-02

Stem Cell Biology B30

Stress S-04-01, S-04-02, S-04-03, S-05-02, S-14-01, OR-02-05, OR-04-03, A06, A31, A42, A43, A49, A52, B03, B36, C43, D11, D48, D49

Stress-induced behaviors S-04-01, S-04-02, OR-04-03, A49, B36

Striatum C-01-02, A21, B51

Stroke S-08-03, S-14-04, OR-01-03, OR-02-03, PS-B-45, D09, D20, D35, D46, D49, D50, D51

Structure OR-02-01, A44, B01, B38, C55, D40

Subventricular OR-03-02, A05, A34

Sulfatide B11

Swelling S-08-03, S-08-04, B40

Synapse S-09-02, S-09-03, S-09-04, C-04-02, A24, A38, A41, B09, B38, C11, C37

Synaptogenesis S-12-04, B09, C12

T-cells C13, D13

Tamoxifen D45

TDP-43 D15

Thermogenesis B27

Thermoregulation B27

TLR4 A07

Tolerance S-10-04, A18, D07

Toxoplasma S-03-04, A24, B24

Tracing B51

Trafficking C-07-02

Transcription S-04-01, C-02-01, OR-02-02, OR-03-03, OR-04-04, A02, B22, C08, C46

Transcription factor C-02-01

Transcriptome OR-04-04, A37, C08

Translation OR-01-02, OR-03-03, A02, B23, D39

Traumatic brain injury S-11-02, S-11-03, A09, B14, B15, D45

Trisomy D16

Tubulin C-05-03, C54

Tumor C09

Tutorial B38

Tyrosine A38, C18

Ubiquitination S-15-03, B19, C10

Ultrastructure OR-02-01, C28, D40

Valproic acid D31

VEGF A08

Vesicles S-13-01, S-13-02, A19, A20, A36, C31, C48

Viral infection S-14-02, A23, A25

Viruses S-03-01, S-14-02, C-06-02, OR-02-02, OR-03-05, A23, A29, B49, C34, C46, C47

Visual S-09-01, C-03-02, OR-03-05, C34, C42, D25, D29

Visual Cortex C-03-02

Vitamin C33

White matter injury S-11-01, S-11-04, B195xFAD S-13-03

Zebrafish C-02-02, D19, D25

